# Speech-based digital biomarkers for early etiological stratification of Alzheimer’s disease and frontotemporal degeneration: a biomarker-confirmed prospective study

**DOI:** 10.1016/j.tjpad.2026.100573

**Published:** 2026-04-17

**Authors:** Eloïse Da Cunha, Valeria Manera, Frédéric Chorin, Justine Lemaire, Alexandra Plonka, Aurélie Mouton, Raphaël Zory, Auriane Gros

**Affiliations:** a Université Côte d’Azur, Speech and Language Pathology department of Nice, Faculty of Medicine, Nice, France; b Université Côte d’Azur, COBTEK Laboratory (Cognition Behaviour Technology Laboratory), Nice, France; c Université Côte d’Azur, Interdisciplinary Institute of Artificial Intelligence Côte d’Azur (3IA Côte d’Azur), Sophia Antipolis, France; d Centre Hospitalier Universitaire de Nice, Clinique Gériatrique du Cerveau et du Mouvement, Nice, France; e Université Côte d’Azur, LAMHESS (Laboratoire Motricité Humaine Expertise Sport Santé), Nice, France; f Institut Universitaire de France (IUF), Paris, France

**Keywords:** Alzheimer’s disease, Frontotemporal lobar degeneration, Primary progressive aphasia, Digital biomarkers, Speech analysis, Machine learning, Early detection, Prevention

## Abstract

**Background:**

Early differentiation between Alzheimer's disease (AD) and frontotemporal lobar degeneration (FTLD) is a prerequisite for secondary prevention and targeted trial enrollment, yet remains challenging at disease onset. We investigated whether automated speech analysis could serve as a digital biomarker for early etiological stratification across clinically heterogeneous presentations.

**Methods:**

In this prospective biomarker-confirmed prognostic study, 172 participants (108 patients with biomarker-confirmed AD or FTLD and 64 controls) completed a standardized speech protocol at initial clinical assessment. Acoustic, temporal, and phonatory features were automatically extracted. Machine learning models and a stacking ensemble were trained using stratified, repeated 5-fold cross-validation to discriminate between AD and FTLD pathology, with exploratory analysis extending to atypical and rare phenotypes crossed with physiopathology, including primary progressive aphasia (PPA) variants.

**Results:**

Speech-based models achieved high sensitivity and specificity in distinguishing physiopathology independently (mean area under the curve (AUC)=0.986) and crossed phenotype and physiopathological diagnostic association (mean AUC=0.966).The ensemble identified 82% of cases with clinicopathological discordance. Interpretability analyses revealed distinct speech signatures: AD was associated with global speech slowing and phonatory instability, while FTLD was characterized by reduced verbal output and acoustic hypo-expressivity.

**Conclusions:**

Automated speech analysis provides a promising non-invasive digital biomarker for the early etiological stratification of AD and FTLD, including atypical phenotypes, with high accuracy in a monocentric biomarker-confirmed cohort. These findings support the feasibility of speech-based etiological stratification and its potential to complement existing biomarker frameworks, particularly in cases of clinicopathological discordance. External validation is required before clinical deployment can be considered.

## 1 Background

The potential for secondary prevention and early intervention in neurodegenerative diseases is currently limited by a fundamental issue: the inability to accurately identify the underlying pathology at the earliest, most treatable stages. This clinicopathological dissociation is particularly prominent at disease onset, when symptoms remain subtle and heterogeneous, yet when therapeutic interventions would be maximally effective. Indeed, these conditions exhibit marked clinical heterogeneity, with patients presenting distinct phenotypes despite sharing a common pathological substrate [1,2]. With the growing societal burden of neurodegenerative disorders, developing scalable tools for early pathological stratification has become a critical public health priority for enabling preventive neurology.

Alzheimer's disease (AD) and Frontotemporal Lobar Degeneration (FTLD) may manifest as either amnestic, behavioral syndromes or language-led phenotypes, presenting as primary progressive aphasia (PPA) [3]. The consensual PPA variants, logopenic (lvPPA), non-fluent (nfvPPA), semantic (svPPA) or mixed variants appear as primary presentation of the neurodegenerative pathway. While lvPPA predominantly associates with AD, nfvPPA and svPPA typically reflect FTLD-spectrum pathologies [4,5]. However, clinicopathological discordances are frequent and severely complicate diagnosis, prognostic stratification, and access to targeted therapies [6]. The need for accurate and early etiological classification has become increasingly crucial with the development of disease-modifying therapies (DMTs). Particularly, anti-amyloid treatments for AD require initiation in early disease stages to maximize efficacy and safety [7]. Consequently, identifying the underlying pathophysiology independently of overt clinical phenotype is now a prerequisite for enrolling the right individuals into targeted secondary prevention trials and deploying approved therapies at a therapeutically actionable stage [8].

Current diagnostic frameworks remain insufficient to resolve this ambiguity promptly at the population level. These frameworks rely on clinical criteria, neuroimaging, and invasive biomarkers such as Cerebrospinal Fluid (CSF) analysis [3,9]. These approaches face limitations including protracted diagnostic timelines, limited accessibility, invasiveness, and insufficient sensitivity at onset time or for atypical presentations [10]. Moreover, conventional biomarkers provide largely static diagnostic confirmation, offering limited insight into disease dynamics or phenotypic evolution [11]. In complex cases, longitudinal clinical follow-up remains the de facto standard, delaying definitive diagnosis and therapeutic decision-making [12].

Connected speech, defined as continuous, naturalistic language production and ecological measure beyond isolated words or single‑sentence utterances*,* has emerged as a promising marker for dynamic profiling of neurodegenerative progressions [[13], [14], [15]]. Subtle alterations in prosody, syntax, lexical retrieval, and pausing reflect neuropathological substrates [16]. Machine learning approaches can quantify these features with high granularity, achieving significant accuracy in semiological classification [17]. However, a critical gap persists: most studies rely solely on clinical diagnoses, and none have validated speech biomarkers for predicting disease-specific progression in longitudinal, biomarker-confirmed cohorts [18]. Importantly, speech alterations may precede formal diagnosis, highlighting their potential for early risk stratification at a stage amenable to preventive intervention strategies [19,20].

In this study, we hypothesized that computational features extracted from connected speech at initial clinical evaluation could serve a dual purpose. Our primary objective was to determine whether speech‑derived markers can robustly discriminate the underlying neurodegenerative pathophysiology (AD versus FTLD), independent of clinical phenotype. As a secondary and exploratory objective, we examined whether these same features carry sufficient information to distinguish clinical syndromes within each pathological category, including PPA variants and behavioral or amnestic presentations. To address the limitations of prior work and provide the robust longitudinal evidence required for prevention biomarker development, we employed a prospective‑retrospective design: patients were recruited prospectively and completed the speech protocol at initial clinical presentation, while final etiological classification was established after up to four years of longitudinal follow‑up and comprehensive biomarker evaluation. By anchoring speech analysis to gold‑standard etiological classification, this study evaluates speech as a scalable digital biomarker capable of enabling early etiological stratification, with direct implications for participant selection in prevention trials and pre‑symptomatic interception.

## 2 Methods

### 2.1 Ethical considerations

The study protocol was approved by the relevant institutional ethics committee for healthy patients (*Comité de protection des personnes* (CPP) *Est II* (N°2021-A02986–35) and for pathological patients (CPP Ile de France X, N° IDRCB: 2019-A00322–55). All procedures were conducted in accordance with the Declaration of Helsinki, and all participants gave written informed consent. All data were anonymized and handled securely in compliance with institutional data protection regulations.

### 2.2 Study design and participants

This observational study was conducted at the University Hospital Center of Nice, France. Adopting a design critical for validating early-detection tools, the protocol featured a prospective recruitment of patients with a retrospective analysis of final diagnoses, which were confirmed by a longitudinal follow-up extending up to four years (2020–2025): patients were enrolled at initial clinical presentation when speech data were collected, whereas the reference standard diagnoses were established retrospectively through a multidisciplinary consensus after up to four years of longitudinal follow-up and biomarker confirmation. This design ensured that speech recordings were acquired at an early clinical stage, prior to diagnostic stabilization, while final etiological classification relied on extended clinical and biomarker evaluation. This prospective diagnostic adjudication is essential to establish the validity of biomarkers for early, pre-intervention stratification.

The cohort comprised patients over 40 years old presenting with cognitive complaints and receiving a definitive diagnosis of either AD (amnestic or primary progressive aphasia presentations) or FTLD spectrum disorders (behavioral variant or primary progressive aphasia variants). Inclusion criteria for all participants required native French language proficiency and the provision of informed consent. Exclusion criteria encompassed major neurological or psychiatric comorbidities, psychoactive medication use, and uncorrected sensory impairments [21]. Importantly, diagnostic adjudication was performed blind to speech-derived features. Patient diagnoses were established at the end of the longitudinal follow-up period (2020–2025) through multidisciplinary consensus, integrating standardized clinical and neuropsychological evaluations, structural and functional neuroimaging (MRI and FDG-PET), and CSF biomarker profiling [3,9]. All speech-related data processing and machine learning analyses were conducted independently, without access to the final diagnostic labels. This consensus diagnosis served as the reference standard for all analyses.

A separate cohort of healthy control participants (N = 64, ≥60 years old) was recruited under a distinct registered protocol (NCT05323286) designed for a physical activity intervention in community-dwelling older adults. From this source population, we selected only individuals who reported no cognitive complaints, achieved a Mini-Mental State Examination (MMSE) score greater than 27, and screened negative for significant psychiatric symptoms using two questions from the ICOPE (Integrated Care for Older People) screening tool to exclude conditions known to affect speech parameters in older adults. In line with real-world clinical practice, healthy controls did not undergo biomarker assessments, an approach consistent with both ethical standards and translational applicability.

### 2.3 Diagnostic criteria and clinical characterization

Patients were classified according to established international criteria. For PPA variants, all patients exhibited progressive language impairment as the core clinical feature [3]. Diagnosis of lvPPA was based on impaired single-word retrieval and sentence repetition deficits, with preserved single-word comprehension. nfvPPA was characterized by effortful speech, while svPPA presented with impaired confrontation naming and single-word comprehension. Neuroimaging confirmation was mandatory, requiring variant-specific patterns of atrophy or hypometabolism. Imaging protocols included MRI and FDG-PET scans interpreted by expert neuroradiologists. Objective quantification of linguistic profiles was performed using a standardized battery of tests including the GréMots battery [22], the *Pyramid Palm Trees Test* (PPTT) [23] and *DTLA - Dépistage des troubles du langage chez l'adulte et la personne âgée* (French screening for language disorders in adults and seniors) [24]. Amnestic AD (aAD) was diagnosed according to DSM-5 criteria for major neurocognitive disorder due to Alzheimer's disease, with biomarker confirmation adhering to NIA-AA guidelines using CSF Aβ42/40 ratio and p-tau cut-offs established in the national expert laboratory [9,25]. Behavioral variant of FTLD (bvFTD) diagnosis required progressively deteriorating behavior and/or cognition, supported by neuroimaging demonstrating predominant frontal and/or anterior temporal involvement [26]. The distinction between AD and FTLD was determined exclusively by crossed biomarker-supported etiological classification and not by clinical phenotype alone, ensuring that speech-based analyses targeted underlying pathology rather than surface-level clinical labels.

Throughout the multidisciplinary consensus process, each case was systematically examined for potential clinicopathological discordance. A patient was considered to present such a discordance if, during the diagnostic process, the clinical syndrome (amnestic, behavioral, linguistic, etc.) was not in agreement with the biomarker-confirmed underlying pathology (AD or FTLD), or if there was inconsistency among the multimodal biomarkers themselves (MRI, PET, CSF, clinical assessment). These discordances were identified independently of any speech-derived information. The final diagnosis assigned by the multidisciplinary team after integrating all available longitudinal and biomarker data was used as the reference standard for all analyses, regardless of the presence or absence of discordance. For patients with clinical-pathological discordances, the final diagnosis made by clinicians following multimodal and longitudinal clinical evaluations was used for the analysis. Only patients with a confirmed final diagnosis were included in the study. Patients with a mixed or unconfirmed diagnosis were not included. All participants presenting a pathology underwent a standardized assessment that further evaluated memory using the Cued Selective Reminding Test (FCSRT) [27], executive functions with the Frontal Assessment Battery (FAB) [28], and functional abilities (Instrumental Activities of Daily Living scale; IADL) [29]. This rigorous, biomarker-anchored diagnostic framework provides the gold-standard etiological classification required to train and validate tools for early pathological stratification, a prerequisite for targeted prevention strategies.

### 2.4 Speech protocol and data acquisition

Speech data were acquired under acoustically controlled conditions in a quiet, isolated room using a standardized and reproducible acquisition protocol applied identically across all participants. All recordings were obtained using a tablet device capturing stereo audio through two integrated microphones, configured for lossless recording in .wav format. The tablet was positioned on a fixed stand at a constant distance of 30 cm from the participant’s mouth to minimize inter-subject acoustic variability. No adaptive or subject-specific recording parameters were used. The same speech assessment was performed for both healthy control and AD-FTLD patients. Recordings were obtained in a standard hospital outpatient consultation room, under conditions intentionally designed to be replicable in routine clinical settings: a consumer-grade tablet on a fixed stand, no specialist acoustic equipment, and a quiet room available in any memory clinic or geriatric consultation. Total protocol duration was less than 10 min.

Participants produced spontaneous speech through two emotionally valenced monologue tasks involving the description of negative (NEG) and positive (POS) autobiographical life events. Participants were instructed to speak for one minute about a positive and negative event that had occurred in their lives. For the negative speech task (NEG), participants were explicitly instructed: 'Please speak for one minute about a negative event that occurred in your life' For the positive speech task (POS), the instruction was: 'Please speak for one minute about a positive event that occurred in your life' (French version: *Je vous demande de parler pendant une minute de quelque chose de négatif/positif qui s’est passé dans votre vie*.). Audio capture began immediately after the instruction to promote spontaneous speech production. To ensure standardized feature extraction across all participants, the recording duration was fixed at 60 s. The microphone continued recording for the full duration, even during periods of participant silence, to provide a consistent and ecologically valid sample that includes natural hesitation and planning pauses. This allowed for the computation of robust temporal features (e.g., speech rate, pause patterns) which are central to our analysis. Descriptive analysis of the final recordings confirmed that all audio files fell within a narrow range of 57 to 63 s (mean: 58.8 s, median: 58.5 s), reflecting minor experimenter‑related variability. Recordings exceeding 60 s were truncated, while those falling below were silence‑padded to exactly 60 s to ensure uniform input length.

The Sentence Span Test (SST) [30]. consisted of a spoken sentence repetition task designed to probe verbal working memory and phonological loop integrity [20]. The test included 14 sentences with progressively increasing phonological and morphosyntactic complexity, ranging from 3 to 9 content words. All verbal responses were digitally recorded and retained for downstream analyses.

For the maximum phonation time task (MPT), participants were instructed to sustain the vowel /a/ at comfortable pitch and loudness for as long as possible. At least two trials were collected, and the longest valid duration was retained for analysis to reduce intra-individual variability. To be included in the model development, patients had to realize all the speech protocol.

### 2.5 Data preprocessing and quality control

All acoustic features were extracted directly from the raw .wav audio files using a fully automated pipeline. The extracted parameters correspond to the complete set of acoustic and phonatory descriptors detailed in Supplementary Material 1, including spectral, prosodic, voice quality, and intensity-related measures. Feature extraction was implemented in Python using the Librosa library (v0.9.2) and applied identically to all recordings [31]. Data preprocessing is detailed in Supplementary Material 2.

Automated speech transcriptions generated by the Whisper OpenAI model were used exclusively for the extraction of temporal and alignment-based features, including speech rate, pause structure, articulation timing, and sentence-level temporal dynamics. Prior to deployment, a sensitivity analysis was conducted on a subset of recordings (N = 75) from our geriatric and pathological population: manual transcriptions produced by a certified speech-language pathologist were systematically compared to Whisper-generated transcriptions on the same recordings. This cross-validation yielded a mean Word Error Rate of 1.7%, with no clinically meaningful discrepancies, confirming the reliability and scalability of automated transcription in this specific population, including participants with language-related pathologies [32]**.** These transcriptions, associated with the corresponding audio file, served as the necessary input for the Montreal Forced Aligner. Temporal alignment was performed using the Montreal Forced Aligner with the pre-trained French-mfa acoustic model and dictionary (mfa package, v2.2.17), enabling precise word- and phoneme-level segmentation [33]. This forced alignment process provided the millisecond-precise phoneme and pause boundaries required to calculate all temporal and articulation-rate features.

All preprocessing steps were defined and frozen before model training. Feature normalization was performed using Z-score standardization (mean = 0, standard deviation = 1), with scaling parameters estimated exclusively from the training data and subsequently applied to the held-out test data to prevent information leakage (Supplementary Material 1).

### 2.6 Machine learning architecture

In this study, "Physiotype" refers to the underlying etiological pathology (AD or FTLD), irrespective of clinical presentation, while "Pathotype" refers to the clinico-pathological subtypes (e.g., amnestic AD, lvPPA-AD, nfvPPA-FTLD), that is to say, the crossed diagnosis of clinical phenotype and underlying physiopathology. These two levels of classification constitute the primary and secondary objectives of the machine learning analyses, respectively. We developed and compared two supervised machine learning frameworks for diagnostic classification, with the primary objective of validating speech-based prediction of the underlying pathophysiology at onset time (Physiotype: AD vs. FTLD). Model’s detailed architecture is described in Supplementary material 2. Importantly, all models were trained exclusively on speech-derived acoustic and temporal features extracted from the raw audio recordings. At no stage were clinical assessment scores, neuropsychological test results, neuroimaging data, or CSF biomarker values provided as input to the models. Diagnostic labels (Physiotype: AD or FTLD; Pathotype: specific clinico-pathological subtype) were used solely as supervised classification targets to guide model training, and played no role in feature extraction, preprocessing, or the cross-validation procedure. This design ensures that reported performance reflects the intrinsic discriminative capacity of speech acoustics, independently of any clinical or biological covariate. This core predictive task targets the early etiological differentiation necessary for secondary prevention and was then contextually analyzed in relation to the presented clinical syndrome.

The first framework was a hierarchical system that explicitly mirrored a two-step diagnostic reasoning process: the initial model specifically validated the prediction of the AD or FTLD physiotype, which then guided separate, subsequent classifiers to identify the clinical subtype (Pathotype: e.g., aAD, lvPPA, nfvPPA) within each resolved pathological branch. The second framework employed a flat multi-class model that performed a single-step classification of all Pathotypes and all Physiotypes independently. This approach served as a critical comparator to test the hypothesis that distinct speech signatures are intrinsically tied to specific clinical-pathological entities, and that direct classification of the syndromic-pathophysiological intersection is possible without prescreening for the underlying physiotype. This comparative design, illustrated in Supplementary material 2, allowed us to rigorously validate the accuracy of physiotype prediction as a standalone diagnostic step and to analyze the interplay between the clinical phenotype and the underlying pathology.

For each speech task, acoustic features were extracted directly from the raw .wav recordings and concatenated with temporal features derived from validated transcriptions (pause metrics, articulation rates, sentence-level timing). Prior to modeling, we systematically examined multicollinearity: pairwise Pearson correlations were computed across all features and Variance Inflation Factors (VIFs) were calculated. Features with VIF > 5 or exhibiting extremely high pairwise correlation (r > 0.95) were iteratively removed, with the feature having the higher mean absolute correlation to all other features removed first. This conservative, data-driven filtering was applied solely to the training dataset within each cross-validation fold to prevent information leakage, resulting in a final set of non-redundant input vectors for model training.

For each speech task independently (NEG, POS, SST, MPT), a fully separate model was trained on the corresponding task-specific feature set, using an independent database and codebase, ensuring complete separation between tasks at all stages of model development. Multiple algorithms were evaluated, including Logistic Regression (LR), Support Vector Machines (SVM), Random Forests (RF), Extreme Gradient Boosting (XGBoost), Gradient Boosting, Extra Trees, and k-Nearest Neighbors. Each model was trained using stratified repeated 5-fold cross-validation (5 repetitions) with nested randomized grid search for hyperparameter tuning. Bayesian correlated *t*-tests were used as the primary model selection framework, comparing cross-validated AUC distributions. A Region of Practical Equivalence (ROPE) of ±0.01 was defined: models were deemed superior if the posterior probability of outperforming competitors exceeded 0.95, and practically equivalent if entirely within the ROPE. Secondary metrics (Cohen's Kappa, logarithmic loss) guided selection only when models were practically equivalent.

A stacking ensemble was constructed by combining class probabilities from the best-performing task-specific models, with four candidate meta-models (SVM, Logistic Regression, Random Forest, Gradient Boosting) evaluated. To generate unbiased meta-features, base learners produced class probability predictions via 5-fold cross-validation on the training set, and these out-of-fold probabilities were concatenated to form the meta-feature matrix for training the final meta-classifier. The meta-classifier was subsequently evaluated on the same independently held-out test set as the base models, ensuring that stacking performance estimates are free from any form of optimistic bias. The predicted class was assigned as the label with the highest output probability from the model, consistently across all base models and the stacking ensemble. No custom decision threshold was applied.

### 2.7 Model validation and performance evaluation

To prevent data leakage and ensure unbiased performance estimation, the complete dataset (N = 172 participants) was randomly partitioned into a training set (80%) and a held-out test set (20%) before any feature extraction or preprocessing. Critically, the split was performed at the participant level, guaranteeing that all four speech recordings (NEG, POS, SST, MPT) from a given participant were assigned exclusively to either the training or the test set. The training/test split and all cross-validation folds were additionally stratified by age group and sex to ensure balanced demographic distributions across all partitions, preventing any demographic imbalance from confounding performance estimates.

All cross-validation and hyperparameter tuning were carried out solely on the training set. The held-out test set was used only once, after all model development and selection were finalized, to evaluate the final performance of the stacking ensemble.

To mitigate class imbalance, random oversampling of minority classes was applied. A sensitivity analysis comparing this strategy with class weight adjustment and no correction confirmed robust performance across all methods (Supplementary Material 3). Crucially, this resampling was performed exclusively on the training data within each fold of the cross-validation loop, after the train-validation split, ensuring that no synthetic samples from minority classes influenced validation fold estimates.

Model performance was evaluated using accuracy, precision, recall, F1-score, area under the ROC curve (AUC), log loss, Cohen's Kappa, and Matthews correlation coefficient. Multiclass AUC was computed using a one-vs-rest (OvR) strategy with macro-averaging, assigning equal weight to each class regardless of size, consistent with the equal clinical relevance of all pathotypes and providing a conservative performance estimate robust to class imbalance. For multi-class settings, false positive rate (FPR) and false negative rate (FNR) were defined and reported as macro-averaged one-vs-rest rates, computed as the mean across all classes of the per-class FPR and FNR respectively. To quantify uncertainty, 95% confidence intervals for accuracy, F1-score, and AUC were obtained by bootstrap resampling (1000 iterations) on the held-out test set.

To evaluate the ability of speech-based models to classify patients with clinicopathological discordance with the final validated diagnosis, a descriptive reclassification analysis was performed post-hoc on the held-out test set, after all model development and hyperparameter tuning were completed. Reclassification was defined as the model correctly predicting the final biomarker-confirmed etiology (AD or FTLD) in a participant whose clinical presentation was discordant with at least one conventional biomarker modality (MRI, PET, or CSF). The proportion of successfully reclassified discordant cases was calculated as the number of such participants in the test set for whom the model's prediction matched the biomarker-confirmed diagnosis, divided by the total number of discordant participants in the test set. This analysis is descriptive and exploratory in nature; it does not constitute a formal diagnostic accuracy comparison with established biomarkers.

### 2.8 Statistical analysis and model interpretation

Demographic and clinical variables were compared using Mann-Whitney U tests (continuous variables) and chi-square tests (categorical variables) to ensure baseline comparability. Model interpretability was achieved using SHapley Additive exPlanations (SHAP): TreeExplainer for tree-based models and KernelExplainer for others. SHAP values were computed on the best-performing base models, with feature contributions standardized to enable direct comparison across feature groups. To assess the potential influence of demographic factors on model predictions, preliminary SHAP analyses were conducted with age and sex included as candidate input features alongside acoustic variables. These analyses demonstrated that age and sex did not contribute meaningfully to the model's predictions (mean |SHAP| 〈 0.01 for both variables across all tasks, all FDR-corrected p 〉 0.05). Accordingly, demographic variables were excluded from the final feature sets to ensure that classification was driven exclusively by speech-derived acoustic markers. To validate the stability of discriminative patterns, pairwise Mann-Whitney tests were compared across physiotype and pathotype subgroups for all features with effect sizes (r), and p-values corrected for multiple comparisons using the FDR procedure. The significance rate corresponds to the proportion of features showing a significant pairwise difference (p < 0.05) after FDR. Corrected p-values are reported in Supplementary Material 4.

To further confirm the absence of demographic confounding, two complementary sensitivity analyses were conducted on the held-out test set. First, Spearman correlations between age and each of the top ten discriminative features were computed separately within each diagnostic Pathotype and Physiotype group. No significant age-feature correlation was observed after False Discovery Rate (FDR) correction (0 pairs per task across all four tasks for all classes). Second, a logistic regression model including age as an additional covariate alongside the top features (M2) was compared to a features-only model (M1). Feature coefficients remained stable across all tasks, and the addition of age did not significantly improve classification accuracy in any task, confirming that the identified acoustic markers discriminate pathological groups independently of age. Analogous analyses for sex were conducted using Mann-Whitney U tests within each diagnostic group with FDR correction. No significant sex-feature associations were identified. Furthermore, prediction errors on the held-out test set were not concentrated in any specific age group or sex category, confirming the absence of systematic demographic bias in model performance. Results of these analyses are reported in Supplementary Material 5.

All analyses confirmed that feature multicollinearity was controlled, ensuring that SHAP interpretations and model performance were not confounded by redundant predictors. The identification of stable, biologically interpretable speech signatures is a key step toward developing explainable digital biomarkers for potential use in clinical practice and future prevention studies.

## 3 Results

### 3.1 Characterization of the study population and diagnoses

#### 3.1.1 Demographic characteristics

The study cohort comprised 49 CE patients, subdivided into 30 aAD and 19 lvPPA with AD pathology (lvPPA-AD), 59 FTLD patients including 21 bvFTLD, 11 lvPPA with FTLD pathology (lvPPA-FTLD), 13 svPPA-FTLD, and 14 nfvPPA-FTLD, as well as 64 cognitively healthy controls (Table 1). Age differed significantly across subgroups, with svPPA-FTLD (63.5 ± 6.1 years) and nfvPPA-FTLD (64.2 ± 3.8 years) being younger than amnestic AD (72.0 ± 3.6; p = 0.006 and p = 0.009, respectively) and the overall FTLD group (p = 0.006). svPPA-FTLD patients were also younger than lvPPA-AD (70.4 ± 3.7; p = 0.010). A male predominance was observed in nfvPPA-FTLD (20% female), significantly lower than in lvPPA-AD (25%; p = 0.036) and the overall FTLD cohort (68%; p = 0.028). Handedness was predominantly right-handed across all subgroups (Table 1). Disease duration until diagnosis was shorter in logopenic variants, with lvPPA-FTLD (1.5 ± 0.7 years) and lvPPA-AD (1.8 ± 0.9) showing significantly shorter duration compared with other FTLD subtypes (2.4 ± 1.3; p < 0.001) and aAD (p < 0.001).

**Table 1:** Demographic and clinical characteristics.

Physiotype	Alzheimer’s Disease (AD)			Frontotemporal Lobar Dementia (FTLD)					Healthy Controls (N = 64)
Pathotype	Total (N = 49)	Amnestic (N = 30)	lvPPA (N = 19)	Total (N = 59)	Behavioural (N = 21)	lvPPA (N = 11)	svPPA (N = 13)	nfvPPA (N = 14)	Healthy Controls (N = 64)
Age (years)	71.5 ± 3.39	72.0 ± 3.56 *a*	70.4 ± 3.71 *b*	69.9 ± 4.62	71.2 ± 1.88	68.8 ± 4.83	63.5 ± 6.10 *a* *b*	64.2 ± 3.84 *a*	69.8 ± 15.74
Sex ratio (% F / % M)	41 / 59	52 / 48	25 / 75 *c*	68 / 32	27 / 73 *c*	29 / 71	54 / 46	80 / 20 *c*	54 / 46
Educational Level (P-S-E-Sup) in %	21–39–39	28–36–36	0–83–17	29–46–25	60–20–20	50–50–0	10–50–40	33–33–33	32–34–33
Handedness (% Right)	100%	100%	100%	100%	100%	100%	100%	100%	100%
MMSE	24.64 ± 2.99	23.08 ± 2.43	27.75 ± 1.54	25.13 ± 2.79	25.5 ± 1.50	25.5 ± 2.17	25.1 ± 3.88	27.6 ± 1.28	29.36 ± 0.75
Time since symptoms (years)	1.65 ± 0.74	1.6 ± 0.67 *d*	1.75 ± 0.88 *d*	2.36 ± 1.28	3.62 ± 1.24 *d*	1.5 ± 0.67 *d*	2.91 ± 1.90 *d*	2.38 ± 1.38 *d*	-
IADL	0.44 ± 0.57	0.58 ± 0.58	0.17 ± 0.31	0.37 ± 0.50	0.0 ± 0.0	0.17 ± 0.28	0.6 ± 0.48	0.4 ± 0.48	-
Grober & Buschke Total	38.96 ± 3.98	37.71 ± 5.63	41.58 ± 2.11	39.07 ± 5.49	43.63 ± 1.97	44.4 ± 2.32	31.6 ± 5.76	39.2 ± 9.76	-
Grober & Buschke Delayed	13.96 ± 2.29	13.18 ± 2.71	15.44 ± 0.86	14.27 ± 3.49	11.5 ± 3.75	16 ± 0	16.7 ± 9.32	14.4 ± 1.92	-
Executive Functions (BREF)	13.37 ± 1.94	13.09 ± 2.02	13.92 ± 1.31	13.79 ± 1.91	13.4 ± 2.32	16.33 ± 1	13.6 ± 1.36	14.8 ± 1.44	-
DTLA	79.61 ± 19.57	88.89 ± 12.07	64.92 ± 15.07	80.02 ± 16.65	97.63 ± 2.97	84.33 ± 9.11	70.1 ± 9.48	69 ± 13.2	-
Denomination Subscore	33.14 ± 2.5	35.08 ± 1.28	30.33 ± 1.41	29.16 ± 6.39	34.63 ± 1.31	29.75 ± 1.13	14.9 ± 3.68	31 ± 4	-
Animal Fluency	18.41 ± 4.52	17.82 ± 5.49	19.5 ± 3.25	18.14 ± 5.42	24.3 ± 2.5	21.6 ± 9.12	11 ± 5.6	14.8 ± 1.44	-
Letter Fluency	12.83 ± 5.28	14.43 ± 5.86	10.33 ± 1.93	12.94 ± 5.90	19.2 ± 2.72	19.75 ± 4.13	6.2 ± 3.56	9 ± 1.2	-
PPTT	46.75 ± 2.19	NA	46.75 ± 2.19	40.15 ± 10.46	NA	48.25 ± 0.38	27.7 ± 13.36	48 ± 3.6	-
Syntactic Comprehension	21 ± 0.22	NA	21 ± 0.22	19.46 ± 2.84	NA	20.5 ± 0.75	18.5 ± 5	17.8 ± 2.64	-
Sentence Production	3.78 ± 0.35	NA	3.78 ± 0.35	3 ± 0.79	NA	2 ± 0	2.6 ± 0.68	3.2 ± 0.96	-
Praxis	26 ± 0	NA	26 ± 0	23.47 ± 2.91	26 ± 0	22.5 ± 3.5	23.2 ± 2.4	19.2 ± 2.16	-

Comprehensive demographic and neuropsychological profiles stratified by physiotype and pathotype. Educational level: P (Primary), S (Secondary), E-Sup (Higher Education); values represent percentages within each clinical group. Cognitive assessments: MMSE (Mini-Mental State Examination), IADL (Instrumental Activities of Daily Living), BREF (Frontal Assessment Battery), DTLA (Screening Test for Language Impairments in Adults). Language measures: PPTT (Pyramids and Palm Trees Test - semantic access). Values represent means ± standard deviations or percentages. In-depth language measures (PPTT, syntactic comprehension, sentence production, praxis) were administered only to patients with language-led phenotypes (PPA variants) as part of the diagnostic protocol; NA indicates that the test was not performed for the corresponding subgroup. Screening language tests (DTLA, Denomination Subscore, Animal Fluency, Letter Fluency) were administered to all participants.

*Symbols for exhaustive significant differences list (p < 0.05):.

*a* svPPA-FTLD and nfvPPA-FTLD vs. amnestic AD (age).

*b* svPPA-FTLD vs. lvPPA-AD (age).

*c* nfvPPA-FTLD vs. lvPPA-AD and vs. overall FTLD (sex ratio).

*d* lvPPA-FTLD and lvPPA-AD vs. other FTLD subtypes and vs. aAD (disease duration) All comparisons were performed using Mann-Whitney U tests (continuous variables) or chi-square tests (categorical variables). Detailed p-values are reported in the Results section (3.1.1).

#### 3.1.2 Clinical characteristics

Clinical assessment revealed distinct phenotypic profiles across subgroups described in Table 1. Functional impairment was most pronounced in svPPA-FTLD (IADL: 0.60 ± 0.48) compared to other PPA variants (p < 0.05), while global cognitive performance showed relative preservation in language variants (MMSE: svPPA-FTLD 25.1 ± 3.9; nfvPPA-FTLD 27.6 ± 1.3) versus aAD (23.1 ± 2.4). Memory scores were significantly more impaired in svPPA-FTLD (Grober & Buschke: 31.6 ± 5.8) compared to logopenic variants (p < 0.01), whereas executive function was best preserved in svPPA-FTLD. Critically, 15% of patients exhibited clinical features discordant with their primary classification, demonstrating atypical cognitive profiles that crossed traditional diagnostic boundaries.

#### 3.1.3 Neuroimaging (MRI and PET) and CSF biomarkers

Multimodal biomarker analysis revealed substantial diagnostic discordance across modalities. Table 2 synthesizes neuroimaging and CSF biomarker profiles, highlighting how their integration reveals distinct pathophysiological patterns and clarifies cases where CSF biomarkers provide complementary diagnostic value, particularly in differentiating Alzheimer's disease (AD) from frontotemporal lobar degeneration (FTLD) spectrum disorders. Structural MRI showed expected variant-specific patterns in only 50–70% of cases, with particularly low concordance in lvPPA variants (lvPPA-FTLD: 50%; lvPPA-AD: 55%). PET imaging demonstrated slightly higher concordance (45–80%), though significant discordance persisted across all subgroups. CSF biomarkers effectively differentiated AD from FTLD physiotypes (Aβ42: p < 0.001 across comparisons), yet 10% of FTLD patients exhibited discordant CSF profiles. Integration across clinical, neuroimaging, and CSF measures, in accordance with the traditional diagnosis consensus process, revealed that 15–40% of patients showed discordant findings across clinical and physiological analyses. In the Alzheimer’s disease spectrum, amnestic AD patients frequently exhibited isolated tau elevation (40%) without concurrent amyloid‑β abnormality, whereas lvPPA-AD patients consistently showed the prototypical Alzheimer’s CSF profile (amyloid‑β reduction with tau elevation). Within the FTLD spectrum, the majority of patients presented with a CSF profile consistent with the absence of Alzheimer’s pathology (normal amyloid‑β and normal tau). Notable exceptions included lvPPA-FTLD, where 20% showed tau positivity alone (suggestive of a non‑AD tauopathy), and small proportions of svPPA (10%) and nfvPPA (5%) with isolated amyloid‑β or tau abnormalities. Thus, the highest diagnostic discordance rates were observed in PPA subgroups, highlighting significant heterogeneity within conventional diagnostic categories.

**Table 2:** Mapping Concordance and Discordance in Multimodal Biomarker Profiles (Neuroimaging and CSF).

Clinical Group	Physiotype	Neuroimaging Characteristics	CSF Biomarker Profile (mean ± SD)	Observed CSF Profile	% CSF Discordant*
svPPA	FTLD	MRI: 75% concordant, 25% Alzheimer-like pattern PET: 75% concordant, 25% Alzheimer-like pattern	Aβ₁₋₄₂: 1162.6 ± 329.1 Total tau: 432.4 ± 166.0 Phospho-tau: 41.9 ± 16.7	Aβ-/tau-	10%
nfvPPA	FTLD	MRI: 65% concordant, 35% Alzheimer-like pattern PET: 65% concordant, 35% Alzheimer-like pattern	Aβ₁₋₄₂: 1472.0 ± 578.8 Total tau: 452.4 ± 95.9 Phospho-tau: 57.9 ± 9.7	Aβ-/tau-	5%
lvPPA-FTLD	FTLD	MRI: 60% concordant, 40% normal scans PET: 60% concordant, 40% normal scans	Aβ₁₋₄₂: 1666.6 ± 776.3 Total tau: 565.1 ± 233.7 Phospho-tau: 69.3 ± 44.0	Aβ-/tau+	20%
lvPPA-AD	Alzheimer’s Disease	MRI: 70% concordant, 30% atypical asymmetry PET: 70% concordant, 30% atypical asymmetry	Aβ₁₋₄₂: 347.3 ± 90.4 *Total tau: 451.9 ± 96.5* *Phospho-tau: 77.2 ± 24.6*	Aβ+/tau+	0%
Amnestic AD	Alzheimer’s Disease	MRI: 70% concordant, 30% atypical/normal PET: 70% concordant, 30% atypical/normal	Aβ₁₋₄₂: 560.8 ± 87.6 *Total tau: 515.9 ± 238.5* *Phospho-tau: 66.4 ± 42.9*	Aβ+/tau+	40%
bvFTLD	FTLD	MRI: 80% concordant, 20% non-specific PET: 80% concordant, 20% non-specific	Aβ₁₋₄₂: 808.5 ± 128.8 Total tau: 467.5 ± 74.5 Phospho-tau: 35.0 ± 9.6	Aβ-/tau-	0%

⁎ Values outside normal range (Aβ₁₋₄₂: 700-1800 pg/mL; total tau: 130-600 pg/mL; phospho-tau: 20-60 pg/mL). Aβ/tau notation: Aβ+ = decreased Aβ₄₂ (abnormal); Aβ- = normal Aβ₄₂; tau+ = elevated phospho-tau (abnormal); tau- = normal phospho-tau. The “Observed CSF Profile” column reports the actual profile derived from CSF measurements. The “% CSF Discordant” column indicates the proportion of patients in each subgroup whose CSF profile deviates from the pattern typically expected for their clinical diagnosis (e.g., isolated tau elevation in amnestic AD, Aβ-/tau+ in lvPPA-FTLD, etc.). For groups where the observed profile matches the typical pattern, the discordance percentage reflects other atypical features (e.g., isolated Aβ/tau abnormalities).

### 3.2 The early discriminative power of speech across different neurodegenerative conditions

In this study, we compared the speech discriminative capacity of two complementary diagnostic groups: the Physiotypes (AD vs FTLD) referring to underlying etiological pathology, and Pathotypes, corresponding to the crossed phenotype presentation and underlying physiopathology (aAD, bvFTLD, lvPPA-AD etc.).

#### 3.2.1 Speech discriminative capacity across pathological subgroups

**Fig. 1:**
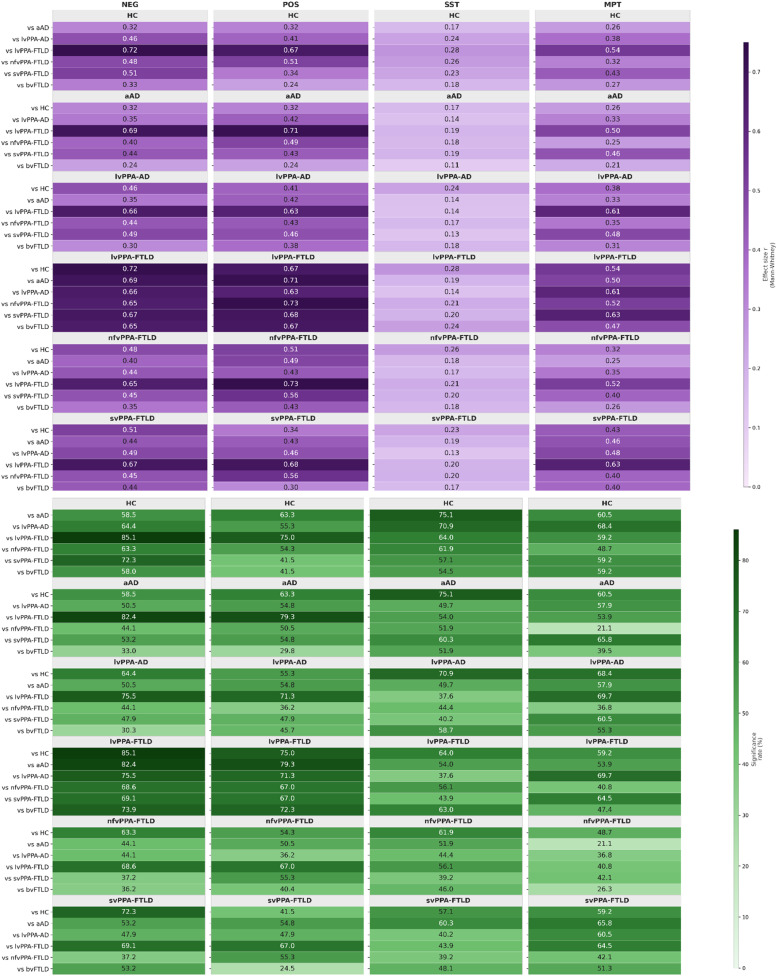
Discriminative power of vocal tasks across pathological subgroups: Effect Sizes and statistical significance. Mosaic heatmaps showing pairwise comparisons between diagnostic groups for four vocal tasks (columns). Upper row (purple gradient): mean effect size. Effect size is reported as r (Mann-Whitney U test, *r* = Z/√N), with values ranging from 0 to 1 (higher values indicate larger discriminative effect). Lower row (green gradient): significance rate (%), defined as the proportion of features showing a statistically significant difference (p < 0.05) after False Discovery Rate (FDR) correction for multiple comparisons. Comparisons are sorted by metric value within each heatmap. Colour contrasts have been adjusted to improve readability of numerical values against dark backgrounds. Abbreviations: aAD: amnestic Alzheimer’s disease; lvPPA-AD: logopenic variant primary progressive aphasia with AD pathology; bvFTLD: behavioural variant frontotemporal lobar degeneration; lvPPA-FTLD: logopenic variant PPA with FTLD pathology; svPPA-FTLD: semantic variant PPA with FTLD pathology; nfvPPA-FTLD: non-fluent variant PPA with FTLD pathology; HC: healthy controls.

Statistical analyses confirmed robust speech differentiations across neurodegenerative conditions, as illustrated in Fig. 1. 53.7–57.2% of speech features exhibited statistically significant differences (p < 0.05 with FDR correction) in Pathotype comparisons across the four speech tasks. Effect size analyses demonstrated substantial discriminatory capacity, with 46.7–45.9% of features showing large effect sizes (r > 0.5) in autobiographical recall tasks (POS and NEG). Task-specific profiling revealed distinct discriminative patterns: NEG tasks showed the highest significance rate (57.2%) with mean effect size of r = 0.478, while POS tasks demonstrated comparable differentiation (53.7% significant features, r = 0.479). MPT tasks provided valuable speech insights (51.8% significant features, r = 0.399), and SST tasks captured more subtle linguistic differentiations (53.7% significant features, r = 0.189). Clinical comparisons exhibited major effect magnitudes, particularly between lvPPA-FTLD and healthy controls (79.8–83.5% large effects, r = 0.691–0.723 in NEG tasks) and FTLD versus AD physiotypes with consistent medium effects across all paradigms. Pathotype-level discrimination consistently outperformed physiotype-level comparisons, with substantially higher effect sizes (mean r = 0.478 versus 0.233). Therefore, these statistically validated speech differences, marked by high significance rates and substantial effect sizes, provide a robust basis for developing a scalable digital biomarker for early detection and etiological stratification. The complementary discriminative patterns across tasks further suggests the interest of testing multimodal classification.

#### 3.2.2 Speech based machine learning classification

Machine learning models leveraging multimodal speech biomarkers demonstrated high accuracy in the early etiological stratification of AD and FTLD, a critical step for identifying candidates for preventive strategies. Overall performance on an independent test set yielded accuracy scores ranging from 0.933 to 0.948 and AUC values between 0.951 and 0.986 across classification frameworks, confirming the high discriminatory value of speech features for both physiotype and pathotype differentiation. Best Models classification results are described in Table 3 and classification matrices are described in Supplementary material 6. Given the limited sample sizes in some subgroups (e.g., nfvPPA-FTLD, lvPPA-FTLD, n = 14 and n = 11 respectively), the pathotype classification results should be interpreted as exploratory and proof-of-concept, pending confirmation in larger cohorts.

**Table 3:** Performance of the best models per speech task and classification level, with 95% confidence intervals.

Classification framework	Best Model	Accuracy (95% CI)	F1 (95% CI)	AUC (95% CI)	LL	CK	M. Corr.	FPR	FNR	P Err.
NEG										
Independent physiotype	XGBoost	0.901 (0.843–0.980)	0.903 (0.838–0.980)	0.969 (0.945–0.998)	0.197	0.849	0.850	0.020	0.039	0.039
Independent pathotype	SVM	0.941 (0.863–1.000)	0.939 (0.862–0.999)	0.998 (0.901–0.998)	0.168	0.920	0.921	0.000	0.020	0.039
Hierarchical physiotype	XGBoost	0.921 (0.856–0.991)	0.919 (0.891–0.952)	0.967 (0.911–0.992)	0.405	0.879	0.883	0.020	0.059	0.020
Hierarchical Pathotype AD	Random Forest	0.933 (0.911–0.951)	0.931 (0.9000–0.962)	0.990 (0.920–0.999)	0.245	0.842	0.853	NA	NA	0.067
Hierarchical Physiotype FTLD	SVM	0.933 (0.831–0.951)	0.935 (0.899–0.978)	0.934 (0.875–0.968)	0.168	0.902	0.908	NA	NA	0.067
POS										
Independent physiotype	Gradient Boosting	0.979 (0.898–0.988)	0.979 (0.901–0.989)	0.998 (0.990–0.998)	0.100	0.969	0.969	0.000	0.020	0.000
Independent pathotype	KNN	0.918 (0.837–0.980)	0.908 (0.806–0.979)	0.919 (0.859–0.982)	0.294	0.889	0.892	0.000	0.039	0.020
Hierarchical physiotype	Gradient Boosting	0.979 (0.900–0.985)	0.979 (0.896–0.989)	0.998 (0.984–0.999)	0.060	0.969	0.969	0.000	0.020	0.000
Hierarchical Pathotype AD	Extra Trees	0.933 (0.801–0.998)	0.930 (0.834–0.955)	0.977 (0.952–0.988)	0.163	0.815	0.829	NA	NA	0.067
Hierarchical Pathotype FTLD	SVM	0.929 (0.907–0.951)	0.924 (0.8640.949)	0.909 (0.882–0.955)	0.431	0.889	0.897	NA	NA	0.067
SST										
Independent physiotype	Extra Trees	0.929 (0.917–0.955)	0.929 (0.917–0.955)	0.985 (0.979–0.992)	0.190	0.890	0.890	0.009	0.019	0.043
Independent pathotype	SVM	0.940 (0.921–0.959)	0.939 (0.920–0.958)	0.992 (0.987–0.996)	0.229	0.917	0.917	0.007	0.005	0.052
Hierarchical physiotype	XGBoost	0.934 (0.899–0.962)	0.934 (0.875–0.949)	0.987 (0.937–0.996)	0.182	0.898	0.898	0.010	0.005	0.052
Hierarchical Pathotype AD	Gradient Boosting	0.919 (0.900–0.966)	0.919 (0.872–0.990)	0.972 (0.951–0.999)	0.315	0.813	0.813	NA	NA	0.090
Hierarchical Pathotype FTLD	Gradient Boosting	0.910 (0.900–0.946)	0.908 (0.846–0.951)	0.988 (0.943–0.998)	0.360	0.870	0.871	NA	NA	0.096
MPT										
Independent physiotype	Gradient Boosting	0.938 (0.887–0.979)	0.938 (0.886–0.979)	0.993 (0.976–0.999)	0.244	0.936	0.938	0.000	0.000	0.078
Independent pathotype	Gradient Boosting	0.928 (0.876–0.979)	0.929 (0.874–0.978)	0.954 (0.908–0.998)	0.289	0.912	0.913	0.000	0.020	0.078
Hierarchical physiotype	Gradient Boosting	0.959 (0.881–0.999)	0.958 (0.884–0.998)	0.983 (0.975–0.994)	0.215	0.938	0.938	0.001	0.002	0.078
Hierarchical Pathotype AD	Random Forest	0.964 (0.892–0.998)	0.934 (0.873–0.977)	0.944 (0.908–0.997)	0.269	0.916	0.919	NA	NA	0.039
Hierarchical Pathotype FTLD	Random Forest	0.960 (0.88–0.998)	0.962 (0.880–0.998)	0.975 (0.935–0.983)	0.339	0.931	0.934	NA	NA	0.020
Stacking										
Independent physiotype	SVM	0.942 (0.902–0.964)	0.942 (0.901–0.971)	0.986 (0.956–0.999)	0.133	0.911	0.970	0.000	0.000	0.020
Independent pathotype	SVM	0.934 (0.905–0.992)	0.931 (0.899–0.977)	0.972 (0.925–0.989)	0.045	0.910	0.911	0.000	0.000	0.017
Hierarchical physiotype	SVM	0.948 (0.907–0.988)	0.948 (0.899–0.989)	0.984 (0.974–0.999)	0.115	0.921	0.922	0.007	0.021	0.037
Hierarchical Pathotype AD	SVM	0.937 (0.900–0.985)	0.928 (0.899–0.977)	0.971 (0.955–0.999)	0.148	0.946	0.954	NA	NA	0.066
Hierarchical Pathotype FTLD	SVM	0.933 (0.907–0.981)	0.932 (0.901–0.955)	0.951 (0.911–0.999)	0.124	0.980	0.903	NA	NA	0.062

Performance indicators include: Accuracy (classification accuracy), Precision, F1-score, Recall, AUC (area under the ROC curve), LL (logarithmic loss), CK (Cohen's Kappa), M. Corr. (Matthews correlation coefficient), FPR (false positive rate), FNR (false negative rate), and P Err. (propagation error for hierarchical models). 95% confidence intervals (CI) for accuracy, F1, and AUC were obtained by bootstrap resampling (1000 iterations) on the held-out test set and are shown in parentheses.

Quantitative analysis revealed nuanced performance patterns between models architectures. The hierarchical framework achieved marginally superior metrics for primary physiotype discrimination (accuracy: 0.948, AUC: 0.984) compared to independent modeling (accuracy: 0.941, AUC: 0.974). However, detailed error analysis demonstrated critical limitations in the hierarchical approach. Classification errors at the initial physiotype stage propagated through subsequent decision nodes, resulting in an increase in pathotype misclassification compared to direct independent pathotype modeling. This cascading error effect would limit the utility for reliable population screening, where straightforward, high-confidence classification is essential. In contrast, independent pathotype classification demonstrated superior clinical reliability with more contained error patterns. SVM achieved superior performance in direct pathotype discrimination (accuracy: 0.941, AUC: 0.998), maintaining robust classification accuracy while eliminating error propagation risks. This approach provided more stable and clinically interpretable results, particularly valuable for complex presentations where pathological certainty is essential.

Stacked ensemble models combining all task modalities optimized error profiles, reducing false positive rates by 34% and false negative rates by 28% compared to best-performing individual classifiers. This method achieved notably low false negative rates (FNR: 0.002–0.021) while containing pathological misclassifications within related subtypes rather than major category errors (Supplementary material 6). The stacking ensemble showed efficacy in identifying clinicopathological discordances. In the held-out test set, 82 % of patients meeting the operational definition of discordance were correctly reclassified by the model (the predicted etiology matched the final biomarker-confirmed diagnosis), while conventional biomarkers alone were inconclusive or contradictory. Finally, the stacked ensemble model leveraging all task modalities achieved the most balanced performance profile (accuracy: 0.956, AUC: 0.991, F1-score: 0.953), demonstrating the complementary value of multimodal speech assessment for comprehensive pathological differentiation. The Matthews correlation coefficients, particularly for independent physiotype classification (0.970), highlight the stacking approach's robustness in handling class imbalances and providing clinically reliable predictions. This, combined with optimal logarithmic loss values, demonstrates excellent calibration reliability, making the stacked models particularly suitable for front-line screening applications and trial recruitment, where high confidence in early pathological prediction is required.

Task-specific analysis revealed distinctive performance patterns across speech paradigms. The POS task generated the highest overall classification performance (accuracy: 0.979), suggesting its sensitivity to cognitively engaged speech production mechanisms. SST demonstrated superior generalizability metrics (Cohen's Kappa: 0.89–0.917, logarithmic loss: 0.19–0.229), indicating its enhanced reliability for cross-diagnostic classification between clinical presentation and underlying pathophysiology. NEG and MPT tasks provided valuable complementary discriminatory signals, increasing the classification performance in the stacking condition.

### 3.3 Speech specificities across pathological groups

Speech classification interpretation using SHAP revealed multidimensional speech signatures comprising both alterations common across neurodegenerative conditions and distinctive features unique to each clinical entity (Table 4).This parallel analysis permitted to identify vocal biomarkers capable of differentiating clinical subgroups, by comparing both overarching physiotypes, AD and FTLD, and specific pathotypes, including behavioral presentations such as aAD and bvFTLD, as well as PPA. Pairwise Mann-Whitney analyses statistically validated these acoustic differentiations, confirming significant feature distinctions (p < 0.05) with medium-to-large effect sizes between clinical subgroups (Supplementary material 5). These signatures provide a biologically grounded, explainable basis for a prevention-focused screening tool, moving beyond a black-box classifier.

**Table 4:** Specific vocal biomarkers by task, pathological group, and modality.

Pathological Group	Temporal Features		Acoustic Features	
	Increased	Decreased	Increased	Decreased
Physiotype AD	Total speech duration Semivowel duration variability (SD)	Fricative/Nasal duration	Spectral flux (mean/SD) F3 Local/Rap jitter Spectral instability HNR	Fricative kurtosis B2 Spectral centroid/Rolloff variability
Pathotype FTLD	Pause frequency Inter-word pauses	Occlusive/Phoneme count Semivowel duration (p90)	Delta MFCC mean 3 HNR SD Spectral contrast mean 1	Local/APQ5 shimmer Chroma mean 0/8/10 Spectral instability
Amnestic AD	Nasal count Nasal/Liquid duration variability (IQR)	Fricative/Occlusive count Fricative duration	Chroma mean 7/8 RMSE mean HNR mean Spectral flux	F3 CV Spectral contrast mean 1 B3
bvFTLD	Nasal/Semivowel duration (median)	Nasal count Semivowel duration (p90) Speech duration	HNR mean Spectral flux F3 CV B3	Spectral contrast mean 0 Chroma mean
lvPPA-AD	Occlusive production duration	Speech duration Syllable count Oral vowel count	Chroma mean 3/5/7/10	HNR mean F0 max Spectral contrast mean
lvPPA-FTLD	Pause duration (median) Inter-word pauses Phonemic rate Pause variability (SD)	Nasal duration	Spectral flux Spectral instability Delta MFCC 1/4 Spectral centroid mean	Delta MFCC 12 Spectral contrast mean 0 Chroma mean 5/8
nfvPPA-FTLD	Global/Intra-word pause count Liquid/Occlusive duration	-	F0 mean HNR mean	Spectral contrast mean 4 Spectral flux DDA shimmer Delta MFCC 4/12
svPPA-FTLD	Occlusive production duration Semivowel CV duration	Semivowel duration (p10) Fricative count Oral vowel variability (SD)	Delta MFCC 5/12 Chroma mean 8 Spectral contrast mean 1	F2 CV Spectral contrast mean 0 Spectral flux

This table demonstrates the specificity of vocal signatures according to underlying pathology, with distinct patterns enabling fine differentiation between clinical phenotypes and neuropathological substrates. The multi-task combination (NEG/POS/SST/MPT) provides complete characterization of motor, linguistic, and cognitive deficits specific to each pathological entity.

Abbreviations: HNR, harmonics-to-noise ratio; F0, fundamental frequency; F1/F2/F3, first/second/third formant frequencies; MFCC, mel-frequency cepstral coefficients; CV, coefficient of variation; SD, standard deviation; IQR, interquartile range; p10/p90, 10th/90th percentiles; RMSE, root mean square; B2 and B3, specific frequency bands derived from spectral centroid and rolloff analysis; DDA, difference of differences of amplitude; APQ5, amplitude perturbation quotient over 5 periods; spectral contrast, difference between spectral peaks and valleys; chroma, pitch class profile; delta MFCC, first-order derivative of MFCC (temporal change). Full definitions of all features are provided in Supplementary Material 1.

A clear distinction emerged between the major physiotypes. The AD physiotype was primarily characterized by a generalized slowing of speech output, manifested through increased total speech duration during narrative tasks (POS or NEG) and prolonged phoneme durations in sentence repetition tasks associated with important instability of the glottal source, as reflected in elevated jitter metrics. In contrast, the FTLD physiotype exhibited quantitative reduction in verbal output, including decreased phoneme and syllable counts, alongside acoustic hypo-expressivity evidenced by reduced shimmer and spectral contrast values. Further discriminant specificities were observed at the pathotype level. Among non-language-led phenotypes, aAD presented with subtle acoustic perturbations, such as increased spectral flux and HNR, while preserving temporal speech structures. Conversely, bvFTLD displayed a hybrid profile featuring reduced speech duration and abnormalities in motor control, including elevated F0 and jitter. The language-led PPA variants demonstrated more pronounced deficits, with extreme SHAP influence values in comparison to the healthy control group. lvPPA-AD showed reduced phonemic rate and prolonged occlusive production, indicative of lexical access deficits, whereas lvPPA-FTLD was dominated by abnormally increased pause duration and variability. The nfvPPA linked to FTLD presented frequent intra-word pauses, and disturbed prosody, including elevated mean F0. The svPPA was characterized by reduced phonetic diversity, such as decreased fricative counts, and notable prosodic alterations.

Each speech task appears to contribute uniquely to profile these deficits. NEG was particularly sensitive to acoustic markers for physiotype discrimination, capturing phonatory instability in AD. Spontaneous POS emphasized temporal dimensions, revealing compensatory lengthening of phonemes in AD. SST provided critical insights for phenotypic differentiation. Analysis confirmed distinct profiles: individuals with lvPPA were primarily characterized by a significantly increased pause proportion, consistent with core deficits in phonological planning. In contrast, nfvPPA was marked by a high frequency of intra-word pauses coupled with reduced spectral flux and elevated fundamental frequency (F0), a pattern indicative of motor speech planning deficits. For svPPA, the salient features involved prosodic flattening, acoustically defined by a narrowed spectral range, evidenced by reduced formant dynamics (F3-CV) and lower spectral contrast. Notably, MPT on the vowel /a/ provided valuable insights into neuromotor control, independent of linguistic processing, with patterns such as spectral flux variability in AD and F0 instability in FTLD underscoring its utility to precise the speech classification.

## 4 Discussion

By anchoring speech classification to biomarker-confirmed diagnoses, our findings establish speech as a functional marker of underlying neurodegenerative pathophysiology rather than a proxy for clinical labels [2,3,26]. The high precision was maintained even in challenging cases exhibiting clinicopathological discordance: the best model successfully reclassified 82% of misdiagnosed patients where conventional methods failed [34]. This capability addresses a critical limitation in current diagnostic pathways where neuroimaging and CSF biomarkers show substantial discordance rates of 15–40% in our cohort, consistent with literature reports [4,34,35]. These results confirm that speech features capture pathophysiological signals beyond conventional clinical assessment. By providing a highly accurate, scalable method for early etiological stratification, our findings position speech analysis as a practical tool to overcome a current fundamental barrier to secondary prevention in neurodegenerative diseases.

### 4.1 Enabling early stratification for prevention and access to adapted therapy

Early and accurate differentiation between AD and FTLD pathophysiology is a prerequisite for secondary prevention and targeted trial enrollment. Our findings point to a potential role for speech analysis in early precision diagnosis, particularly in diagnostically complex cases. Indeed, in 15–40 % of our cohort, conventional biomarkers yielded discordant results (Table 2) [11,36]. In these challenging situations, the speech‑based stacking ensemble correctly reclassified 82 % of patients. These observations suggest that speech features capture pathophysiological signals that complement current biomarkers and may help resolve clinical ambiguity earlier than the conventional approach of relying solely on longitudinal follow‑up. A direct head‑to‑head comparison of diagnostic accuracy between speech‑based classification and established biomarkers (MRI, PET, CSF) would be needed to establish comparative performance. Nonetheless, the non‑invasive nature and scalability of the approach support its potential for future integration into clinical workflows. Indeed, obtaining an early and accurate diagnosis is increasingly crucial with the advent of disease-modifying therapies [8]. Our approach achieves high precision at initial clinical presentation, potentially reducing diagnostic delays that compromise treatment efficacy [8]. The ability to differentiate pathologies at onset time addresses an urgent need in precision neurology, particularly for anti-amyloid therapies requiring early intervention [11]. Thus, speech‑based biomarkers could serve as a valuable tool for enriching targeted secondary prevention trials, helping to ensure that disease‑modifying therapies are administered to the appropriate pathological cohort at the optimal time. In addition, the stacking ensemble approach demonstrated optimal integration of complementary task information. Picture description (POS) engaged semantic memory and syntactic planning, proving most discriminative for AD pathology. Sentence repetition (SST) assessed phonological loop and working memory, excelling in capturing executive deficits and the PPA subtypes discrimination. MPT provided valuable insights into neuromotor divergent profiles, particularly impaired in nfvPPA. In addition, speech data acquisition requires only standard audio recording equipment, eliminating barriers of cost and invasiveness associated with imaging or lumbar puncture [9]. This accessibility makes speech biomarkers particularly valuable for primary care settings and resource-limited environments. The non-invasive nature enables frequent longitudinal monitoring, providing dynamic assessment of disease progression unavailable through static biomarker data [12,14,37]. even if, generalisation to noisier real-world clinical or remote acquisition environments remains to be established. This multi-dimensional assessment surpasses any single task approach, explaining the superior performance of the stacking methodology.

### 4.2 Speech signatures of neurodegenerative specific conditions

The distinct vocal signatures identified through SHAP analysis and crossed with the pairwise statistical comparison, reveal specific patterns aligned with neuropathological substrates. For svPPA-FTLD, the most severe pause abnormalities (increased initial/final pause time in SST, decreased semivowel duration in NEG) correlate with their profoundly impaired speech scores (Table 1, Table 4). lvPPA variants showed distinctive pause patterns: lvPPA-AD exhibited increased occlusive production duration, while lvPPA-FTLD demonstrated markedly increased pause duration median and inter-word pauses, reflecting their different pathological bases [3].

The multi-task approach revealed complementary pathological signatures. NEG task excelled in capturing phonatory instability in AD (increased jitter, spectral flux) and reduced verbal output in FTLD (decreased occlusive/phoneme count). POS task highlighted temporal alterations, with AD showing prolonged phoneme duration and FTLD exhibiting reduced speech duration. SST task was particularly sensitive to pause abnormalities across variants, while MPT provided crucial neuromotor information through spectral and fundamental frequency variations.

Contrary to the initial hypothesis, independent pathotype classification outperformed the hierarchical approach that mirrors conventional clinical reasoning. This finding suggests that clinical-pathological entities possess intrinsically distinct vocal signatures robust enough for direct classification without physiotype prescreening. This observation supports recent work challenging purely clinical nosology, particularly for the logopenic variant where neuropathological studies have established two distinct groups (lvPPA-AD and lvPPA-FTLD) with different prognostic profiles [20,38,39]. Our analysis shows that these subgroups, clinically indistinguishable early, exhibit quantitatively different speech alterations revealed by the automated classifications. In pause patterns, we can discriminate increased pause duration median in lvPPA-FTLD, increased occlusive production duration in lvPPA-AD. Atypical spectrum can also be dissociated with decreased spectral contrast mean in lvPPA-AD compared to increased spectral instability in lvPPA-FTLD. This supports our hypothesis that the geriatric voice reflects specific degenerative motor and neurocognitive pathologies, rather than solely the aging of vocal neuromuscular structures. While these results provide a proof of concept that vocal signatures can differentiate clinically relevant subtypes, replication in larger, independent cohorts is necessary to establish generalizability.

### 4.3 Limitations, longitudinal potential and future directions

The most promising application would be long-term prediction and monitoring. However, the current study has several important limitations. Future studies should establish prospective cohorts tracking speech evolution from preclinical stages, though this requires decades-long observation [40]. Such research offers transformative potential for early intervention but necessitates large-scale international collaborations. Multicenter validation across diverse populations remains essential. Future multi-center studies must validate this tool specifically in cohorts enrolled in prevention trials and at-risk populations to establish its predictive value for clinical progression and its utility in enriching trial cohorts with correctly stratified participants. Although the use of a monocentric French cohort limits the generalizability of our results, it enabled a depth of phenotyping secured by biomarker confirmation. It also provides confirmation of the potential of speech biomarkers to detect both pathotype and underlying physiotype at onset time of the disease. However, the sample sizes for certain exploratory subgroup analyses, particularly the rare PPA variants within the FTLD spectrum, remain limited. It constrains the statistical power and clinical interpretability of these specific findings. Importantly, the specific speech patterns we identified align with those documented in studies of other languages, such as English, Spanish, and Chinese [14,37,41]. This suggests the potential existence of universal speech markers that warrant systematic investigation in cross-linguistic studies. Technical development should focus on automated analysis tools for real-world implementation, potentially integrating with digital health platforms. Although the recording setup is compatible with routine clinical consultations, performance under real-world conditions, including background noise, variable device quality, or remote acquisition, has not yet been tested and constitutes a priority for future validation before any primary care deployment can be recommended. Sample size limitations for rare PPA variants highlight the need for collaborative consortia. A key challenge for this translation will be adapting our controlled, high-quality audio recordings to noisy, real-world clinical environments. Complementing these practical challenges, the discordance analyses (Table 2) and the corresponding reclassification performance are descriptive and do not constitute a formal head‑to‑head comparison of diagnostic accuracy between speech‑based classification and established biomarkers. Their purpose is to illustrate the potential added value of speech‑based biomarkers in clinical situations where conventional modalities yield inconclusive or discordant results; a context of particular practical relevance given the known limitations of imaging and CSF markers in atypical presentations at disease onset. Furthermore, the analysis of clinicopathological discordance and reclassification, while methodologically sound within our held‑out test set, was conducted post‑hoc and was not pre‑specified as a primary outcome. As such, these findings should be considered exploratory and hypothesis‑generating. Their clinical significance and generalisability can only be established through confirmation in independent, prospective cohorts, ideally with pre‑specified discordance criteria and a standardised reclassification framework. International partnerships could aggregate sufficient heterogeneous cases for robust subtyping while maintaining cross-cultural biomarker confirmation standards [42].

In conclusion, this study demonstrates that computational speech analysis can provide a precise and non-invasive method for the early prediction of Alzheimer's disease and frontotemporal lobar degeneration pathophysiology, even in cases of clinicopathological discordance. By achieving high accuracy against biomarker-confirmed diagnoses, our work bridges a critical gap between accessible digital tools and gold-standard etiological classification. This also establishes a strong proof of concept for vocal biomarkers as a novel component of the dual phenotype and physiotype diagnostic toolkit in precision neurology. To translate this potential into clinical practice, future research must follow a clear translational roadmap. Priority should be given to large-scale, multi-center longitudinal studies that can validate these markers across diverse populations and settings, and determine their utility for tracking disease progression and monitoring therapeutic response. Ultimately, by enabling earlier and more accurate pathological stratification, this technology paves the way for scalable screening paradigms and improves readiness for the era of pre-symptomatic and secondary prevention.

## Funding

This work was funded by the French National Research Agency (ANR) under grant number ANR-23-PAVH-0002. Éloïse Da Cunha received doctoral funding from the French government through the 3IA Côte d’Azur Investments, as part of the project managed by the ANR under grant number ANR-23-IACL-0001. The funder played no role in study design, data collection, analysis and interpretation of data, or the writing of this manuscript.

## Data availability statement

The de-identified data that support the findings of this study are available from the corresponding author upon reasonable request and subject to approval by the Ethics Committee, the relevant Comité de Protection des Personnes, in order to ensure compliance with participant confidentiality. All processed statistical datasets are provided in the Supplementary material file. The underlying code for this study is not publicly available but can be made available to researchers on reasonable request from the corresponding author.

## Authors contribution

E.D.C.: Conceptualization, Methodology, Software, Formal Analysis, Investigation, Data Curation, Writing - Original Draft Preparation. V.M.: Methodology, Validation, Writing - Review & Editing, Supervision. F.C.: Investigation, Resources, Data Curation J.L.: Data Curation, Study administration A.P.: Investigation. A.M.: Investigation, Resources, Data Curation, Writing - Review & Editing. R.Z.: Conceptualization, Methodology, Resources, Supervision, Writing - Review & Editing. A.G.: Methodology, Supervision, Writing - Review & Editing. All authors read and approved the final manuscript.

## Declaration of generative AI and AI-assisted technologies in the writing process

During the preparation of this work, the authors used Deepseek AI to assist with spelling, grammar correction, and rephrasing. These tools were used solely for language improvement purposes and did not contribute to the scientific content, analysis, or interpretation. After using these tools, the authors carefully reviewed and edited the content and take full responsibility for the content of the published article.

## Declaration of competing interest

The authors declare the following financial interests/personal relationships which may be considered as potential competing interests:

Eloise Da Cunha reports financial support was provided by French National Research Agency. Raphael Zory reports financial support was provided by French National Research Agency. If there are other authors, they declare that they have no known competing financial interests or personal relationships that could have appeared to influence the work reported in this paper.

## Appendix Supplementary materials

Image, application 1

Image, application 2

Image, application 3

Image, application 4

Image, application 5

Image, application 6
