# Supplementary material for: Speech-based digital biomarkers for early etiological stratification of Alzheimer’s disease and frontotemporal degeneration: a biomarker-confirmed prospective study
Source: J Prev Alzheimers Dis. 2026 Apr 17;13(6):100573. doi: 10.1016/j.tjpad.2026.100573 (PMC13098405; doi:10.1016/j.tjpad.2026.100573)
Supplement: Supplementary file 1 [file mmc1.docx]

**Supplementary Table 1 : Speech Features definition**

| Feature name | Definition |
| --- | --- |
| Voice perturbation (jitter, shimmer) | |
| jitter_local | Cycle-to-cycle variation in fundamental frequency (F0), expressed as a percentage. |
| jitter_rap | Relative average perturbation: mean absolute difference between a period and the average of its two neighbors, divided by the average period. |
| jitter_ppq5 | Pitch perturbation quotient over 5 consecutive periods. |
| shimmer_local | Cycle-to-cycle variation in amplitude, expressed as a percentage. |
| shimmer_apq3 | Amplitude perturbation quotient across 3 periods. |
| shimmer_apq5 | Amplitude perturbation quotient across 5 periods. |
| shimmer_dda | Difference of differences of amplitudes: average absolute difference between consecutive differences in peak amplitudes. |
| Spectral descriptors |  |
| spectral_flux | Frame-to-frame change in the power spectrum (spectral dynamics). |
| spectral_instability | Variability of the spectral envelope across frames. |
| f1_cv, f2_cv, f3_cv | Coefficient of variation of the first, second, and third formant frequencies. |
| hnr_mean | Mean harmonic-to-noise ratio (periodicity vs. noise in the signal). |
| hnr_std | Standard deviation of harmonic-to-noise ratio. |
| MFCC-based features |  |
| delta_mfcc_mean_1–12 | Mean values of temporal derivatives (Δ) of Mel-frequency cepstral coefficients (MFCCs 1–12), reflecting dynamic spectral information. |
| Time-domain descriptors |  |
| zcr_mean | Mean zero-crossing rate (average number of sign changes of the waveform per frame). |
| Chroma features |  |
| chroma_mean_0–11 | Mean energy distribution across the 12 chroma bands (pitch classes), capturing harmonic content. |
| Spectral contrast |  |
| contrast_mean_0–6 | Average difference between peaks and valleys in the spectrum across seven sub-bands. |
| Spectral shape and energy |  |
| spectral_centroid_mean/std | Mean and variability of the spectral centroid (perceptual “brightness” of the sound). |
| spectral_centroid_slope | Temporal slope of the spectral centroid. |
| rmse_mean | Mean root-mean-square energy (signal power). |
| rolloff_mean/std | Mean and variability of spectral roll-off (frequency below which 85% of the spectrum energy is contained). |
| spectral_flux_mean/std | Mean and variability of spectral flux. |
| Fundamental frequency (F0) |  |
| f0_mean, f0_std, f0_min, f0_max | Descriptive statistics of fundamental frequency (mean, variability, min, max). |
| f0_slope | Temporal slope of F0 across the utterance. |
| Intensity (loudness) |  |
| intensity_mean, intensity_std | Mean and variability of intensity (dB). |
| intensity_dynamic_range | Range of intensity values. |
| intensity_slope | Temporal slope of intensity. |
| Formants and bandwidths |  |
| f1, f2, f3 | Mean frequency of the first three formants. |
| b1, b2, b3 | Mean bandwidth of the first three formants. |
| Duplicated acoustic features (suffix “,1”) | Same as above, but extracted from secondary or repeated segments (e.g., second trial or sentence). |
| Temporal speech measures |  |
| total_duration | Total recording duration (s). |
| Latency time | Time for the patient to initiate the speech (s). |
| speech_duration | Duration of voiced segments (s). |
| pause_total_duration | Total duration of pauses (s). |
| phoneme_count, syllable_count | Number of phonemes or syllables produced. |
| num_speech_phonemes | Number of phonemes classified as speech sounds. |
| num_pauses | Total number of pauses. |
| num_inter_word_pauses | Number of pauses between words. |
| num_intra_word_pauses | Number of pauses within words. |
| num_initial_pauses, num_final_pauses | Pauses occurring at the beginning or end of the recording. |
| num_inter_pauses | Pauses between utterances or phrases. |
| mean/median/std/min/max phoneme_duration | Descriptive statistics of phoneme duration distribution. |
| cv/skew/kurtosis phoneme_duration | Coefficient of variation, skewness, and kurtosis of phoneme duration. |
| pause_mean/median/std/min/max_duration | Descriptive statistics of pause duration distribution. |
| pause_cv | Coefficient of variation of pause durations. |
| inter_word_pause_mean–max | Descriptive statistics of inter-word pause durations. |
| long_pause_count | Number of pauses exceeding a pre-defined threshold (e.g., >250 ms). |
| pause_frequency | Number of pauses per unit time. |
| pause_proportion | Ratio of pause duration to total duration. |
| pause_ratio | Ratio of pause count to speech units (e.g., syllables). |
| speech_rate_phonemes_per_sec | Speech rate expressed as phonemes per second. |
| rate_speech_phonemes | Alternative measure of phoneme production rate. |
| articulation_rate | Number of speech units produced per second, excluding pauses. |
| syllable_rate_per_sec | Number of syllables produced per second. |
