# Supplementary material for: Speech-based digital biomarkers for early etiological stratification of Alzheimer’s disease and frontotemporal degeneration: a biomarker-confirmed prospective study
Source: J Prev Alzheimers Dis. 2026 Apr 17;13(6):100573. doi: 10.1016/j.tjpad.2026.100573 (PMC13098405; doi:10.1016/j.tjpad.2026.100573)
Supplement: Supplementary file 2 [file mmc2.docx]

**Supplementary Figure 2 : Methodological synthesis of speech analysis for physiotype and pathotype prediction**

**Figure 1. Global study design and participants’ stratification**

**
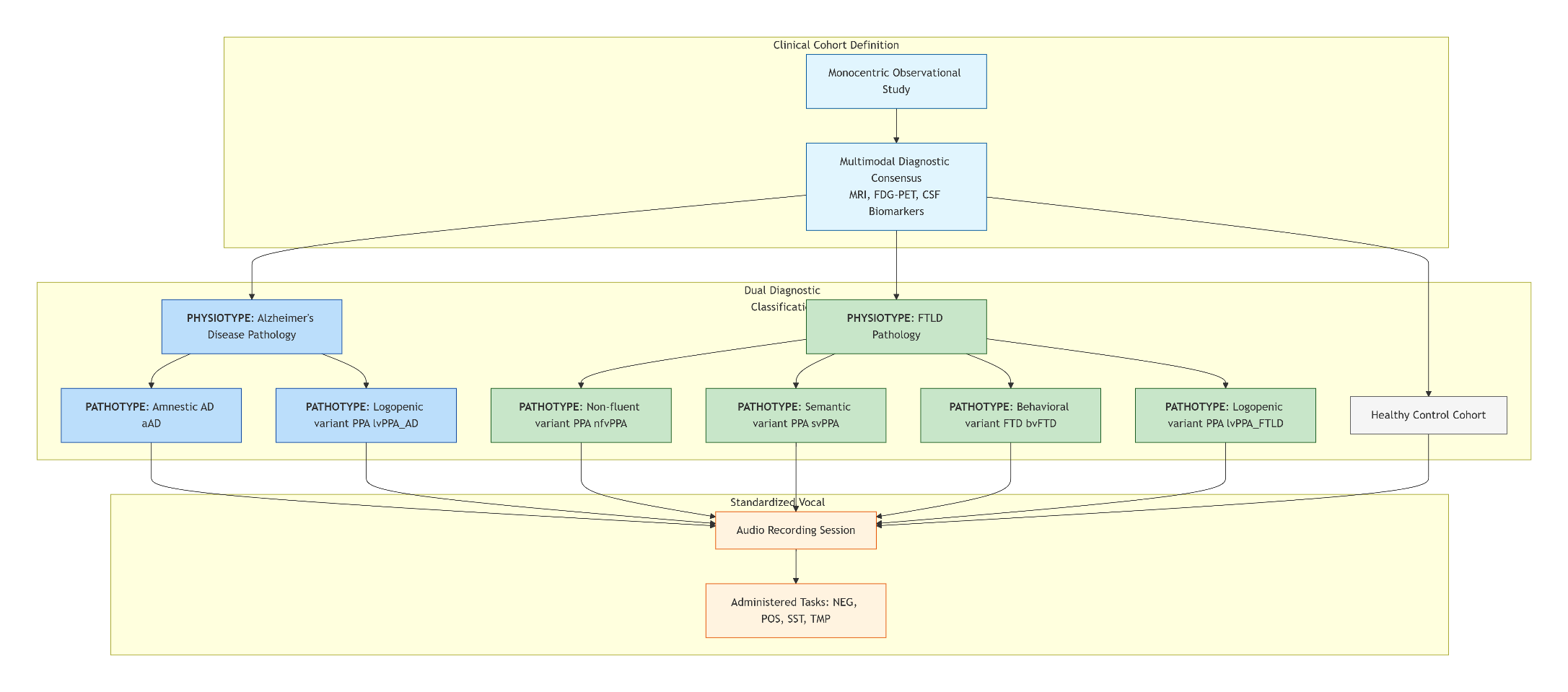
**

**Figure 2. Data Preprocessing and Feature Engineering Pipeline**

**
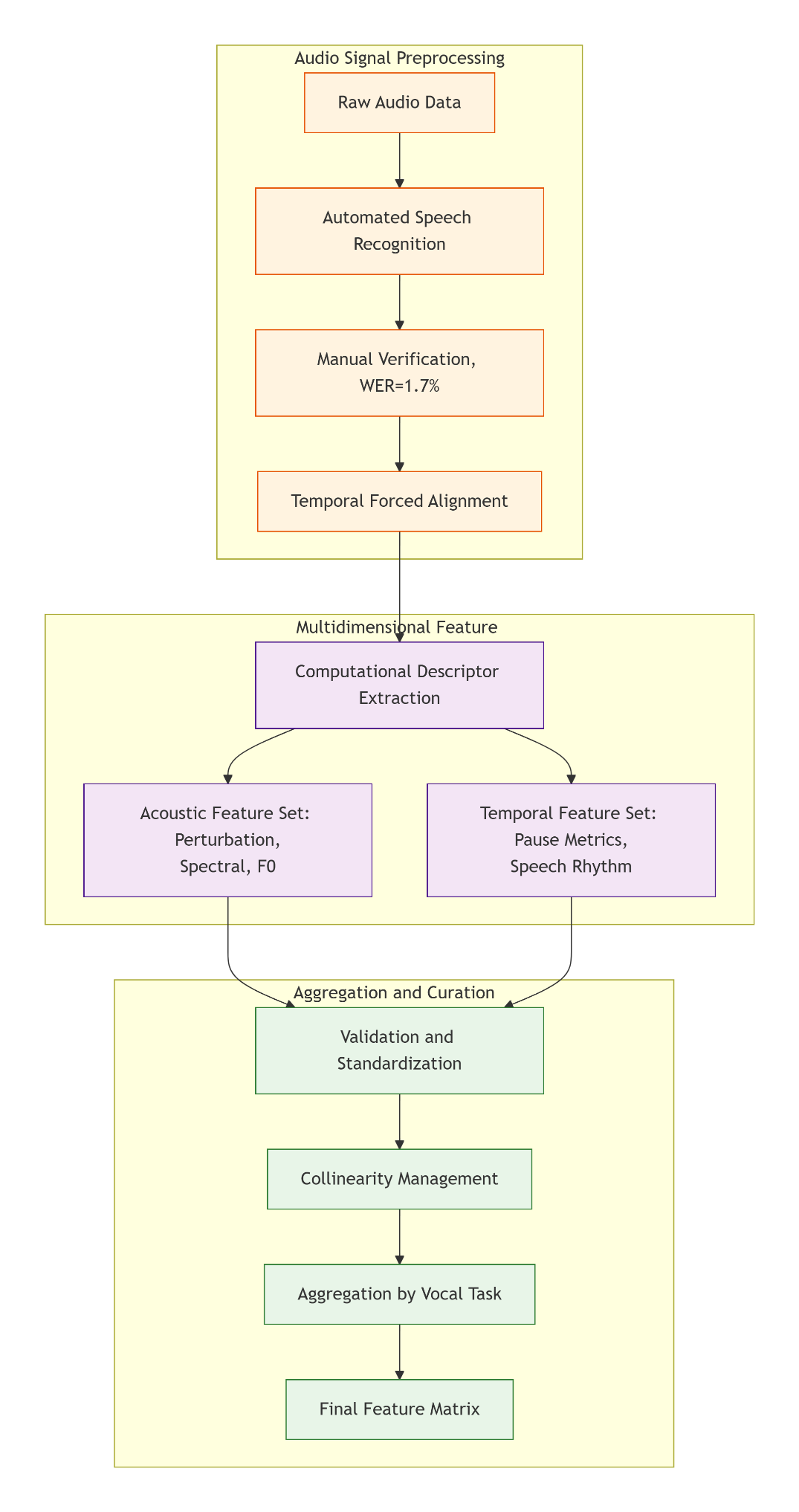
**

**Figure 3. Modeling Framework and Algorithm Selection**

**
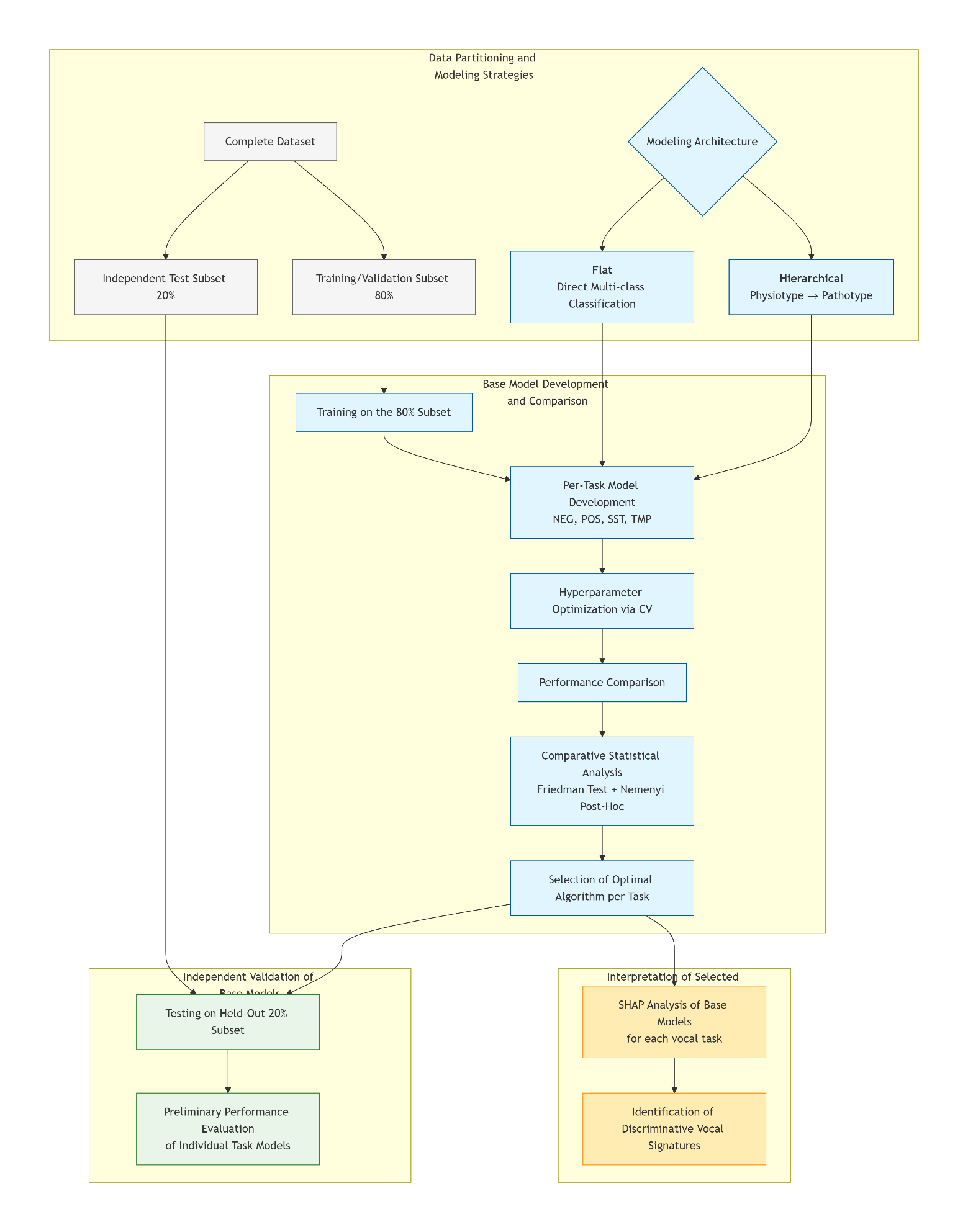
**

**Figure 4. Stacking Ensemble Construction and Final Evaluation**

**
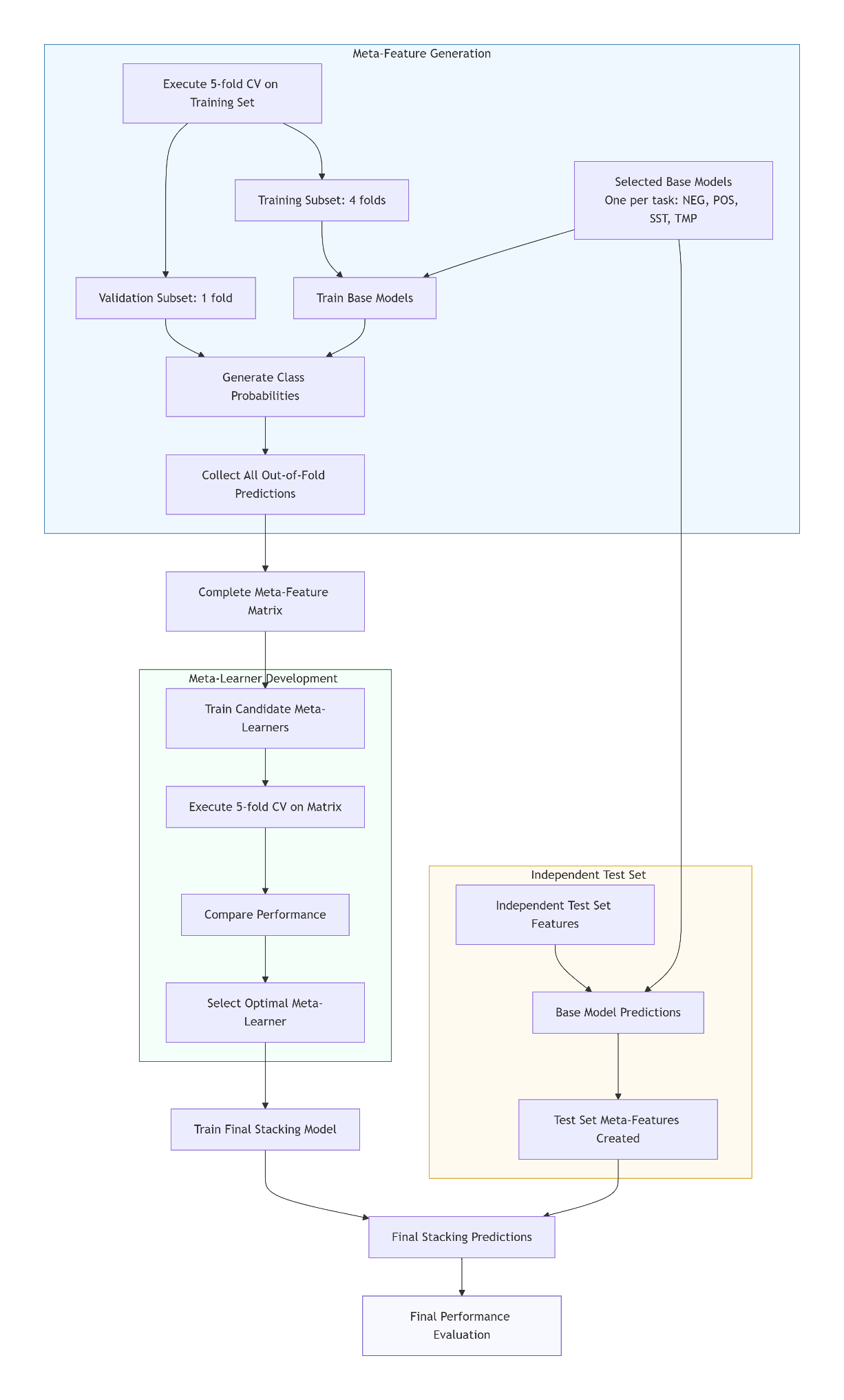
**
