# Supplementary material for: Speech-based digital biomarkers for early etiological stratification of Alzheimer’s disease and frontotemporal degeneration: a biomarker-confirmed prospective study
Source: J Prev Alzheimers Dis. 2026 Apr 17;13(6):100573. doi: 10.1016/j.tjpad.2026.100573 (PMC13098405; doi:10.1016/j.tjpad.2026.100573)
Supplement: Supplementary file 3 [file mmc3.docx]

**Supplementary Material 3 : Comparison of imbalance handling strategies for speech‑based classification**

| Level | Strategy | Best_Model | Accuracy | F1_score | AUC |
| --- | --- | --- | --- | --- | --- |
| NEG |  |  |  |  |  |
| Physiotype | SMOTE | GradientBoosting | 0.941 | 0.939 | 0.992 |
|  | ClassWeights | ExtraTrees | 0.922 | 0.917 | 0.979 |
|  | NoCorrection | ExtraTrees | 0.922 | 0.917 | 0.979 |
|  | **Oversampling** | **XGBoost** | **0.901** | **0.903** | **0.969** |
| Pathotype | SMOTE | XGBoost | 0.941 | 0.940 | 0.998 |
|  | ClassWeights | SVM | 0.941 | 0.939 | 0.995 |
|  | NoCorrection | SVM | 0.941 | 0.939 | 0.995 |
|  | **Oversampling** | **SVM** | **0.941** | **0.939** | **0.998** |
| POS |  |  |  |  |  |
| Physiotype | SMOTE | GradientBoosting | 0.970 | 0.961 | 0.957 |
|  | ClassWeights | XGBoost | 0.988 | 0.989 | 0.999 |
|  | NoCorrection | XGBoost | 0.977 | 0.969 | 0.999 |
|  | **Oversampling** | **GradientBoosting** | **0.979** | **0.979** | **0.998** |
| Pathotype | SMOTE | ExtraTrees | 0.898 | 0.888 | 0.986 |
|  | ClassWeights | ExtraTrees | 0.918 | 0.909 | 0.978 |
|  | NoCorrection | ExtraTrees | 0.918 | 0.909 | 0.978 |
|  | **Oversampling** | **KNN** | **0.918** | **0.908** | **0.919** |
| SST |  |  |  |  |  |
| Physiotype | SMOTE | ExtraTrees | 0.936 | 0.936 | 0.986 |
|  | ClassWeights | ExtraTrees | 0.940 | 0.939 | 0.987 |
|  | NoCorrection | ExtraTrees | 0.940 | 0.939 | 0.987 |
|  | **Oversampling** | **ExtraTrees** | **0.929** | **0.929** | **0.985** |
| Pathotype | SMOTE | SVM | 0.940 | 0.939 | 0.992 |
|  | ClassWeights | SVM | 0.934 | 0.934 | 0.992 |
|  | NoCorrection | SVM | 0.934 | 0.934 | 0.992 |
|  | **Oversampling** | **SVM** | **0.940** | **0.939** | **0.992** |
| MPT |  |  |  |  |  |
| Physiotype | SMOTE | GradientBoosting | 0.928 | 0.927 | 0.991 |
|  | ClassWeights | XGBoost | 0.959 | 0.958 | 0.991 |
|  | NoCorrection | XGBoost | 0.959 | 0.958 | 0.991 |
|  | **Oversampling** | **GradientBoosting** | **0.959** | **0.958** | **0.993** |
| Pathotype | SMOTE | GradientBoosting | 0.918 | 0.917 | 0.979 |
|  | ClassWeights | GradientBoosting | 0.948 | 0.948 | 0.985 |
|  | NoCorrection | GradientBoosting | 0.948 | 0.948 | 0.985 |
|  | **Oversampling** | **GradientBoosting** | **0.938** | **0.936** | **0.978** |

*Performance metrics (Accuracy, F1‑score, AUC) are reported for the best model identified through cross‑validation for each speech task (NEG, POS, SST, TMP) and each classification level (Physiotype, Pathotype). Four strategies were compared:****SMOTE****(Synthetic Minority Over‑sampling Technique),****ClassWeights****(class weight adjustment using class_weight='balanced' where applicable),****NoCorrection****(no imbalance correction), and****Oversampling****(random oversampling with RandomOverSampler, applied strictly within cross‑validation folds, which constitutes the primary method used in this study). Values are rounded to three decimal places.
Abbreviations: AUC, area under the ROC curve; F1, F1‑score; SMOTE, Synthetic Minority Over‑sampling Technique; SVM, Support Vector Machine; XGBoost, Extreme Gradient Boosting; KNN, k‑Nearest Neighbors.*
