# Supplementary material for: Speech-based digital biomarkers for early etiological stratification of Alzheimer’s disease and frontotemporal degeneration: a biomarker-confirmed prospective study
Source: J Prev Alzheimers Dis. 2026 Apr 17;13(6):100573. doi: 10.1016/j.tjpad.2026.100573 (PMC13098405; doi:10.1016/j.tjpad.2026.100573)
Supplement: Supplementary file 4 [file mmc4.docx]

| **Supplementary Table 4 : Speech Features comparisons’ significant across classifications groups** | | | | | |
| --- | --- | --- | --- | --- | --- |
| **Group_Type** | **Comparison** | **Feature** | **P_Value** | **ES** | **Interpretation** |
| **neg** |  |  |  |  |  |
| Pathotype | healthy_control_vs_amnestic_AD | contrast_mean_0 | 0,000 | 0,88 | large |
| Pathotype | healthy_control_vs_amnestic_AD | zcr_mean | 0,000 | 0,81 | large |
| Pathotype | healthy_control_vs_lvPPA_AD | jitter_rap | 0,000 | 1,00 | large |
| Pathotype | healthy_control_vs_lvPPA_AD | pause_median_duration | 0,000 | 0,99 | large |
| Pathotype | healthy_control_vs_lvPPA_AD | inter_word_pause_median | 0,000 | 0,99 | large |
| Pathotype | FTLD_vs_healthy_control | contrast_mean_0 | 0,000 | 0,89 | large |
| Pathotype | healthy_control_vs_amnestic_AD | chroma_mean_8 | 0,000 | 0,76 | large |
| Pathotype | healthy_control_vs_lvPPA_AD | contrast_mean_0 | 0,000 | 0,98 | large |
| Pathotype | healthy_control_vs_lvPPA_AD | jitter_local | 0,000 | 0,98 | large |
| Pathotype | healthy_control_vs_lvPPA_AD | jitter_ppq5 | 0,000 | 0,97 | large |
| Pathotype | healthy_control_vs_lvPPA_AD | contrast_mean_4 | 0,000 | 0,96 | large |
| Pathotype | healthy_control_vs_lvPPA_AD | contrast_mean_1 | 0,000 | 0,96 | large |
| Pathotype | healthy_control_vs_amnestic_AD | pause_median_duration | 0,000 | 0,73 | large |
| Pathotype | healthy_control_vs_amnestic_AD | inter_word_pause_median | 0,000 | 0,73 | large |
| Pathotype | healthy_control_vs_lvPPA_AD | pause_proportion | 0,000 | 0,92 | large |
| Pathotype | healthy_control_vs_lvPPA_AD | pause_ratio | 0,000 | 0,92 | large |
| Pathotype | healthy_control_vs_lvPPA_AD | semi_voyelles_count | 0,000 | 0,90 | large |
| Pathotype | healthy_control_vs_lvPPA_AD | shimmer_apq3 | 0,000 | 0,91 | large |
| Pathotype | healthy_control_vs_lvPPA_AD | shimmer_dda | 0,000 | 0,91 | large |
| Pathotype | healthy_control_vs_lvPPA_AD | syllable_count | 0,000 | 0,91 | large |
| Pathotype | healthy_control_vs_lvPPA_AD | voyelles_oral_count | 0,000 | 0,91 | large |
| Pathotype | healthy_control_vs_lvPPA_AD | phoneme_count | 0,000 | 0,90 | large |
| Pathotype | healthy_control_vs_lvPPA_AD | num_speech_phonemes | 0,000 | 0,90 | large |
| Pathotype | healthy_control_vs_lvPPA_AD | fricatives_count | 0,000 | 0,90 | large |
| Pathotype | healthy_control_vs_lvPPA_AD | long_pause_count | 0,000 | 0,89 | large |
| Pathotype | healthy_control_vs_lvPPA_AD | articulation_rate | 0,000 | 0,89 | large |
| Pathotype | healthy_control_vs_lvPPA_AD | pause_mean_duration | 0,000 | 0,90 | large |
| Pathotype | healthy_control_vs_lvPPA_AD | inter_word_pause_mean | 0,000 | 0,90 | large |
| Pathotype | healthy_control_vs_lvPPA_AD | speech_duration | 0,000 | 0,90 | large |
| Pathotype | FTLD_vs_healthy_control | hnr_mean | 0,000 | 0,80 | large |
| Pathotype | healthy_control_vs_amnestic_AD | f3_cv | 0,000 | 0,69 | large |
| Pathotype | healthy_control_vs_lvPPA_AD | spectral_flux | 0,000 | 0,89 | large |
| Pathotype | healthy_control_vs_lvPPA_AD | pause_total_duration | 0,000 | 0,88 | large |
| Pathotype | healthy_control_vs_lvPPA_AD | hnr_mean,1 | 0,000 | 0,88 | large |
| Pathotype | healthy_control_vs_lvPPA_AD | hnr_mean | 0,000 | 0,88 | large |
| Pathotype | healthy_control_vs_lvPPA_AD | shimmer_apq5 | 0,000 | 0,88 | large |
| Pathotype | healthy_control_vs_amnestic_AD | spectral_flux,1 | 0,000 | 0,68 | large |
| Pathotype | FTLD_vs_healthy_control | shimmer_local | 0,000 | 0,79 | large |
| Pathotype | healthy_control_vs_amnestic_AD | shimmer_apq3 | 0,000 | 0,67 | large |
| Pathotype | healthy_control_vs_lvPPA_AD | liquides_count | 0,000 | 0,86 | large |
| Pathotype | healthy_control_vs_lvPPA_AD | occlusives_count | 0,000 | 0,86 | large |
| Pathotype | healthy_control_vs_amnestic_AD | spectral_centroid_mean | 0,000 | 0,66 | large |
| Pathotype | healthy_control_vs_lvPPA_AD | f0_mean | 0,000 | 0,85 | large |
| Pathotype | healthy_control_vs_amnestic_AD | rolloff_mean | 0,000 | 0,65 | large |
| Pathotype | healthy_control_vs_lvPPA_AD | shimmer_local | 0,000 | 0,84 | large |
| Pathotype | healthy_control_vs_lvPPA_AD | chroma_mean_1 | 0,000 | 0,84 | large |
| Pathotype | healthy_control_vs_amnestic_AD | spectral_centroid_std | 0,000 | 0,65 | large |
| Pathotype | healthy_control_vs_lvPPA_AD | spectral_flux,1 | 0,000 | 0,83 | large |
| Pathotype | healthy_control_vs_amnestic_AD | hnr_std,1 | 0,000 | 0,64 | large |
| Pathotype | FTLD_vs_healthy_control | shimmer_apq5 | 0,000 | 0,74 | large |
| Pathotype | nfvPPA_FTLD_vs_healthy_control | semi_voyelles_cv | 0,000 | 0,99 | large |
| Pathotype | healthy_control_vs_lvPPA_AD | delta_mfcc_mean_12 | 0,000 | 0,82 | large |
| Pathotype | FTLD_vs_lvPPA_AD | jitter_rap | 0,000 | 1,00 | large |
| Pathotype | FTLD_vs_lvPPA_AD | shimmer_local | 0,000 | 1,00 | large |
| Pathotype | FTLD_vs_lvPPA_AD | shimmer_apq3 | 0,000 | 1,00 | large |
| Pathotype | FTLD_vs_lvPPA_AD | shimmer_dda | 0,000 | 1,00 | large |
| Pathotype | FTLD_vs_lvPPA_AD | hnr_mean | 0,000 | 1,00 | large |
| Pathotype | FTLD_vs_lvPPA_AD | contrast_mean_1 | 0,000 | 1,00 | large |
| Pathotype | healthy_control_vs_amnestic_AD | rolloff_std | 0,000 | 0,63 | large |
| Pathotype | nfvPPA_FTLD_vs_healthy_control | semi_voyelles_std | 0,000 | 0,98 | large |
| Pathotype | FTLD_vs_lvPPA_AD | shimmer_apq5 | 0,000 | 0,99 | large |
| Pathotype | nfvPPA_FTLD_vs_healthy_control | shimmer_local | 0,000 | 0,97 | large |
| Pathotype | healthy_control_vs_amnestic_AD | fricatives_kurtosis | 0,000 | 0,62 | large |
| Pathotype | healthy_control_vs_amnestic_AD | chroma_mean_7 | 0,000 | 0,62 | large |
| Pathotype | nfvPPA_FTLD_vs_healthy_control | shimmer_apq3 | 0,000 | 0,97 | large |
| Pathotype | nfvPPA_FTLD_vs_healthy_control | shimmer_dda | 0,000 | 0,97 | large |
| Pathotype | nfvPPA_FTLD_vs_healthy_control | semi_voyelles_count | 0,000 | 0,94 | large |
| Pathotype | healthy_control_vs_amnestic_AD | occlusives_count | 0,000 | 0,61 | large |
| Pathotype | amnestic_AD_vs_lvPPA_AD | hnr_mean,1 | 0,000 | 0,90 | large |
| Pathotype | FTLD_vs_lvPPA_AD | jitter_ppq5 | 0,000 | 0,98 | large |
| Pathotype | amnestic_AD_vs_lvPPA_AD | shimmer_apq3 | 0,000 | 0,89 | large |
| Pathotype | nfvPPA_FTLD_vs_healthy_control | shimmer_apq5 | 0,000 | 0,96 | large |
| Pathotype | healthy_control_vs_lvPPA_AD | f0_min | 0,000 | 0,77 | large |
| Pathotype | healthy_control_vs_lvPPA_AD | chroma_mean_8 | 0,000 | 0,79 | large |
| Pathotype | FTLD_vs_lvPPA_AD | jitter_local | 0,000 | 0,97 | large |
| Pathotype | healthy_control_vs_lvPPA_AD | rolloff_std | 0,000 | 0,79 | large |
| Pathotype | healthy_control_vs_amnestic_AD | contrast_mean_6 | 0,000 | 0,61 | large |
| Pathotype | nfvPPA_FTLD_vs_healthy_control | semi_voyelles_max | 0,000 | 0,95 | large |
| Pathotype | nfvPPA_FTLD_vs_healthy_control | chroma_mean_2 | 0,000 | 0,95 | large |
| Pathotype | FTLD_vs_healthy_control | shimmer_apq3 | 0,000 | 0,70 | large |
| Pathotype | FTLD_vs_healthy_control | shimmer_dda | 0,000 | 0,70 | large |
| Pathotype | svPPA_FTLD_vs_healthy_control | pause_frequency | 0,000 | 0,94 | large |
| Pathotype | FTLD_vs_healthy_control | semi_voyelles_count | 0,000 | 0,68 | large |
| Pathotype | svPPA_FTLD_vs_healthy_control | voyelles_oral_max | 0,000 | 0,94 | large |
| Pathotype | healthy_control_vs_lvPPA_AD | spectral_centroid_std | 0,000 | 0,77 | large |
| Pathotype | healthy_control_vs_lvPPA_AD | spectral_flux_mean | 0,000 | 0,77 | large |
| Pathotype | nfvPPA_FTLD_vs_healthy_control | semi_voyelles_percentile_90 | 0,000 | 0,93 | large |
| Pathotype | svPPA_FTLD_vs_healthy_control | chroma_mean_11 | 0,000 | 0,93 | large |
| Pathotype | nfvPPA_FTLD_vs_healthy_control | syllable_rate_per_sec | 0,000 | 0,93 | large |
| Pathotype | FTLD_vs_healthy_control | nasales_count | 0,000 | 0,67 | large |
| Pathotype | healthy_control_vs_amnestic_AD | jitter_local | 0,000 | 0,59 | large |
| Pathotype | healthy_control_vs_lvPPA_AD | pause_std_duration | 0,000 | 0,76 | large |
| Pathotype | healthy_control_vs_lvPPA_AD | inter_word_pause_std | 0,000 | 0,76 | large |
| Pathotype | nfvPPA_FTLD_vs_healthy_control | semi_voyelles_iqr | 0,000 | 0,92 | large |
| Pathotype | FTLD_vs_healthy_control | nasales_iqr | 0,000 | 0,68 | large |
| Pathotype | healthy_control_vs_lvPPA_AD | chroma_mean_9 | 0,000 | 0,76 | large |
| Pathotype | healthy_control_vs_lvPPA_AD | chroma_mean_10 | 0,000 | 0,76 | large |
| Pathotype | FTLD_vs_healthy_control | occlusives_count | 0,000 | 0,67 | large |
| Pathotype | healthy_control_vs_lvPPA_AD | nasales_count | 0,000 | 0,74 | large |
| Pathotype | nfvPPA_FTLD_vs_healthy_control | speech_rate_phonemes_per_sec | 0,000 | 0,91 | large |
| Pathotype | nfvPPA_FTLD_vs_healthy_control | rate_speech_phonemes | 0,000 | 0,91 | large |
| Pathotype | svPPA_FTLD_vs_healthy_control | chroma_mean_2 | 0,000 | 0,91 | large |
| Pathotype | healthy_control_vs_lvPPA_AD | chroma_mean_7 | 0,000 | 0,75 | large |
| Pathotype | svPPA_FTLD_vs_healthy_control | voyelles_oral_median | 0,000 | 0,87 | large |
| Pathotype | healthy_control_vs_amnestic_AD | liquides_count | 0,000 | 0,57 | large |
| Pathotype | lvPPA_FTLD_vs_healthy_control | occlusives_min | 0,000 | 0,97 | large |
| Pathotype | svPPA_FTLD_vs_healthy_control | delta_mfcc_mean_5 | 0,000 | 0,89 | large |
| Pathotype | FTLD_vs_healthy_control | nasales_median | 0,000 | 0,65 | large |
| Pathotype | svPPA_FTLD_vs_amnestic_AD | pause_frequency | 0,000 | 0,98 | large |
| Pathotype | healthy_control_vs_lvPPA_AD | pause_max_duration | 0,000 | 0,73 | large |
| Pathotype | healthy_control_vs_lvPPA_AD | inter_word_pause_max | 0,000 | 0,73 | large |
| Pathotype | svPPA_FTLD_vs_healthy_control | f0_slope | 0,000 | 0,88 | large |
| Pathotype | healthy_control_vs_amnestic_AD | contrast_mean_1 | 0,000 | 0,56 | large |
| Pathotype | healthy_control_vs_amnestic_AD | fricatives_iqr | 0,000 | 0,56 | large |
| Pathotype | lvPPA_FTLD_vs_healthy_control | semi_voyelles_count | 0,000 | 1,00 | large |
| Pathotype | lvPPA_FTLD_vs_healthy_control | nasales_count | 0,000 | 1,00 | large |
| Pathotype | nfvPPA_FTLD_vs_healthy_control | occlusives_count | 0,000 | 0,87 | large |
| Pathotype | FTLD_vs_healthy_control | fricatives_count | 0,000 | 0,64 | large |
| Pathotype | FTLD_vs_healthy_control | nasales_mean | 0,000 | 0,65 | large |
| Pathotype | FTLD_vs_healthy_control | hnr_std | 0,000 | 0,64 | large |
| Pathotype | lvPPA_FTLD_vs_healthy_control | num_pauses | 0,000 | 1,00 | large |
| Pathotype | lvPPA_FTLD_vs_healthy_control | num_inter_word_pauses | 0,000 | 1,00 | large |
| Pathotype | svPPA_FTLD_vs_healthy_control | semi_voyelles_percentile_10 | 0,000 | 0,85 | large |
| Pathotype | svPPA_FTLD_vs_healthy_control | voyelles_oral_std | 0,000 | 0,87 | large |
| Pathotype | lvPPA_FTLD_vs_healthy_control | num_inter_pauses | 0,000 | 1,00 | large |
| Pathotype | FTLD_vs_healthy_control | phoneme_count | 0,000 | 0,64 | large |
| Pathotype | FTLD_vs_healthy_control | num_speech_phonemes | 0,000 | 0,64 | large |
| Pathotype | lvPPA_FTLD_vs_healthy_control | median_phoneme_duration | 0,000 | 0,96 | large |
| Pathotype | lvPPA_FTLD_vs_healthy_control | semi_voyelles_mean | 0,000 | 1,00 | large |
| Pathotype | lvPPA_FTLD_vs_healthy_control | min_phoneme_duration | 0,000 | 0,92 | large |
| Pathotype | nfvPPA_FTLD_vs_healthy_control | contrast_mean_0 | 0,000 | 0,86 | large |
| Pathotype | lvPPA_FTLD_vs_healthy_control | long_pause_count | 0,000 | 0,99 | large |
| Pathotype | lvPPA_FTLD_vs_healthy_control | occlusives_count | 0,000 | 1,00 | large |
| Pathotype | lvPPA_FTLD_vs_healthy_control | occlusives_iqr | 0,000 | 1,00 | large |
| Pathotype | svPPA_FTLD_vs_healthy_control | chroma_mean_0 | 0,000 | 0,86 | large |
| Pathotype | lvPPA_FTLD_vs_healthy_control | semi_voyelles_std | 0,000 | 1,00 | large |
| Pathotype | lvPPA_FTLD_vs_healthy_control | nasales_iqr | 0,000 | 1,00 | large |
| Pathotype | lvPPA_FTLD_vs_healthy_control | syllable_count | 0,000 | 1,00 | large |
| Pathotype | lvPPA_FTLD_vs_healthy_control | voyelles_oral_count | 0,000 | 1,00 | large |
| Pathotype | lvPPA_FTLD_vs_healthy_control | liquides_percentile_90 | 0,000 | 1,00 | large |
| Pathotype | lvPPA_FTLD_vs_healthy_control | phoneme_count | 0,000 | 1,00 | large |
| Pathotype | lvPPA_FTLD_vs_healthy_control | num_speech_phonemes | 0,000 | 1,00 | large |
| Pathotype | lvPPA_FTLD_vs_healthy_control | delta_mfcc_mean_1 | 0,000 | 1,00 | large |
| Pathotype | svPPA_FTLD_vs_healthy_control | voyelles_oral_mean | 0,000 | 0,85 | large |
| Pathotype | lvPPA_FTLD_vs_healthy_control | semi_voyelles_cv | 0,000 | 1,00 | large |
| Pathotype | lvPPA_FTLD_vs_healthy_control | occlusives_percentile_90 | 0,000 | 1,00 | large |
| Pathotype | lvPPA_FTLD_vs_healthy_control | liquides_max | 0,000 | 1,00 | large |
| Pathotype | lvPPA_FTLD_vs_healthy_control | semi_voyelles_max | 0,000 | 1,00 | large |
| Pathotype | lvPPA_FTLD_vs_healthy_control | occlusives_max | 0,000 | 1,00 | large |
| Pathotype | lvPPA_FTLD_vs_healthy_control | semi_voyelles_percentile_90 | 0,000 | 1,00 | large |
| Pathotype | lvPPA_FTLD_vs_healthy_control | liquides_mean | 0,000 | 1,00 | large |
| Pathotype | lvPPA_FTLD_vs_healthy_control | nasales_std | 0,000 | 1,00 | large |
| Pathotype | lvPPA_FTLD_vs_healthy_control | speech_duration | 0,000 | 1,00 | large |
| Pathotype | lvPPA_FTLD_vs_healthy_control | delta_mfcc_mean_7 | 0,000 | 1,00 | large |
| Pathotype | lvPPA_FTLD_vs_healthy_control | contrast_mean_0 | 0,000 | 1,00 | large |
| Pathotype | lvPPA_FTLD_vs_healthy_control | contrast_mean_4 | 0,000 | 1,00 | large |
| Pathotype | lvPPA_FTLD_vs_healthy_control | total_duration | 0,000 | 1,00 | large |
| Pathotype | nfvPPA_FTLD_vs_healthy_control | delta_mfcc_mean_12 | 0,000 | 0,85 | large |
| Pathotype | lvPPA_FTLD_vs_healthy_control | semi_voyelles_median | 0,000 | 0,99 | large |
| Pathotype | nfvPPA_FTLD_vs_healthy_control | phoneme_count | 0,000 | 0,85 | large |
| Pathotype | nfvPPA_FTLD_vs_healthy_control | num_speech_phonemes | 0,000 | 0,85 | large |
| Pathotype | lvPPA_FTLD_vs_healthy_control | spectral_flux | 0,000 | 1,00 | large |
| Pathotype | lvPPA_FTLD_vs_healthy_control | fricatives_count | 0,000 | 0,99 | large |
| Pathotype | lvPPA_FTLD_vs_healthy_control | nasales_max | 0,000 | 0,99 | large |
| Pathotype | lvPPA_FTLD_vs_healthy_control | nasales_cv | 0,000 | 0,99 | large |
| Pathotype | lvPPA_FTLD_vs_healthy_control | spectral_flux_mean | 0,000 | 0,99 | large |
| Pathotype | svPPA_FTLD_vs_healthy_control | liquides_median | 0,000 | 0,85 | large |
| Pathotype | nfvPPA_FTLD_vs_healthy_control | syllable_count | 0,000 | 0,85 | large |
| Pathotype | nfvPPA_FTLD_vs_healthy_control | voyelles_oral_count | 0,000 | 0,85 | large |
| Pathotype | healthy_control_vs_lvPPA_AD | f0_max | 0,000 | 0,70 | large |
| Pathotype | lvPPA_FTLD_vs_healthy_control | delta_mfcc_mean_12 | 0,000 | 0,99 | large |
| Pathotype | lvPPA_FTLD_vs_healthy_control | voyelles_oral_skewness | 0,000 | 0,99 | large |
| Pathotype | healthy_control_vs_amnestic_AD | nasales_iqr | 0,000 | 0,54 | large |
| Pathotype | svPPA_FTLD_vs_amnestic_AD | spectral_instability,1 | 0,000 | 0,93 | large |
| Pathotype | lvPPA_FTLD_vs_healthy_control | semi_voyelles_iqr | 0,000 | 0,99 | large |
| Pathotype | lvPPA_FTLD_vs_amnestic_AD | min_phoneme_duration | 0,000 | 0,99 | large |
| Pathotype | healthy_control_vs_amnestic_AD | nasales_cv | 0,000 | 0,53 | large |
| Pathotype | svPPA_FTLD_vs_healthy_control | contrast_mean_2 | 0,000 | 0,84 | large |
| Pathotype | lvPPA_FTLD_vs_healthy_control | rolloff_mean | 0,000 | 0,98 | large |
| Pathotype | amnestic_AD_vs_lvPPA_AD | delta_mfcc_mean_12 | 0,000 | 0,78 | large |
| Pathotype | healthy_control_vs_lvPPA_AD | fricatives_kurtosis | 0,000 | 0,68 | large |
| Pathotype | healthy_control_vs_amnestic_AD | fricatives_skewness | 0,000 | 0,53 | large |
| Pathotype | amnestic_AD_vs_lvPPA_AD | articulation_rate | 0,000 | 0,72 | large |
| Pathotype | lvPPA_FTLD_vs_healthy_control | pause_min_duration | 0,000 | 0,95 | large |
| Pathotype | lvPPA_FTLD_vs_healthy_control | inter_word_pause_min | 0,000 | 0,95 | large |
| Pathotype | nfvPPA_FTLD_vs_healthy_control | chroma_mean_3 | 0,000 | 0,83 | large |
| Pathotype | nfvPPA_FTLD_vs_amnestic_AD | spectral_flux,1 | 0,000 | 0,92 | large |
| Pathotype | amnestic_AD_vs_lvPPA_AD | hnr_mean | 0,000 | 0,77 | large |
| Pathotype | lvPPA_FTLD_vs_healthy_control | max_phoneme_duration | 0,000 | 0,96 | large |
| Pathotype | lvPPA_FTLD_vs_healthy_control | intensity_slope | 0,000 | 0,97 | large |
| Pathotype | lvPPA_FTLD_vs_healthy_control | occlusives_std | 0,000 | 0,97 | large |
| Pathotype | lvPPA_FTLD_vs_healthy_control | nasales_percentile_90 | 0,000 | 0,97 | large |
| Pathotype | FTLD_vs_healthy_control | articulation_rate | 0,000 | 0,61 | large |
| Pathotype | nfvPPA_FTLD_vs_amnestic_AD | shimmer_local | 0,000 | 0,91 | large |
| Pathotype | amnestic_AD_vs_lvPPA_AD | shimmer_dda | 0,000 | 0,77 | large |
| Pathotype | healthy_control_vs_amnestic_AD | fricatives_max | 0,000 | 0,52 | large |
| Pathotype | lvPPA_FTLD_vs_healthy_control | liquides_count | 0,000 | 0,96 | large |
| Pathotype | nfvPPA_FTLD_vs_healthy_control | semi_voyelles_skewness | 0,000 | 0,82 | large |
| Pathotype | svPPA_FTLD_vs_healthy_control | contrast_mean_4 | 0,000 | 0,82 | large |
| Pathotype | svPPA_FTLD_vs_amnestic_AD | chroma_mean_11 | 0,000 | 0,91 | large |
| Pathotype | svPPA_FTLD_vs_healthy_control | jitter_rap | 0,000 | 0,82 | large |
| Pathotype | svPPA_FTLD_vs_healthy_control | occlusives_percentile_10 | 0,000 | 0,82 | large |
| Pathotype | nfvPPA_FTLD_vs_amnestic_AD | semi_voyelles_cv | 0,000 | 0,91 | large |
| Pathotype | lvPPA_FTLD_vs_healthy_control | fricatives_max | 0,000 | 0,96 | large |
| Pathotype | lvPPA_FTLD_vs_healthy_control | delta_mfcc_mean_4 | 0,000 | 0,96 | large |
| Pathotype | lvPPA_FTLD_vs_healthy_control | delta_mfcc_mean_8 | 0,000 | 0,96 | large |
| Pathotype | lvPPA_FTLD_vs_healthy_control | liquides_iqr | 0,000 | 0,96 | large |
| Pathotype | lvPPA_FTLD_vs_healthy_control | liquides_std | 0,000 | 0,96 | large |
| Pathotype | healthy_control_vs_amnestic_AD | nasales_std | 0,000 | 0,52 | large |
| Pathotype | svPPA_FTLD_vs_healthy_control | jitter_ppq5 | 0,000 | 0,82 | large |
| Pathotype | svPPA_FTLD_vs_amnestic_AD | delta_mfcc_mean_5 | 0,000 | 0,90 | large |
| Pathotype | nfvPPA_FTLD_vs_amnestic_AD | shimmer_apq5 | 0,000 | 0,90 | large |
| Pathotype | svPPA_FTLD_vs_healthy_control | voyelles_oral_cv | 0,000 | 0,82 | large |
| Pathotype | lvPPA_FTLD_vs_healthy_control | liquides_cv | 0,000 | 0,95 | large |
| Pathotype | lvPPA_FTLD_vs_healthy_control | kurt_phoneme_duration | 0,000 | 0,95 | large |
| Pathotype | svPPA_FTLD_vs_healthy_control | occlusives_median | 0,000 | 0,80 | large |
| Pathotype | lvPPA_FTLD_vs_healthy_control | rmse_mean | 0,000 | 0,95 | large |
| Pathotype | lvPPA_FTLD_vs_healthy_control | intensity_mean | 0,000 | 0,95 | large |
| Pathotype | svPPA_FTLD_vs_healthy_control | delta_mfcc_mean_12 | 0,000 | 0,81 | large |
| Pathotype | healthy_control_vs_lvPPA_AD | pause_min_duration | 0,000 | 0,65 | large |
| Pathotype | healthy_control_vs_lvPPA_AD | inter_word_pause_min | 0,000 | 0,65 | large |
| Pathotype | healthy_control_vs_lvPPA_AD | f3_cv | 0,000 | 0,67 | large |
| Pathotype | FTLD_vs_healthy_control | num_inter_pauses | 0,000 | 0,59 | large |
| Pathotype | healthy_control_vs_amnestic_AD | spectral_flux | 0,000 | 0,51 | large |
| Pathotype | amnestic_AD_vs_lvPPA_AD | long_pause_count | 0,000 | 0,75 | large |
| Pathotype | nfvPPA_FTLD_vs_healthy_control | fricatives_kurtosis | 0,000 | 0,81 | large |
| Pathotype | FTLD_vs_healthy_control | speech_duration | 0,000 | 0,60 | large |
| Pathotype | FTLD_vs_lvPPA_AD | hnr_mean,1 | 0,000 | 0,82 | large |
| Pathotype | FTLD_vs_healthy_control | delta_mfcc_mean_1 | 0,000 | 0,60 | large |
| Pathotype | healthy_control_vs_lvPPA_AD | chroma_mean_6 | 0,000 | 0,66 | large |
| Pathotype | FTLD_vs_healthy_control | num_pauses | 0,000 | 0,59 | large |
| Pathotype | FTLD_vs_healthy_control | num_inter_word_pauses | 0,000 | 0,59 | large |
| Pathotype | lvPPA_FTLD_vs_healthy_control | nasales_skewness | 0,000 | 0,95 | large |
| Pathotype | healthy_control_vs_lvPPA_AD | total_duration | 0,000 | 0,66 | large |
| Pathotype | lvPPA_FTLD_vs_healthy_control | voyelles_oral_kurtosis | 0,000 | 0,95 | large |
| Pathotype | amnestic_AD_vs_lvPPA_AD | nasales_count | 0,000 | 0,75 | large |
| Pathotype | svPPA_FTLD_vs_lvPPA_AD | pause_max_duration | 0,000 | 1,00 | large |
| Pathotype | svPPA_FTLD_vs_lvPPA_AD | inter_word_pause_max | 0,000 | 1,00 | large |
| Pathotype | healthy_control_vs_lvPPA_AD | f3_cv,1 | 0,000 | 0,66 | large |
| Pathotype | FTLD_vs_healthy_control | chroma_mean_7 | 0,000 | 0,60 | large |
| Pathotype | nfvPPA_FTLD_vs_lvPPA_AD | spectral_flux | 0,000 | 1,00 | large |
| Pathotype | nfvPPA_FTLD_vs_lvPPA_AD | spectral_flux,1 | 0,000 | 1,00 | large |
| Pathotype | svPPA_FTLD_vs_lvPPA_AD | jitter_rap | 0,000 | 1,00 | large |
| Pathotype | svPPA_FTLD_vs_lvPPA_AD | spectral_flux | 0,000 | 1,00 | large |
| Pathotype | svPPA_FTLD_vs_lvPPA_AD | spectral_instability | 0,000 | 1,00 | large |
| Pathotype | svPPA_FTLD_vs_lvPPA_AD | hnr_mean | 0,000 | 1,00 | large |
| Pathotype | svPPA_FTLD_vs_lvPPA_AD | chroma_mean_0 | 0,000 | 1,00 | large |
| Pathotype | svPPA_FTLD_vs_lvPPA_AD | chroma_mean_1 | 0,000 | 1,00 | large |
| Pathotype | svPPA_FTLD_vs_lvPPA_AD | contrast_mean_1 | 0,000 | 1,00 | large |
| Pathotype | svPPA_FTLD_vs_lvPPA_AD | spectral_flux,1 | 0,000 | 1,00 | large |
| Pathotype | svPPA_FTLD_vs_lvPPA_AD | spectral_instability,1 | 0,000 | 1,00 | large |
| Pathotype | svPPA_FTLD_vs_lvPPA_AD | hnr_mean,1 | 0,000 | 1,00 | large |
| Pathotype | nfvPPA_FTLD_vs_lvPPA_AD | jitter_local | 0,000 | 1,00 | large |
| Pathotype | nfvPPA_FTLD_vs_lvPPA_AD | jitter_rap | 0,000 | 1,00 | large |
| Pathotype | nfvPPA_FTLD_vs_lvPPA_AD | jitter_ppq5 | 0,000 | 1,00 | large |
| Pathotype | nfvPPA_FTLD_vs_lvPPA_AD | shimmer_local | 0,000 | 1,00 | large |
| Pathotype | nfvPPA_FTLD_vs_lvPPA_AD | shimmer_apq3 | 0,000 | 1,00 | large |
| Pathotype | nfvPPA_FTLD_vs_lvPPA_AD | shimmer_apq5 | 0,000 | 1,00 | large |
| Pathotype | nfvPPA_FTLD_vs_lvPPA_AD | shimmer_dda | 0,000 | 1,00 | large |
| Pathotype | nfvPPA_FTLD_vs_lvPPA_AD | chroma_mean_2 | 0,000 | 1,00 | large |
| Pathotype | svPPA_FTLD_vs_lvPPA_AD | pause_mean_duration | 0,000 | 1,00 | large |
| Pathotype | svPPA_FTLD_vs_lvPPA_AD | pause_median_duration | 0,000 | 1,00 | large |
| Pathotype | svPPA_FTLD_vs_lvPPA_AD | pause_std_duration | 0,000 | 1,00 | large |
| Pathotype | svPPA_FTLD_vs_lvPPA_AD | inter_word_pause_mean | 0,000 | 1,00 | large |
| Pathotype | svPPA_FTLD_vs_lvPPA_AD | inter_word_pause_median | 0,000 | 1,00 | large |
| Pathotype | svPPA_FTLD_vs_lvPPA_AD | inter_word_pause_std | 0,000 | 1,00 | large |
| Pathotype | svPPA_FTLD_vs_healthy_control | jitter_local | 0,000 | 0,81 | large |
| Pathotype | FTLD_vs_lvPPA_AD | f0_mean | 0,000 | 0,82 | large |
| Pathotype | healthy_control_vs_amnestic_AD | contrast_mean_2 | 0,000 | 0,51 | large |
| Pathotype | lvPPA_FTLD_vs_healthy_control | f3_cv | 0,000 | 0,94 | large |
| Pathotype | amnestic_AD_vs_lvPPA_AD | f0_mean | 0,000 | 0,75 | large |
| Pathotype | amnestic_AD_vs_lvPPA_AD | hnr_std,1 | 0,000 | 0,75 | large |
| Pathotype | svPPA_FTLD_vs_amnestic_AD | semi_voyelles_min | 0,000 | 0,84 | large |
| Pathotype | svPPA_FTLD_vs_healthy_control | chroma_mean_6 | 0,000 | 0,80 | large |
| Pathotype | nfvPPA_FTLD_vs_healthy_control | hnr_mean | 0,000 | 0,80 | large |
| Pathotype | nfvPPA_FTLD_vs_FTLD | syllable_rate_per_sec | 0,000 | 0,94 | large |
| Pathotype | nfvPPA_FTLD_vs_healthy_control | liquides_count | 0,000 | 0,79 | large |
| Pathotype | healthy_control_vs_amnestic_AD | phoneme_count | 0,000 | 0,51 | large |
| Pathotype | healthy_control_vs_amnestic_AD | num_speech_phonemes | 0,000 | 0,51 | large |
| Pathotype | healthy_control_vs_amnestic_AD | liquides_percentile_90 | 0,000 | 0,50 | large |
| Pathotype | lvPPA_FTLD_vs_healthy_control | liquides_median | 0,000 | 0,93 | large |
| Pathotype | lvPPA_FTLD_vs_healthy_control | mean_phoneme_duration | 0,000 | 0,93 | large |
| Pathotype | lvPPA_FTLD_vs_healthy_control | speech_rate_phonemes_per_sec | 0,000 | 0,93 | large |
| Pathotype | lvPPA_FTLD_vs_healthy_control | rate_speech_phonemes | 0,000 | 0,93 | large |
| Pathotype | svPPA_FTLD_vs_lvPPA_AD | shimmer_apq5 | 0,000 | 0,99 | large |
| Pathotype | svPPA_FTLD_vs_lvPPA_AD | chroma_mean_11 | 0,000 | 0,99 | large |
| Pathotype | svPPA_FTLD_vs_lvPPA_AD | pause_frequency | 0,000 | 0,99 | large |
| Pathotype | FTLD_vs_healthy_control | nasales_percentile_90 | 0,000 | 0,59 | large |
| Pathotype | healthy_control_vs_amnestic_AD | jitter_rap | 0,000 | 0,50 | large |
| Pathotype | svPPA_FTLD_vs_healthy_control | f3_cv | 0,000 | 0,79 | large |
| Pathotype | lvPPA_FTLD_vs_healthy_control | contrast_mean_6 | 0,000 | 0,93 | large |
| Pathotype | svPPA_FTLD_vs_healthy_control | semi_voyelles_median | 0,000 | 0,79 | large |
| Pathotype | lvPPA_FTLD_vs_amnestic_AD | long_pause_count | 0,000 | 1,00 | large |
| Pathotype | lvPPA_FTLD_vs_amnestic_AD | num_pauses | 0,000 | 1,00 | large |
| Pathotype | lvPPA_FTLD_vs_amnestic_AD | num_inter_word_pauses | 0,000 | 1,00 | large |
| Pathotype | lvPPA_FTLD_vs_amnestic_AD | semi_voyelles_count | 0,000 | 1,00 | large |
| Pathotype | lvPPA_FTLD_vs_amnestic_AD | num_inter_pauses | 0,000 | 1,00 | large |
| Pathotype | lvPPA_FTLD_vs_amnestic_AD | occlusives_count | 0,000 | 1,00 | large |
| Pathotype | lvPPA_FTLD_vs_amnestic_AD | delta_mfcc_mean_1 | 0,000 | 1,00 | large |
| Pathotype | lvPPA_FTLD_vs_amnestic_AD | phoneme_count | 0,000 | 1,00 | large |
| Pathotype | lvPPA_FTLD_vs_amnestic_AD | num_speech_phonemes | 0,000 | 1,00 | large |
| Pathotype | lvPPA_FTLD_vs_lvPPA_AD | f0_min | 0,000 | 0,86 | large |
| Pathotype | nfvPPA_FTLD_vs_healthy_control | occlusives_iqr | 0,000 | 0,78 | large |
| Pathotype | lvPPA_FTLD_vs_amnestic_AD | semi_voyelles_std | 0,000 | 1,00 | large |
| Pathotype | lvPPA_FTLD_vs_amnestic_AD | semi_voyelles_iqr | 0,000 | 1,00 | large |
| Pathotype | lvPPA_FTLD_vs_amnestic_AD | occlusives_percentile_90 | 0,000 | 1,00 | large |
| Pathotype | lvPPA_FTLD_vs_amnestic_AD | nasales_std | 0,000 | 1,00 | large |
| Pathotype | lvPPA_FTLD_vs_amnestic_AD | nasales_max | 0,000 | 1,00 | large |
| Pathotype | lvPPA_FTLD_vs_amnestic_AD | semi_voyelles_cv | 0,000 | 1,00 | large |
| Pathotype | lvPPA_FTLD_vs_amnestic_AD | nasales_count | 0,000 | 1,00 | large |
| Pathotype | lvPPA_FTLD_vs_amnestic_AD | nasales_cv | 0,000 | 1,00 | large |
| Pathotype | lvPPA_FTLD_vs_amnestic_AD | syllable_count | 0,000 | 1,00 | large |
| Pathotype | lvPPA_FTLD_vs_amnestic_AD | voyelles_oral_count | 0,000 | 1,00 | large |
| Pathotype | lvPPA_FTLD_vs_amnestic_AD | delta_mfcc_mean_12 | 0,000 | 1,00 | large |
| Pathotype | lvPPA_FTLD_vs_healthy_control | chroma_mean_5 | 0,000 | 0,92 | large |
| Pathotype | lvPPA_FTLD_vs_healthy_control | spectral_centroid_slope | 0,000 | 0,92 | large |
| Pathotype | lvPPA_FTLD_vs_amnestic_AD | speech_duration | 0,000 | 1,00 | large |
| Pathotype | svPPA_FTLD_vs_lvPPA_AD | f0_min | 0,000 | 0,80 | large |
| Pathotype | lvPPA_FTLD_vs_amnestic_AD | occlusives_max | 0,000 | 1,00 | large |
| Pathotype | lvPPA_FTLD_vs_amnestic_AD | nasales_iqr | 0,000 | 1,00 | large |
| Pathotype | svPPA_FTLD_vs_healthy_control | intensity_dynamic_range | 0,000 | 0,79 | large |
| Pathotype | lvPPA_FTLD_vs_amnestic_AD | voyelles_oral_kurtosis | 0,000 | 1,00 | large |
| Pathotype | nfvPPA_FTLD_vs_healthy_control | contrast_mean_4 | 0,000 | 0,79 | large |
| Pathotype | svPPA_FTLD_vs_healthy_control | chroma_mean_10 | 0,000 | 0,79 | large |
| Pathotype | lvPPA_FTLD_vs_amnestic_AD | nasales_percentile_90 | 0,000 | 1,00 | large |
| Pathotype | lvPPA_FTLD_vs_amnestic_AD | delta_mfcc_mean_7 | 0,000 | 1,00 | large |
| Pathotype | lvPPA_FTLD_vs_amnestic_AD | delta_mfcc_mean_9 | 0,000 | 1,00 | large |
| Pathotype | lvPPA_FTLD_vs_amnestic_AD | chroma_mean_8 | 0,000 | 1,00 | large |
| Pathotype | lvPPA_FTLD_vs_amnestic_AD | contrast_mean_4 | 0,000 | 1,00 | large |
| Pathotype | lvPPA_FTLD_vs_amnestic_AD | rmse_mean | 0,000 | 1,00 | large |
| Pathotype | lvPPA_FTLD_vs_amnestic_AD | intensity_mean | 0,000 | 1,00 | large |
| Pathotype | lvPPA_FTLD_vs_amnestic_AD | voyelles_oral_skewness | 0,000 | 1,00 | large |
| Pathotype | svPPA_FTLD_vs_lvPPA_AD | articulation_rate | 0,000 | 0,80 | large |
| Pathotype | amnestic_AD_vs_lvPPA_AD | contrast_mean_1 | 0,000 | 0,73 | large |
| Pathotype | healthy_control_vs_amnestic_AD | b1 | 0,000 | 0,50 | medium |
| Pathotype | lvPPA_FTLD_vs_healthy_control | f3_cv,1 | 0,000 | 0,92 | large |
| Pathotype | lvPPA_FTLD_vs_amnestic_AD | fricatives_count | 0,000 | 0,99 | large |
| Pathotype | healthy_control_vs_amnestic_AD | f1_cv,1 | 0,000 | 0,50 | medium |
| Pathotype | nfvPPA_FTLD_vs_amnestic_AD | delta_mfcc_mean_12 | 0,000 | 0,86 | large |
| Pathotype | FTLD_vs_healthy_control | syllable_count | 0,000 | 0,58 | large |
| Pathotype | FTLD_vs_healthy_control | voyelles_oral_count | 0,000 | 0,58 | large |
| Pathotype | healthy_control_vs_lvPPA_AD | rmse_mean | 0,000 | 0,64 | large |
| Pathotype | healthy_control_vs_lvPPA_AD | intensity_mean | 0,000 | 0,64 | large |
| Pathotype | nfvPPA_FTLD_vs_healthy_control | std_phoneme_duration | 0,000 | 0,78 | large |
| Pathotype | FTLD_vs_lvPPA_AD | hnr_std | 0,000 | 0,79 | large |
| Pathotype | nfvPPA_FTLD_vs_healthy_control | liquides_median | 0,000 | 0,78 | large |
| Pathotype | lvPPA_FTLD_vs_healthy_control | fricatives_skewness | 0,000 | 0,91 | large |
| Pathotype | lvPPA_FTLD_vs_healthy_control | skew_phoneme_duration | 0,000 | 0,91 | large |
| Pathotype | healthy_control_vs_amnestic_AD | fricatives_min | 0,000 | 0,48 | medium |
| Pathotype | nfvPPA_FTLD_vs_amnestic_AD | jitter_local | 0,000 | 0,86 | large |
| Pathotype | lvPPA_FTLD_vs_healthy_control | voyelles_oral_max | 0,000 | 0,91 | large |
| Pathotype | amnestic_AD_vs_lvPPA_AD | semi_voyelles_count | 0,000 | 0,72 | large |
| Pathotype | svPPA_FTLD_vs_healthy_control | spectral_instability | 0,000 | 0,78 | large |
| Pathotype | nfvPPA_FTLD_vs_healthy_control | liquides_skewness | 0,000 | 0,78 | large |
| Pathotype | svPPA_FTLD_vs_healthy_control | speech_rate_phonemes_per_sec | 0,000 | 0,78 | large |
| Pathotype | svPPA_FTLD_vs_healthy_control | rate_speech_phonemes | 0,000 | 0,78 | large |
| Pathotype | nfvPPA_FTLD_vs_amnestic_AD | semi_voyelles_std | 0,000 | 0,86 | large |
| Pathotype | amnestic_AD_vs_lvPPA_AD | pause_median_duration | 0,000 | 0,72 | large |
| Pathotype | amnestic_AD_vs_lvPPA_AD | inter_word_pause_median | 0,000 | 0,72 | large |
| Pathotype | healthy_control_vs_amnestic_AD | fricatives_mean | 0,000 | 0,49 | medium |
| Pathotype | svPPA_FTLD_vs_lvPPA_AD | jitter_local | 0,000 | 0,96 | large |
| Pathotype | svPPA_FTLD_vs_lvPPA_AD | chroma_mean_10 | 0,000 | 0,96 | large |
| Pathotype | svPPA_FTLD_vs_healthy_control | f3_cv,1 | 0,000 | 0,78 | large |
| Pathotype | lvPPA_FTLD_vs_healthy_control | occlusives_mean | 0,000 | 0,91 | large |
| Pathotype | lvPPA_FTLD_vs_amnestic_AD | total_duration | 0,000 | 0,98 | large |
| Pathotype | lvPPA_FTLD_vs_healthy_control | liquides_skewness | 0,000 | 0,90 | large |
| Pathotype | nfvPPA_FTLD_vs_healthy_control | speech_duration | 0,000 | 0,77 | large |
| Pathotype | svPPA_FTLD_vs_FTLD | semi_voyelles_min | 0,000 | 0,89 | large |
| Pathotype | lvPPA_FTLD_vs_healthy_control | f2_cv | 0,000 | 0,90 | large |
| Pathotype | nfvPPA_FTLD_vs_healthy_control | nasales_median | 0,000 | 0,76 | large |
| Pathotype | FTLD_vs_lvPPA_AD | f0_max | 0,000 | 0,78 | large |
| Pathotype | lvPPA_FTLD_vs_amnestic_AD | rolloff_mean | 0,000 | 0,98 | large |
| Pathotype | FTLD_vs_healthy_control | semi_voyelles_iqr | 0,000 | 0,57 | large |
| Pathotype | svPPA_FTLD_vs_amnestic_AD | spectral_flux,1 | 0,000 | 0,85 | large |
| Pathotype | svPPA_FTLD_vs_healthy_control | fricatives_max | 0,000 | 0,77 | large |
| Pathotype | healthy_control_vs_lvPPA_AD | occlusives_iqr | 0,000 | 0,63 | large |
| Pathotype | nfvPPA_FTLD_vs_healthy_control | delta_mfcc_mean_4 | 0,000 | 0,77 | large |
| Pathotype | lvPPA_FTLD_vs_healthy_control | contrast_mean_2 | 0,000 | 0,90 | large |
| Pathotype | healthy_control_vs_amnestic_AD | shimmer_dda | 0,000 | 0,49 | medium |
| Pathotype | svPPA_FTLD_vs_FTLD | pause_frequency | 0,000 | 0,90 | large |
| Pathotype | nfvPPA_FTLD_vs_healthy_control | occlusives_mean | 0,000 | 0,77 | large |
| Pathotype | svPPA_FTLD_vs_lvPPA_AD | shimmer_local | 0,000 | 0,95 | large |
| Pathotype | svPPA_FTLD_vs_lvPPA_AD | shimmer_apq3 | 0,000 | 0,95 | large |
| Pathotype | svPPA_FTLD_vs_lvPPA_AD | shimmer_dda | 0,000 | 0,95 | large |
| Pathotype | svPPA_FTLD_vs_lvPPA_AD | chroma_mean_2 | 0,000 | 0,95 | large |
| Pathotype | svPPA_FTLD_vs_lvPPA_AD | contrast_mean_2 | 0,000 | 0,95 | large |
| Pathotype | svPPA_FTLD_vs_lvPPA_AD | pause_proportion | 0,000 | 0,95 | large |
| Pathotype | svPPA_FTLD_vs_lvPPA_AD | pause_ratio | 0,000 | 0,95 | large |
| Pathotype | lvPPA_FTLD_vs_amnestic_AD | spectral_flux_mean | 0,000 | 0,97 | large |
| Pathotype | lvPPA_FTLD_vs_amnestic_AD | hnr_std,1 | 0,000 | 0,97 | large |
| Pathotype | svPPA_FTLD_vs_FTLD | semi_voyelles_percentile_10 | 0,000 | 0,90 | large |
| Pathotype | amnestic_AD_vs_lvPPA_AD | f0_max | 0,000 | 0,71 | large |
| Pathotype | svPPA_FTLD_vs_healthy_control | fricatives_kurtosis | 0,000 | 0,76 | large |
| Pathotype | healthy_control_vs_amnestic_AD | pause_mean_duration | 0,000 | 0,48 | medium |
| Pathotype | healthy_control_vs_amnestic_AD | inter_word_pause_mean | 0,000 | 0,48 | medium |
| Pathotype | svPPA_FTLD_vs_healthy_control | spectral_instability,1 | 0,000 | 0,76 | large |
| Pathotype | lvPPA_FTLD_vs_amnestic_AD | semi_voyelles_percentile_90 | 0,000 | 0,96 | large |
| Pathotype | lvPPA_FTLD_vs_amnestic_AD | occlusives_mean | 0,000 | 0,96 | large |
| Pathotype | amnestic_AD_vs_lvPPA_AD | syllable_count | 0,000 | 0,71 | large |
| Pathotype | amnestic_AD_vs_lvPPA_AD | voyelles_oral_count | 0,000 | 0,71 | large |
| Pathotype | FTLD_vs_healthy_control | pause_min_duration | 0,000 | 0,55 | large |
| Pathotype | FTLD_vs_healthy_control | inter_word_pause_min | 0,000 | 0,55 | large |
| Pathotype | lvPPA_FTLD_vs_amnestic_AD | semi_voyelles_max | 0,000 | 0,96 | large |
| Pathotype | healthy_control_vs_amnestic_AD | delta_mfcc_mean_12 | 0,000 | 0,48 | medium |
| Pathotype | svPPA_FTLD_vs_healthy_control | semi_voyelles_min | 0,000 | 0,75 | large |
| Pathotype | lvPPA_FTLD_vs_amnestic_AD | spectral_centroid_mean | 0,000 | 0,96 | large |
| Pathotype | FTLD_vs_healthy_control | chroma_mean_6 | 0,000 | 0,56 | large |
| Pathotype | amnestic_AD_vs_lvPPA_AD | chroma_mean_9 | 0,000 | 0,70 | large |
| Pathotype | lvPPA_FTLD_vs_healthy_control | fricatives_kurtosis | 0,000 | 0,88 | large |
| Pathotype | lvPPA_FTLD_vs_healthy_control | chroma_mean_8 | 0,000 | 0,88 | large |
| Pathotype | nfvPPA_FTLD_vs_amnestic_AD | chroma_mean_3 | 0,000 | 0,83 | large |
| Pathotype | healthy_control_vs_amnestic_AD | semi_voyelles_count | 0,000 | 0,46 | medium |
| Pathotype | nfvPPA_FTLD_vs_healthy_control | delta_mfcc_mean_8 | 0,000 | 0,75 | large |
| Pathotype | svPPA_FTLD_vs_amnestic_AD | chroma_mean_0 | 0,000 | 0,83 | large |
| Pathotype | nfvPPA_FTLD_vs_healthy_control | nasales_count | 0,000 | 0,73 | large |
| Pathotype | amnestic_AD_vs_lvPPA_AD | phoneme_count | 0,000 | 0,70 | large |
| Pathotype | amnestic_AD_vs_lvPPA_AD | num_speech_phonemes | 0,000 | 0,70 | large |
| Pathotype | FTLD_vs_lvPPA_FTLD | long_pause_count | 0,000 | 1,00 | large |
| Pathotype | svPPA_FTLD_vs_healthy_control | mean_phoneme_duration | 0,000 | 0,75 | large |
| Pathotype | lvPPA_FTLD_vs_amnestic_AD | spectral_flux | 0,000 | 0,96 | large |
| Pathotype | nfvPPA_FTLD_vs_amnestic_AD | liquides_percentile_10 | 0,000 | 0,81 | large |
| Pathotype | nfvPPA_FTLD_vs_FTLD | speech_rate_phonemes_per_sec | 0,000 | 0,89 | large |
| Pathotype | nfvPPA_FTLD_vs_FTLD | rate_speech_phonemes | 0,000 | 0,89 | large |
| Pathotype | svPPA_FTLD_vs_healthy_control | max_phoneme_duration | 0,000 | 0,75 | large |
| Pathotype | nfvPPA_FTLD_vs_healthy_control | occlusives_percentile_90 | 0,000 | 0,75 | large |
| Pathotype | svPPA_FTLD_vs_amnestic_AD | semi_voyelles_percentile_10 | 0,000 | 0,82 | large |
| Pathotype | FTLD_vs_healthy_control | pause_median_duration | 0,000 | 0,55 | large |
| Pathotype | FTLD_vs_healthy_control | inter_word_pause_median | 0,000 | 0,55 | large |
| Pathotype | svPPA_FTLD_vs_healthy_control | median_phoneme_duration | 0,000 | 0,72 | large |
| Pathotype | FTLD_vs_lvPPA_FTLD | occlusives_max | 0,000 | 1,00 | large |
| Pathotype | nfvPPA_FTLD_vs_amnestic_AD | semi_voyelles_count | 0,000 | 0,82 | large |
| Pathotype | FTLD_vs_lvPPA_FTLD | contrast_mean_4 | 0,000 | 1,00 | large |
| Pathotype | FTLD_vs_lvPPA_FTLD | num_inter_pauses | 0,000 | 1,00 | large |
| Pathotype | FTLD_vs_lvPPA_FTLD | occlusives_count | 0,000 | 1,00 | large |
| Pathotype | FTLD_vs_lvPPA_FTLD | occlusives_percentile_90 | 0,000 | 1,00 | large |
| Pathotype | nfvPPA_FTLD_vs_lvPPA_AD | delta_mfcc_mean_4 | 0,000 | 0,93 | large |
| Pathotype | FTLD_vs_lvPPA_FTLD | liquides_max | 0,000 | 1,00 | large |
| Pathotype | FTLD_vs_lvPPA_FTLD | nasales_std | 0,000 | 1,00 | large |
| Pathotype | FTLD_vs_lvPPA_FTLD | nasales_max | 0,000 | 1,00 | large |
| Pathotype | FTLD_vs_lvPPA_FTLD | nasales_percentile_90 | 0,000 | 1,00 | large |
| Pathotype | FTLD_vs_lvPPA_FTLD | hnr_mean | 0,000 | 1,00 | large |
| Pathotype | FTLD_vs_lvPPA_FTLD | delta_mfcc_mean_7 | 0,000 | 1,00 | large |
| Pathotype | FTLD_vs_lvPPA_FTLD | delta_mfcc_mean_9 | 0,000 | 1,00 | large |
| Pathotype | FTLD_vs_lvPPA_FTLD | rmse_mean | 0,000 | 1,00 | large |
| Pathotype | FTLD_vs_lvPPA_FTLD | intensity_mean | 0,000 | 1,00 | large |
| Pathotype | FTLD_vs_lvPPA_FTLD | nasales_cv | 0,000 | 1,00 | large |
| Pathotype | svPPA_FTLD_vs_healthy_control | f1_cv,1 | 0,000 | 0,75 | large |
| Pathotype | svPPA_FTLD_vs_lvPPA_AD | chroma_mean_3 | 0,000 | 0,93 | large |
| Pathotype | FTLD_vs_lvPPA_FTLD | nasales_iqr | 0,000 | 1,00 | large |
| Pathotype | FTLD_vs_lvPPA_FTLD | num_pauses | 0,000 | 1,00 | large |
| Pathotype | FTLD_vs_lvPPA_FTLD | num_inter_word_pauses | 0,000 | 1,00 | large |
| Pathotype | lvPPA_FTLD_vs_amnestic_AD | delta_mfcc_mean_8 | 0,000 | 0,95 | large |
| Pathotype | nfvPPA_FTLD_vs_amnestic_AD | delta_mfcc_mean_11 | 0,000 | 0,82 | large |
| Pathotype | healthy_control_vs_amnestic_AD | liquides_mean | 0,000 | 0,47 | medium |
| Pathotype | nfvPPA_FTLD_vs_FTLD | delta_mfcc_mean_11 | 0,000 | 0,88 | large |
| Pathotype | lvPPA_FTLD_vs_amnestic_AD | occlusives_min | 0,000 | 0,92 | large |
| Pathotype | healthy_control_vs_amnestic_AD | f3_cv,1 | 0,000 | 0,47 | medium |
| Pathotype | healthy_control_vs_lvPPA_AD | pause_frequency | 0,000 | 0,61 | large |
| Pathotype | svPPA_FTLD_vs_amnestic_AD | chroma_mean_10 | 0,000 | 0,82 | large |
| Pathotype | svPPA_FTLD_vs_amnestic_AD | pause_mean_duration | 0,000 | 0,82 | large |
| Pathotype | svPPA_FTLD_vs_amnestic_AD | inter_word_pause_mean | 0,000 | 0,82 | large |
| Pathotype | svPPA_FTLD_vs_healthy_control | kurt_phoneme_duration | 0,000 | 0,74 | large |
| Pathotype | nfvPPA_FTLD_vs_healthy_control | jitter_ppq5 | 0,000 | 0,74 | large |
| Pathotype | lvPPA_FTLD_vs_healthy_control | spectral_flux_std | 0,000 | 0,87 | large |
| Pathotype | healthy_control_vs_lvPPA_AD | chroma_mean_11 | 0,000 | 0,61 | large |
| Pathotype | FTLD_vs_lvPPA_AD | contrast_mean_4 | 0,000 | 0,75 | large |
| Pathotype | svPPA_FTLD_vs_healthy_control | skew_phoneme_duration | 0,000 | 0,74 | large |
| Pathotype | nfvPPA_FTLD_vs_healthy_control | jitter_local | 0,000 | 0,74 | large |
| Pathotype | healthy_control_vs_lvPPA_AD | fricatives_max | 0,000 | 0,61 | large |
| Pathotype | nfvPPA_FTLD_vs_amnestic_AD | delta_mfcc_mean_4 | 0,000 | 0,81 | large |
| Pathotype | nfvPPA_FTLD_vs_amnestic_AD | shimmer_dda | 0,000 | 0,81 | large |
| Pathotype | nfvPPA_FTLD_vs_healthy_control | f3_cv | 0,000 | 0,74 | large |
| Pathotype | healthy_control_vs_amnestic_AD | spectral_flux_std | 0,000 | 0,47 | medium |
| Pathotype | lvPPA_FTLD_vs_amnestic_AD | contrast_mean_2 | 0,000 | 0,94 | large |
| Pathotype | lvPPA_FTLD_vs_amnestic_AD | intensity_slope | 0,000 | 0,94 | large |
| Pathotype | nfvPPA_FTLD_vs_amnestic_AD | liquides_median | 0,000 | 0,81 | large |
| Pathotype | nfvPPA_FTLD_vs_FTLD | chroma_mean_2 | 0,000 | 0,87 | large |
| Pathotype | nfvPPA_FTLD_vs_healthy_control | f0_slope | 0,000 | 0,73 | large |
| Pathotype | FTLD_vs_healthy_control | semi_voyelles_cv | 0,000 | 0,54 | large |
| Pathotype | FTLD_vs_lvPPA_FTLD | nasales_count | 0,000 | 0,99 | large |
| Pathotype | FTLD_vs_lvPPA_FTLD | delta_mfcc_mean_8 | 0,000 | 0,99 | large |
| Pathotype | FTLD_vs_lvPPA_FTLD | delta_mfcc_mean_12 | 0,000 | 0,99 | large |
| Pathotype | nfvPPA_FTLD_vs_healthy_control | delta_mfcc_mean_11 | 0,000 | 0,73 | large |
| Pathotype | amnestic_AD_vs_lvPPA_AD | chroma_mean_0 | 0,000 | 0,68 | large |
| Pathotype | FTLD_vs_lvPPA_AD | long_pause_count | 0,000 | 0,74 | large |
| Pathotype | lvPPA_FTLD_vs_amnestic_AD | semi_voyelles_mean | 0,000 | 0,93 | large |
| Pathotype | svPPA_FTLD_vs_amnestic_AD | intensity_dynamic_range | 0,000 | 0,81 | large |
| Pathotype | svPPA_FTLD_vs_healthy_control | spectral_centroid_slope | 0,000 | 0,73 | large |
| Pathotype | svPPA_FTLD_vs_healthy_control | intensity_std | 0,000 | 0,73 | large |
| Pathotype | svPPA_FTLD_vs_amnestic_AD | spectral_instability | 0,000 | 0,81 | large |
| Pathotype | svPPA_FTLD_vs_FTLD | occlusives_median | 0,000 | 0,85 | large |
| Pathotype | lvPPA_FTLD_vs_amnestic_AD | f0_min | 0,000 | 0,82 | large |
| Pathotype | nfvPPA_FTLD_vs_healthy_control | hnr_mean,1 | 0,000 | 0,73 | large |
| Pathotype | nfvPPA_FTLD_vs_healthy_control | occlusives_median | 0,000 | 0,71 | large |
| Pathotype | FTLD_vs_healthy_control | contrast_mean_1 | 0,000 | 0,54 | large |
| Pathotype | FTLD_vs_lvPPA_FTLD | liquides_count | 0,000 | 0,97 | large |
| Pathotype | nfvPPA_FTLD_vs_healthy_control | f3_cv,1 | 0,000 | 0,73 | large |
| Pathotype | FTLD_vs_lvPPA_FTLD | delta_mfcc_mean_1 | 0,000 | 0,97 | large |
| Pathotype | svPPA_FTLD_vs_amnestic_AD | liquides_median | 0,000 | 0,80 | large |
| Pathotype | FTLD_vs_healthy_control | contrast_mean_4 | 0,000 | 0,54 | large |
| Pathotype | svPPA_FTLD_vs_amnestic_AD | pause_max_duration | 0,000 | 0,80 | large |
| Pathotype | svPPA_FTLD_vs_amnestic_AD | inter_word_pause_max | 0,000 | 0,80 | large |
| Pathotype | FTLD_vs_lvPPA_FTLD | liquides_iqr | 0,000 | 0,97 | large |
| Pathotype | svPPA_FTLD_vs_lvPPA_AD | delta_mfcc_mean_5 | 0,000 | 0,90 | large |
| Pathotype | svPPA_FTLD_vs_lvPPA_AD | spectral_flux_mean | 0,000 | 0,90 | large |
| Pathotype | FTLD_vs_lvPPA_FTLD | spectral_flux_mean | 0,000 | 0,97 | large |
| Pathotype | FTLD_vs_lvPPA_FTLD | liquides_std | 0,000 | 0,97 | large |
| Pathotype | nfvPPA_FTLD_vs_lvPPA_AD | chroma_mean_1 | 0,000 | 0,90 | large |
| Pathotype | nfvPPA_FTLD_vs_lvPPA_AD | chroma_mean_3 | 0,000 | 0,90 | large |
| Pathotype | nfvPPA_FTLD_vs_lvPPA_AD | speech_rate_phonemes_per_sec | 0,000 | 0,90 | large |
| Pathotype | nfvPPA_FTLD_vs_lvPPA_AD | rate_speech_phonemes | 0,000 | 0,90 | large |
| Pathotype | FTLD_vs_lvPPA_FTLD | phoneme_count | 0,000 | 0,97 | large |
| Pathotype | FTLD_vs_lvPPA_FTLD | syllable_count | 0,000 | 0,97 | large |
| Pathotype | FTLD_vs_lvPPA_FTLD | num_speech_phonemes | 0,000 | 0,97 | large |
| Pathotype | FTLD_vs_lvPPA_FTLD | voyelles_oral_count | 0,000 | 0,97 | large |
| Pathotype | FTLD_vs_lvPPA_FTLD | liquides_cv | 0,000 | 0,97 | large |
| Pathotype | FTLD_vs_lvPPA_FTLD | liquides_percentile_90 | 0,000 | 0,97 | large |
| Pathotype | healthy_control_vs_amnestic_AD | nasales_max | 0,000 | 0,46 | medium |
| Pathotype | lvPPA_FTLD_vs_amnestic_AD | delta_mfcc_mean_4 | 0,000 | 0,92 | large |
| Pathotype | FTLD_vs_lvPPA_FTLD | speech_duration | 0,000 | 0,97 | large |
| Pathotype | healthy_control_vs_lvPPA_AD | chroma_mean_3 | 0,000 | 0,59 | large |
| Pathotype | FTLD_vs_healthy_control | pause_ratio | 0,000 | 0,53 | large |
| Pathotype | nfvPPA_FTLD_vs_amnestic_AD | semi_voyelles_iqr | 0,000 | 0,79 | large |
| Pathotype | svPPA_FTLD_vs_nfvPPA_FTLD | jitter_local | 0,000 | 1,00 | large |
| Pathotype | svPPA_FTLD_vs_nfvPPA_FTLD | jitter_rap | 0,000 | 1,00 | large |
| Pathotype | svPPA_FTLD_vs_nfvPPA_FTLD | jitter_ppq5 | 0,000 | 1,00 | large |
| Pathotype | svPPA_FTLD_vs_nfvPPA_FTLD | shimmer_local | 0,000 | 1,00 | large |
| Pathotype | svPPA_FTLD_vs_nfvPPA_FTLD | shimmer_apq3 | 0,000 | 1,00 | large |
| Pathotype | svPPA_FTLD_vs_nfvPPA_FTLD | shimmer_apq5 | 0,000 | 1,00 | large |
| Pathotype | svPPA_FTLD_vs_nfvPPA_FTLD | shimmer_dda | 0,000 | 1,00 | large |
| Pathotype | svPPA_FTLD_vs_nfvPPA_FTLD | delta_mfcc_mean_5 | 0,000 | 1,00 | large |
| Pathotype | lvPPA_FTLD_vs_healthy_control | intensity_dynamic_range | 0,000 | 0,84 | large |
| Pathotype | nfvPPA_FTLD_vs_amnestic_AD | spectral_flux | 0,000 | 0,79 | large |
| Pathotype | nfvPPA_FTLD_vs_healthy_control | mean_phoneme_duration | 0,000 | 0,72 | large |
| Pathotype | svPPA_FTLD_vs_nfvPPA_FTLD | speech_rate_phonemes_per_sec | 0,000 | 1,00 | large |
| Pathotype | svPPA_FTLD_vs_nfvPPA_FTLD | rate_speech_phonemes | 0,000 | 1,00 | large |
| Pathotype | svPPA_FTLD_vs_nfvPPA_FTLD | syllable_rate_per_sec | 0,000 | 1,00 | large |
| Pathotype | svPPA_FTLD_vs_nfvPPA_FTLD | nasales_kurtosis | 0,000 | 1,00 | large |
| Pathotype | svPPA_FTLD_vs_FTLD | shimmer_apq3 | 0,000 | 0,85 | large |
| Pathotype | svPPA_FTLD_vs_FTLD | shimmer_dda | 0,000 | 0,85 | large |
| Pathotype | nfvPPA_FTLD_vs_FTLD | shimmer_apq3 | 0,000 | 0,85 | large |
| Pathotype | nfvPPA_FTLD_vs_FTLD | shimmer_dda | 0,000 | 0,85 | large |
| Pathotype | lvPPA_FTLD_vs_amnestic_AD | spectral_centroid_slope | 0,000 | 0,91 | large |
| Pathotype | amnestic_AD_vs_lvPPA_AD | speech_duration | 0,000 | 0,67 | large |
| Pathotype | lvPPA_FTLD_vs_lvPPA_AD | long_pause_count | 0,000 | 1,00 | large |
| Pathotype | FTLD_vs_healthy_control | fricatives_iqr | 0,000 | 0,53 | large |
| Pathotype | amnestic_AD_vs_lvPPA_AD | chroma_mean_10 | 0,000 | 0,67 | large |
| Pathotype | amnestic_AD_vs_lvPPA_AD | contrast_mean_2 | 0,000 | 0,67 | large |
| Pathotype | lvPPA_FTLD_vs_amnestic_AD | fricatives_max | 0,000 | 0,91 | large |
| Pathotype | nfvPPA_FTLD_vs_amnestic_AD | jitter_ppq5 | 0,000 | 0,79 | large |
| Pathotype | lvPPA_FTLD_vs_healthy_control | semi_voyelles_skewness | 0,000 | 0,84 | large |
| Pathotype | FTLD_vs_healthy_control | semi_voyelles_max | 0,000 | 0,53 | large |
| Pathotype | lvPPA_FTLD_vs_amnestic_AD | contrast_mean_0 | 0,000 | 0,91 | large |
| Pathotype | amnestic_AD_vs_lvPPA_AD | occlusives_count | 0,000 | 0,66 | large |
| Pathotype | FTLD_vs_lvPPA_FTLD | occlusives_iqr | 0,000 | 0,96 | large |
| Pathotype | lvPPA_FTLD_vs_lvPPA_AD | num_inter_pauses | 0,000 | 1,00 | large |
| Pathotype | lvPPA_FTLD_vs_lvPPA_AD | liquides_std | 0,000 | 1,00 | large |
| Pathotype | lvPPA_FTLD_vs_lvPPA_AD | liquides_cv | 0,000 | 1,00 | large |
| Pathotype | lvPPA_FTLD_vs_lvPPA_AD | liquides_iqr | 0,000 | 1,00 | large |
| Pathotype | nfvPPA_FTLD_vs_amnestic_AD | nasales_count | 0,000 | 0,79 | large |
| Pathotype | nfvPPA_FTLD_vs_lvPPA_AD | chroma_mean_8 | 0,000 | 0,89 | large |
| Pathotype | svPPA_FTLD_vs_amnestic_AD | shimmer_apq3 | 0,000 | 0,79 | large |
| Pathotype | lvPPA_FTLD_vs_lvPPA_AD | liquides_max | 0,000 | 1,00 | large |
| Pathotype | lvPPA_FTLD_vs_lvPPA_AD | hnr_mean | 0,000 | 1,00 | large |
| Pathotype | lvPPA_FTLD_vs_lvPPA_AD | f0_mean | 0,000 | 1,00 | large |
| Pathotype | lvPPA_FTLD_vs_lvPPA_AD | hnr_mean,1 | 0,000 | 1,00 | large |
| Pathotype | lvPPA_FTLD_vs_lvPPA_AD | num_pauses | 0,000 | 1,00 | large |
| Pathotype | lvPPA_FTLD_vs_lvPPA_AD | num_inter_word_pauses | 0,000 | 1,00 | large |
| Pathotype | healthy_control_vs_amnestic_AD | fricatives_count | 0,000 | 0,45 | medium |
| Pathotype | lvPPA_FTLD_vs_healthy_control | occlusives_kurtosis | 0,000 | 0,84 | large |
| Pathotype | lvPPA_FTLD_vs_lvPPA_AD | phoneme_count | 0,000 | 1,00 | large |
| Pathotype | lvPPA_FTLD_vs_lvPPA_AD | num_speech_phonemes | 0,000 | 1,00 | large |
| Pathotype | lvPPA_FTLD_vs_lvPPA_AD | liquides_mean | 0,000 | 1,00 | large |
| Pathotype | lvPPA_FTLD_vs_lvPPA_AD | liquides_percentile_90 | 0,000 | 1,00 | large |
| Pathotype | lvPPA_FTLD_vs_healthy_control | intensity_std | 0,000 | 0,84 | large |
| Pathotype | healthy_control_vs_lvPPA_AD | fricatives_skewness | 0,000 | 0,59 | large |
| Pathotype | svPPA_FTLD_vs_healthy_control | semi_voyelles_iqr | 0,000 | 0,71 | large |
| Pathotype | nfvPPA_FTLD_vs_healthy_control | liquides_mean | 0,000 | 0,71 | large |
| Pathotype | nfvPPA_FTLD_vs_FTLD | shimmer_local | 0,000 | 0,84 | large |
| Pathotype | nfvPPA_FTLD_vs_FTLD | shimmer_apq5 | 0,000 | 0,84 | large |
| Pathotype | FTLD_vs_healthy_control | hnr_std,1 | 0,000 | 0,52 | large |
| Pathotype | lvPPA_FTLD_vs_amnestic_AD | liquides_std | 0,000 | 0,90 | large |
| Pathotype | lvPPA_FTLD_vs_amnestic_AD | liquides_cv | 0,000 | 0,90 | large |
| Pathotype | lvPPA_FTLD_vs_amnestic_AD | liquides_iqr | 0,000 | 0,90 | large |
| Pathotype | amnestic_AD_vs_lvPPA_AD | pause_proportion | 0,000 | 0,66 | large |
| Pathotype | svPPA_FTLD_vs_amnestic_AD | pause_median_duration | 0,000 | 0,78 | large |
| Pathotype | svPPA_FTLD_vs_amnestic_AD | inter_word_pause_median | 0,000 | 0,78 | large |
| Pathotype | nfvPPA_FTLD_vs_healthy_control | liquides_iqr | 0,000 | 0,71 | large |
| Pathotype | healthy_control_vs_amnestic_AD | nasales_percentile_90 | 0,000 | 0,45 | medium |
| Pathotype | lvPPA_FTLD_vs_lvPPA_AD | occlusives_count | 0,000 | 0,99 | large |
| Pathotype | svPPA_FTLD_vs_healthy_control | semi_voyelles_kurtosis | 0,000 | 0,70 | large |
| Pathotype | amnestic_AD_vs_lvPPA_AD | pause_total_duration | 0,000 | 0,66 | large |
| Pathotype | svPPA_FTLD_vs_lvPPA_AD | f0_mean | 0,000 | 0,88 | large |
| Pathotype | nfvPPA_FTLD_vs_lvPPA_AD | chroma_mean_0 | 0,000 | 0,88 | large |
| Pathotype | FTLD_vs_lvPPA_FTLD | spectral_flux | 0,000 | 0,95 | large |
| Pathotype | FTLD_vs_lvPPA_FTLD | total_duration | 0,000 | 0,95 | large |
| Pathotype | FTLD_vs_lvPPA_AD | chroma_mean_3 | 0,000 | 0,71 | large |
| Pathotype | svPPA_FTLD_vs_healthy_control | voyelles_oral_skewness | 0,000 | 0,70 | large |
| Pathotype | nfvPPA_FTLD_vs_amnestic_AD | occlusives_mean | 0,000 | 0,78 | large |
| Pathotype | svPPA_FTLD_vs_FTLD | chroma_mean_2 | 0,000 | 0,83 | large |
| Pathotype | healthy_control_vs_amnestic_AD | fricatives_std | 0,000 | 0,45 | medium |
| Pathotype | svPPA_FTLD_vs_FTLD | shimmer_local | 0,000 | 0,83 | large |
| Pathotype | svPPA_FTLD_vs_FTLD | delta_mfcc_mean_5 | 0,000 | 0,83 | large |
| Pathotype | lvPPA_FTLD_vs_amnestic_AD | liquides_skewness | 0,000 | 0,89 | large |
| Pathotype | lvPPA_FTLD_vs_lvPPA_AD | fricatives_count | 0,000 | 0,98 | large |
| Pathotype | nfvPPA_FTLD_vs_amnestic_AD | delta_mfcc_mean_8 | 0,000 | 0,78 | large |
| Pathotype | healthy_control_vs_amnestic_AD | occlusives_mean | 0,000 | 0,45 | medium |
| Pathotype | lvPPA_FTLD_vs_lvPPA_AD | syllable_count | 0,000 | 0,98 | large |
| Pathotype | lvPPA_FTLD_vs_lvPPA_AD | voyelles_oral_count | 0,000 | 0,98 | large |
| Pathotype | lvPPA_FTLD_vs_amnestic_AD | contrast_mean_3 | 0,000 | 0,89 | large |
| Pathotype | svPPA_FTLD_vs_healthy_control | zcr_mean | 0,000 | 0,70 | large |
| Pathotype | lvPPA_FTLD_vs_lvPPA_AD | spectral_flux | 0,000 | 0,98 | large |
| Pathotype | lvPPA_FTLD_vs_lvPPA_AD | delta_mfcc_mean_1 | 0,000 | 0,98 | large |
| Pathotype | lvPPA_FTLD_vs_lvPPA_AD | delta_mfcc_mean_12 | 0,000 | 0,98 | large |
| Pathotype | lvPPA_FTLD_vs_lvPPA_AD | contrast_mean_4 | 0,000 | 0,98 | large |
| Pathotype | lvPPA_FTLD_vs_lvPPA_AD | spectral_flux_mean | 0,000 | 0,98 | large |
| Pathotype | lvPPA_FTLD_vs_lvPPA_AD | intensity_slope | 0,000 | 0,98 | large |
| Pathotype | lvPPA_FTLD_vs_lvPPA_AD | total_duration | 0,000 | 0,98 | large |
| Pathotype | lvPPA_FTLD_vs_lvPPA_AD | speech_duration | 0,000 | 0,98 | large |
| Pathotype | lvPPA_FTLD_vs_lvPPA_AD | pause_total_duration | 0,000 | 0,98 | large |
| Pathotype | healthy_control_vs_amnestic_AD | delta_mfcc_mean_3 | 0,000 | 0,44 | medium |
| Pathotype | nfvPPA_FTLD_vs_healthy_control | jitter_rap | 0,000 | 0,70 | large |
| Pathotype | lvPPA_FTLD_vs_amnestic_AD | semi_voyelles_median | 0,000 | 0,89 | large |
| Pathotype | FTLD_vs_amnestic_AD | contrast_mean_3 | 0,000 | 0,60 | large |
| Pathotype | healthy_control_vs_lvPPA_AD | delta_mfcc_mean_1 | 0,000 | 0,57 | large |
| Pathotype | healthy_control_vs_lvPPA_AD | occlusives_percentile_90 | 0,000 | 0,57 | large |
| Pathotype | nfvPPA_FTLD_vs_amnestic_AD | semi_voyelles_max | 0,000 | 0,77 | large |
| Pathotype | healthy_control_vs_amnestic_AD | spectral_instability,1 | 0,000 | 0,44 | medium |
| Pathotype | lvPPA_FTLD_vs_healthy_control | hnr_mean | 0,000 | 0,82 | large |
| Pathotype | lvPPA_FTLD_vs_amnestic_AD | chroma_mean_9 | 0,000 | 0,88 | large |
| Pathotype | amnestic_AD_vs_lvPPA_AD | fricatives_count | 0,000 | 0,65 | large |
| Pathotype | healthy_control_vs_amnestic_AD | pause_total_duration | 0,000 | 0,44 | medium |
| Pathotype | FTLD_vs_healthy_control | pause_proportion | 0,000 | 0,51 | large |
| Pathotype | amnestic_AD_vs_lvPPA_AD | chroma_mean_1 | 0,000 | 0,65 | large |
| Pathotype | amnestic_AD_vs_lvPPA_AD | spectral_flux_std | 0,000 | 0,65 | large |
| Pathotype | healthy_control_vs_lvPPA_AD | spectral_instability,1 | 0,000 | 0,57 | large |
| Pathotype | lvPPA_FTLD_vs_amnestic_AD | spectral_instability,1 | 0,000 | 0,88 | large |
| Pathotype | FTLD_vs_lvPPA_FTLD | rolloff_mean | 0,000 | 0,93 | large |
| Pathotype | healthy_control_vs_lvPPA_AD | num_inter_pauses | 0,000 | 0,57 | large |
| Pathotype | svPPA_FTLD_vs_FTLD | chroma_mean_0 | 0,000 | 0,82 | large |
| Pathotype | FTLD_vs_lvPPA_FTLD | chroma_mean_8 | 0,000 | 0,93 | large |
| Pathotype | nfvPPA_FTLD_vs_lvPPA_AD | chroma_mean_10 | 0,000 | 0,86 | large |
| Pathotype | svPPA_FTLD_vs_FTLD | voyelles_oral_mean | 0,000 | 0,82 | large |
| Pathotype | FTLD_vs_lvPPA_FTLD | nasales_mean | 0,000 | 0,93 | large |
| Pathotype | svPPA_FTLD_vs_healthy_control | chroma_mean_1 | 0,000 | 0,69 | large |
| Pathotype | svPPA_FTLD_vs_healthy_control | std_phoneme_duration | 0,000 | 0,69 | large |
| Pathotype | svPPA_FTLD_vs_amnestic_AD | semi_voyelles_median | 0,000 | 0,76 | large |
| Pathotype | FTLD_vs_healthy_control | nasales_percentile_10 | 0,000 | 0,51 | large |
| Pathotype | lvPPA_FTLD_vs_lvPPA_AD | liquides_count | 0,000 | 0,96 | large |
| Pathotype | lvPPA_FTLD_vs_amnestic_AD | occlusives_median | 0,000 | 0,88 | large |
| Pathotype | amnestic_AD_vs_lvPPA_AD | pause_ratio | 0,000 | 0,64 | large |
| Pathotype | FTLD_vs_healthy_control | chroma_mean_5 | 0,000 | 0,51 | large |
| Pathotype | svPPA_FTLD_vs_nfvPPA_FTLD | liquides_skewness | 0,000 | 0,96 | large |
| Pathotype | FTLD_vs_amnestic_AD | hnr_mean | 0,000 | 0,59 | large |
| Pathotype | lvPPA_FTLD_vs_amnestic_AD | liquides_count | 0,000 | 0,88 | large |
| Pathotype | svPPA_FTLD_vs_healthy_control | cv_phoneme_duration | 0,000 | 0,69 | large |
| Pathotype | lvPPA_FTLD_vs_amnestic_AD | nasales_skewness | 0,000 | 0,88 | large |
| Pathotype | nfvPPA_FTLD_vs_healthy_control | liquides_percentile_10 | 0,000 | 0,68 | large |
| Pathotype | lvPPA_FTLD_vs_lvPPA_AD | jitter_ppq5 | 0,000 | 0,96 | large |
| Pathotype | nfvPPA_FTLD_vs_healthy_control | occlusives_percentile_10 | 0,000 | 0,68 | large |
| Pathotype | lvPPA_FTLD_vs_amnestic_AD | occlusives_kurtosis | 0,000 | 0,88 | large |
| Pathotype | FTLD_vs_amnestic_AD | nasales_count | 0,000 | 0,58 | large |
| Pathotype | FTLD_vs_lvPPA_AD | f0_std | 0,000 | 0,70 | large |
| Pathotype | svPPA_FTLD_vs_healthy_control | voyelles_oral_percentile_90 | 0,000 | 0,68 | large |
| Pathotype | FTLD_vs_lvPPA_AD | rolloff_std | 0,000 | 0,70 | large |
| Pathotype | healthy_control_vs_amnestic_AD | semi_voyelles_iqr | 0,000 | 0,43 | medium |
| Pathotype | healthy_control_vs_amnestic_AD | jitter_ppq5 | 0,000 | 0,43 | medium |
| Pathotype | svPPA_FTLD_vs_amnestic_AD | chroma_mean_1 | 0,000 | 0,76 | large |
| Pathotype | svPPA_FTLD_vs_amnestic_AD | pause_std_duration | 0,000 | 0,76 | large |
| Pathotype | svPPA_FTLD_vs_amnestic_AD | inter_word_pause_std | 0,000 | 0,76 | large |
| Pathotype | svPPA_FTLD_vs_healthy_control | chroma_mean_5 | 0,000 | 0,68 | large |
| Pathotype | svPPA_FTLD_vs_healthy_control | voyelles_oral_kurtosis | 0,000 | 0,68 | large |
| Pathotype | svPPA_FTLD_vs_lvPPA_AD | jitter_ppq5 | 0,000 | 0,85 | large |
| Pathotype | healthy_control_vs_amnestic_AD | long_pause_count | 0,000 | 0,43 | medium |
| Pathotype | FTLD_vs_lvPPA_FTLD | nasales_skewness | 0,000 | 0,90 | large |
| Pathotype | FTLD_vs_lvPPA_FTLD | nasales_median | 0,000 | 0,92 | large |
| Pathotype | FTLD_vs_lvPPA_FTLD | delta_mfcc_mean_4 | 0,000 | 0,92 | large |
| Pathotype | lvPPA_FTLD_vs_healthy_control | spectral_instability | 0,000 | 0,80 | large |
| Pathotype | lvPPA_FTLD_vs_healthy_control | delta_mfcc_mean_9 | 0,000 | 0,80 | large |
| Pathotype | lvPPA_FTLD_vs_healthy_control | spectral_centroid_mean | 0,000 | 0,80 | large |
| Pathotype | lvPPA_FTLD_vs_amnestic_AD | liquides_max | 0,000 | 0,87 | large |
| Pathotype | lvPPA_FTLD_vs_lvPPA_AD | nasales_skewness | 0,000 | 0,94 | large |
| Pathotype | nfvPPA_FTLD_vs_amnestic_AD | occlusives_percentile_90 | 0,000 | 0,75 | large |
| Pathotype | svPPA_FTLD_vs_amnestic_AD | voyelles_oral_median | 0,000 | 0,75 | large |
| Pathotype | nfvPPA_FTLD_vs_amnestic_AD | semi_voyelles_mean | 0,000 | 0,75 | large |
| Pathotype | nfvPPA_FTLD_vs_healthy_control | liquides_percentile_90 | 0,000 | 0,68 | large |
| Pathotype | healthy_control_vs_amnestic_AD | pause_ratio | 0,000 | 0,43 | medium |
| Pathotype | lvPPA_FTLD_vs_amnestic_AD | liquides_median | 0,000 | 0,87 | large |
| Pathotype | lvPPA_FTLD_vs_amnestic_AD | liquides_mean | 0,000 | 0,87 | large |
| Pathotype | lvPPA_FTLD_vs_amnestic_AD | liquides_percentile_90 | 0,000 | 0,87 | large |
| Pathotype | amnestic_AD_vs_lvPPA_AD | delta_mfcc_mean_10 | 0,000 | 0,63 | large |
| Pathotype | amnestic_AD_vs_lvPPA_AD | pause_mean_duration | 0,000 | 0,63 | large |
| Pathotype | amnestic_AD_vs_lvPPA_AD | inter_word_pause_mean | 0,000 | 0,63 | large |
| Pathotype | nfvPPA_FTLD_vs_amnestic_AD | liquides_skewness | 0,000 | 0,75 | large |
| Pathotype | nfvPPA_FTLD_vs_amnestic_AD | chroma_mean_2 | 0,000 | 0,75 | large |
| Pathotype | svPPA_FTLD_vs_amnestic_AD | occlusives_percentile_10 | 0,000 | 0,69 | large |
| Pathotype | nfvPPA_FTLD_vs_healthy_control | semi_voyelles_mean | 0,000 | 0,67 | large |
| Pathotype | healthy_control_vs_lvPPA_AD | num_pauses | 0,000 | 0,55 | large |
| Pathotype | healthy_control_vs_lvPPA_AD | num_inter_word_pauses | 0,000 | 0,55 | large |
| Pathotype | svPPA_FTLD_vs_FTLD | voyelles_oral_max | 0,000 | 0,80 | large |
| Pathotype | svPPA_FTLD_vs_FTLD | voyelles_oral_std | 0,000 | 0,80 | large |
| Pathotype | nfvPPA_FTLD_vs_FTLD | occlusives_mean | 0,000 | 0,80 | large |
| Pathotype | lvPPA_FTLD_vs_amnestic_AD | median_phoneme_duration | 0,000 | 0,83 | large |
| Pathotype | FTLD_vs_healthy_control | semi_voyelles_min | 0,000 | 0,49 | medium |
| Pathotype | svPPA_FTLD_vs_healthy_control | f1_cv | 0,000 | 0,68 | large |
| Pathotype | healthy_control_vs_lvPPA_AD | median_phoneme_duration | 0,000 | 0,53 | large |
| Pathotype | healthy_control_vs_amnestic_AD | contrast_mean_4 | 0,000 | 0,43 | medium |
| Pathotype | FTLD_vs_healthy_control | zcr_mean | 0,000 | 0,50 | medium |
| Pathotype | healthy_control_vs_amnestic_AD | pause_proportion | 0,000 | 0,43 | medium |
| Pathotype | nfvPPA_FTLD_vs_amnestic_AD | semi_voyelles_percentile_90 | 0,000 | 0,74 | large |
| Pathotype | lvPPA_FTLD_vs_amnestic_AD | chroma_mean_5 | 0,000 | 0,86 | large |
| Pathotype | lvPPA_FTLD_vs_lvPPA_AD | jitter_local | 0,000 | 0,95 | large |
| Pathotype | lvPPA_FTLD_vs_lvPPA_AD | chroma_mean_8 | 0,000 | 0,95 | large |
| Pathotype | lvPPA_FTLD_vs_lvPPA_AD | rolloff_mean | 0,000 | 0,95 | large |
| Pathotype | lvPPA_FTLD_vs_lvPPA_AD | spectral_instability,1 | 0,000 | 0,95 | large |
| Pathotype | healthy_control_vs_lvPPA_AD | f0_std | 0,000 | 0,55 | large |
| Pathotype | FTLD_vs_healthy_control | contrast_mean_3 | 0,000 | 0,50 | medium |
| Pathotype | FTLD_vs_lvPPA_AD | chroma_mean_8 | 0,000 | 0,68 | large |
| Pathotype | FTLD_vs_lvPPA_AD | delta_mfcc_mean_9 | 0,000 | 0,68 | large |
| Pathotype | FTLD_vs_lvPPA_FTLD | contrast_mean_0 | 0,000 | 0,90 | large |
| Pathotype | FTLD_vs_lvPPA_FTLD | contrast_mean_2 | 0,000 | 0,90 | large |
| Pathotype | lvPPA_FTLD_vs_healthy_control | voyelles_oral_median | 0,000 | 0,76 | large |
| Pathotype | nfvPPA_FTLD_vs_amnestic_AD | jitter_rap | 0,000 | 0,74 | large |
| Pathotype | lvPPA_FTLD_vs_healthy_control | articulation_rate | 0,000 | 0,79 | large |
| Pathotype | lvPPA_FTLD_vs_amnestic_AD | occlusives_std | 0,000 | 0,86 | large |
| Pathotype | FTLD_vs_healthy_control | nasales_std | 0,000 | 0,50 | medium |
| Pathotype | FTLD_vs_healthy_control | hnr_mean,1 | 0,000 | 0,50 | medium |
| Pathotype | FTLD_vs_lvPPA_FTLD | chroma_mean_5 | 0,000 | 0,90 | large |
| Pathotype | FTLD_vs_lvPPA_FTLD | intensity_slope | 0,000 | 0,90 | large |
| Pathotype | nfvPPA_FTLD_vs_amnestic_AD | speech_rate_phonemes_per_sec | 0,000 | 0,74 | large |
| Pathotype | nfvPPA_FTLD_vs_amnestic_AD | rate_speech_phonemes | 0,000 | 0,74 | large |
| Pathotype | lvPPA_FTLD_vs_healthy_control | nasales_mean | 0,000 | 0,79 | large |
| Pathotype | nfvPPA_FTLD_vs_healthy_control | rolloff_mean | 0,000 | 0,67 | large |
| Pathotype | FTLD_vs_lvPPA_FTLD | occlusives_min | 0,000 | 0,88 | large |
| Pathotype | healthy_control_vs_amnestic_AD | syllable_count | 0,000 | 0,43 | medium |
| Pathotype | healthy_control_vs_amnestic_AD | voyelles_oral_count | 0,000 | 0,43 | medium |
| Pathotype | FTLD_vs_healthy_control | chroma_mean_8 | 0,000 | 0,50 | medium |
| Pathotype | FTLD_vs_healthy_control | semi_voyelles_percentile_90 | 0,000 | 0,50 | medium |
| Pathotype | healthy_control_vs_lvPPA_AD | voyelles_oral_median | 0,000 | 0,54 | large |
| Pathotype | amnestic_AD_vs_lvPPA_AD | contrast_mean_4 | 0,000 | 0,63 | large |
| Pathotype | svPPA_FTLD_vs_FTLD | chroma_mean_1 | 0,000 | 0,79 | large |
| Pathotype | svPPA_FTLD_vs_FTLD | chroma_mean_11 | 0,000 | 0,79 | large |
| Pathotype | svPPA_FTLD_vs_lvPPA_FTLD | nasales_skewness | 0,000 | 1,00 | large |
| Pathotype | svPPA_FTLD_vs_lvPPA_FTLD | nasales_kurtosis | 0,000 | 1,00 | large |
| Pathotype | svPPA_FTLD_vs_amnestic_AD | chroma_mean_3 | 0,000 | 0,74 | large |
| Pathotype | healthy_control_vs_lvPPA_AD | delta_mfcc_mean_10 | 0,000 | 0,55 | large |
| Pathotype | FTLD_vs_lvPPA_AD | pause_total_duration | 0,000 | 0,68 | large |
| Pathotype | svPPA_FTLD_vs_healthy_control | fricatives_std | 0,000 | 0,67 | large |
| Pathotype | lvPPA_FTLD_vs_healthy_control | contrast_mean_3 | 0,000 | 0,78 | large |
| Pathotype | nfvPPA_FTLD_vs_amnestic_AD | liquides_mean | 0,001 | 0,74 | large |
| Pathotype | lvPPA_FTLD_vs_amnestic_AD | max_phoneme_duration | 0,001 | 0,85 | large |
| Pathotype | FTLD_vs_healthy_control | liquides_percentile_90 | 0,001 | 0,49 | medium |
| Pathotype | lvPPA_FTLD_vs_amnestic_AD | nasales_mean | 0,001 | 0,85 | large |
| Pathotype | FTLD_vs_lvPPA_FTLD | spectral_centroid_mean | 0,001 | 0,89 | large |
| Pathotype | FTLD_vs_lvPPA_FTLD | semi_voyelles_max | 0,001 | 0,89 | large |
| Pathotype | FTLD_vs_healthy_control | pause_mean_duration | 0,001 | 0,49 | medium |
| Pathotype | FTLD_vs_healthy_control | inter_word_pause_mean | 0,001 | 0,49 | medium |
| Pathotype | FTLD_vs_amnestic_AD | delta_mfcc_mean_1 | 0,001 | 0,57 | large |
| Pathotype | svPPA_FTLD_vs_lvPPA_AD | spectral_centroid_slope | 0,001 | 0,83 | large |
| Pathotype | svPPA_FTLD_vs_lvPPA_AD | f0_max | 0,001 | 0,83 | large |
| Pathotype | svPPA_FTLD_vs_lvPPA_AD | f0_slope | 0,001 | 0,83 | large |
| Pathotype | svPPA_FTLD_vs_amnestic_AD | chroma_mean_5 | 0,001 | 0,73 | large |
| Pathotype | svPPA_FTLD_vs_amnestic_AD | spectral_centroid_slope | 0,001 | 0,73 | large |
| Pathotype | nfvPPA_FTLD_vs_lvPPA_AD | pause_proportion | 0,001 | 0,83 | large |
| Pathotype | nfvPPA_FTLD_vs_lvPPA_AD | pause_ratio | 0,001 | 0,83 | large |
| Pathotype | FTLD_vs_lvPPA_FTLD | semi_voyelles_mean | 0,001 | 0,89 | large |
| Pathotype | svPPA_FTLD_vs_lvPPA_AD | nasales_kurtosis | 0,001 | 0,83 | large |
| Pathotype | FTLD_vs_lvPPA_FTLD | semi_voyelles_percentile_90 | 0,001 | 0,89 | large |
| Pathotype | svPPA_FTLD_vs_healthy_control | delta_mfcc_mean_3 | 0,001 | 0,66 | large |
| Pathotype | FTLD_vs_lvPPA_FTLD | speech_rate_phonemes_per_sec | 0,001 | 0,89 | large |
| Pathotype | FTLD_vs_lvPPA_FTLD | rate_speech_phonemes | 0,001 | 0,89 | large |
| Pathotype | FTLD_vs_lvPPA_FTLD | mean_phoneme_duration | 0,001 | 0,89 | large |
| Pathotype | lvPPA_FTLD_vs_lvPPA_AD | delta_mfcc_mean_4 | 0,001 | 0,93 | large |
| Pathotype | lvPPA_FTLD_vs_lvPPA_AD | chroma_mean_9 | 0,001 | 0,93 | large |
| Pathotype | lvPPA_FTLD_vs_lvPPA_AD | hnr_std,1 | 0,001 | 0,93 | large |
| Pathotype | nfvPPA_FTLD_vs_amnestic_AD | syllable_rate_per_sec | 0,001 | 0,73 | large |
| Pathotype | svPPA_FTLD_vs_FTLD | spectral_instability | 0,001 | 0,78 | large |
| Pathotype | svPPA_FTLD_vs_FTLD | spectral_centroid_slope | 0,001 | 0,78 | large |
| Pathotype | lvPPA_FTLD_vs_lvPPA_AD | liquides_median | 0,001 | 0,93 | large |
| Pathotype | svPPA_FTLD_vs_FTLD | spectral_instability,1 | 0,001 | 0,78 | large |
| Pathotype | svPPA_FTLD_vs_FTLD | pause_mean_duration | 0,001 | 0,78 | large |
| Pathotype | svPPA_FTLD_vs_FTLD | inter_word_pause_mean | 0,001 | 0,78 | large |
| Pathotype | svPPA_FTLD_vs_nfvPPA_FTLD | nasales_skewness | 0,001 | 0,92 | large |
| Pathotype | lvPPA_FTLD_vs_amnestic_AD | occlusives_iqr | 0,001 | 0,84 | large |
| Pathotype | lvPPA_FTLD_vs_healthy_control | occlusives_median | 0,001 | 0,76 | large |
| Pathotype | lvPPA_FTLD_vs_healthy_control | fricatives_std | 0,001 | 0,77 | large |
| Pathotype | nfvPPA_FTLD_vs_FTLD | occlusives_median | 0,001 | 0,77 | large |
| Pathotype | lvPPA_FTLD_vs_amnestic_AD | spectral_flux_std | 0,001 | 0,84 | large |
| Pathotype | nfvPPA_FTLD_vs_amnestic_AD | occlusives_iqr | 0,001 | 0,73 | large |
| Pathotype | svPPA_FTLD_vs_amnestic_AD | spectral_flux | 0,001 | 0,73 | large |
| Pathotype | nfvPPA_FTLD_vs_amnestic_AD | shimmer_apq3 | 0,001 | 0,73 | large |
| Pathotype | nfvPPA_FTLD_vs_amnestic_AD | hnr_mean | 0,001 | 0,73 | large |
| Pathotype | svPPA_FTLD_vs_healthy_control | chroma_mean_7 | 0,001 | 0,66 | large |
| Pathotype | nfvPPA_FTLD_vs_amnestic_AD | chroma_mean_8 | 0,001 | 0,73 | large |
| Pathotype | nfvPPA_FTLD_vs_healthy_control | spectral_flux,1 | 0,001 | 0,65 | large |
| Pathotype | FTLD_vs_amnestic_AD | nasales_percentile_10 | 0,001 | 0,56 | large |
| Pathotype | FTLD_vs_lvPPA_FTLD | semi_voyelles_median | 0,001 | 0,87 | large |
| Pathotype | svPPA_FTLD_vs_healthy_control | contrast_mean_0 | 0,001 | 0,65 | large |
| Pathotype | healthy_control_vs_amnestic_AD | voyelles_oral_skewness | 0,001 | 0,42 | medium |
| Pathotype | FTLD_vs_healthy_control | intensity_slope | 0,001 | 0,48 | medium |
| Pathotype | healthy_control_vs_amnestic_AD | f0_min | 0,001 | 0,40 | medium |
| Pathotype | FTLD_vs_lvPPA_FTLD | f2_cv | 0,001 | 0,88 | large |
| Pathotype | nfvPPA_FTLD_vs_FTLD | chroma_mean_3 | 0,001 | 0,77 | large |
| Pathotype | FTLD_vs_lvPPA_FTLD | spectral_instability | 0,001 | 0,88 | large |
| Pathotype | FTLD_vs_lvPPA_FTLD | max_phoneme_duration | 0,001 | 0,88 | large |
| Pathotype | healthy_control_vs_amnestic_AD | f0_slope | 0,001 | 0,41 | medium |
| Pathotype | FTLD_vs_lvPPA_FTLD | liquides_median | 0,001 | 0,88 | large |
| Pathotype | FTLD_vs_lvPPA_FTLD | occlusives_mean | 0,001 | 0,88 | large |
| Pathotype | lvPPA_FTLD_vs_lvPPA_AD | nasales_std | 0,001 | 0,91 | large |
| Pathotype | lvPPA_FTLD_vs_lvPPA_AD | nasales_cv | 0,001 | 0,91 | large |
| Pathotype | svPPA_FTLD_vs_lvPPA_FTLD | num_inter_pauses | 0,001 | 1,00 | large |
| Pathotype | svPPA_FTLD_vs_lvPPA_FTLD | liquides_std | 0,001 | 1,00 | large |
| Pathotype | svPPA_FTLD_vs_lvPPA_FTLD | liquides_cv | 0,001 | 1,00 | large |
| Pathotype | svPPA_FTLD_vs_lvPPA_FTLD | liquides_skewness | 0,001 | 1,00 | large |
| Pathotype | svPPA_FTLD_vs_lvPPA_FTLD | nasales_iqr | 0,001 | 1,00 | large |
| Pathotype | svPPA_FTLD_vs_healthy_control | syllable_rate_per_sec | 0,001 | 0,65 | large |
| Pathotype | svPPA_FTLD_vs_amnestic_AD | delta_mfcc_mean_12 | 0,001 | 0,72 | large |
| Pathotype | lvPPA_FTLD_vs_healthy_control | fricatives_percentile_90 | 0,001 | 0,76 | large |
| Pathotype | lvPPA_FTLD_vs_healthy_control | spectral_instability,1 | 0,001 | 0,76 | large |
| Pathotype | nfvPPA_FTLD_vs_lvPPA_FTLD | occlusives_min | 0,001 | 1,00 | large |
| Pathotype | svPPA_FTLD_vs_lvPPA_FTLD | voyelles_oral_kurtosis | 0,001 | 1,00 | large |
| Pathotype | svPPA_FTLD_vs_lvPPA_FTLD | nasales_std | 0,001 | 1,00 | large |
| Pathotype | svPPA_FTLD_vs_lvPPA_FTLD | nasales_cv | 0,001 | 1,00 | large |
| Pathotype | nfvPPA_FTLD_vs_healthy_control | liquides_cv | 0,001 | 0,65 | large |
| Pathotype | svPPA_FTLD_vs_healthy_control | f1 | 0,001 | 0,65 | large |
| Pathotype | nfvPPA_FTLD_vs_lvPPA_FTLD | spectral_flux | 0,001 | 1,00 | large |
| Pathotype | nfvPPA_FTLD_vs_lvPPA_FTLD | delta_mfcc_mean_4 | 0,001 | 1,00 | large |
| Pathotype | nfvPPA_FTLD_vs_lvPPA_FTLD | intensity_slope | 0,001 | 1,00 | large |
| Pathotype | amnestic_AD_vs_lvPPA_AD | intensity_std | 0,001 | 0,61 | large |
| Pathotype | svPPA_FTLD_vs_lvPPA_FTLD | spectral_flux | 0,001 | 1,00 | large |
| Pathotype | svPPA_FTLD_vs_lvPPA_FTLD | spectral_instability | 0,001 | 1,00 | large |
| Pathotype | svPPA_FTLD_vs_lvPPA_FTLD | delta_mfcc_mean_4 | 0,001 | 1,00 | large |
| Pathotype | svPPA_FTLD_vs_lvPPA_FTLD | delta_mfcc_mean_7 | 0,001 | 1,00 | large |
| Pathotype | svPPA_FTLD_vs_lvPPA_FTLD | delta_mfcc_mean_12 | 0,001 | 1,00 | large |
| Pathotype | svPPA_FTLD_vs_lvPPA_FTLD | chroma_mean_8 | 0,001 | 1,00 | large |
| Pathotype | svPPA_FTLD_vs_lvPPA_FTLD | contrast_mean_2 | 0,001 | 1,00 | large |
| Pathotype | svPPA_FTLD_vs_lvPPA_FTLD | contrast_mean_4 | 0,001 | 1,00 | large |
| Pathotype | svPPA_FTLD_vs_lvPPA_FTLD | rmse_mean | 0,001 | 1,00 | large |
| Pathotype | svPPA_FTLD_vs_lvPPA_FTLD | spectral_flux_mean | 0,001 | 1,00 | large |
| Pathotype | svPPA_FTLD_vs_lvPPA_FTLD | intensity_mean | 0,001 | 1,00 | large |
| Pathotype | svPPA_FTLD_vs_lvPPA_FTLD | intensity_slope | 0,001 | 1,00 | large |
| Pathotype | svPPA_FTLD_vs_lvPPA_FTLD | num_pauses | 0,001 | 1,00 | large |
| Pathotype | svPPA_FTLD_vs_lvPPA_FTLD | num_inter_word_pauses | 0,001 | 1,00 | large |
| Pathotype | svPPA_FTLD_vs_lvPPA_FTLD | occlusives_count | 0,001 | 1,00 | large |
| Pathotype | svPPA_FTLD_vs_lvPPA_FTLD | occlusives_max | 0,001 | 1,00 | large |
| Pathotype | svPPA_FTLD_vs_lvPPA_FTLD | liquides_count | 0,001 | 1,00 | large |
| Pathotype | svPPA_FTLD_vs_lvPPA_FTLD | nasales_count | 0,001 | 1,00 | large |
| Pathotype | nfvPPA_FTLD_vs_lvPPA_FTLD | jitter_local | 0,001 | 1,00 | large |
| Pathotype | nfvPPA_FTLD_vs_lvPPA_FTLD | shimmer_local | 0,001 | 1,00 | large |
| Pathotype | nfvPPA_FTLD_vs_lvPPA_FTLD | shimmer_apq3 | 0,001 | 1,00 | large |
| Pathotype | nfvPPA_FTLD_vs_lvPPA_FTLD | shimmer_apq5 | 0,001 | 1,00 | large |
| Pathotype | nfvPPA_FTLD_vs_lvPPA_FTLD | shimmer_dda | 0,001 | 1,00 | large |
| Pathotype | nfvPPA_FTLD_vs_lvPPA_FTLD | delta_mfcc_mean_1 | 0,001 | 1,00 | large |
| Pathotype | nfvPPA_FTLD_vs_lvPPA_FTLD | delta_mfcc_mean_7 | 0,001 | 1,00 | large |
| Pathotype | nfvPPA_FTLD_vs_lvPPA_FTLD | delta_mfcc_mean_9 | 0,001 | 1,00 | large |
| Pathotype | lvPPA_FTLD_vs_amnestic_AD | fricatives_min | 0,001 | 0,80 | large |
| Pathotype | svPPA_FTLD_vs_lvPPA_FTLD | phoneme_count | 0,001 | 1,00 | large |
| Pathotype | svPPA_FTLD_vs_lvPPA_FTLD | syllable_count | 0,001 | 1,00 | large |
| Pathotype | svPPA_FTLD_vs_lvPPA_FTLD | num_speech_phonemes | 0,001 | 1,00 | large |
| Pathotype | svPPA_FTLD_vs_lvPPA_FTLD | voyelles_oral_count | 0,001 | 1,00 | large |
| Pathotype | svPPA_FTLD_vs_lvPPA_FTLD | fricatives_count | 0,001 | 1,00 | large |
| Pathotype | svPPA_FTLD_vs_lvPPA_FTLD | liquides_mean | 0,001 | 1,00 | large |
| Pathotype | svPPA_FTLD_vs_lvPPA_FTLD | liquides_max | 0,001 | 1,00 | large |
| Pathotype | svPPA_FTLD_vs_lvPPA_FTLD | liquides_percentile_90 | 0,001 | 1,00 | large |
| Pathotype | svPPA_FTLD_vs_lvPPA_FTLD | nasales_max | 0,001 | 1,00 | large |
| Pathotype | nfvPPA_FTLD_vs_healthy_control | f0_mean | 0,001 | 0,65 | large |
| Pathotype | nfvPPA_FTLD_vs_lvPPA_FTLD | speech_rate_phonemes_per_sec | 0,001 | 1,00 | large |
| Pathotype | nfvPPA_FTLD_vs_lvPPA_FTLD | rate_speech_phonemes | 0,001 | 1,00 | large |
| Pathotype | FTLD_vs_amnestic_AD | nasales_min | 0,001 | 0,55 | large |
| Pathotype | svPPA_FTLD_vs_nfvPPA_FTLD | pause_frequency | 0,001 | 0,90 | large |
| Pathotype | healthy_control_vs_lvPPA_AD | liquides_min | 0,001 | 0,51 | large |
| Pathotype | svPPA_FTLD_vs_lvPPA_FTLD | speech_duration | 0,001 | 1,00 | large |
| Pathotype | svPPA_FTLD_vs_lvPPA_FTLD | occlusives_std | 0,001 | 1,00 | large |
| Pathotype | svPPA_FTLD_vs_lvPPA_FTLD | occlusives_percentile_90 | 0,001 | 1,00 | large |
| Pathotype | svPPA_FTLD_vs_lvPPA_FTLD | occlusives_iqr | 0,001 | 1,00 | large |
| Pathotype | svPPA_FTLD_vs_lvPPA_FTLD | nasales_percentile_90 | 0,001 | 1,00 | large |
| Pathotype | amnestic_AD_vs_lvPPA_AD | chroma_mean_3 | 0,001 | 0,60 | large |
| Pathotype | svPPA_FTLD_vs_healthy_control | chroma_mean_3 | 0,001 | 0,65 | large |
| Pathotype | healthy_control_vs_amnestic_AD | f2_cv | 0,001 | 0,41 | medium |
| Pathotype | svPPA_FTLD_vs_lvPPA_AD | long_pause_count | 0,001 | 0,80 | large |
| Pathotype | svPPA_FTLD_vs_lvPPA_AD | semi_voyelles_min | 0,001 | 0,79 | large |
| Pathotype | healthy_control_vs_amnestic_AD | pause_std_duration | 0,001 | 0,41 | medium |
| Pathotype | healthy_control_vs_amnestic_AD | inter_word_pause_std | 0,001 | 0,41 | medium |
| Pathotype | amnestic_AD_vs_lvPPA_AD | delta_mfcc_mean_9 | 0,001 | 0,60 | large |
| Pathotype | nfvPPA_FTLD_vs_amnestic_AD | hnr_mean,1 | 0,001 | 0,71 | large |
| Pathotype | nfvPPA_FTLD_vs_healthy_control | pause_ratio | 0,001 | 0,64 | large |
| Pathotype | FTLD_vs_lvPPA_FTLD | fricatives_count | 0,001 | 0,86 | large |
| Pathotype | nfvPPA_FTLD_vs_healthy_control | semi_voyelles_median | 0,001 | 0,64 | large |
| Pathotype | healthy_control_vs_lvPPA_AD | nasales_kurtosis | 0,001 | 0,53 | large |
| Pathotype | nfvPPA_FTLD_vs_lvPPA_AD | contrast_mean_1 | 0,001 | 0,80 | large |
| Pathotype | FTLD_vs_healthy_control | chroma_mean_1 | 0,001 | 0,48 | medium |
| Pathotype | svPPA_FTLD_vs_lvPPA_AD | delta_mfcc_mean_11 | 0,001 | 0,80 | large |
| Pathotype | svPPA_FTLD_vs_lvPPA_AD | f0_std | 0,001 | 0,80 | large |
| Pathotype | lvPPA_FTLD_vs_healthy_control | f0_min | 0,001 | 0,75 | large |
| Pathotype | nfvPPA_FTLD_vs_lvPPA_AD | hnr_mean | 0,001 | 0,80 | large |
| Pathotype | nfvPPA_FTLD_vs_lvPPA_AD | delta_mfcc_mean_11 | 0,001 | 0,80 | large |
| Pathotype | nfvPPA_FTLD_vs_lvPPA_AD | f0_mean | 0,001 | 0,80 | large |
| Pathotype | nfvPPA_FTLD_vs_lvPPA_AD | hnr_mean,1 | 0,001 | 0,80 | large |
| Pathotype | nfvPPA_FTLD_vs_lvPPA_AD | pause_median_duration | 0,001 | 0,80 | large |
| Pathotype | nfvPPA_FTLD_vs_lvPPA_AD | inter_word_pause_median | 0,001 | 0,80 | large |
| Pathotype | lvPPA_FTLD_vs_healthy_control | delta_mfcc_mean_10 | 0,001 | 0,75 | large |
| Pathotype | FTLD_vs_healthy_control | semi_voyelles_std | 0,001 | 0,47 | medium |
| Pathotype | FTLD_vs_healthy_control | nasales_max | 0,001 | 0,47 | medium |
| Pathotype | FTLD_vs_healthy_control | fricatives_cv | 0,001 | 0,47 | medium |
| Pathotype | svPPA_FTLD_vs_amnestic_AD | liquides_iqr | 0,001 | 0,71 | large |
| Pathotype | svPPA_FTLD_vs_amnestic_AD | rolloff_mean | 0,001 | 0,71 | large |
| Pathotype | svPPA_FTLD_vs_amnestic_AD | intensity_std | 0,001 | 0,71 | large |
| Pathotype | FTLD_vs_lvPPA_AD | nasales_median | 0,001 | 0,65 | large |
| Pathotype | nfvPPA_FTLD_vs_FTLD | delta_mfcc_mean_1 | 0,001 | 0,75 | large |
| Pathotype | svPPA_FTLD_vs_healthy_control | liquides_mean | 0,001 | 0,64 | large |
| Pathotype | svPPA_FTLD_vs_healthy_control | occlusives_mean | 0,001 | 0,64 | large |
| Pathotype | lvPPA_FTLD_vs_healthy_control | pause_ratio | 0,001 | 0,75 | large |
| Pathotype | svPPA_FTLD_vs_FTLD | jitter_ppq5 | 0,001 | 0,75 | large |
| Pathotype | svPPA_FTLD_vs_FTLD | f0_slope | 0,001 | 0,75 | large |
| Pathotype | svPPA_FTLD_vs_healthy_control | semi_voyelles_mean | 0,001 | 0,63 | large |
| Pathotype | svPPA_FTLD_vs_FTLD | std_phoneme_duration | 0,001 | 0,75 | large |
| Pathotype | lvPPA_FTLD_vs_lvPPA_AD | chroma_mean_5 | 0,001 | 0,89 | large |
| Pathotype | lvPPA_FTLD_vs_lvPPA_AD | contrast_mean_0 | 0,001 | 0,89 | large |
| Pathotype | lvPPA_FTLD_vs_lvPPA_AD | min_phoneme_duration | 0,001 | 0,86 | large |
| Pathotype | lvPPA_FTLD_vs_healthy_control | fricatives_cv | 0,001 | 0,75 | large |
| Pathotype | lvPPA_FTLD_vs_lvPPA_AD | occlusives_std | 0,001 | 0,89 | large |
| Pathotype | lvPPA_FTLD_vs_lvPPA_AD | occlusives_iqr | 0,001 | 0,89 | large |
| Pathotype | svPPA_FTLD_vs_healthy_control | fricatives_mean | 0,001 | 0,64 | large |
| Pathotype | healthy_control_vs_amnestic_AD | liquides_iqr | 0,001 | 0,40 | medium |
| Pathotype | FTLD_vs_lvPPA_FTLD | liquides_skewness | 0,001 | 0,85 | large |
| Pathotype | nfvPPA_FTLD_vs_amnestic_AD | fricatives_percentile_90 | 0,001 | 0,70 | large |
| Pathotype | lvPPA_FTLD_vs_healthy_control | nasales_median | 0,001 | 0,73 | large |
| Pathotype | svPPA_FTLD_vs_healthy_control | pause_mean_duration | 0,001 | 0,63 | large |
| Pathotype | svPPA_FTLD_vs_healthy_control | inter_word_pause_mean | 0,001 | 0,63 | large |
| Pathotype | svPPA_FTLD_vs_amnestic_AD | delta_mfcc_mean_8 | 0,001 | 0,70 | large |
| Pathotype | nfvPPA_FTLD_vs_lvPPA_FTLD | liquides_std | 0,001 | 0,97 | large |
| Pathotype | nfvPPA_FTLD_vs_lvPPA_AD | chroma_mean_9 | 0,001 | 0,79 | large |
| Pathotype | FTLD_vs_healthy_control | fricatives_max | 0,001 | 0,47 | medium |
| Pathotype | amnestic_AD_vs_lvPPA_AD | pause_std_duration | 0,001 | 0,59 | large |
| Pathotype | amnestic_AD_vs_lvPPA_AD | inter_word_pause_std | 0,001 | 0,59 | large |
| Pathotype | FTLD_vs_lvPPA_FTLD | fricatives_max | 0,001 | 0,85 | large |
| Pathotype | nfvPPA_FTLD_vs_lvPPA_FTLD | num_inter_pauses | 0,001 | 0,97 | large |
| Pathotype | nfvPPA_FTLD_vs_lvPPA_FTLD | liquides_iqr | 0,001 | 0,97 | large |
| Pathotype | nfvPPA_FTLD_vs_lvPPA_FTLD | nasales_iqr | 0,001 | 0,97 | large |
| Pathotype | FTLD_vs_lvPPA_FTLD | hnr_std,1 | 0,001 | 0,85 | large |
| Pathotype | lvPPA_FTLD_vs_amnestic_AD | intensity_dynamic_range | 0,001 | 0,80 | large |
| Pathotype | lvPPA_FTLD_vs_lvPPA_AD | nasales_count | 0,001 | 0,88 | large |
| Pathotype | healthy_control_vs_amnestic_AD | nasales_mean | 0,001 | 0,40 | medium |
| Pathotype | nfvPPA_FTLD_vs_healthy_control | pause_proportion | 0,001 | 0,63 | large |
| Pathotype | nfvPPA_FTLD_vs_healthy_control | occlusives_std | 0,001 | 0,63 | large |
| Pathotype | lvPPA_FTLD_vs_healthy_control | hnr_std,1 | 0,001 | 0,74 | large |
| Pathotype | lvPPA_FTLD_vs_amnestic_AD | intensity_std | 0,001 | 0,80 | large |
| Pathotype | healthy_control_vs_amnestic_AD | f1 | 0,001 | 0,40 | medium |
| Pathotype | svPPA_FTLD_vs_lvPPA_FTLD | f1_cv | 0,001 | 0,97 | large |
| Pathotype | svPPA_FTLD_vs_lvPPA_FTLD | hnr_mean | 0,001 | 0,97 | large |
| Pathotype | svPPA_FTLD_vs_lvPPA_FTLD | delta_mfcc_mean_1 | 0,001 | 0,97 | large |
| Pathotype | svPPA_FTLD_vs_lvPPA_FTLD | spectral_centroid_slope | 0,001 | 0,97 | large |
| Pathotype | svPPA_FTLD_vs_lvPPA_FTLD | rolloff_mean | 0,001 | 0,97 | large |
| Pathotype | svPPA_FTLD_vs_lvPPA_FTLD | f0_max | 0,001 | 0,97 | large |
| Pathotype | svPPA_FTLD_vs_lvPPA_FTLD | hnr_std,1 | 0,001 | 0,97 | large |
| Pathotype | healthy_control_vs_lvPPA_AD | nasales_percentile_90 | 0,001 | 0,52 | large |
| Pathotype | svPPA_FTLD_vs_FTLD | jitter_rap | 0,001 | 0,74 | large |
| Pathotype | nfvPPA_FTLD_vs_lvPPA_FTLD | spectral_centroid_slope | 0,001 | 0,97 | large |
| Pathotype | lvPPA_FTLD_vs_healthy_control | occlusives_skewness | 0,001 | 0,74 | large |
| Pathotype | svPPA_FTLD_vs_lvPPA_FTLD | pause_frequency | 0,001 | 0,97 | large |
| Pathotype | healthy_control_vs_lvPPA_AD | delta_mfcc_mean_11 | 0,001 | 0,52 | large |
| Pathotype | svPPA_FTLD_vs_FTLD | voyelles_oral_cv | 0,001 | 0,74 | large |
| Pathotype | nfvPPA_FTLD_vs_amnestic_AD | spectral_instability,1 | 0,001 | 0,69 | large |
| Pathotype | FTLD_vs_lvPPA_FTLD | semi_voyelles_count | 0,001 | 0,83 | large |
| Pathotype | nfvPPA_FTLD_vs_amnestic_AD | f0_mean | 0,001 | 0,69 | large |
| Pathotype | svPPA_FTLD_vs_lvPPA_FTLD | voyelles_oral_skewness | 0,001 | 0,97 | large |
| Pathotype | svPPA_FTLD_vs_lvPPA_FTLD | occlusives_mean | 0,001 | 0,97 | large |
| Pathotype | svPPA_FTLD_vs_amnestic_AD | liquides_mean | 0,001 | 0,69 | large |
| Pathotype | healthy_control_vs_lvPPA_AD | chroma_mean_4 | 0,001 | 0,52 | large |
| Pathotype | nfvPPA_FTLD_vs_amnestic_AD | occlusives_count | 0,001 | 0,69 | large |
| Pathotype | lvPPA_FTLD_vs_amnestic_AD | spectral_instability | 0,001 | 0,79 | large |
| Pathotype | amnestic_AD_vs_lvPPA_AD | f0_std | 0,001 | 0,58 | large |
| Pathotype | amnestic_AD_vs_lvPPA_AD | nasales_kurtosis | 0,001 | 0,58 | large |
| Pathotype | lvPPA_FTLD_vs_healthy_control | f0_mean | 0,001 | 0,73 | large |
| Pathotype | nfvPPA_FTLD_vs_healthy_control | voyelles_oral_skewness | 0,001 | 0,62 | large |
| Pathotype | nfvPPA_FTLD_vs_healthy_control | voyelles_oral_kurtosis | 0,001 | 0,62 | large |
| Pathotype | svPPA_FTLD_vs_healthy_control | delta_mfcc_mean_8 | 0,001 | 0,62 | large |
| Pathotype | lvPPA_FTLD_vs_lvPPA_AD | f3_cv | 0,001 | 0,88 | large |
| Pathotype | lvPPA_FTLD_vs_lvPPA_AD | delta_mfcc_mean_7 | 0,001 | 0,88 | large |
| Pathotype | lvPPA_FTLD_vs_lvPPA_AD | delta_mfcc_mean_10 | 0,001 | 0,88 | large |
| Pathotype | lvPPA_FTLD_vs_lvPPA_AD | rmse_mean | 0,001 | 0,88 | large |
| Pathotype | lvPPA_FTLD_vs_lvPPA_AD | intensity_mean | 0,001 | 0,88 | large |
| Pathotype | lvPPA_FTLD_vs_lvPPA_AD | f3_cv,1 | 0,001 | 0,88 | large |
| Pathotype | lvPPA_FTLD_vs_lvPPA_AD | occlusives_max | 0,001 | 0,88 | large |
| Pathotype | nfvPPA_FTLD_vs_lvPPA_AD | hnr_std | 0,001 | 0,78 | large |
| Pathotype | nfvPPA_FTLD_vs_lvPPA_AD | hnr_std,1 | 0,001 | 0,78 | large |
| Pathotype | svPPA_FTLD_vs_lvPPA_AD | liquides_iqr | 0,001 | 0,78 | large |
| Pathotype | lvPPA_FTLD_vs_lvPPA_AD | occlusives_percentile_90 | 0,001 | 0,88 | large |
| Pathotype | lvPPA_FTLD_vs_healthy_control | pause_total_duration | 0,001 | 0,73 | large |
| Pathotype | FTLD_vs_lvPPA_FTLD | spectral_flux_std | 0,001 | 0,84 | large |
| Pathotype | nfvPPA_FTLD_vs_FTLD | jitter_rap | 0,001 | 0,73 | large |
| Pathotype | FTLD_vs_lvPPA_FTLD | voyelles_oral_skewness | 0,001 | 0,84 | large |
| Pathotype | FTLD_vs_lvPPA_FTLD | liquides_mean | 0,001 | 0,84 | large |
| Pathotype | FTLD_vs_lvPPA_AD | chroma_mean_9 | 0,001 | 0,63 | large |
| Pathotype | nfvPPA_FTLD_vs_FTLD | jitter_local | 0,001 | 0,73 | large |
| Pathotype | healthy_control_vs_amnestic_AD | nasales_min | 0,001 | 0,39 | medium |
| Pathotype | amnestic_AD_vs_lvPPA_AD | spectral_flux | 0,001 | 0,58 | large |
| Pathotype | lvPPA_FTLD_vs_healthy_control | pause_median_duration | 0,001 | 0,73 | large |
| Pathotype | lvPPA_FTLD_vs_healthy_control | inter_word_pause_median | 0,001 | 0,73 | large |
| Pathotype | svPPA_FTLD_vs_healthy_control | fricatives_median | 0,001 | 0,62 | large |
| Pathotype | svPPA_FTLD_vs_healthy_control | semi_voyelles_percentile_90 | 0,001 | 0,62 | large |
| Pathotype | nfvPPA_FTLD_vs_healthy_control | nasales_mean | 0,001 | 0,62 | large |
| Pathotype | nfvPPA_FTLD_vs_healthy_control | b2 | 0,001 | 0,62 | large |
| Pathotype | lvPPA_FTLD_vs_lvPPA_AD | nasales_max | 0,001 | 0,87 | large |
| Pathotype | lvPPA_FTLD_vs_lvPPA_AD | nasales_percentile_90 | 0,001 | 0,87 | large |
| Pathotype | svPPA_FTLD_vs_amnestic_AD | chroma_mean_2 | 0,001 | 0,68 | large |
| Pathotype | nfvPPA_FTLD_vs_amnestic_AD | rolloff_mean | 0,001 | 0,68 | large |
| Pathotype | nfvPPA_FTLD_vs_amnestic_AD | b2 | 0,001 | 0,68 | large |
| Pathotype | FTLD_vs_healthy_control | min_phoneme_duration | 0,001 | 0,43 | medium |
| Pathotype | lvPPA_FTLD_vs_healthy_control | delta_mfcc_mean_3 | 0,001 | 0,72 | large |
| Pathotype | svPPA_FTLD_vs_amnestic_AD | median_phoneme_duration | 0,001 | 0,66 | large |
| Pathotype | svPPA_FTLD_vs_healthy_control | rmse_mean | 0,001 | 0,62 | large |
| Pathotype | svPPA_FTLD_vs_healthy_control | intensity_mean | 0,001 | 0,62 | large |
| Pathotype | svPPA_FTLD_vs_healthy_control | pause_min_duration | 0,001 | 0,60 | large |
| Pathotype | svPPA_FTLD_vs_healthy_control | inter_word_pause_min | 0,001 | 0,60 | large |
| Pathotype | nfvPPA_FTLD_vs_FTLD | occlusives_percentile_90 | 0,001 | 0,72 | large |
| Pathotype | svPPA_FTLD_vs_FTLD | hnr_mean | 0,001 | 0,72 | large |
| Pathotype | nfvPPA_FTLD_vs_healthy_control | fricatives_skewness | 0,001 | 0,61 | large |
| Pathotype | nfvPPA_FTLD_vs_FTLD | fricatives_percentile_90 | 0,001 | 0,72 | large |
| Pathotype | nfvPPA_FTLD_vs_lvPPA_AD | f0_slope | 0,001 | 0,76 | large |
| Pathotype | nfvPPA_FTLD_vs_lvPPA_AD | syllable_rate_per_sec | 0,001 | 0,76 | large |
| Pathotype | svPPA_FTLD_vs_lvPPA_FTLD | fricatives_skewness | 0,001 | 0,94 | large |
| Pathotype | lvPPA_FTLD_vs_healthy_control | std_phoneme_duration | 0,001 | 0,72 | large |
| Pathotype | svPPA_FTLD_vs_FTLD | occlusives_mean | 0,001 | 0,72 | large |
| Pathotype | FTLD_vs_healthy_control | voyelles_oral_percentile_10 | 0,001 | 0,42 | medium |
| Pathotype | FTLD_vs_lvPPA_FTLD | spectral_centroid_slope | 0,001 | 0,82 | large |
| Pathotype | nfvPPA_FTLD_vs_healthy_control | f1 | 0,001 | 0,61 | large |
| Pathotype | nfvPPA_FTLD_vs_amnestic_AD | syllable_count | 0,001 | 0,68 | large |
| Pathotype | nfvPPA_FTLD_vs_amnestic_AD | voyelles_oral_count | 0,001 | 0,68 | large |
| Pathotype | svPPA_FTLD_vs_lvPPA_FTLD | contrast_mean_0 | 0,001 | 0,94 | large |
| Pathotype | FTLD_vs_lvPPA_FTLD | spectral_instability,1 | 0,001 | 0,82 | large |
| Pathotype | nfvPPA_FTLD_vs_lvPPA_FTLD | spectral_instability | 0,001 | 0,94 | large |
| Pathotype | nfvPPA_FTLD_vs_lvPPA_FTLD | delta_mfcc_mean_12 | 0,001 | 0,94 | large |
| Pathotype | nfvPPA_FTLD_vs_lvPPA_FTLD | min_phoneme_duration | 0,001 | 0,94 | large |
| Pathotype | svPPA_FTLD_vs_amnestic_AD | rolloff_std | 0,001 | 0,68 | large |
| Pathotype | nfvPPA_FTLD_vs_healthy_control | voyelles_oral_mean | 0,001 | 0,61 | large |
| Pathotype | lvPPA_FTLD_vs_lvPPA_AD | max_phoneme_duration | 0,001 | 0,86 | large |
| Pathotype | lvPPA_FTLD_vs_amnestic_AD | f0_mean | 0,001 | 0,78 | large |
| Pathotype | healthy_control_vs_amnestic_AD | delta_mfcc_mean_2 | 0,001 | 0,39 | medium |
| Pathotype | FTLD_vs_healthy_control | occlusives_iqr | 0,001 | 0,45 | medium |
| Pathotype | lvPPA_FTLD_vs_amnestic_AD | f3_cv,1 | 0,001 | 0,78 | large |
| Pathotype | FTLD_vs_lvPPA_AD | spectral_flux_mean | 0,001 | 0,62 | large |
| Pathotype | lvPPA_FTLD_vs_amnestic_AD | pause_total_duration | 0,001 | 0,78 | large |
| Pathotype | lvPPA_FTLD_vs_healthy_control | zcr_mean | 0,001 | 0,71 | large |
| Pathotype | lvPPA_FTLD_vs_healthy_control | spectral_centroid_std | 0,001 | 0,71 | large |
| Pathotype | amnestic_AD_vs_lvPPA_AD | occlusives_percentile_90 | 0,002 | 0,57 | large |
| Pathotype | nfvPPA_FTLD_vs_amnestic_AD | occlusives_median | 0,002 | 0,67 | large |
| Pathotype | healthy_control_vs_amnestic_AD | occlusives_min | 0,002 | 0,37 | medium |
| Pathotype | lvPPA_FTLD_vs_amnestic_AD | voyelles_oral_max | 0,002 | 0,77 | large |
| Pathotype | svPPA_FTLD_vs_healthy_control | f0_mean | 0,002 | 0,61 | large |
| Pathotype | lvPPA_FTLD_vs_healthy_control | chroma_mean_9 | 0,002 | 0,71 | large |
| Pathotype | svPPA_FTLD_vs_amnestic_AD | chroma_mean_6 | 0,002 | 0,67 | large |
| Pathotype | nfvPPA_FTLD_vs_FTLD | delta_mfcc_mean_8 | 0,002 | 0,71 | large |
| Pathotype | nfvPPA_FTLD_vs_FTLD | delta_mfcc_mean_9 | 0,002 | 0,71 | large |
| Pathotype | lvPPA_FTLD_vs_healthy_control | pause_mean_duration | 0,002 | 0,71 | large |
| Pathotype | lvPPA_FTLD_vs_healthy_control | inter_word_pause_mean | 0,002 | 0,71 | large |
| Pathotype | svPPA_FTLD_vs_healthy_control | occlusives_std | 0,002 | 0,60 | large |
| Pathotype | svPPA_FTLD_vs_nfvPPA_FTLD | liquides_kurtosis | 0,002 | 0,84 | large |
| Pathotype | amnestic_AD_vs_lvPPA_AD | rmse_mean | 0,002 | 0,56 | large |
| Pathotype | nfvPPA_FTLD_vs_lvPPA_AD | spectral_instability | 0,002 | 0,75 | large |
| Pathotype | nfvPPA_FTLD_vs_lvPPA_AD | spectral_flux_mean | 0,002 | 0,75 | large |
| Pathotype | nfvPPA_FTLD_vs_lvPPA_AD | spectral_instability,1 | 0,002 | 0,75 | large |
| Pathotype | FTLD_vs_lvPPA_FTLD | contrast_mean_6 | 0,002 | 0,81 | large |
| Pathotype | FTLD_vs_lvPPA_FTLD | voyelles_oral_kurtosis | 0,002 | 0,81 | large |
| Pathotype | healthy_control_vs_lvPPA_AD | intensity_dynamic_range | 0,002 | 0,49 | medium |
| Pathotype | FTLD_vs_amnestic_AD | min_phoneme_duration | 0,002 | 0,49 | medium |
| Pathotype | svPPA_FTLD_vs_lvPPA_FTLD | occlusives_median | 0,002 | 0,93 | large |
| Pathotype | healthy_control_vs_amnestic_AD | hnr_std | 0,002 | 0,38 | medium |
| Pathotype | lvPPA_FTLD_vs_healthy_control | voyelles_oral_percentile_10 | 0,002 | 0,63 | large |
| Pathotype | svPPA_FTLD_vs_lvPPA_FTLD | median_phoneme_duration | 0,002 | 0,93 | large |
| Pathotype | lvPPA_FTLD_vs_lvPPA_AD | nasales_iqr | 0,002 | 0,84 | large |
| Pathotype | nfvPPA_FTLD_vs_amnestic_AD | delta_mfcc_mean_2 | 0,002 | 0,66 | large |
| Pathotype | nfvPPA_FTLD_vs_amnestic_AD | median_phoneme_duration | 0,002 | 0,64 | large |
| Pathotype | nfvPPA_FTLD_vs_healthy_control | chroma_mean_0 | 0,002 | 0,60 | large |
| Pathotype | lvPPA_FTLD_vs_healthy_control | chroma_mean_1 | 0,002 | 0,70 | large |
| Pathotype | lvPPA_FTLD_vs_healthy_control | contrast_mean_1 | 0,002 | 0,70 | large |
| Pathotype | lvPPA_FTLD_vs_lvPPA_AD | voyelles_oral_kurtosis | 0,002 | 0,84 | large |
| Pathotype | nfvPPA_FTLD_vs_healthy_control | hnr_std,1 | 0,002 | 0,60 | large |
| Pathotype | lvPPA_FTLD_vs_lvPPA_AD | jitter_rap | 0,002 | 0,84 | large |
| Pathotype | lvPPA_FTLD_vs_lvPPA_AD | contrast_mean_6 | 0,002 | 0,84 | large |
| Pathotype | lvPPA_FTLD_vs_healthy_control | f0_max | 0,002 | 0,70 | large |
| Pathotype | healthy_control_vs_amnestic_AD | pause_max_duration | 0,002 | 0,38 | medium |
| Pathotype | healthy_control_vs_amnestic_AD | inter_word_pause_max | 0,002 | 0,38 | medium |
| Pathotype | lvPPA_FTLD_vs_lvPPA_AD | voyelles_oral_skewness | 0,002 | 0,84 | large |
| Pathotype | svPPA_FTLD_vs_FTLD | shimmer_apq5 | 0,002 | 0,70 | large |
| Pathotype | svPPA_FTLD_vs_healthy_control | rolloff_std | 0,002 | 0,60 | large |
| Pathotype | nfvPPA_FTLD_vs_lvPPA_AD | long_pause_count | 0,002 | 0,74 | large |
| Pathotype | lvPPA_FTLD_vs_healthy_control | chroma_mean_3 | 0,002 | 0,70 | large |
| Pathotype | lvPPA_FTLD_vs_healthy_control | chroma_mean_4 | 0,002 | 0,70 | large |
| Pathotype | lvPPA_FTLD_vs_healthy_control | chroma_mean_6 | 0,002 | 0,70 | large |
| Pathotype | lvPPA_FTLD_vs_healthy_control | pause_frequency | 0,002 | 0,70 | large |
| Pathotype | FTLD_vs_healthy_control | pause_frequency | 0,002 | 0,44 | medium |
| Pathotype | nfvPPA_FTLD_vs_lvPPA_FTLD | liquides_cv | 0,002 | 0,91 | large |
| Pathotype | lvPPA_FTLD_vs_amnestic_AD | f0_slope | 0,002 | 0,76 | large |
| Pathotype | lvPPA_FTLD_vs_amnestic_AD | speech_rate_phonemes_per_sec | 0,002 | 0,76 | large |
| Pathotype | lvPPA_FTLD_vs_amnestic_AD | rate_speech_phonemes | 0,002 | 0,76 | large |
| Pathotype | lvPPA_FTLD_vs_amnestic_AD | fricatives_std | 0,002 | 0,76 | large |
| Pathotype | nfvPPA_FTLD_vs_lvPPA_FTLD | nasales_std | 0,002 | 0,91 | large |
| Pathotype | nfvPPA_FTLD_vs_lvPPA_FTLD | nasales_cv | 0,002 | 0,91 | large |
| Pathotype | lvPPA_FTLD_vs_healthy_control | pause_proportion | 0,002 | 0,69 | large |
| Pathotype | nfvPPA_FTLD_vs_healthy_control | hnr_std | 0,002 | 0,59 | large |
| Pathotype | nfvPPA_FTLD_vs_amnestic_AD | spectral_centroid_mean | 0,002 | 0,66 | large |
| Pathotype | healthy_control_vs_lvPPA_AD | nasales_std | 0,002 | 0,49 | medium |
| Pathotype | nfvPPA_FTLD_vs_lvPPA_AD | pause_total_duration | 0,002 | 0,74 | large |
| Pathotype | healthy_control_vs_lvPPA_AD | nasales_iqr | 0,002 | 0,49 | medium |
| Pathotype | svPPA_FTLD_vs_lvPPA_FTLD | f2_cv | 0,002 | 0,91 | large |
| Pathotype | svPPA_FTLD_vs_lvPPA_FTLD | f0_slope | 0,002 | 0,91 | large |
| Pathotype | nfvPPA_FTLD_vs_lvPPA_AD | nasales_median | 0,002 | 0,74 | large |
| Pathotype | lvPPA_FTLD_vs_lvPPA_AD | semi_voyelles_max | 0,002 | 0,83 | large |
| Pathotype | nfvPPA_FTLD_vs_healthy_control | median_phoneme_duration | 0,002 | 0,56 | large |
| Pathotype | lvPPA_FTLD_vs_lvPPA_AD | semi_voyelles_mean | 0,002 | 0,83 | large |
| Pathotype | lvPPA_FTLD_vs_lvPPA_AD | semi_voyelles_percentile_90 | 0,002 | 0,83 | large |
| Pathotype | FTLD_vs_lvPPA_AD | hnr_std,1 | 0,002 | 0,60 | large |
| Pathotype | svPPA_FTLD_vs_amnestic_AD | occlusives_median | 0,002 | 0,65 | large |
| Pathotype | nfvPPA_FTLD_vs_lvPPA_FTLD | speech_duration | 0,002 | 0,91 | large |
| Pathotype | FTLD_vs_lvPPA_FTLD | semi_voyelles_std | 0,002 | 0,76 | large |
| Pathotype | FTLD_vs_lvPPA_FTLD | semi_voyelles_cv | 0,002 | 0,76 | large |
| Pathotype | lvPPA_FTLD_vs_healthy_control | liquides_min | 0,002 | 0,65 | large |
| Pathotype | lvPPA_FTLD_vs_amnestic_AD | hnr_mean | 0,002 | 0,75 | large |
| Pathotype | lvPPA_FTLD_vs_healthy_control | chroma_mean_2 | 0,002 | 0,69 | large |
| Pathotype | lvPPA_FTLD_vs_healthy_control | cv_phoneme_duration | 0,002 | 0,69 | large |
| Pathotype | nfvPPA_FTLD_vs_healthy_control | nasales_skewness | 0,002 | 0,59 | large |
| Pathotype | svPPA_FTLD_vs_healthy_control | fricatives_skewness | 0,002 | 0,59 | large |
| Pathotype | nfvPPA_FTLD_vs_FTLD | mean_phoneme_duration | 0,002 | 0,70 | large |
| Pathotype | svPPA_FTLD_vs_amnestic_AD | spectral_centroid_std | 0,002 | 0,65 | large |
| Pathotype | svPPA_FTLD_vs_FTLD | semi_voyelles_median | 0,002 | 0,69 | large |
| Pathotype | amnestic_AD_vs_lvPPA_AD | intensity_mean | 0,002 | 0,55 | large |
| Pathotype | FTLD_vs_amnestic_AD | shimmer_local | 0,002 | 0,50 | large |
| Pathotype | FTLD_vs_amnestic_AD | contrast_mean_6 | 0,002 | 0,50 | large |
| Pathotype | FTLD_vs_healthy_control | syllable_rate_per_sec | 0,002 | 0,43 | medium |
| Pathotype | lvPPA_FTLD_vs_lvPPA_AD | delta_mfcc_mean_8 | 0,002 | 0,82 | large |
| Pathotype | lvPPA_FTLD_vs_lvPPA_AD | chroma_mean_10 | 0,002 | 0,82 | large |
| Pathotype | lvPPA_FTLD_vs_lvPPA_AD | spectral_centroid_mean | 0,002 | 0,82 | large |
| Pathotype | FTLD_vs_healthy_control | voyelles_oral_median | 0,002 | 0,42 | medium |
| Pathotype | lvPPA_FTLD_vs_lvPPA_AD | occlusives_mean | 0,002 | 0,82 | large |
| Pathotype | nfvPPA_FTLD_vs_lvPPA_AD | median_phoneme_duration | 0,002 | 0,73 | large |
| Pathotype | healthy_control_vs_lvPPA_AD | zcr_mean | 0,002 | 0,48 | medium |
| Pathotype | lvPPA_FTLD_vs_amnestic_AD | voyelles_oral_median | 0,002 | 0,74 | large |
| Pathotype | lvPPA_FTLD_vs_amnestic_AD | fricatives_cv | 0,002 | 0,74 | large |
| Pathotype | svPPA_FTLD_vs_lvPPA_AD | chroma_mean_9 | 0,002 | 0,73 | large |
| Pathotype | svPPA_FTLD_vs_lvPPA_AD | intensity_slope | 0,002 | 0,73 | large |
| Pathotype | nfvPPA_FTLD_vs_lvPPA_AD | f0_std | 0,002 | 0,73 | large |
| Pathotype | lvPPA_FTLD_vs_healthy_control | voyelles_oral_mean | 0,002 | 0,68 | large |
| Pathotype | lvPPA_FTLD_vs_healthy_control | voyelles_oral_cv | 0,002 | 0,68 | large |
| Pathotype | nfvPPA_FTLD_vs_amnestic_AD | fricatives_std | 0,002 | 0,64 | large |
| Pathotype | svPPA_FTLD_vs_amnestic_AD | speech_rate_phonemes_per_sec | 0,002 | 0,64 | large |
| Pathotype | svPPA_FTLD_vs_amnestic_AD | rate_speech_phonemes | 0,002 | 0,64 | large |
| Pathotype | FTLD_vs_lvPPA_FTLD | fricatives_std | 0,002 | 0,78 | large |
| Pathotype | lvPPA_FTLD_vs_amnestic_AD | fricatives_iqr | 0,002 | 0,74 | large |
| Pathotype | svPPA_FTLD_vs_FTLD | contrast_mean_4 | 0,002 | 0,69 | large |
| Pathotype | svPPA_FTLD_vs_FTLD | intensity_dynamic_range | 0,002 | 0,69 | large |
| Pathotype | svPPA_FTLD_vs_FTLD | liquides_median | 0,002 | 0,69 | large |
| Pathotype | nfvPPA_FTLD_vs_FTLD | voyelles_oral_mean | 0,002 | 0,69 | large |
| Pathotype | svPPA_FTLD_vs_FTLD | speech_rate_phonemes_per_sec | 0,002 | 0,69 | large |
| Pathotype | svPPA_FTLD_vs_FTLD | rate_speech_phonemes | 0,002 | 0,69 | large |
| Pathotype | healthy_control_vs_amnestic_AD | semi_voyelles_kurtosis | 0,003 | 0,37 | medium |
| Pathotype | svPPA_FTLD_vs_lvPPA_AD | liquides_median | 0,003 | 0,72 | large |
| Pathotype | FTLD_vs_amnestic_AD | chroma_mean_5 | 0,003 | 0,49 | medium |
| Pathotype | svPPA_FTLD_vs_amnestic_AD | semi_voyelles_mean | 0,003 | 0,64 | large |
| Pathotype | healthy_control_vs_amnestic_AD | delta_mfcc_mean_4 | 0,003 | 0,37 | medium |
| Pathotype | svPPA_FTLD_vs_lvPPA_AD | semi_voyelles_percentile_10 | 0,003 | 0,71 | large |
| Pathotype | svPPA_FTLD_vs_lvPPA_AD | liquides_percentile_10 | 0,003 | 0,71 | large |
| Pathotype | svPPA_FTLD_vs_healthy_control | fricatives_count | 0,003 | 0,58 | large |
| Pathotype | FTLD_vs_healthy_control | semi_voyelles_mean | 0,003 | 0,42 | medium |
| Pathotype | svPPA_FTLD_vs_healthy_control | delta_mfcc_mean_2 | 0,003 | 0,58 | large |
| Pathotype | svPPA_FTLD_vs_healthy_control | delta_mfcc_mean_11 | 0,003 | 0,58 | large |
| Pathotype | lvPPA_FTLD_vs_healthy_control | b2 | 0,003 | 0,68 | large |
| Pathotype | svPPA_FTLD_vs_amnestic_AD | spectral_centroid_mean | 0,003 | 0,64 | large |
| Pathotype | lvPPA_FTLD_vs_healthy_control | fricatives_iqr | 0,003 | 0,68 | large |
| Pathotype | lvPPA_FTLD_vs_healthy_control | delta_mfcc_mean_2 | 0,003 | 0,68 | large |
| Pathotype | svPPA_FTLD_vs_nfvPPA_FTLD | liquides_percentile_10 | 0,003 | 0,80 | large |
| Pathotype | svPPA_FTLD_vs_nfvPPA_FTLD | liquides_median | 0,003 | 0,80 | large |
| Pathotype | healthy_control_vs_amnestic_AD | chroma_mean_9 | 0,003 | 0,36 | medium |
| Pathotype | svPPA_FTLD_vs_nfvPPA_FTLD | hnr_mean | 0,003 | 0,80 | large |
| Pathotype | svPPA_FTLD_vs_nfvPPA_FTLD | b2 | 0,003 | 0,80 | large |
| Pathotype | svPPA_FTLD_vs_nfvPPA_FTLD | hnr_mean,1 | 0,003 | 0,80 | large |
| Pathotype | svPPA_FTLD_vs_nfvPPA_FTLD | median_phoneme_duration | 0,003 | 0,80 | large |
| Pathotype | svPPA_FTLD_vs_nfvPPA_FTLD | voyelles_oral_max | 0,003 | 0,80 | large |
| Pathotype | svPPA_FTLD_vs_nfvPPA_FTLD | liquides_mean | 0,003 | 0,80 | large |
| Pathotype | svPPA_FTLD_vs_nfvPPA_FTLD | liquides_iqr | 0,003 | 0,80 | large |
| Pathotype | nfvPPA_FTLD_vs_lvPPA_AD | semi_voyelles_max | 0,003 | 0,71 | large |
| Pathotype | svPPA_FTLD_vs_amnestic_AD | fricatives_iqr | 0,003 | 0,63 | large |
| Pathotype | svPPA_FTLD_vs_lvPPA_FTLD | chroma_mean_5 | 0,003 | 0,89 | large |
| Pathotype | svPPA_FTLD_vs_lvPPA_FTLD | chroma_mean_6 | 0,003 | 0,89 | large |
| Pathotype | svPPA_FTLD_vs_lvPPA_FTLD | b2 | 0,003 | 0,89 | large |
| Pathotype | svPPA_FTLD_vs_nfvPPA_FTLD | mean_phoneme_duration | 0,003 | 0,80 | large |
| Pathotype | svPPA_FTLD_vs_nfvPPA_FTLD | voyelles_oral_mean | 0,003 | 0,80 | large |
| Pathotype | svPPA_FTLD_vs_nfvPPA_FTLD | voyelles_oral_std | 0,003 | 0,80 | large |
| Pathotype | svPPA_FTLD_vs_nfvPPA_FTLD | occlusives_percentile_90 | 0,003 | 0,80 | large |
| Pathotype | svPPA_FTLD_vs_nfvPPA_FTLD | fricatives_std | 0,003 | 0,80 | large |
| Pathotype | nfvPPA_FTLD_vs_lvPPA_FTLD | rolloff_mean | 0,003 | 0,89 | large |
| Pathotype | healthy_control_vs_amnestic_AD | hnr_mean | 0,003 | 0,36 | medium |
| Pathotype | FTLD_vs_lvPPA_FTLD | semi_voyelles_percentile_10 | 0,003 | 0,77 | large |
| Pathotype | svPPA_FTLD_vs_lvPPA_FTLD | occlusives_min | 0,003 | 0,89 | large |
| Pathotype | nfvPPA_FTLD_vs_lvPPA_AD | semi_voyelles_skewness | 0,003 | 0,66 | large |
| Pathotype | svPPA_FTLD_vs_FTLD | nasales_median | 0,003 | 0,68 | large |
| Pathotype | nfvPPA_FTLD_vs_lvPPA_AD | occlusives_median | 0,003 | 0,71 | large |
| Pathotype | svPPA_FTLD_vs_FTLD | liquides_mean | 0,003 | 0,68 | large |
| Pathotype | svPPA_FTLD_vs_FTLD | pause_std_duration | 0,003 | 0,68 | large |
| Pathotype | svPPA_FTLD_vs_FTLD | inter_word_pause_std | 0,003 | 0,68 | large |
| Pathotype | nfvPPA_FTLD_vs_healthy_control | nasales_kurtosis | 0,003 | 0,57 | large |
| Pathotype | lvPPA_FTLD_vs_lvPPA_AD | mean_phoneme_duration | 0,003 | 0,80 | large |
| Pathotype | lvPPA_FTLD_vs_lvPPA_AD | speech_rate_phonemes_per_sec | 0,003 | 0,80 | large |
| Pathotype | lvPPA_FTLD_vs_lvPPA_AD | rate_speech_phonemes | 0,003 | 0,80 | large |
| Pathotype | healthy_control_vs_amnestic_AD | liquides_skewness | 0,003 | 0,36 | medium |
| Pathotype | svPPA_FTLD_vs_amnestic_AD | voyelles_oral_std | 0,003 | 0,63 | large |
| Pathotype | FTLD_vs_lvPPA_FTLD | pause_total_duration | 0,003 | 0,77 | large |
| Pathotype | FTLD_vs_lvPPA_FTLD | voyelles_oral_max | 0,003 | 0,77 | large |
| Pathotype | healthy_control_vs_amnestic_AD | semi_voyelles_min | 0,003 | 0,36 | medium |
| Pathotype | healthy_control_vs_lvPPA_AD | delta_mfcc_mean_9 | 0,003 | 0,47 | medium |
| Pathotype | healthy_control_vs_lvPPA_AD | chroma_mean_5 | 0,003 | 0,47 | medium |
| Pathotype | lvPPA_FTLD_vs_healthy_control | f0_slope | 0,003 | 0,67 | large |
| Pathotype | amnestic_AD_vs_lvPPA_AD | spectral_flux_mean | 0,003 | 0,53 | large |
| Pathotype | nfvPPA_FTLD_vs_FTLD | liquides_percentile_10 | 0,003 | 0,67 | large |
| Pathotype | lvPPA_FTLD_vs_healthy_control | rolloff_std | 0,003 | 0,67 | large |
| Pathotype | healthy_control_vs_lvPPA_AD | delta_mfcc_mean_7 | 0,003 | 0,47 | medium |
| Pathotype | FTLD_vs_amnestic_AD | nasales_median | 0,003 | 0,49 | medium |
| Pathotype | lvPPA_FTLD_vs_amnestic_AD | spectral_flux,1 | 0,003 | 0,72 | large |
| Pathotype | lvPPA_FTLD_vs_amnestic_AD | mean_phoneme_duration | 0,003 | 0,72 | large |
| Pathotype | nfvPPA_FTLD_vs_healthy_control | zcr_mean | 0,003 | 0,57 | large |
| Pathotype | svPPA_FTLD_vs_healthy_control | b1 | 0,003 | 0,57 | large |
| Pathotype | amnestic_AD_vs_lvPPA_AD | hnr_std | 0,003 | 0,53 | large |
| Pathotype | amnestic_AD_vs_lvPPA_AD | chroma_mean_11 | 0,003 | 0,53 | large |
| Pathotype | lvPPA_FTLD_vs_amnestic_AD | pause_min_duration | 0,003 | 0,71 | large |
| Pathotype | lvPPA_FTLD_vs_amnestic_AD | inter_word_pause_min | 0,003 | 0,71 | large |
| Pathotype | healthy_control_vs_amnestic_AD | hnr_mean,1 | 0,003 | 0,36 | medium |
| Pathotype | lvPPA_FTLD_vs_lvPPA_AD | semi_voyelles_std | 0,003 | 0,75 | large |
| Pathotype | lvPPA_FTLD_vs_lvPPA_AD | semi_voyelles_cv | 0,003 | 0,75 | large |
| Pathotype | lvPPA_FTLD_vs_lvPPA_AD | semi_voyelles_iqr | 0,003 | 0,75 | large |
| Pathotype | svPPA_FTLD_vs_FTLD | intensity_slope | 0,003 | 0,67 | large |
| Pathotype | healthy_control_vs_lvPPA_AD | spectral_flux_std | 0,003 | 0,46 | medium |
| Pathotype | FTLD_vs_healthy_control | nasales_min | 0,003 | 0,41 | medium |
| Pathotype | svPPA_FTLD_vs_lvPPA_AD | semi_voyelles_median | 0,003 | 0,69 | large |
| Pathotype | svPPA_FTLD_vs_amnestic_AD | voyelles_oral_max | 0,003 | 0,62 | large |
| Pathotype | nfvPPA_FTLD_vs_lvPPA_AD | voyelles_oral_median | 0,003 | 0,70 | large |
| Pathotype | svPPA_FTLD_vs_healthy_control | delta_mfcc_mean_6 | 0,003 | 0,56 | large |
| Pathotype | nfvPPA_FTLD_vs_lvPPA_AD | mean_phoneme_duration | 0,003 | 0,70 | large |
| Pathotype | FTLD_vs_healthy_control | fricatives_mean | 0,003 | 0,42 | medium |
| Pathotype | svPPA_FTLD_vs_lvPPA_AD | pause_total_duration | 0,003 | 0,70 | large |
| Pathotype | lvPPA_FTLD_vs_amnestic_AD | chroma_mean_3 | 0,003 | 0,71 | large |
| Pathotype | amnestic_AD_vs_lvPPA_AD | chroma_mean_7 | 0,003 | 0,52 | large |
| Pathotype | amnestic_AD_vs_lvPPA_AD | contrast_mean_3 | 0,003 | 0,52 | large |
| Pathotype | FTLD_vs_lvPPA_FTLD | contrast_mean_3 | 0,003 | 0,76 | large |
| Pathotype | nfvPPA_FTLD_vs_healthy_control | voyelles_oral_iqr | 0,003 | 0,56 | large |
| Pathotype | lvPPA_FTLD_vs_amnestic_AD | delta_mfcc_mean_2 | 0,003 | 0,71 | large |
| Pathotype | lvPPA_FTLD_vs_amnestic_AD | delta_mfcc_mean_3 | 0,003 | 0,71 | large |
| Pathotype | lvPPA_FTLD_vs_amnestic_AD | zcr_mean | 0,003 | 0,71 | large |
| Pathotype | FTLD_vs_lvPPA_FTLD | occlusives_std | 0,003 | 0,76 | large |
| Pathotype | svPPA_FTLD_vs_nfvPPA_FTLD | voyelles_oral_median | 0,003 | 0,78 | large |
| Pathotype | FTLD_vs_healthy_control | rmse_mean | 0,003 | 0,41 | medium |
| Pathotype | FTLD_vs_healthy_control | intensity_mean | 0,003 | 0,41 | medium |
| Pathotype | lvPPA_FTLD_vs_amnestic_AD | chroma_mean_2 | 0,004 | 0,71 | large |
| Pathotype | lvPPA_FTLD_vs_amnestic_AD | chroma_mean_4 | 0,004 | 0,71 | large |
| Pathotype | lvPPA_FTLD_vs_amnestic_AD | spectral_centroid_std | 0,004 | 0,71 | large |
| Pathotype | lvPPA_FTLD_vs_amnestic_AD | std_phoneme_duration | 0,004 | 0,71 | large |
| Pathotype | nfvPPA_FTLD_vs_amnestic_AD | nasales_median | 0,004 | 0,62 | large |
| Pathotype | svPPA_FTLD_vs_amnestic_AD | f0_mean | 0,004 | 0,62 | large |
| Pathotype | svPPA_FTLD_vs_amnestic_AD | f0_slope | 0,004 | 0,62 | large |
| Pathotype | nfvPPA_FTLD_vs_amnestic_AD | contrast_mean_1 | 0,004 | 0,62 | large |
| Pathotype | nfvPPA_FTLD_vs_healthy_control | fricatives_count | 0,004 | 0,56 | large |
| Pathotype | nfvPPA_FTLD_vs_amnestic_AD | nasales_skewness | 0,004 | 0,62 | large |
| Pathotype | nfvPPA_FTLD_vs_FTLD | semi_voyelles_skewness | 0,004 | 0,61 | large |
| Pathotype | lvPPA_FTLD_vs_lvPPA_AD | f2_cv | 0,004 | 0,79 | large |
| Pathotype | svPPA_FTLD_vs_lvPPA_AD | semi_voyelles_kurtosis | 0,004 | 0,65 | large |
| Pathotype | lvPPA_FTLD_vs_healthy_control | pause_std_duration | 0,004 | 0,65 | large |
| Pathotype | lvPPA_FTLD_vs_healthy_control | inter_word_pause_std | 0,004 | 0,65 | large |
| Pathotype | svPPA_FTLD_vs_lvPPA_FTLD | liquides_iqr | 0,004 | 0,86 | large |
| Pathotype | healthy_control_vs_lvPPA_AD | liquides_skewness | 0,004 | 0,46 | medium |
| Pathotype | svPPA_FTLD_vs_healthy_control | pause_proportion | 0,004 | 0,56 | large |
| Pathotype | nfvPPA_FTLD_vs_lvPPA_FTLD | f0_min | 0,004 | 0,84 | large |
| Pathotype | nfvPPA_FTLD_vs_lvPPA_FTLD | voyelles_oral_kurtosis | 0,004 | 0,86 | large |
| Pathotype | FTLD_vs_lvPPA_AD | nasales_percentile_10 | 0,004 | 0,57 | large |
| Pathotype | nfvPPA_FTLD_vs_FTLD | chroma_mean_0 | 0,004 | 0,66 | large |
| Pathotype | svPPA_FTLD_vs_FTLD | liquides_iqr | 0,004 | 0,66 | large |
| Pathotype | nfvPPA_FTLD_vs_FTLD | nasales_percentile_10 | 0,004 | 0,66 | large |
| Pathotype | healthy_control_vs_lvPPA_AD | fricatives_mean | 0,004 | 0,46 | medium |
| Pathotype | nfvPPA_FTLD_vs_FTLD | voyelles_oral_skewness | 0,004 | 0,66 | large |
| Pathotype | lvPPA_FTLD_vs_healthy_control | voyelles_oral_std | 0,004 | 0,65 | large |
| Pathotype | nfvPPA_FTLD_vs_healthy_control | contrast_mean_5 | 0,004 | 0,56 | large |
| Pathotype | svPPA_FTLD_vs_FTLD | articulation_rate | 0,004 | 0,62 | large |
| Pathotype | healthy_control_vs_lvPPA_AD | liquides_percentile_90 | 0,004 | 0,46 | medium |
| Pathotype | amnestic_AD_vs_lvPPA_AD | liquides_min | 0,004 | 0,51 | large |
| Pathotype | nfvPPA_FTLD_vs_amnestic_AD | delta_mfcc_mean_9 | 0,004 | 0,61 | large |
| Pathotype | lvPPA_FTLD_vs_healthy_control | f1 | 0,004 | 0,65 | large |
| Pathotype | svPPA_FTLD_vs_healthy_control | contrast_mean_6 | 0,004 | 0,55 | large |
| Pathotype | nfvPPA_FTLD_vs_lvPPA_AD | chroma_mean_7 | 0,004 | 0,69 | large |
| Pathotype | lvPPA_FTLD_vs_healthy_control | f2 | 0,004 | 0,65 | large |
| Pathotype | svPPA_FTLD_vs_lvPPA_AD | chroma_mean_8 | 0,004 | 0,69 | large |
| Pathotype | svPPA_FTLD_vs_lvPPA_AD | spectral_centroid_std | 0,004 | 0,69 | large |
| Pathotype | svPPA_FTLD_vs_lvPPA_AD | rolloff_std | 0,004 | 0,69 | large |
| Pathotype | nfvPPA_FTLD_vs_lvPPA_AD | rolloff_mean | 0,004 | 0,69 | large |
| Pathotype | nfvPPA_FTLD_vs_lvPPA_AD | pause_cv | 0,004 | 0,69 | large |
| Pathotype | FTLD_vs_lvPPA_FTLD | fricatives_cv | 0,004 | 0,74 | large |
| Pathotype | FTLD_vs_lvPPA_AD | chroma_mean_10 | 0,004 | 0,56 | large |
| Pathotype | healthy_control_vs_amnestic_AD | pause_frequency | 0,004 | 0,35 | medium |
| Pathotype | nfvPPA_FTLD_vs_healthy_control | liquides_std | 0,004 | 0,55 | large |
| Pathotype | svPPA_FTLD_vs_amnestic_AD | liquides_skewness | 0,004 | 0,61 | large |
| Pathotype | nfvPPA_FTLD_vs_amnestic_AD | voyelles_oral_mean | 0,004 | 0,61 | large |
| Pathotype | nfvPPA_FTLD_vs_amnestic_AD | voyelles_oral_skewness | 0,004 | 0,61 | large |
| Pathotype | nfvPPA_FTLD_vs_FTLD | jitter_ppq5 | 0,004 | 0,65 | large |
| Pathotype | FTLD_vs_healthy_control | liquides_iqr | 0,004 | 0,40 | medium |
| Pathotype | lvPPA_FTLD_vs_amnestic_AD | f2_cv | 0,004 | 0,70 | large |
| Pathotype | healthy_control_vs_amnestic_AD | occlusives_median | 0,004 | 0,34 | medium |
| Pathotype | FTLD_vs_amnestic_AD | f3_cv | 0,004 | 0,47 | medium |
| Pathotype | FTLD_vs_amnestic_AD | num_final_pauses | 0,004 | 0,24 | small |
| Pathotype | FTLD_vs_lvPPA_FTLD | median_phoneme_duration | 0,004 | 0,73 | large |
| Pathotype | svPPA_FTLD_vs_lvPPA_FTLD | min_phoneme_duration | 0,004 | 0,84 | large |
| Pathotype | lvPPA_FTLD_vs_lvPPA_AD | spectral_instability | 0,004 | 0,77 | large |
| Pathotype | lvPPA_FTLD_vs_lvPPA_AD | contrast_mean_2 | 0,004 | 0,77 | large |
| Pathotype | lvPPA_FTLD_vs_amnestic_AD | kurt_phoneme_duration | 0,004 | 0,70 | large |
| Pathotype | svPPA_FTLD_vs_healthy_control | hnr_std,1 | 0,004 | 0,55 | large |
| Pathotype | healthy_control_vs_lvPPA_AD | nasales_mean | 0,004 | 0,45 | medium |
| Pathotype | svPPA_FTLD_vs_nfvPPA_FTLD | pause_max_duration | 0,004 | 0,76 | large |
| Pathotype | svPPA_FTLD_vs_nfvPPA_FTLD | inter_word_pause_max | 0,004 | 0,76 | large |
| Pathotype | svPPA_FTLD_vs_nfvPPA_FTLD | voyelles_oral_percentile_90 | 0,004 | 0,76 | large |
| Pathotype | svPPA_FTLD_vs_nfvPPA_FTLD | fricatives_percentile_90 | 0,004 | 0,76 | large |
| Pathotype | svPPA_FTLD_vs_nfvPPA_FTLD | liquides_percentile_90 | 0,004 | 0,76 | large |
| Pathotype | svPPA_FTLD_vs_nfvPPA_FTLD | nasales_median | 0,004 | 0,76 | large |
| Pathotype | healthy_control_vs_amnestic_AD | voyelles_oral_median | 0,004 | 0,33 | medium |
| Pathotype | svPPA_FTLD_vs_nfvPPA_FTLD | pause_cv | 0,005 | 0,76 | large |
| Pathotype | svPPA_FTLD_vs_nfvPPA_FTLD | occlusives_mean | 0,005 | 0,76 | large |
| Pathotype | svPPA_FTLD_vs_nfvPPA_FTLD | occlusives_iqr | 0,005 | 0,76 | large |
| Pathotype | nfvPPA_FTLD_vs_healthy_control | fricatives_percentile_90 | 0,005 | 0,54 | large |
| Pathotype | svPPA_FTLD_vs_amnestic_AD | chroma_mean_8 | 0,005 | 0,60 | large |
| Pathotype | svPPA_FTLD_vs_amnestic_AD | spectral_flux_std | 0,005 | 0,60 | large |
| Pathotype | svPPA_FTLD_vs_amnestic_AD | intensity_slope | 0,005 | 0,60 | large |
| Pathotype | svPPA_FTLD_vs_lvPPA_AD | occlusives_median | 0,005 | 0,68 | large |
| Pathotype | nfvPPA_FTLD_vs_lvPPA_AD | contrast_mean_4 | 0,005 | 0,68 | large |
| Pathotype | nfvPPA_FTLD_vs_lvPPA_AD | liquides_median | 0,005 | 0,68 | large |
| Pathotype | nfvPPA_FTLD_vs_lvPPA_FTLD | long_pause_count | 0,005 | 0,83 | large |
| Pathotype | FTLD_vs_healthy_control | liquides_cv | 0,005 | 0,40 | medium |
| Pathotype | svPPA_FTLD_vs_lvPPA_AD | nasales_skewness | 0,005 | 0,68 | large |
| Pathotype | svPPA_FTLD_vs_healthy_control | spectral_flux_std | 0,005 | 0,54 | large |
| Pathotype | amnestic_AD_vs_lvPPA_AD | occlusives_min | 0,005 | 0,48 | medium |
| Pathotype | healthy_control_vs_lvPPA_AD | contrast_mean_6 | 0,005 | 0,45 | medium |
| Pathotype | svPPA_FTLD_vs_amnestic_AD | long_pause_count | 0,005 | 0,59 | large |
| Pathotype | FTLD_vs_lvPPA_FTLD | delta_mfcc_mean_11 | 0,005 | 0,73 | large |
| Pathotype | FTLD_vs_lvPPA_FTLD | chroma_mean_9 | 0,005 | 0,73 | large |
| Pathotype | healthy_control_vs_lvPPA_AD | fricatives_percentile_90 | 0,005 | 0,44 | medium |
| Pathotype | nfvPPA_FTLD_vs_healthy_control | pause_std_duration | 0,005 | 0,54 | large |
| Pathotype | nfvPPA_FTLD_vs_healthy_control | inter_word_pause_std | 0,005 | 0,54 | large |
| Pathotype | svPPA_FTLD_vs_FTLD | max_phoneme_duration | 0,005 | 0,64 | large |
| Pathotype | FTLD_vs_healthy_control | pause_std_duration | 0,005 | 0,40 | medium |
| Pathotype | FTLD_vs_healthy_control | inter_word_pause_std | 0,005 | 0,40 | medium |
| Pathotype | FTLD_vs_lvPPA_FTLD | std_phoneme_duration | 0,005 | 0,73 | large |
| Pathotype | lvPPA_FTLD_vs_amnestic_AD | fricatives_percentile_90 | 0,005 | 0,69 | large |
| Pathotype | lvPPA_FTLD_vs_lvPPA_AD | semi_voyelles_count | 0,005 | 0,75 | large |
| Pathotype | svPPA_FTLD_vs_FTLD | nasales_mean | 0,005 | 0,64 | large |
| Pathotype | FTLD_vs_healthy_control | delta_mfcc_mean_12 | 0,005 | 0,40 | medium |
| Pathotype | FTLD_vs_healthy_control | nasales_skewness | 0,005 | 0,40 | medium |
| Pathotype | lvPPA_FTLD_vs_amnestic_AD | delta_mfcc_mean_10 | 0,005 | 0,69 | large |
| Pathotype | lvPPA_FTLD_vs_amnestic_AD | chroma_mean_6 | 0,005 | 0,69 | large |
| Pathotype | lvPPA_FTLD_vs_amnestic_AD | f2_cv,1 | 0,005 | 0,69 | large |
| Pathotype | FTLD_vs_lvPPA_AD | nasales_mean | 0,005 | 0,55 | large |
| Pathotype | svPPA_FTLD_vs_lvPPA_FTLD | occlusives_kurtosis | 0,005 | 0,83 | large |
| Pathotype | svPPA_FTLD_vs_amnestic_AD | contrast_mean_3 | 0,005 | 0,59 | large |
| Pathotype | FTLD_vs_amnestic_AD | occlusives_count | 0,005 | 0,46 | medium |
| Pathotype | lvPPA_FTLD_vs_healthy_control | f1_cv,1 | 0,005 | 0,63 | large |
| Pathotype | svPPA_FTLD_vs_lvPPA_FTLD | total_duration | 0,005 | 0,83 | large |
| Pathotype | svPPA_FTLD_vs_lvPPA_FTLD | liquides_kurtosis | 0,005 | 0,80 | large |
| Pathotype | nfvPPA_FTLD_vs_lvPPA_FTLD | syllable_count | 0,005 | 0,83 | large |
| Pathotype | nfvPPA_FTLD_vs_lvPPA_FTLD | voyelles_oral_count | 0,005 | 0,83 | large |
| Pathotype | nfvPPA_FTLD_vs_lvPPA_FTLD | nasales_mean | 0,005 | 0,83 | large |
| Pathotype | nfvPPA_FTLD_vs_lvPPA_FTLD | nasales_median | 0,005 | 0,83 | large |
| Pathotype | nfvPPA_FTLD_vs_lvPPA_FTLD | nasales_max | 0,005 | 0,83 | large |
| Pathotype | nfvPPA_FTLD_vs_lvPPA_FTLD | nasales_percentile_90 | 0,005 | 0,83 | large |
| Pathotype | svPPA_FTLD_vs_lvPPA_FTLD | occlusives_cv | 0,005 | 0,83 | large |
| Pathotype | svPPA_FTLD_vs_lvPPA_FTLD | occlusives_skewness | 0,005 | 0,83 | large |
| Pathotype | svPPA_FTLD_vs_FTLD | semi_voyelles_mean | 0,005 | 0,63 | large |
| Pathotype | lvPPA_FTLD_vs_lvPPA_AD | f0_max | 0,005 | 0,75 | large |
| Pathotype | lvPPA_FTLD_vs_lvPPA_AD | b2 | 0,005 | 0,75 | large |
| Pathotype | svPPA_FTLD_vs_healthy_control | speech_duration | 0,005 | 0,53 | large |
| Pathotype | nfvPPA_FTLD_vs_healthy_control | spectral_instability | 0,005 | 0,53 | large |
| Pathotype | FTLD_vs_lvPPA_AD | contrast_mean_2 | 0,005 | 0,54 | large |
| Pathotype | lvPPA_FTLD_vs_lvPPA_AD | voyelles_oral_median | 0,005 | 0,75 | large |
| Pathotype | lvPPA_FTLD_vs_lvPPA_AD | fricatives_std | 0,005 | 0,75 | large |
| Pathotype | lvPPA_FTLD_vs_lvPPA_AD | fricatives_cv | 0,005 | 0,75 | large |
| Pathotype | FTLD_vs_amnestic_AD | occlusives_kurtosis | 0,005 | 0,46 | medium |
| Pathotype | amnestic_AD_vs_lvPPA_AD | intensity_dynamic_range | 0,006 | 0,50 | medium |
| Pathotype | lvPPA_FTLD_vs_amnestic_AD | chroma_mean_0 | 0,006 | 0,68 | large |
| Pathotype | lvPPA_FTLD_vs_amnestic_AD | chroma_mean_1 | 0,006 | 0,68 | large |
| Pathotype | lvPPA_FTLD_vs_amnestic_AD | rolloff_std | 0,006 | 0,68 | large |
| Pathotype | svPPA_FTLD_vs_FTLD | delta_mfcc_mean_9 | 0,006 | 0,63 | large |
| Pathotype | lvPPA_FTLD_vs_amnestic_AD | cv_phoneme_duration | 0,006 | 0,68 | large |
| Pathotype | nfvPPA_FTLD_vs_FTLD | hnr_mean | 0,006 | 0,63 | large |
| Pathotype | svPPA_FTLD_vs_amnestic_AD | shimmer_dda | 0,006 | 0,59 | large |
| Pathotype | nfvPPA_FTLD_vs_amnestic_AD | speech_duration | 0,006 | 0,59 | large |
| Pathotype | svPPA_FTLD_vs_lvPPA_AD | occlusives_percentile_90 | 0,006 | 0,66 | large |
| Pathotype | nfvPPA_FTLD_vs_FTLD | hnr_mean,1 | 0,006 | 0,63 | large |
| Pathotype | svPPA_FTLD_vs_FTLD | mean_phoneme_duration | 0,006 | 0,63 | large |
| Pathotype | nfvPPA_FTLD_vs_amnestic_AD | liquides_percentile_90 | 0,006 | 0,59 | large |
| Pathotype | FTLD_vs_lvPPA_FTLD | delta_mfcc_mean_3 | 0,006 | 0,71 | large |
| Pathotype | FTLD_vs_lvPPA_FTLD | chroma_mean_2 | 0,006 | 0,71 | large |
| Pathotype | FTLD_vs_amnestic_AD | semi_voyelles_count | 0,006 | 0,45 | medium |
| Pathotype | FTLD_vs_lvPPA_FTLD | chroma_mean_4 | 0,006 | 0,71 | large |
| Pathotype | FTLD_vs_amnestic_AD | phoneme_count | 0,006 | 0,45 | medium |
| Pathotype | FTLD_vs_amnestic_AD | num_speech_phonemes | 0,006 | 0,45 | medium |
| Pathotype | FTLD_vs_lvPPA_FTLD | zcr_mean | 0,006 | 0,71 | large |
| Pathotype | FTLD_vs_lvPPA_FTLD | chroma_mean_3 | 0,006 | 0,71 | large |
| Pathotype | FTLD_vs_lvPPA_FTLD | chroma_mean_6 | 0,006 | 0,71 | large |
| Pathotype | FTLD_vs_lvPPA_FTLD | spectral_centroid_std | 0,006 | 0,71 | large |
| Pathotype | nfvPPA_FTLD_vs_FTLD | semi_voyelles_std | 0,006 | 0,62 | large |
| Pathotype | nfvPPA_FTLD_vs_FTLD | semi_voyelles_cv | 0,006 | 0,62 | large |
| Pathotype | FTLD_vs_amnestic_AD | articulation_rate | 0,006 | 0,44 | medium |
| Pathotype | FTLD_vs_lvPPA_FTLD | pause_mean_duration | 0,006 | 0,71 | large |
| Pathotype | FTLD_vs_lvPPA_FTLD | inter_word_pause_mean | 0,006 | 0,71 | large |
| Pathotype | FTLD_vs_lvPPA_FTLD | pause_frequency | 0,006 | 0,71 | large |
| Pathotype | FTLD_vs_lvPPA_FTLD | pause_median_duration | 0,006 | 0,71 | large |
| Pathotype | FTLD_vs_lvPPA_FTLD | inter_word_pause_median | 0,006 | 0,71 | large |
| Pathotype | lvPPA_FTLD_vs_lvPPA_AD | semi_voyelles_median | 0,006 | 0,74 | large |
| Pathotype | FTLD_vs_lvPPA_FTLD | semi_voyelles_skewness | 0,006 | 0,67 | large |
| Pathotype | svPPA_FTLD_vs_healthy_control | semi_voyelles_cv | 0,006 | 0,53 | large |
| Pathotype | nfvPPA_FTLD_vs_healthy_control | fricatives_std | 0,006 | 0,53 | large |
| Pathotype | nfvPPA_FTLD_vs_healthy_control | pause_max_duration | 0,006 | 0,53 | large |
| Pathotype | nfvPPA_FTLD_vs_healthy_control | inter_word_pause_max | 0,006 | 0,53 | large |
| Pathotype | nfvPPA_FTLD_vs_healthy_control | voyelles_oral_cv | 0,006 | 0,53 | large |
| Pathotype | FTLD_vs_amnestic_AD | num_inter_pauses | 0,006 | 0,45 | medium |
| Pathotype | lvPPA_FTLD_vs_lvPPA_AD | median_phoneme_duration | 0,006 | 0,74 | large |
| Pathotype | lvPPA_FTLD_vs_lvPPA_AD | semi_voyelles_skewness | 0,006 | 0,69 | large |
| Pathotype | lvPPA_FTLD_vs_healthy_control | semi_voyelles_kurtosis | 0,006 | 0,62 | large |
| Pathotype | nfvPPA_FTLD_vs_healthy_control | delta_mfcc_mean_2 | 0,006 | 0,53 | large |
| Pathotype | lvPPA_FTLD_vs_amnestic_AD | semi_voyelles_percentile_10 | 0,006 | 0,67 | large |
| Pathotype | nfvPPA_FTLD_vs_FTLD | chroma_mean_1 | 0,006 | 0,62 | large |
| Pathotype | svPPA_FTLD_vs_FTLD | intensity_std | 0,006 | 0,62 | large |
| Pathotype | lvPPA_FTLD_vs_healthy_control | semi_voyelles_percentile_10 | 0,006 | 0,59 | large |
| Pathotype | svPPA_FTLD_vs_FTLD | delta_mfcc_mean_6 | 0,006 | 0,62 | large |
| Pathotype | svPPA_FTLD_vs_FTLD | nasales_iqr | 0,006 | 0,62 | large |
| Pathotype | nfvPPA_FTLD_vs_lvPPA_AD | semi_voyelles_percentile_90 | 0,006 | 0,65 | large |
| Pathotype | FTLD_vs_lvPPA_FTLD | fricatives_skewness | 0,006 | 0,70 | large |
| Pathotype | svPPA_FTLD_vs_lvPPA_AD | liquides_cv | 0,007 | 0,65 | large |
| Pathotype | nfvPPA_FTLD_vs_lvPPA_AD | pause_mean_duration | 0,007 | 0,65 | large |
| Pathotype | nfvPPA_FTLD_vs_lvPPA_AD | inter_word_pause_mean | 0,007 | 0,65 | large |
| Pathotype | FTLD_vs_healthy_control | chroma_mean_4 | 0,007 | 0,39 | medium |
| Pathotype | svPPA_FTLD_vs_healthy_control | phoneme_count | 0,007 | 0,52 | large |
| Pathotype | svPPA_FTLD_vs_healthy_control | num_speech_phonemes | 0,007 | 0,52 | large |
| Pathotype | svPPA_FTLD_vs_healthy_control | pause_std_duration | 0,007 | 0,52 | large |
| Pathotype | svPPA_FTLD_vs_healthy_control | inter_word_pause_std | 0,007 | 0,52 | large |
| Pathotype | svPPA_FTLD_vs_healthy_control | pause_ratio | 0,007 | 0,52 | large |
| Pathotype | FTLD_vs_lvPPA_FTLD | f1 | 0,007 | 0,70 | large |
| Pathotype | lvPPA_FTLD_vs_lvPPA_AD | shimmer_local | 0,007 | 0,73 | large |
| Pathotype | lvPPA_FTLD_vs_lvPPA_AD | shimmer_apq5 | 0,007 | 0,73 | large |
| Pathotype | lvPPA_FTLD_vs_lvPPA_AD | spectral_centroid_slope | 0,007 | 0,73 | large |
| Pathotype | lvPPA_FTLD_vs_lvPPA_AD | spectral_flux,1 | 0,007 | 0,73 | large |
| Pathotype | FTLD_vs_lvPPA_FTLD | f0_max | 0,007 | 0,70 | large |
| Pathotype | FTLD_vs_amnestic_AD | num_pauses | 0,007 | 0,44 | medium |
| Pathotype | FTLD_vs_amnestic_AD | num_inter_word_pauses | 0,007 | 0,44 | medium |
| Pathotype | nfvPPA_FTLD_vs_amnestic_AD | voyelles_oral_kurtosis | 0,007 | 0,58 | large |
| Pathotype | lvPPA_FTLD_vs_amnestic_AD | pause_median_duration | 0,007 | 0,66 | large |
| Pathotype | lvPPA_FTLD_vs_amnestic_AD | inter_word_pause_median | 0,007 | 0,66 | large |
| Pathotype | FTLD_vs_lvPPA_AD | f0_min | 0,007 | 0,38 | medium |
| Pathotype | lvPPA_FTLD_vs_amnestic_AD | b1 | 0,007 | 0,66 | large |
| Pathotype | FTLD_vs_lvPPA_AD | articulation_rate | 0,007 | 0,38 | medium |
| Pathotype | nfvPPA_FTLD_vs_lvPPA_FTLD | contrast_mean_2 | 0,007 | 0,80 | large |
| Pathotype | nfvPPA_FTLD_vs_lvPPA_FTLD | contrast_mean_4 | 0,007 | 0,80 | large |
| Pathotype | nfvPPA_FTLD_vs_lvPPA_FTLD | rmse_mean | 0,007 | 0,80 | large |
| Pathotype | nfvPPA_FTLD_vs_lvPPA_FTLD | spectral_flux_std | 0,007 | 0,80 | large |
| Pathotype | nfvPPA_FTLD_vs_lvPPA_FTLD | intensity_mean | 0,007 | 0,80 | large |
| Pathotype | nfvPPA_FTLD_vs_lvPPA_FTLD | hnr_std,1 | 0,007 | 0,80 | large |
| Pathotype | nfvPPA_FTLD_vs_lvPPA_FTLD | nasales_count | 0,007 | 0,80 | large |
| Pathotype | lvPPA_FTLD_vs_amnestic_AD | occlusives_skewness | 0,007 | 0,66 | large |
| Pathotype | svPPA_FTLD_vs_lvPPA_FTLD | spectral_centroid_mean | 0,007 | 0,80 | large |
| Pathotype | svPPA_FTLD_vs_lvPPA_FTLD | f0_std | 0,007 | 0,80 | large |
| Pathotype | svPPA_FTLD_vs_lvPPA_FTLD | f1_cv,1 | 0,007 | 0,80 | large |
| Pathotype | svPPA_FTLD_vs_lvPPA_FTLD | f2_cv,1 | 0,007 | 0,80 | large |
| Pathotype | svPPA_FTLD_vs_lvPPA_FTLD | f3_cv,1 | 0,007 | 0,80 | large |
| Pathotype | nfvPPA_FTLD_vs_lvPPA_FTLD | f3_cv | 0,007 | 0,80 | large |
| Pathotype | nfvPPA_FTLD_vs_lvPPA_FTLD | hnr_mean | 0,007 | 0,80 | large |
| Pathotype | nfvPPA_FTLD_vs_lvPPA_FTLD | delta_mfcc_mean_8 | 0,007 | 0,80 | large |
| Pathotype | nfvPPA_FTLD_vs_lvPPA_FTLD | chroma_mean_8 | 0,007 | 0,80 | large |
| Pathotype | nfvPPA_FTLD_vs_lvPPA_FTLD | spectral_flux_mean | 0,007 | 0,80 | large |
| Pathotype | nfvPPA_FTLD_vs_lvPPA_FTLD | f0_max | 0,007 | 0,80 | large |
| Pathotype | nfvPPA_FTLD_vs_lvPPA_FTLD | f3_cv,1 | 0,007 | 0,80 | large |
| Pathotype | nfvPPA_FTLD_vs_lvPPA_FTLD | hnr_mean,1 | 0,007 | 0,80 | large |
| Pathotype | nfvPPA_FTLD_vs_lvPPA_FTLD | num_pauses | 0,007 | 0,80 | large |
| Pathotype | nfvPPA_FTLD_vs_lvPPA_FTLD | num_inter_word_pauses | 0,007 | 0,80 | large |
| Pathotype | nfvPPA_FTLD_vs_lvPPA_FTLD | std_phoneme_duration | 0,007 | 0,80 | large |
| Pathotype | nfvPPA_FTLD_vs_lvPPA_FTLD | max_phoneme_duration | 0,007 | 0,80 | large |
| Pathotype | nfvPPA_FTLD_vs_lvPPA_FTLD | occlusives_count | 0,007 | 0,80 | large |
| Pathotype | nfvPPA_FTLD_vs_lvPPA_FTLD | occlusives_max | 0,007 | 0,80 | large |
| Pathotype | nfvPPA_FTLD_vs_lvPPA_FTLD | liquides_max | 0,007 | 0,80 | large |
| Pathotype | nfvPPA_FTLD_vs_healthy_control | intensity_dynamic_range | 0,007 | 0,52 | large |
| Pathotype | svPPA_FTLD_vs_healthy_control | f0_std | 0,007 | 0,52 | large |
| Pathotype | svPPA_FTLD_vs_nfvPPA_FTLD | delta_mfcc_mean_2 | 0,007 | 0,72 | large |
| Pathotype | svPPA_FTLD_vs_nfvPPA_FTLD | liquides_cv | 0,007 | 0,72 | large |
| Pathotype | svPPA_FTLD_vs_lvPPA_AD | semi_voyelles_mean | 0,007 | 0,64 | large |
| Pathotype | nfvPPA_FTLD_vs_lvPPA_FTLD | phoneme_count | 0,007 | 0,80 | large |
| Pathotype | nfvPPA_FTLD_vs_lvPPA_FTLD | num_speech_phonemes | 0,007 | 0,80 | large |
| Pathotype | nfvPPA_FTLD_vs_lvPPA_FTLD | mean_phoneme_duration | 0,007 | 0,80 | large |
| Pathotype | nfvPPA_FTLD_vs_lvPPA_FTLD | median_phoneme_duration | 0,007 | 0,80 | large |
| Pathotype | nfvPPA_FTLD_vs_lvPPA_FTLD | pause_cv | 0,007 | 0,80 | large |
| Pathotype | nfvPPA_FTLD_vs_lvPPA_FTLD | occlusives_mean | 0,007 | 0,80 | large |
| Pathotype | nfvPPA_FTLD_vs_lvPPA_FTLD | occlusives_median | 0,007 | 0,80 | large |
| Pathotype | nfvPPA_FTLD_vs_lvPPA_FTLD | occlusives_std | 0,007 | 0,80 | large |
| Pathotype | nfvPPA_FTLD_vs_lvPPA_FTLD | occlusives_percentile_90 | 0,007 | 0,80 | large |
| Pathotype | nfvPPA_FTLD_vs_lvPPA_FTLD | occlusives_iqr | 0,007 | 0,80 | large |
| Pathotype | nfvPPA_FTLD_vs_lvPPA_FTLD | liquides_mean | 0,007 | 0,80 | large |
| Pathotype | nfvPPA_FTLD_vs_lvPPA_FTLD | liquides_median | 0,007 | 0,80 | large |
| Pathotype | nfvPPA_FTLD_vs_lvPPA_FTLD | liquides_percentile_10 | 0,007 | 0,80 | large |
| Pathotype | nfvPPA_FTLD_vs_lvPPA_FTLD | liquides_percentile_90 | 0,007 | 0,80 | large |
| Pathotype | svPPA_FTLD_vs_FTLD | chroma_mean_3 | 0,007 | 0,61 | large |
| Pathotype | svPPA_FTLD_vs_nfvPPA_FTLD | pause_std_duration | 0,007 | 0,72 | large |
| Pathotype | svPPA_FTLD_vs_nfvPPA_FTLD | inter_word_pause_std | 0,007 | 0,72 | large |
| Pathotype | FTLD_vs_lvPPA_AD | chroma_mean_2 | 0,007 | 0,52 | large |
| Pathotype | nfvPPA_FTLD_vs_healthy_control | contrast_mean_2 | 0,007 | 0,52 | large |
| Pathotype | lvPPA_FTLD_vs_healthy_control | fricatives_mean | 0,007 | 0,60 | large |
| Pathotype | svPPA_FTLD_vs_lvPPA_FTLD | pause_min_duration | 0,007 | 0,80 | large |
| Pathotype | svPPA_FTLD_vs_lvPPA_FTLD | inter_word_pause_min | 0,007 | 0,80 | large |
| Pathotype | svPPA_FTLD_vs_lvPPA_FTLD | nasales_mean | 0,007 | 0,80 | large |
| Pathotype | lvPPA_FTLD_vs_lvPPA_AD | occlusives_min | 0,007 | 0,71 | large |
| Pathotype | nfvPPA_FTLD_vs_FTLD | liquides_skewness | 0,007 | 0,61 | large |
| Pathotype | svPPA_FTLD_vs_FTLD | cv_phoneme_duration | 0,007 | 0,61 | large |
| Pathotype | FTLD_vs_lvPPA_AD | pause_ratio | 0,007 | 0,52 | large |
| Pathotype | nfvPPA_FTLD_vs_amnestic_AD | spectral_instability | 0,007 | 0,57 | large |
| Pathotype | lvPPA_FTLD_vs_lvPPA_AD | articulation_rate | 0,007 | 0,43 | medium |
| Pathotype | svPPA_FTLD_vs_amnestic_AD | liquides_kurtosis | 0,007 | 0,57 | large |
| Pathotype | svPPA_FTLD_vs_amnestic_AD | contrast_mean_1 | 0,007 | 0,57 | large |
| Pathotype | svPPA_FTLD_vs_amnestic_AD | voyelles_oral_mean | 0,007 | 0,57 | large |
| Pathotype | lvPPA_FTLD_vs_lvPPA_AD | occlusives_median | 0,007 | 0,72 | large |
| Pathotype | lvPPA_FTLD_vs_lvPPA_AD | nasales_mean | 0,007 | 0,72 | large |
| Pathotype | healthy_control_vs_lvPPA_AD | fricatives_iqr | 0,007 | 0,42 | medium |
| Pathotype | amnestic_AD_vs_lvPPA_AD | delta_mfcc_mean_1 | 0,008 | 0,48 | medium |
| Pathotype | FTLD_vs_amnestic_AD | hnr_std | 0,008 | 0,44 | medium |
| Pathotype | lvPPA_FTLD_vs_healthy_control | chroma_mean_0 | 0,008 | 0,60 | large |
| Pathotype | nfvPPA_FTLD_vs_lvPPA_AD | spectral_flux_std | 0,008 | 0,64 | large |
| Pathotype | svPPA_FTLD_vs_lvPPA_AD | chroma_mean_7 | 0,008 | 0,64 | large |
| Pathotype | nfvPPA_FTLD_vs_healthy_control | liquides_kurtosis | 0,008 | 0,51 | large |
| Pathotype | nfvPPA_FTLD_vs_lvPPA_AD | delta_mfcc_mean_8 | 0,008 | 0,64 | large |
| Pathotype | amnestic_AD_vs_lvPPA_AD | median_phoneme_duration | 0,008 | 0,46 | medium |
| Pathotype | healthy_control_vs_amnestic_AD | liquides_kurtosis | 0,008 | 0,32 | medium |
| Pathotype | FTLD_vs_lvPPA_FTLD | delta_mfcc_mean_2 | 0,008 | 0,69 | large |
| Pathotype | svPPA_FTLD_vs_amnestic_AD | semi_voyelles_percentile_90 | 0,008 | 0,56 | large |
| Pathotype | FTLD_vs_lvPPA_FTLD | f1_cv | 0,008 | 0,69 | large |
| Pathotype | FTLD_vs_lvPPA_FTLD | contrast_mean_1 | 0,008 | 0,69 | large |
| Pathotype | FTLD_vs_lvPPA_FTLD | rolloff_std | 0,008 | 0,69 | large |
| Pathotype | FTLD_vs_lvPPA_FTLD | f0_slope | 0,008 | 0,69 | large |
| Pathotype | svPPA_FTLD_vs_amnestic_AD | delta_mfcc_mean_11 | 0,008 | 0,56 | large |
| Pathotype | lvPPA_FTLD_vs_healthy_control | hnr_std | 0,008 | 0,60 | large |
| Pathotype | svPPA_FTLD_vs_amnestic_AD | liquides_percentile_90 | 0,008 | 0,56 | large |
| Pathotype | nfvPPA_FTLD_vs_amnestic_AD | voyelles_oral_std | 0,008 | 0,56 | large |
| Pathotype | svPPA_FTLD_vs_amnestic_AD | mean_phoneme_duration | 0,008 | 0,56 | large |
| Pathotype | healthy_control_vs_amnestic_AD | chroma_mean_6 | 0,008 | 0,32 | medium |
| Pathotype | svPPA_FTLD_vs_FTLD | occlusives_max | 0,008 | 0,60 | large |
| Pathotype | FTLD_vs_amnestic_AD | fricatives_count | 0,008 | 0,43 | medium |
| Pathotype | lvPPA_FTLD_vs_lvPPA_AD | shimmer_apq3 | 0,008 | 0,71 | large |
| Pathotype | lvPPA_FTLD_vs_lvPPA_AD | shimmer_dda | 0,008 | 0,71 | large |
| Pathotype | lvPPA_FTLD_vs_lvPPA_AD | delta_mfcc_mean_2 | 0,008 | 0,71 | large |
| Pathotype | lvPPA_FTLD_vs_amnestic_AD | nasales_median | 0,008 | 0,65 | large |
| Pathotype | svPPA_FTLD_vs_FTLD | chroma_mean_4 | 0,008 | 0,60 | large |
| Pathotype | svPPA_FTLD_vs_FTLD | contrast_mean_1 | 0,008 | 0,60 | large |
| Pathotype | nfvPPA_FTLD_vs_FTLD | delta_mfcc_mean_12 | 0,008 | 0,60 | large |
| Pathotype | lvPPA_FTLD_vs_lvPPA_AD | pause_median_duration | 0,008 | 0,71 | large |
| Pathotype | lvPPA_FTLD_vs_lvPPA_AD | inter_word_pause_median | 0,008 | 0,71 | large |
| Pathotype | nfvPPA_FTLD_vs_FTLD | voyelles_oral_kurtosis | 0,008 | 0,60 | large |
| Pathotype | svPPA_FTLD_vs_FTLD | pause_median_duration | 0,008 | 0,60 | large |
| Pathotype | svPPA_FTLD_vs_FTLD | inter_word_pause_median | 0,008 | 0,60 | large |
| Pathotype | nfvPPA_FTLD_vs_amnestic_AD | semi_voyelles_median | 0,008 | 0,56 | large |
| Pathotype | svPPA_FTLD_vs_healthy_control | long_pause_count | 0,008 | 0,50 | large |
| Pathotype | amnestic_AD_vs_lvPPA_AD | delta_mfcc_mean_7 | 0,008 | 0,47 | medium |
| Pathotype | FTLD_vs_lvPPA_FTLD | f0_min | 0,008 | 0,64 | large |
| Pathotype | nfvPPA_FTLD_vs_healthy_control | spectral_centroid_mean | 0,008 | 0,51 | large |
| Pathotype | healthy_control_vs_lvPPA_AD | liquides_kurtosis | 0,008 | 0,42 | medium |
| Pathotype | healthy_control_vs_lvPPA_AD | liquides_mean | 0,008 | 0,42 | medium |
| Pathotype | FTLD_vs_amnestic_AD | delta_mfcc_mean_2 | 0,008 | 0,43 | medium |
| Pathotype | healthy_control_vs_amnestic_AD | b3 | 0,009 | 0,32 | medium |
| Pathotype | svPPA_FTLD_vs_healthy_control | pause_max_duration | 0,009 | 0,51 | large |
| Pathotype | svPPA_FTLD_vs_healthy_control | inter_word_pause_max | 0,009 | 0,51 | large |
| Pathotype | lvPPA_FTLD_vs_amnestic_AD | skew_phoneme_duration | 0,009 | 0,64 | large |
| Pathotype | nfvPPA_FTLD_vs_amnestic_AD | voyelles_oral_median | 0,009 | 0,56 | large |
| Pathotype | lvPPA_FTLD_vs_amnestic_AD | f0_max | 0,009 | 0,64 | large |
| Pathotype | lvPPA_FTLD_vs_amnestic_AD | b3 | 0,009 | 0,64 | large |
| Pathotype | lvPPA_FTLD_vs_amnestic_AD | pause_ratio | 0,009 | 0,64 | large |
| Pathotype | nfvPPA_FTLD_vs_amnestic_AD | intensity_dynamic_range | 0,009 | 0,56 | large |
| Pathotype | healthy_control_vs_amnestic_AD | fricatives_percentile_90 | 0,009 | 0,32 | medium |
| Pathotype | nfvPPA_FTLD_vs_healthy_control | delta_mfcc_mean_9 | 0,009 | 0,50 | large |
| Pathotype | FTLD_vs_lvPPA_AD | pause_median_duration | 0,009 | 0,51 | large |
| Pathotype | FTLD_vs_lvPPA_AD | inter_word_pause_median | 0,009 | 0,51 | large |
| Pathotype | svPPA_FTLD_vs_healthy_control | f0_max | 0,009 | 0,50 | large |
| Pathotype | svPPA_FTLD_vs_amnestic_AD | contrast_mean_4 | 0,009 | 0,56 | large |
| Pathotype | svPPA_FTLD_vs_lvPPA_AD | liquides_count | 0,009 | 0,63 | large |
| Pathotype | nfvPPA_FTLD_vs_amnestic_AD | mean_phoneme_duration | 0,009 | 0,56 | large |
| Pathotype | svPPA_FTLD_vs_amnestic_AD | pause_total_duration | 0,009 | 0,56 | large |
| Pathotype | FTLD_vs_amnestic_AD | shimmer_apq5 | 0,009 | 0,43 | medium |
| Pathotype | nfvPPA_FTLD_vs_lvPPA_AD | contrast_mean_2 | 0,009 | 0,63 | large |
| Pathotype | svPPA_FTLD_vs_lvPPA_AD | occlusives_percentile_10 | 0,009 | 0,61 | large |
| Pathotype | svPPA_FTLD_vs_nfvPPA_FTLD | spectral_centroid_slope | 0,009 | 0,70 | large |
| Pathotype | svPPA_FTLD_vs_nfvPPA_FTLD | fricatives_max | 0,009 | 0,70 | large |
| Pathotype | nfvPPA_FTLD_vs_lvPPA_AD | f0_max | 0,009 | 0,63 | large |
| Pathotype | FTLD_vs_lvPPA_FTLD | pause_min_duration | 0,009 | 0,67 | large |
| Pathotype | FTLD_vs_lvPPA_FTLD | inter_word_pause_min | 0,009 | 0,67 | large |
| Pathotype | nfvPPA_FTLD_vs_healthy_control | fricatives_cv | 0,009 | 0,50 | large |
| Pathotype | FTLD_vs_healthy_control | f3 | 0,009 | 0,37 | medium |
| Pathotype | svPPA_FTLD_vs_FTLD | pause_max_duration | 0,009 | 0,59 | large |
| Pathotype | svPPA_FTLD_vs_FTLD | inter_word_pause_max | 0,009 | 0,59 | large |
| Pathotype | FTLD_vs_lvPPA_FTLD | voyelles_oral_median | 0,009 | 0,67 | large |
| Pathotype | svPPA_FTLD_vs_FTLD | contrast_mean_2 | 0,009 | 0,59 | large |
| Pathotype | nfvPPA_FTLD_vs_FTLD | fricatives_std | 0,009 | 0,59 | large |
| Pathotype | FTLD_vs_lvPPA_FTLD | fricatives_iqr | 0,009 | 0,67 | large |
| Pathotype | healthy_control_vs_lvPPA_AD | fricatives_std | 0,009 | 0,41 | medium |
| Pathotype | svPPA_FTLD_vs_FTLD | chroma_mean_10 | 0,009 | 0,59 | large |
| Pathotype | nfvPPA_FTLD_vs_FTLD | delta_mfcc_mean_4 | 0,009 | 0,59 | large |
| Pathotype | nfvPPA_FTLD_vs_FTLD | f2 | 0,009 | 0,59 | large |
| Pathotype | svPPA_FTLD_vs_FTLD | delta_mfcc_mean_8 | 0,009 | 0,59 | large |
| Pathotype | svPPA_FTLD_vs_FTLD | chroma_mean_6 | 0,009 | 0,59 | large |
| Pathotype | svPPA_FTLD_vs_FTLD | rolloff_std | 0,009 | 0,59 | large |
| Pathotype | nfvPPA_FTLD_vs_amnestic_AD | phoneme_count | 0,009 | 0,55 | large |
| Pathotype | nfvPPA_FTLD_vs_amnestic_AD | num_speech_phonemes | 0,009 | 0,55 | large |
| Pathotype | FTLD_vs_lvPPA_FTLD | voyelles_oral_cv | 0,009 | 0,67 | large |
| Pathotype | FTLD_vs_lvPPA_FTLD | cv_phoneme_duration | 0,009 | 0,67 | large |
| Pathotype | nfvPPA_FTLD_vs_lvPPA_FTLD | liquides_count | 0,009 | 0,77 | large |
| Pathotype | FTLD_vs_lvPPA_FTLD | pause_ratio | 0,009 | 0,67 | large |
| Pathotype | amnestic_AD_vs_lvPPA_AD | chroma_mean_5 | 0,009 | 0,46 | medium |
| Pathotype | healthy_control_vs_amnestic_AD | fricatives_cv | 0,009 | 0,32 | medium |
| Pathotype | svPPA_FTLD_vs_FTLD | skew_phoneme_duration | 0,009 | 0,59 | large |
| Pathotype | amnestic_AD_vs_lvPPA_AD | occlusives_iqr | 0,009 | 0,46 | medium |
| Pathotype | svPPA_FTLD_vs_lvPPA_FTLD | delta_mfcc_mean_5 | 0,009 | 0,77 | large |
| Pathotype | svPPA_FTLD_vs_lvPPA_FTLD | contrast_mean_3 | 0,009 | 0,77 | large |
| Pathotype | FTLD_vs_amnestic_AD | syllable_count | 0,009 | 0,43 | medium |
| Pathotype | FTLD_vs_amnestic_AD | voyelles_oral_count | 0,009 | 0,43 | medium |
| Pathotype | lvPPA_FTLD_vs_amnestic_AD | delta_mfcc_mean_11 | 0,009 | 0,63 | large |
| Pathotype | nfvPPA_FTLD_vs_lvPPA_FTLD | delta_mfcc_mean_5 | 0,009 | 0,77 | large |
| Pathotype | nfvPPA_FTLD_vs_lvPPA_FTLD | fricatives_max | 0,009 | 0,77 | large |
| Pathotype | svPPA_FTLD_vs_healthy_control | occlusives_count | 0,009 | 0,50 | medium |
| Pathotype | svPPA_FTLD_vs_lvPPA_FTLD | fricatives_max | 0,009 | 0,77 | large |
| Pathotype | healthy_control_vs_lvPPA_AD | f1_cv,1 | 0,009 | 0,41 | medium |
| Pathotype | healthy_control_vs_lvPPA_AD | delta_mfcc_mean_8 | 0,009 | 0,41 | medium |
| Pathotype | lvPPA_FTLD_vs_amnestic_AD | f3_cv | 0,010 | 0,63 | large |
| Pathotype | nfvPPA_FTLD_vs_lvPPA_FTLD | voyelles_oral_median | 0,010 | 0,77 | large |
| Pathotype | nfvPPA_FTLD_vs_lvPPA_FTLD | fricatives_count | 0,010 | 0,77 | large |
| Pathotype | nfvPPA_FTLD_vs_lvPPA_FTLD | fricatives_std | 0,010 | 0,77 | large |
| Pathotype | lvPPA_FTLD_vs_amnestic_AD | pause_std_duration | 0,010 | 0,63 | large |
| Pathotype | lvPPA_FTLD_vs_amnestic_AD | inter_word_pause_std | 0,010 | 0,63 | large |
| Pathotype | lvPPA_FTLD_vs_lvPPA_AD | fricatives_iqr | 0,010 | 0,70 | large |
| Pathotype | svPPA_FTLD_vs_healthy_control | delta_mfcc_mean_1 | 0,010 | 0,50 | medium |
| Pathotype | svPPA_FTLD_vs_amnestic_AD | semi_voyelles_iqr | 0,010 | 0,55 | large |
| Pathotype | lvPPA_FTLD_vs_lvPPA_AD | contrast_mean_3 | 0,010 | 0,70 | large |
| Pathotype | FTLD_vs_amnestic_AD | shimmer_dda | 0,010 | 0,42 | medium |
| Pathotype | lvPPA_FTLD_vs_lvPPA_AD | occlusives_skewness | 0,010 | 0,70 | large |
| Pathotype | amnestic_AD_vs_lvPPA_AD | pause_max_duration | 0,010 | 0,46 | medium |
| Pathotype | amnestic_AD_vs_lvPPA_AD | inter_word_pause_max | 0,010 | 0,46 | medium |
| Pathotype | nfvPPA_FTLD_vs_FTLD | median_phoneme_duration | 0,010 | 0,58 | large |
| Pathotype | svPPA_FTLD_vs_healthy_control | intensity_slope | 0,010 | 0,49 | medium |
| Pathotype | FTLD_vs_healthy_control | fricatives_median | 0,010 | 0,36 | medium |
| Pathotype | nfvPPA_FTLD_vs_lvPPA_AD | semi_voyelles_mean | 0,010 | 0,61 | large |
| Pathotype | svPPA_FTLD_vs_healthy_control | voyelles_oral_percentile_10 | 0,010 | 0,44 | medium |
| Pathotype | nfvPPA_FTLD_vs_FTLD | semi_voyelles_mean | 0,010 | 0,58 | large |
| Pathotype | svPPA_FTLD_vs_amnestic_AD | std_phoneme_duration | 0,010 | 0,54 | large |
| Pathotype | svPPA_FTLD_vs_amnestic_AD | voyelles_oral_cv | 0,010 | 0,54 | large |
| Pathotype | svPPA_FTLD_vs_FTLD | spectral_flux_std | 0,010 | 0,58 | large |
| Pathotype | svPPA_FTLD_vs_lvPPA_AD | delta_mfcc_mean_6 | 0,010 | 0,61 | large |
| Pathotype | svPPA_FTLD_vs_lvPPA_AD | chroma_mean_4 | 0,010 | 0,61 | large |
| Pathotype | nfvPPA_FTLD_vs_lvPPA_AD | fricatives_max | 0,010 | 0,61 | large |
| Pathotype | nfvPPA_FTLD_vs_lvPPA_AD | delta_mfcc_mean_1 | 0,010 | 0,61 | large |
| Pathotype | svPPA_FTLD_vs_FTLD | jitter_local | 0,010 | 0,58 | large |
| Pathotype | nfvPPA_FTLD_vs_FTLD | spectral_instability | 0,010 | 0,58 | large |
| Pathotype | nfvPPA_FTLD_vs_FTLD | b2 | 0,010 | 0,58 | large |
| Pathotype | nfvPPA_FTLD_vs_FTLD | std_phoneme_duration | 0,010 | 0,58 | large |
| Pathotype | FTLD_vs_amnestic_AD | spectral_centroid_mean | 0,010 | 0,42 | medium |
| Pathotype | nfvPPA_FTLD_vs_FTLD | spectral_instability,1 | 0,011 | 0,58 | large |
| Pathotype | lvPPA_FTLD_vs_amnestic_AD | liquides_min | 0,011 | 0,62 | large |
| Pathotype | lvPPA_FTLD_vs_amnestic_AD | chroma_mean_7 | 0,011 | 0,63 | large |
| Pathotype | healthy_control_vs_lvPPA_AD | delta_mfcc_mean_4 | 0,011 | 0,40 | medium |
| Pathotype | lvPPA_FTLD_vs_healthy_control | f2_cv,1 | 0,011 | 0,57 | large |
| Pathotype | nfvPPA_FTLD_vs_lvPPA_AD | semi_voyelles_std | 0,011 | 0,60 | large |
| Pathotype | nfvPPA_FTLD_vs_lvPPA_AD | semi_voyelles_cv | 0,011 | 0,60 | large |
| Pathotype | nfvPPA_FTLD_vs_lvPPA_AD | semi_voyelles_iqr | 0,011 | 0,60 | large |
| Pathotype | FTLD_vs_amnestic_AD | contrast_mean_5 | 0,011 | 0,42 | medium |
| Pathotype | svPPA_FTLD_vs_nfvPPA_FTLD | chroma_mean_10 | 0,011 | 0,68 | large |
| Pathotype | svPPA_FTLD_vs_nfvPPA_FTLD | chroma_mean_11 | 0,011 | 0,68 | large |
| Pathotype | svPPA_FTLD_vs_nfvPPA_FTLD | std_phoneme_duration | 0,011 | 0,68 | large |
| Pathotype | lvPPA_FTLD_vs_healthy_control | voyelles_oral_percentile_90 | 0,011 | 0,57 | large |
| Pathotype | amnestic_AD_vs_lvPPA_AD | delta_mfcc_mean_11 | 0,011 | 0,45 | medium |
| Pathotype | FTLD_vs_healthy_control | contrast_mean_5 | 0,011 | 0,36 | medium |
| Pathotype | amnestic_AD_vs_lvPPA_AD | shimmer_local | 0,011 | 0,45 | medium |
| Pathotype | amnestic_AD_vs_lvPPA_AD | zcr_mean | 0,011 | 0,45 | medium |
| Pathotype | nfvPPA_FTLD_vs_healthy_control | long_pause_count | 0,011 | 0,48 | medium |
| Pathotype | svPPA_FTLD_vs_amnestic_AD | delta_mfcc_mean_9 | 0,011 | 0,54 | large |
| Pathotype | svPPA_FTLD_vs_amnestic_AD | voyelles_oral_skewness | 0,011 | 0,54 | large |
| Pathotype | healthy_control_vs_lvPPA_AD | liquides_percentile_10 | 0,011 | 0,39 | medium |
| Pathotype | nfvPPA_FTLD_vs_FTLD | semi_voyelles_max | 0,011 | 0,57 | large |
| Pathotype | FTLD_vs_lvPPA_AD | liquides_count | 0,012 | 0,49 | medium |
| Pathotype | FTLD_vs_amnestic_AD | speech_duration | 0,012 | 0,41 | medium |
| Pathotype | nfvPPA_FTLD_vs_lvPPA_FTLD | pause_min_duration | 0,012 | 0,74 | large |
| Pathotype | nfvPPA_FTLD_vs_lvPPA_FTLD | inter_word_pause_min | 0,012 | 0,74 | large |
| Pathotype | svPPA_FTLD_vs_FTLD | delta_mfcc_mean_2 | 0,012 | 0,57 | large |
| Pathotype | nfvPPA_FTLD_vs_amnestic_AD | num_final_pauses | 0,012 | 0,20 | small |
| Pathotype | svPPA_FTLD_vs_FTLD | nasales_kurtosis | 0,012 | 0,57 | large |
| Pathotype | nfvPPA_FTLD_vs_FTLD | voyelles_oral_median | 0,012 | 0,57 | large |
| Pathotype | lvPPA_FTLD_vs_healthy_control | spectral_flux,1 | 0,012 | 0,57 | large |
| Pathotype | nfvPPA_FTLD_vs_FTLD | spectral_flux | 0,012 | 0,57 | large |
| Pathotype | nfvPPA_FTLD_vs_FTLD | spectral_flux,1 | 0,012 | 0,57 | large |
| Pathotype | nfvPPA_FTLD_vs_FTLD | voyelles_oral_cv | 0,012 | 0,57 | large |
| Pathotype | svPPA_FTLD_vs_FTLD | nasales_skewness | 0,012 | 0,57 | large |
| Pathotype | svPPA_FTLD_vs_FTLD | voyelles_oral_skewness | 0,012 | 0,57 | large |
| Pathotype | FTLD_vs_healthy_control | semi_voyelles_percentile_10 | 0,012 | 0,35 | medium |
| Pathotype | lvPPA_FTLD_vs_lvPPA_AD | hnr_std | 0,012 | 0,68 | large |
| Pathotype | lvPPA_FTLD_vs_lvPPA_AD | delta_mfcc_mean_3 | 0,012 | 0,68 | large |
| Pathotype | lvPPA_FTLD_vs_lvPPA_AD | zcr_mean | 0,012 | 0,68 | large |
| Pathotype | lvPPA_FTLD_vs_lvPPA_AD | chroma_mean_3 | 0,012 | 0,68 | large |
| Pathotype | lvPPA_FTLD_vs_lvPPA_AD | fricatives_max | 0,012 | 0,68 | large |
| Pathotype | amnestic_AD_vs_lvPPA_AD | rolloff_mean | 0,012 | 0,45 | medium |
| Pathotype | svPPA_FTLD_vs_amnestic_AD | f2_cv | 0,012 | 0,53 | large |
| Pathotype | svPPA_FTLD_vs_lvPPA_AD | delta_mfcc_mean_4 | 0,012 | 0,60 | large |
| Pathotype | svPPA_FTLD_vs_lvPPA_AD | contrast_mean_0 | 0,012 | 0,60 | large |
| Pathotype | FTLD_vs_amnestic_AD | fricatives_cv | 0,012 | 0,41 | medium |
| Pathotype | svPPA_FTLD_vs_lvPPA_FTLD | f0_min | 0,012 | 0,74 | large |
| Pathotype | nfvPPA_FTLD_vs_lvPPA_FTLD | zcr_mean | 0,012 | 0,74 | large |
| Pathotype | nfvPPA_FTLD_vs_lvPPA_FTLD | contrast_mean_0 | 0,012 | 0,74 | large |
| Pathotype | nfvPPA_FTLD_vs_lvPPA_FTLD | f1 | 0,012 | 0,74 | large |
| Pathotype | nfvPPA_FTLD_vs_amnestic_AD | f0_slope | 0,012 | 0,53 | large |
| Pathotype | nfvPPA_FTLD_vs_amnestic_AD | liquides_iqr | 0,012 | 0,53 | large |
| Pathotype | FTLD_vs_lvPPA_AD | spectral_centroid_std | 0,012 | 0,49 | medium |
| Pathotype | nfvPPA_FTLD_vs_healthy_control | nasales_iqr | 0,012 | 0,48 | medium |
| Pathotype | svPPA_FTLD_vs_lvPPA_FTLD | jitter_ppq5 | 0,012 | 0,74 | large |
| Pathotype | FTLD_vs_lvPPA_FTLD | f2 | 0,012 | 0,65 | large |
| Pathotype | nfvPPA_FTLD_vs_lvPPA_FTLD | chroma_mean_0 | 0,012 | 0,74 | large |
| Pathotype | nfvPPA_FTLD_vs_lvPPA_FTLD | chroma_mean_1 | 0,012 | 0,74 | large |
| Pathotype | nfvPPA_FTLD_vs_lvPPA_FTLD | chroma_mean_2 | 0,012 | 0,74 | large |
| Pathotype | nfvPPA_FTLD_vs_lvPPA_FTLD | chroma_mean_3 | 0,012 | 0,74 | large |
| Pathotype | nfvPPA_FTLD_vs_lvPPA_FTLD | chroma_mean_4 | 0,012 | 0,74 | large |
| Pathotype | nfvPPA_FTLD_vs_lvPPA_FTLD | chroma_mean_5 | 0,012 | 0,74 | large |
| Pathotype | nfvPPA_FTLD_vs_lvPPA_FTLD | contrast_mean_6 | 0,012 | 0,74 | large |
| Pathotype | nfvPPA_FTLD_vs_lvPPA_FTLD | spectral_centroid_mean | 0,012 | 0,74 | large |
| Pathotype | nfvPPA_FTLD_vs_lvPPA_FTLD | spectral_centroid_std | 0,012 | 0,74 | large |
| Pathotype | nfvPPA_FTLD_vs_lvPPA_FTLD | f2 | 0,012 | 0,74 | large |
| Pathotype | nfvPPA_FTLD_vs_lvPPA_FTLD | pause_total_duration | 0,012 | 0,74 | large |
| Pathotype | svPPA_FTLD_vs_FTLD | nasales_percentile_10 | 0,012 | 0,57 | large |
| Pathotype | FTLD_vs_healthy_control | pause_max_duration | 0,013 | 0,35 | medium |
| Pathotype | FTLD_vs_healthy_control | inter_word_pause_max | 0,013 | 0,35 | medium |
| Pathotype | lvPPA_FTLD_vs_healthy_control | liquides_kurtosis | 0,013 | 0,56 | large |
| Pathotype | svPPA_FTLD_vs_lvPPA_FTLD | max_phoneme_duration | 0,013 | 0,74 | large |
| Pathotype | svPPA_FTLD_vs_lvPPA_FTLD | nasales_median | 0,013 | 0,74 | large |
| Pathotype | healthy_control_vs_amnestic_AD | intensity_std | 0,013 | 0,30 | medium |
| Pathotype | nfvPPA_FTLD_vs_lvPPA_FTLD | total_duration | 0,013 | 0,74 | large |
| Pathotype | nfvPPA_FTLD_vs_lvPPA_FTLD | pause_mean_duration | 0,013 | 0,74 | large |
| Pathotype | nfvPPA_FTLD_vs_lvPPA_FTLD | pause_median_duration | 0,013 | 0,74 | large |
| Pathotype | nfvPPA_FTLD_vs_lvPPA_FTLD | pause_std_duration | 0,013 | 0,74 | large |
| Pathotype | nfvPPA_FTLD_vs_lvPPA_FTLD | inter_word_pause_mean | 0,013 | 0,74 | large |
| Pathotype | nfvPPA_FTLD_vs_lvPPA_FTLD | inter_word_pause_median | 0,013 | 0,74 | large |
| Pathotype | nfvPPA_FTLD_vs_lvPPA_FTLD | inter_word_pause_std | 0,013 | 0,74 | large |
| Pathotype | nfvPPA_FTLD_vs_lvPPA_FTLD | pause_proportion | 0,013 | 0,74 | large |
| Pathotype | nfvPPA_FTLD_vs_lvPPA_FTLD | pause_ratio | 0,013 | 0,74 | large |
| Pathotype | nfvPPA_FTLD_vs_lvPPA_FTLD | fricatives_percentile_90 | 0,013 | 0,74 | large |
| Pathotype | healthy_control_vs_amnestic_AD | nasales_count | 0,013 | 0,30 | medium |
| Pathotype | FTLD_vs_healthy_control | liquides_skewness | 0,013 | 0,35 | medium |
| Pathotype | svPPA_FTLD_vs_lvPPA_FTLD | pause_mean_duration | 0,013 | 0,74 | large |
| Pathotype | svPPA_FTLD_vs_lvPPA_FTLD | pause_median_duration | 0,013 | 0,74 | large |
| Pathotype | svPPA_FTLD_vs_lvPPA_FTLD | inter_word_pause_mean | 0,013 | 0,74 | large |
| Pathotype | svPPA_FTLD_vs_lvPPA_FTLD | inter_word_pause_median | 0,013 | 0,74 | large |
| Pathotype | amnestic_AD_vs_lvPPA_AD | chroma_mean_2 | 0,013 | 0,45 | medium |
| Pathotype | svPPA_FTLD_vs_FTLD | occlusives_percentile_10 | 0,013 | 0,54 | large |
| Pathotype | lvPPA_FTLD_vs_amnestic_AD | contrast_mean_1 | 0,013 | 0,61 | large |
| Pathotype | healthy_control_vs_lvPPA_AD | b3 | 0,013 | 0,39 | medium |
| Pathotype | svPPA_FTLD_vs_lvPPA_AD | semi_voyelles_percentile_90 | 0,013 | 0,59 | large |
| Pathotype | nfvPPA_FTLD_vs_FTLD | voyelles_oral_percentile_90 | 0,013 | 0,56 | large |
| Pathotype | healthy_control_vs_lvPPA_AD | intensity_std | 0,013 | 0,39 | medium |
| Pathotype | FTLD_vs_lvPPA_FTLD | occlusives_median | 0,013 | 0,63 | large |
| Pathotype | nfvPPA_FTLD_vs_amnestic_AD | contrast_mean_5 | 0,013 | 0,53 | large |
| Pathotype | svPPA_FTLD_vs_healthy_control | contrast_mean_3 | 0,013 | 0,47 | medium |
| Pathotype | healthy_control_vs_lvPPA_AD | nasales_median | 0,013 | 0,38 | medium |
| Pathotype | svPPA_FTLD_vs_healthy_control | semi_voyelles_count | 0,013 | 0,46 | medium |
| Pathotype | nfvPPA_FTLD_vs_FTLD | nasales_min | 0,013 | 0,55 | large |
| Pathotype | amnestic_AD_vs_lvPPA_AD | shimmer_apq5 | 0,014 | 0,44 | medium |
| Pathotype | nfvPPA_FTLD_vs_healthy_control | voyelles_oral_std | 0,014 | 0,47 | medium |
| Pathotype | amnestic_AD_vs_lvPPA_AD | contrast_mean_0 | 0,014 | 0,44 | medium |
| Pathotype | svPPA_FTLD_vs_nfvPPA_FTLD | rolloff_mean | 0,014 | 0,66 | large |
| Pathotype | nfvPPA_FTLD_vs_amnestic_AD | semi_voyelles_skewness | 0,014 | 0,52 | large |
| Pathotype | nfvPPA_FTLD_vs_healthy_control | voyelles_oral_median | 0,014 | 0,46 | medium |
| Pathotype | svPPA_FTLD_vs_nfvPPA_FTLD | pause_mean_duration | 0,014 | 0,66 | large |
| Pathotype | svPPA_FTLD_vs_nfvPPA_FTLD | inter_word_pause_mean | 0,014 | 0,66 | large |
| Pathotype | svPPA_FTLD_vs_nfvPPA_FTLD | fricatives_mean | 0,014 | 0,66 | large |
| Pathotype | lvPPA_FTLD_vs_healthy_control | delta_mfcc_mean_11 | 0,014 | 0,55 | large |
| Pathotype | healthy_control_vs_amnestic_AD | semi_voyelles_cv | 0,014 | 0,30 | small |
| Pathotype | nfvPPA_FTLD_vs_lvPPA_AD | liquides_mean | 0,014 | 0,59 | large |
| Pathotype | nfvPPA_FTLD_vs_lvPPA_AD | fricatives_std | 0,014 | 0,59 | large |
| Pathotype | nfvPPA_FTLD_vs_lvPPA_AD | liquides_percentile_90 | 0,014 | 0,59 | large |
| Pathotype | nfvPPA_FTLD_vs_healthy_control | spectral_flux | 0,014 | 0,47 | medium |
| Pathotype | svPPA_FTLD_vs_healthy_control | delta_mfcc_mean_4 | 0,014 | 0,47 | medium |
| Pathotype | FTLD_vs_healthy_control | occlusives_skewness | 0,014 | 0,35 | medium |
| Pathotype | svPPA_FTLD_vs_FTLD | nasales_min | 0,014 | 0,55 | large |
| Pathotype | lvPPA_FTLD_vs_healthy_control | b3 | 0,014 | 0,55 | large |
| Pathotype | FTLD_vs_lvPPA_FTLD | chroma_mean_1 | 0,014 | 0,63 | large |
| Pathotype | svPPA_FTLD_vs_amnestic_AD | f1_cv | 0,014 | 0,52 | large |
| Pathotype | lvPPA_FTLD_vs_lvPPA_AD | chroma_mean_6 | 0,014 | 0,66 | large |
| Pathotype | lvPPA_FTLD_vs_lvPPA_AD | chroma_mean_7 | 0,014 | 0,66 | large |
| Pathotype | svPPA_FTLD_vs_lvPPA_FTLD | articulation_rate | 0,014 | 0,71 | large |
| Pathotype | amnestic_AD_vs_lvPPA_AD | jitter_rap | 0,014 | 0,44 | medium |
| Pathotype | lvPPA_FTLD_vs_lvPPA_AD | pause_mean_duration | 0,015 | 0,66 | large |
| Pathotype | lvPPA_FTLD_vs_lvPPA_AD | pause_std_duration | 0,015 | 0,66 | large |
| Pathotype | lvPPA_FTLD_vs_lvPPA_AD | inter_word_pause_mean | 0,015 | 0,66 | large |
| Pathotype | lvPPA_FTLD_vs_lvPPA_AD | inter_word_pause_std | 0,015 | 0,66 | large |
| Pathotype | lvPPA_FTLD_vs_lvPPA_AD | pause_ratio | 0,015 | 0,66 | large |
| Pathotype | FTLD_vs_lvPPA_FTLD | spectral_flux,1 | 0,015 | 0,63 | large |
| Pathotype | lvPPA_FTLD_vs_healthy_control | f0_std | 0,015 | 0,55 | large |
| Pathotype | FTLD_vs_lvPPA_FTLD | semi_voyelles_iqr | 0,015 | 0,57 | large |
| Pathotype | FTLD_vs_lvPPA_AD | delta_mfcc_mean_11 | 0,015 | 0,48 | medium |
| Pathotype | FTLD_vs_lvPPA_AD | spectral_flux_std | 0,015 | 0,48 | medium |
| Pathotype | svPPA_FTLD_vs_FTLD | fricatives_std | 0,015 | 0,55 | large |
| Pathotype | nfvPPA_FTLD_vs_FTLD | semi_voyelles_percentile_90 | 0,015 | 0,55 | large |
| Pathotype | svPPA_FTLD_vs_FTLD | delta_mfcc_mean_11 | 0,015 | 0,55 | large |
| Pathotype | nfvPPA_FTLD_vs_FTLD | contrast_mean_1 | 0,015 | 0,55 | large |
| Pathotype | FTLD_vs_amnestic_AD | intensity_dynamic_range | 0,015 | 0,40 | medium |
| Pathotype | FTLD_vs_amnestic_AD | chroma_mean_0 | 0,015 | 0,40 | medium |
| Pathotype | svPPA_FTLD_vs_healthy_control | liquides_iqr | 0,015 | 0,47 | medium |
| Pathotype | lvPPA_FTLD_vs_healthy_control | pause_max_duration | 0,015 | 0,55 | large |
| Pathotype | lvPPA_FTLD_vs_healthy_control | inter_word_pause_max | 0,015 | 0,55 | large |
| Pathotype | healthy_control_vs_lvPPA_AD | speech_rate_phonemes_per_sec | 0,015 | 0,38 | medium |
| Pathotype | healthy_control_vs_lvPPA_AD | rate_speech_phonemes | 0,015 | 0,38 | medium |
| Pathotype | svPPA_FTLD_vs_healthy_control | nasales_skewness | 0,015 | 0,47 | medium |
| Pathotype | FTLD_vs_healthy_control | num_final_pauses | 0,016 | 0,18 | small |
| Pathotype | lvPPA_FTLD_vs_amnestic_AD | jitter_ppq5 | 0,016 | 0,59 | large |
| Pathotype | lvPPA_FTLD_vs_lvPPA_AD | semi_voyelles_percentile_10 | 0,016 | 0,65 | large |
| Pathotype | svPPA_FTLD_vs_amnestic_AD | max_phoneme_duration | 0,016 | 0,51 | large |
| Pathotype | healthy_control_vs_amnestic_AD | semi_voyelles_skewness | 0,016 | 0,29 | small |
| Pathotype | FTLD_vs_lvPPA_AD | syllable_count | 0,016 | 0,47 | medium |
| Pathotype | FTLD_vs_lvPPA_AD | voyelles_oral_count | 0,016 | 0,47 | medium |
| Pathotype | healthy_control_vs_amnestic_AD | voyelles_oral_kurtosis | 0,016 | 0,29 | small |
| Pathotype | svPPA_FTLD_vs_healthy_control | occlusives_cv | 0,016 | 0,46 | medium |
| Pathotype | nfvPPA_FTLD_vs_lvPPA_AD | liquides_percentile_10 | 0,016 | 0,58 | large |
| Pathotype | nfvPPA_FTLD_vs_lvPPA_FTLD | delta_mfcc_mean_2 | 0,016 | 0,71 | large |
| Pathotype | nfvPPA_FTLD_vs_lvPPA_FTLD | delta_mfcc_mean_3 | 0,016 | 0,71 | large |
| Pathotype | lvPPA_FTLD_vs_amnestic_AD | b2 | 0,016 | 0,59 | large |
| Pathotype | lvPPA_FTLD_vs_amnestic_AD | voyelles_oral_mean | 0,016 | 0,59 | large |
| Pathotype | svPPA_FTLD_vs_lvPPA_AD | hnr_std | 0,016 | 0,58 | large |
| Pathotype | svPPA_FTLD_vs_lvPPA_AD | hnr_std,1 | 0,016 | 0,58 | large |
| Pathotype | svPPA_FTLD_vs_lvPPA_FTLD | jitter_rap | 0,016 | 0,71 | large |
| Pathotype | svPPA_FTLD_vs_lvPPA_FTLD | delta_mfcc_mean_3 | 0,016 | 0,71 | large |
| Pathotype | svPPA_FTLD_vs_lvPPA_FTLD | zcr_mean | 0,016 | 0,71 | large |
| Pathotype | svPPA_FTLD_vs_lvPPA_FTLD | chroma_mean_0 | 0,016 | 0,71 | large |
| Pathotype | svPPA_FTLD_vs_lvPPA_FTLD | chroma_mean_1 | 0,016 | 0,71 | large |
| Pathotype | svPPA_FTLD_vs_lvPPA_FTLD | chroma_mean_2 | 0,016 | 0,71 | large |
| Pathotype | svPPA_FTLD_vs_lvPPA_FTLD | chroma_mean_3 | 0,016 | 0,71 | large |
| Pathotype | svPPA_FTLD_vs_lvPPA_FTLD | chroma_mean_4 | 0,016 | 0,71 | large |
| Pathotype | svPPA_FTLD_vs_lvPPA_FTLD | chroma_mean_11 | 0,016 | 0,71 | large |
| Pathotype | svPPA_FTLD_vs_lvPPA_FTLD | contrast_mean_1 | 0,016 | 0,71 | large |
| Pathotype | svPPA_FTLD_vs_lvPPA_FTLD | spectral_centroid_std | 0,016 | 0,71 | large |
| Pathotype | svPPA_FTLD_vs_lvPPA_FTLD | b1 | 0,016 | 0,71 | large |
| Pathotype | svPPA_FTLD_vs_lvPPA_FTLD | spectral_instability,1 | 0,016 | 0,71 | large |
| Pathotype | nfvPPA_FTLD_vs_lvPPA_AD | b2 | 0,016 | 0,58 | large |
| Pathotype | healthy_control_vs_lvPPA_AD | mean_phoneme_duration | 0,016 | 0,38 | medium |
| Pathotype | nfvPPA_FTLD_vs_lvPPA_FTLD | f0_slope | 0,016 | 0,71 | large |
| Pathotype | FTLD_vs_healthy_control | spectral_instability,1 | 0,016 | 0,34 | medium |
| Pathotype | svPPA_FTLD_vs_lvPPA_FTLD | pause_max_duration | 0,016 | 0,71 | large |
| Pathotype | svPPA_FTLD_vs_lvPPA_FTLD | inter_word_pause_max | 0,016 | 0,71 | large |
| Pathotype | svPPA_FTLD_vs_lvPPA_FTLD | voyelles_oral_median | 0,016 | 0,71 | large |
| Pathotype | svPPA_FTLD_vs_lvPPA_FTLD | voyelles_oral_max | 0,016 | 0,71 | large |
| Pathotype | svPPA_FTLD_vs_lvPPA_FTLD | fricatives_percentile_90 | 0,016 | 0,71 | large |
| Pathotype | nfvPPA_FTLD_vs_lvPPA_FTLD | voyelles_oral_max | 0,017 | 0,71 | large |
| Pathotype | nfvPPA_FTLD_vs_lvPPA_FTLD | fricatives_iqr | 0,017 | 0,71 | large |
| Pathotype | svPPA_FTLD_vs_lvPPA_FTLD | mean_phoneme_duration | 0,017 | 0,71 | large |
| Pathotype | svPPA_FTLD_vs_lvPPA_FTLD | pause_std_duration | 0,017 | 0,71 | large |
| Pathotype | svPPA_FTLD_vs_lvPPA_FTLD | inter_word_pause_std | 0,017 | 0,71 | large |
| Pathotype | svPPA_FTLD_vs_lvPPA_FTLD | pause_proportion | 0,017 | 0,71 | large |
| Pathotype | svPPA_FTLD_vs_lvPPA_FTLD | pause_ratio | 0,017 | 0,71 | large |
| Pathotype | svPPA_FTLD_vs_lvPPA_FTLD | speech_rate_phonemes_per_sec | 0,017 | 0,71 | large |
| Pathotype | svPPA_FTLD_vs_lvPPA_FTLD | rate_speech_phonemes | 0,017 | 0,71 | large |
| Pathotype | svPPA_FTLD_vs_lvPPA_FTLD | fricatives_std | 0,017 | 0,71 | large |
| Pathotype | svPPA_FTLD_vs_lvPPA_FTLD | fricatives_cv | 0,017 | 0,71 | large |
| Pathotype | svPPA_FTLD_vs_lvPPA_FTLD | fricatives_iqr | 0,017 | 0,71 | large |
| Pathotype | svPPA_FTLD_vs_FTLD | fricatives_cv | 0,017 | 0,54 | large |
| Pathotype | nfvPPA_FTLD_vs_FTLD | semi_voyelles_median | 0,017 | 0,54 | large |
| Pathotype | svPPA_FTLD_vs_FTLD | liquides_kurtosis | 0,017 | 0,54 | large |
| Pathotype | lvPPA_FTLD_vs_healthy_control | f1_cv | 0,017 | 0,54 | large |
| Pathotype | svPPA_FTLD_vs_FTLD | liquides_skewness | 0,017 | 0,54 | large |
| Pathotype | nfvPPA_FTLD_vs_FTLD | liquides_iqr | 0,017 | 0,54 | large |
| Pathotype | svPPA_FTLD_vs_nfvPPA_FTLD | contrast_mean_2 | 0,017 | 0,64 | large |
| Pathotype | FTLD_vs_lvPPA_FTLD | kurt_phoneme_duration | 0,017 | 0,62 | large |
| Pathotype | svPPA_FTLD_vs_amnestic_AD | occlusives_mean | 0,017 | 0,51 | large |
| Pathotype | FTLD_vs_lvPPA_FTLD | voyelles_oral_mean | 0,017 | 0,62 | large |
| Pathotype | svPPA_FTLD_vs_nfvPPA_FTLD | delta_mfcc_mean_6 | 0,017 | 0,64 | large |
| Pathotype | svPPA_FTLD_vs_amnestic_AD | delta_mfcc_mean_2 | 0,017 | 0,51 | large |
| Pathotype | svPPA_FTLD_vs_amnestic_AD | skew_phoneme_duration | 0,017 | 0,51 | large |
| Pathotype | svPPA_FTLD_vs_healthy_control | liquides_cv | 0,017 | 0,46 | medium |
| Pathotype | svPPA_FTLD_vs_nfvPPA_FTLD | nasales_mean | 0,017 | 0,64 | large |
| Pathotype | svPPA_FTLD_vs_amnestic_AD | kurt_phoneme_duration | 0,017 | 0,51 | large |
| Pathotype | nfvPPA_FTLD_vs_healthy_control | fricatives_iqr | 0,017 | 0,46 | medium |
| Pathotype | nfvPPA_FTLD_vs_healthy_control | chroma_mean_7 | 0,017 | 0,46 | medium |
| Pathotype | svPPA_FTLD_vs_healthy_control | nasales_kurtosis | 0,017 | 0,46 | medium |
| Pathotype | lvPPA_FTLD_vs_lvPPA_AD | chroma_mean_4 | 0,017 | 0,64 | large |
| Pathotype | FTLD_vs_lvPPA_AD | b3 | 0,017 | 0,46 | medium |
| Pathotype | FTLD_vs_amnestic_AD | b1 | 0,017 | 0,39 | medium |
| Pathotype | amnestic_AD_vs_lvPPA_AD | liquides_count | 0,017 | 0,42 | medium |
| Pathotype | FTLD_vs_lvPPA_AD | pause_proportion | 0,017 | 0,46 | medium |
| Pathotype | lvPPA_FTLD_vs_healthy_control | nasales_kurtosis | 0,018 | 0,53 | large |
| Pathotype | lvPPA_FTLD_vs_amnestic_AD | pause_mean_duration | 0,018 | 0,58 | large |
| Pathotype | lvPPA_FTLD_vs_amnestic_AD | inter_word_pause_mean | 0,018 | 0,58 | large |
| Pathotype | svPPA_FTLD_vs_FTLD | long_pause_count | 0,018 | 0,53 | large |
| Pathotype | FTLD_vs_healthy_control | nasales_kurtosis | 0,018 | 0,33 | medium |
| Pathotype | FTLD_vs_amnestic_AD | occlusives_skewness | 0,018 | 0,39 | medium |
| Pathotype | amnestic_AD_vs_lvPPA_AD | total_duration | 0,018 | 0,42 | medium |
| Pathotype | lvPPA_FTLD_vs_lvPPA_AD | semi_voyelles_kurtosis | 0,018 | 0,56 | large |
| Pathotype | svPPA_FTLD_vs_amnestic_AD | voyelles_oral_kurtosis | 0,019 | 0,50 | large |
| Pathotype | nfvPPA_FTLD_vs_lvPPA_AD | chroma_mean_6 | 0,019 | 0,56 | large |
| Pathotype | svPPA_FTLD_vs_lvPPA_AD | liquides_skewness | 0,019 | 0,56 | large |
| Pathotype | FTLD_vs_healthy_control | intensity_dynamic_range | 0,019 | 0,33 | medium |
| Pathotype | nfvPPA_FTLD_vs_lvPPA_FTLD | semi_voyelles_std | 0,019 | 0,60 | large |
| Pathotype | nfvPPA_FTLD_vs_lvPPA_FTLD | semi_voyelles_cv | 0,019 | 0,60 | large |
| Pathotype | nfvPPA_FTLD_vs_lvPPA_FTLD | semi_voyelles_iqr | 0,019 | 0,60 | large |
| Pathotype | FTLD_vs_amnestic_AD | rolloff_mean | 0,019 | 0,38 | medium |
| Pathotype | amnestic_AD_vs_lvPPA_AD | jitter_local | 0,019 | 0,42 | medium |
| Pathotype | FTLD_vs_lvPPA_FTLD | chroma_mean_7 | 0,019 | 0,61 | large |
| Pathotype | FTLD_vs_lvPPA_FTLD | skew_phoneme_duration | 0,019 | 0,61 | large |
| Pathotype | healthy_control_vs_amnestic_AD | speech_duration | 0,020 | 0,28 | small |
| Pathotype | healthy_control_vs_amnestic_AD | rmse_mean | 0,020 | 0,28 | small |
| Pathotype | healthy_control_vs_amnestic_AD | intensity_mean | 0,020 | 0,28 | small |
| Pathotype | FTLD_vs_amnestic_AD | occlusives_max | 0,020 | 0,38 | medium |
| Pathotype | FTLD_vs_healthy_control | chroma_mean_2 | 0,020 | 0,33 | medium |
| Pathotype | svPPA_FTLD_vs_healthy_control | b3 | 0,020 | 0,45 | medium |
| Pathotype | svPPA_FTLD_vs_lvPPA_FTLD | occlusives_percentile_10 | 0,020 | 0,69 | large |
| Pathotype | nfvPPA_FTLD_vs_amnestic_AD | nasales_cv | 0,020 | 0,49 | medium |
| Pathotype | nfvPPA_FTLD_vs_healthy_control | occlusives_min | 0,020 | 0,41 | medium |
| Pathotype | nfvPPA_FTLD_vs_amnestic_AD | voyelles_oral_percentile_90 | 0,020 | 0,49 | medium |
| Pathotype | nfvPPA_FTLD_vs_amnestic_AD | contrast_mean_4 | 0,020 | 0,49 | medium |
| Pathotype | svPPA_FTLD_vs_amnestic_AD | delta_mfcc_mean_10 | 0,020 | 0,49 | medium |
| Pathotype | svPPA_FTLD_vs_amnestic_AD | fricatives_mean | 0,020 | 0,49 | medium |
| Pathotype | FTLD_vs_healthy_control | cv_phoneme_duration | 0,020 | 0,33 | medium |
| Pathotype | healthy_control_vs_amnestic_AD | voyelles_oral_percentile_90 | 0,020 | 0,28 | small |
| Pathotype | FTLD_vs_lvPPA_AD | f3_cv | 0,020 | 0,45 | medium |
| Pathotype | healthy_control_vs_lvPPA_AD | occlusives_median | 0,020 | 0,36 | medium |
| Pathotype | svPPA_FTLD_vs_lvPPA_AD | liquides_min | 0,020 | 0,54 | large |
| Pathotype | FTLD_vs_lvPPA_AD | f3_cv,1 | 0,021 | 0,45 | medium |
| Pathotype | lvPPA_FTLD_vs_lvPPA_AD | occlusives_kurtosis | 0,021 | 0,63 | large |
| Pathotype | lvPPA_FTLD_vs_lvPPA_AD | liquides_skewness | 0,021 | 0,63 | large |
| Pathotype | svPPA_FTLD_vs_nfvPPA_FTLD | intensity_std | 0,021 | 0,62 | large |
| Pathotype | svPPA_FTLD_vs_nfvPPA_FTLD | max_phoneme_duration | 0,021 | 0,62 | large |
| Pathotype | svPPA_FTLD_vs_nfvPPA_FTLD | liquides_std | 0,021 | 0,62 | large |
| Pathotype | svPPA_FTLD_vs_nfvPPA_FTLD | cv_phoneme_duration | 0,021 | 0,62 | large |
| Pathotype | lvPPA_FTLD_vs_lvPPA_AD | pause_min_duration | 0,021 | 0,63 | large |
| Pathotype | lvPPA_FTLD_vs_lvPPA_AD | inter_word_pause_min | 0,021 | 0,63 | large |
| Pathotype | lvPPA_FTLD_vs_lvPPA_AD | pause_frequency | 0,021 | 0,63 | large |
| Pathotype | nfvPPA_FTLD_vs_FTLD | delta_mfcc_mean_2 | 0,021 | 0,52 | large |
| Pathotype | lvPPA_FTLD_vs_amnestic_AD | liquides_kurtosis | 0,021 | 0,56 | large |
| Pathotype | lvPPA_FTLD_vs_amnestic_AD | nasales_kurtosis | 0,021 | 0,56 | large |
| Pathotype | nfvPPA_FTLD_vs_lvPPA_FTLD | b3 | 0,021 | 0,69 | large |
| Pathotype | FTLD_vs_healthy_control | f0_max | 0,021 | 0,33 | medium |
| Pathotype | nfvPPA_FTLD_vs_lvPPA_AD | voyelles_oral_percentile_10 | 0,021 | 0,55 | large |
| Pathotype | FTLD_vs_amnestic_AD | nasales_kurtosis | 0,021 | 0,38 | medium |
| Pathotype | svPPA_FTLD_vs_FTLD | liquides_cv | 0,021 | 0,52 | large |
| Pathotype | svPPA_FTLD_vs_amnestic_AD | delta_mfcc_mean_1 | 0,021 | 0,49 | medium |
| Pathotype | svPPA_FTLD_vs_FTLD | voyelles_oral_kurtosis | 0,021 | 0,52 | large |
| Pathotype | svPPA_FTLD_vs_lvPPA_FTLD | jitter_local | 0,021 | 0,69 | large |
| Pathotype | svPPA_FTLD_vs_lvPPA_FTLD | f3_cv | 0,021 | 0,69 | large |
| Pathotype | svPPA_FTLD_vs_FTLD | kurt_phoneme_duration | 0,021 | 0,52 | large |
| Pathotype | svPPA_FTLD_vs_healthy_control | semi_voyelles_std | 0,021 | 0,44 | medium |
| Pathotype | lvPPA_FTLD_vs_amnestic_AD | f2 | 0,022 | 0,56 | large |
| Pathotype | svPPA_FTLD_vs_amnestic_AD | delta_mfcc_mean_4 | 0,022 | 0,49 | medium |
| Pathotype | svPPA_FTLD_vs_lvPPA_AD | delta_mfcc_mean_2 | 0,022 | 0,55 | large |
| Pathotype | svPPA_FTLD_vs_lvPPA_AD | max_phoneme_duration | 0,022 | 0,55 | large |
| Pathotype | svPPA_FTLD_vs_lvPPA_AD | liquides_mean | 0,022 | 0,55 | large |
| Pathotype | nfvPPA_FTLD_vs_lvPPA_AD | chroma_mean_4 | 0,022 | 0,55 | large |
| Pathotype | amnestic_AD_vs_lvPPA_AD | f0_min | 0,022 | 0,28 | small |
| Pathotype | svPPA_FTLD_vs_lvPPA_FTLD | std_phoneme_duration | 0,022 | 0,69 | large |
| Pathotype | nfvPPA_FTLD_vs_healthy_control | pause_min_duration | 0,022 | 0,43 | medium |
| Pathotype | nfvPPA_FTLD_vs_healthy_control | inter_word_pause_min | 0,022 | 0,43 | medium |
| Pathotype | svPPA_FTLD_vs_amnestic_AD | f3_cv,1 | 0,022 | 0,49 | medium |
| Pathotype | FTLD_vs_healthy_control | f1_cv | 0,022 | 0,33 | medium |
| Pathotype | amnestic_AD_vs_lvPPA_AD | f1 | 0,022 | 0,41 | medium |
| Pathotype | FTLD_vs_lvPPA_AD | chroma_mean_11 | 0,022 | 0,45 | medium |
| Pathotype | FTLD_vs_amnestic_AD | intensity_std | 0,022 | 0,38 | medium |
| Pathotype | svPPA_FTLD_vs_healthy_control | b2 | 0,022 | 0,44 | medium |
| Pathotype | nfvPPA_FTLD_vs_healthy_control | spectral_instability,1 | 0,022 | 0,44 | medium |
| Pathotype | FTLD_vs_lvPPA_FTLD | fricatives_percentile_90 | 0,022 | 0,59 | large |
| Pathotype | FTLD_vs_lvPPA_FTLD | pause_std_duration | 0,022 | 0,59 | large |
| Pathotype | FTLD_vs_lvPPA_FTLD | inter_word_pause_std | 0,022 | 0,59 | large |
| Pathotype | svPPA_FTLD_vs_healthy_control | hnr_std | 0,023 | 0,44 | medium |
| Pathotype | FTLD_vs_healthy_control | fricatives_kurtosis | 0,023 | 0,32 | medium |
| Pathotype | healthy_control_vs_amnestic_AD | semi_voyelles_std | 0,023 | 0,28 | small |
| Pathotype | svPPA_FTLD_vs_healthy_control | semi_voyelles_max | 0,023 | 0,44 | medium |
| Pathotype | amnestic_AD_vs_lvPPA_AD | pause_cv | 0,023 | 0,41 | medium |
| Pathotype | svPPA_FTLD_vs_FTLD | delta_mfcc_mean_3 | 0,023 | 0,51 | large |
| Pathotype | FTLD_vs_healthy_control | spectral_centroid_slope | 0,023 | 0,32 | medium |
| Pathotype | svPPA_FTLD_vs_FTLD | voyelles_oral_median | 0,023 | 0,51 | large |
| Pathotype | healthy_control_vs_amnestic_AD | total_duration | 0,023 | 0,28 | small |
| Pathotype | svPPA_FTLD_vs_FTLD | liquides_percentile_90 | 0,024 | 0,51 | large |
| Pathotype | FTLD_vs_healthy_control | voyelles_oral_percentile_90 | 0,024 | 0,32 | medium |
| Pathotype | lvPPA_FTLD_vs_amnestic_AD | fricatives_skewness | 0,024 | 0,55 | large |
| Pathotype | svPPA_FTLD_vs_healthy_control | spectral_flux,1 | 0,024 | 0,43 | medium |
| Pathotype | svPPA_FTLD_vs_healthy_control | delta_mfcc_mean_9 | 0,024 | 0,43 | medium |
| Pathotype | FTLD_vs_healthy_control | semi_voyelles_kurtosis | 0,024 | 0,32 | medium |
| Pathotype | lvPPA_FTLD_vs_healthy_control | delta_mfcc_mean_5 | 0,024 | 0,51 | large |
| Pathotype | amnestic_AD_vs_lvPPA_AD | fricatives_percentile_90 | 0,025 | 0,40 | medium |
| Pathotype | amnestic_AD_vs_lvPPA_AD | liquides_percentile_10 | 0,025 | 0,39 | medium |
| Pathotype | amnestic_AD_vs_lvPPA_AD | chroma_mean_4 | 0,025 | 0,40 | medium |
| Pathotype | nfvPPA_FTLD_vs_amnestic_AD | occlusives_percentile_10 | 0,025 | 0,44 | medium |
| Pathotype | nfvPPA_FTLD_vs_lvPPA_AD | std_phoneme_duration | 0,025 | 0,54 | large |
| Pathotype | lvPPA_FTLD_vs_lvPPA_AD | delta_mfcc_mean_11 | 0,025 | 0,61 | large |
| Pathotype | lvPPA_FTLD_vs_lvPPA_AD | chroma_mean_0 | 0,025 | 0,61 | large |
| Pathotype | lvPPA_FTLD_vs_lvPPA_AD | chroma_mean_2 | 0,025 | 0,61 | large |
| Pathotype | lvPPA_FTLD_vs_lvPPA_AD | spectral_centroid_std | 0,025 | 0,61 | large |
| Pathotype | lvPPA_FTLD_vs_lvPPA_AD | rolloff_std | 0,025 | 0,61 | large |
| Pathotype | lvPPA_FTLD_vs_lvPPA_AD | f0_slope | 0,025 | 0,61 | large |
| Pathotype | healthy_control_vs_lvPPA_AD | liquides_max | 0,025 | 0,35 | medium |
| Pathotype | lvPPA_FTLD_vs_lvPPA_AD | std_phoneme_duration | 0,025 | 0,61 | large |
| Pathotype | lvPPA_FTLD_vs_lvPPA_AD | voyelles_oral_max | 0,025 | 0,61 | large |
| Pathotype | svPPA_FTLD_vs_healthy_control | rolloff_mean | 0,025 | 0,43 | medium |
| Pathotype | lvPPA_FTLD_vs_lvPPA_AD | fricatives_min | 0,025 | 0,60 | large |
| Pathotype | lvPPA_FTLD_vs_healthy_control | shimmer_local | 0,025 | 0,50 | large |
| Pathotype | nfvPPA_FTLD_vs_healthy_control | pause_median_duration | 0,025 | 0,43 | medium |
| Pathotype | nfvPPA_FTLD_vs_healthy_control | inter_word_pause_median | 0,025 | 0,43 | medium |
| Pathotype | svPPA_FTLD_vs_healthy_control | hnr_mean | 0,025 | 0,43 | medium |
| Pathotype | svPPA_FTLD_vs_amnestic_AD | cv_phoneme_duration | 0,025 | 0,48 | medium |
| Pathotype | svPPA_FTLD_vs_amnestic_AD | pause_cv | 0,025 | 0,48 | medium |
| Pathotype | healthy_control_vs_lvPPA_AD | spectral_centroid_slope | 0,025 | 0,35 | medium |
| Pathotype | nfvPPA_FTLD_vs_lvPPA_AD | articulation_rate | 0,026 | 0,30 | medium |
| Pathotype | svPPA_FTLD_vs_healthy_control | occlusives_max | 0,026 | 0,43 | medium |
| Pathotype | svPPA_FTLD_vs_FTLD | semi_voyelles_percentile_90 | 0,026 | 0,50 | large |
| Pathotype | FTLD_vs_lvPPA_FTLD | occlusives_cv | 0,026 | 0,58 | large |
| Pathotype | svPPA_FTLD_vs_healthy_control | articulation_rate | 0,026 | 0,43 | medium |
| Pathotype | healthy_control_vs_lvPPA_AD | nasales_skewness | 0,026 | 0,35 | medium |
| Pathotype | nfvPPA_FTLD_vs_healthy_control | delta_mfcc_mean_3 | 0,026 | 0,43 | medium |
| Pathotype | nfvPPA_FTLD_vs_FTLD | chroma_mean_8 | 0,026 | 0,50 | large |
| Pathotype | nfvPPA_FTLD_vs_amnestic_AD | voyelles_oral_percentile_10 | 0,026 | 0,46 | medium |
| Pathotype | FTLD_vs_healthy_control | spectral_centroid_std | 0,026 | 0,32 | medium |
| Pathotype | svPPA_FTLD_vs_FTLD | hnr_mean,1 | 0,026 | 0,50 | large |
| Pathotype | svPPA_FTLD_vs_amnestic_AD | fricatives_median | 0,027 | 0,47 | medium |
| Pathotype | FTLD_vs_amnestic_AD | f3 | 0,027 | 0,36 | medium |
| Pathotype | FTLD_vs_amnestic_AD | chroma_mean_4 | 0,027 | 0,36 | medium |
| Pathotype | FTLD_vs_lvPPA_AD | nasales_max | 0,027 | 0,43 | medium |
| Pathotype | svPPA_FTLD_vs_amnestic_AD | f0_min | 0,027 | 0,42 | medium |
| Pathotype | healthy_control_vs_lvPPA_AD | semi_voyelles_min | 0,027 | 0,35 | medium |
| Pathotype | svPPA_FTLD_vs_amnestic_AD | delta_mfcc_mean_6 | 0,027 | 0,47 | medium |
| Pathotype | FTLD_vs_lvPPA_FTLD | fricatives_min | 0,027 | 0,56 | large |
| Pathotype | amnestic_AD_vs_lvPPA_AD | jitter_ppq5 | 0,027 | 0,39 | medium |
| Pathotype | healthy_control_vs_amnestic_AD | f0_mean | 0,027 | 0,27 | small |
| Pathotype | nfvPPA_FTLD_vs_amnestic_AD | nasales_kurtosis | 0,027 | 0,47 | medium |
| Pathotype | svPPA_FTLD_vs_amnestic_AD | f0_std | 0,027 | 0,47 | medium |
| Pathotype | svPPA_FTLD_vs_lvPPA_FTLD | shimmer_apq3 | 0,027 | 0,66 | large |
| Pathotype | svPPA_FTLD_vs_lvPPA_FTLD | shimmer_dda | 0,027 | 0,66 | large |
| Pathotype | svPPA_FTLD_vs_lvPPA_FTLD | delta_mfcc_mean_2 | 0,027 | 0,66 | large |
| Pathotype | svPPA_FTLD_vs_lvPPA_FTLD | delta_mfcc_mean_10 | 0,027 | 0,66 | large |
| Pathotype | svPPA_FTLD_vs_lvPPA_FTLD | chroma_mean_7 | 0,027 | 0,66 | large |
| Pathotype | svPPA_FTLD_vs_lvPPA_FTLD | contrast_mean_6 | 0,027 | 0,66 | large |
| Pathotype | svPPA_FTLD_vs_lvPPA_FTLD | b3 | 0,027 | 0,66 | large |
| Pathotype | nfvPPA_FTLD_vs_amnestic_AD | fricatives_mean | 0,027 | 0,47 | medium |
| Pathotype | nfvPPA_FTLD_vs_lvPPA_FTLD | voyelles_oral_skewness | 0,028 | 0,66 | large |
| Pathotype | FTLD_vs_amnestic_AD | occlusives_median | 0,028 | 0,36 | medium |
| Pathotype | lvPPA_FTLD_vs_amnestic_AD | nasales_min | 0,028 | 0,54 | large |
| Pathotype | FTLD_vs_amnestic_AD | occlusives_iqr | 0,028 | 0,36 | medium |
| Pathotype | FTLD_vs_amnestic_AD | b3 | 0,028 | 0,36 | medium |
| Pathotype | svPPA_FTLD_vs_healthy_control | contrast_mean_5 | 0,028 | 0,42 | medium |
| Pathotype | FTLD_vs_amnestic_AD | nasales_skewness | 0,028 | 0,36 | medium |
| Pathotype | FTLD_vs_lvPPA_FTLD | semi_voyelles_min | 0,028 | 0,55 | large |
| Pathotype | nfvPPA_FTLD_vs_lvPPA_AD | liquides_max | 0,028 | 0,53 | large |
| Pathotype | FTLD_vs_healthy_control | f2 | 0,028 | 0,31 | medium |
| Pathotype | healthy_control_vs_amnestic_AD | occlusives_cv | 0,028 | 0,27 | small |
| Pathotype | lvPPA_FTLD_vs_amnestic_AD | semi_voyelles_skewness | 0,028 | 0,53 | large |
| Pathotype | svPPA_FTLD_vs_lvPPA_AD | intensity_dynamic_range | 0,028 | 0,53 | large |
| Pathotype | nfvPPA_FTLD_vs_lvPPA_AD | spectral_centroid_slope | 0,028 | 0,53 | large |
| Pathotype | nfvPPA_FTLD_vs_lvPPA_AD | occlusives_mean | 0,028 | 0,53 | large |
| Pathotype | healthy_control_vs_lvPPA_AD | occlusives_cv | 0,028 | 0,35 | medium |
| Pathotype | amnestic_AD_vs_lvPPA_AD | min_phoneme_duration | 0,028 | 0,34 | medium |
| Pathotype | svPPA_FTLD_vs_lvPPA_AD | std_phoneme_duration | 0,028 | 0,53 | large |
| Pathotype | svPPA_FTLD_vs_lvPPA_AD | cv_phoneme_duration | 0,028 | 0,53 | large |
| Pathotype | FTLD_vs_healthy_control | long_pause_count | 0,029 | 0,31 | medium |
| Pathotype | nfvPPA_FTLD_vs_healthy_control | b3 | 0,029 | 0,42 | medium |
| Pathotype | FTLD_vs_lvPPA_FTLD | f0_std | 0,029 | 0,56 | large |
| Pathotype | amnestic_AD_vs_lvPPA_AD | delta_mfcc_mean_8 | 0,029 | 0,39 | medium |
| Pathotype | amnestic_AD_vs_lvPPA_AD | spectral_centroid_mean | 0,029 | 0,39 | medium |
| Pathotype | svPPA_FTLD_vs_healthy_control | delta_mfcc_mean_10 | 0,029 | 0,42 | medium |
| Pathotype | svPPA_FTLD_vs_FTLD | b3 | 0,029 | 0,50 | medium |
| Pathotype | svPPA_FTLD_vs_FTLD | pause_total_duration | 0,029 | 0,50 | medium |
| Pathotype | FTLD_vs_lvPPA_FTLD | voyelles_oral_std | 0,029 | 0,56 | large |
| Pathotype | svPPA_FTLD_vs_FTLD | pause_ratio | 0,029 | 0,50 | medium |
| Pathotype | FTLD_vs_amnestic_AD | chroma_mean_1 | 0,029 | 0,36 | medium |
| Pathotype | lvPPA_FTLD_vs_lvPPA_AD | cv_phoneme_duration | 0,030 | 0,59 | large |
| Pathotype | FTLD_vs_healthy_control | delta_mfcc_mean_3 | 0,030 | 0,31 | medium |
| Pathotype | FTLD_vs_lvPPA_FTLD | min_phoneme_duration | 0,030 | 0,56 | large |
| Pathotype | FTLD_vs_healthy_control | occlusives_kurtosis | 0,030 | 0,31 | medium |
| Pathotype | FTLD_vs_healthy_control | semi_voyelles_median | 0,030 | 0,31 | medium |
| Pathotype | FTLD_vs_lvPPA_AD | delta_mfcc_mean_12 | 0,030 | 0,42 | medium |
| Pathotype | svPPA_FTLD_vs_healthy_control | nasales_max | 0,031 | 0,41 | medium |
| Pathotype | svPPA_FTLD_vs_nfvPPA_FTLD | spectral_flux_std | 0,031 | 0,58 | large |
| Pathotype | svPPA_FTLD_vs_nfvPPA_FTLD | skew_phoneme_duration | 0,031 | 0,58 | large |
| Pathotype | svPPA_FTLD_vs_nfvPPA_FTLD | kurt_phoneme_duration | 0,031 | 0,58 | large |
| Pathotype | healthy_control_vs_lvPPA_AD | f1_cv | 0,031 | 0,34 | medium |
| Pathotype | FTLD_vs_amnestic_AD | voyelles_oral_std | 0,031 | 0,35 | medium |
| Pathotype | healthy_control_vs_amnestic_AD | chroma_mean_0 | 0,031 | 0,26 | small |
| Pathotype | healthy_control_vs_lvPPA_AD | occlusives_percentile_10 | 0,031 | 0,34 | medium |
| Pathotype | healthy_control_vs_lvPPA_AD | liquides_iqr | 0,031 | 0,34 | medium |
| Pathotype | healthy_control_vs_lvPPA_AD | chroma_mean_2 | 0,032 | 0,34 | medium |
| Pathotype | nfvPPA_FTLD_vs_healthy_control | pause_cv | 0,032 | 0,41 | medium |
| Pathotype | FTLD_vs_amnestic_AD | occlusives_min | 0,032 | 0,34 | medium |
| Pathotype | FTLD_vs_amnestic_AD | delta_mfcc_mean_10 | 0,032 | 0,35 | medium |
| Pathotype | FTLD_vs_amnestic_AD | rolloff_std | 0,032 | 0,35 | medium |
| Pathotype | svPPA_FTLD_vs_lvPPA_AD | syllable_rate_per_sec | 0,033 | 0,51 | large |
| Pathotype | svPPA_FTLD_vs_lvPPA_AD | occlusives_iqr | 0,033 | 0,51 | large |
| Pathotype | svPPA_FTLD_vs_healthy_control | pause_total_duration | 0,033 | 0,41 | medium |
| Pathotype | nfvPPA_FTLD_vs_FTLD | f3_cv | 0,033 | 0,49 | medium |
| Pathotype | amnestic_AD_vs_lvPPA_AD | liquides_skewness | 0,033 | 0,38 | medium |
| Pathotype | amnestic_AD_vs_lvPPA_AD | num_inter_pauses | 0,033 | 0,38 | medium |
| Pathotype | FTLD_vs_lvPPA_AD | spectral_flux,1 | 0,033 | 0,42 | medium |
| Pathotype | FTLD_vs_lvPPA_AD | nasales_min | 0,033 | 0,41 | medium |
| Pathotype | lvPPA_FTLD_vs_healthy_control | chroma_mean_11 | 0,033 | 0,48 | medium |
| Pathotype | lvPPA_FTLD_vs_amnestic_AD | jitter_local | 0,034 | 0,52 | large |
| Pathotype | svPPA_FTLD_vs_amnestic_AD | semi_voyelles_max | 0,034 | 0,45 | medium |
| Pathotype | FTLD_vs_amnestic_AD | spectral_centroid_std | 0,034 | 0,35 | medium |
| Pathotype | nfvPPA_FTLD_vs_amnestic_AD | voyelles_oral_iqr | 0,034 | 0,45 | medium |
| Pathotype | svPPA_FTLD_vs_amnestic_AD | syllable_rate_per_sec | 0,034 | 0,45 | medium |
| Pathotype | lvPPA_FTLD_vs_amnestic_AD | chroma_mean_11 | 0,034 | 0,52 | large |
| Pathotype | svPPA_FTLD_vs_healthy_control | nasales_cv | 0,035 | 0,41 | medium |
| Pathotype | lvPPA_FTLD_vs_amnestic_AD | contrast_mean_6 | 0,035 | 0,52 | large |
| Pathotype | lvPPA_FTLD_vs_amnestic_AD | pause_proportion | 0,035 | 0,52 | large |
| Pathotype | lvPPA_FTLD_vs_amnestic_AD | voyelles_oral_std | 0,035 | 0,52 | large |
| Pathotype | lvPPA_FTLD_vs_lvPPA_AD | f1_cv | 0,035 | 0,57 | large |
| Pathotype | lvPPA_FTLD_vs_lvPPA_AD | spectral_flux_std | 0,035 | 0,57 | large |
| Pathotype | lvPPA_FTLD_vs_lvPPA_AD | b3 | 0,035 | 0,57 | large |
| Pathotype | nfvPPA_FTLD_vs_lvPPA_FTLD | f1_cv | 0,035 | 0,63 | large |
| Pathotype | nfvPPA_FTLD_vs_lvPPA_FTLD | rolloff_std | 0,035 | 0,63 | large |
| Pathotype | nfvPPA_FTLD_vs_lvPPA_FTLD | b2 | 0,035 | 0,63 | large |
| Pathotype | svPPA_FTLD_vs_lvPPA_FTLD | liquides_min | 0,035 | 0,63 | large |
| Pathotype | healthy_control_vs_lvPPA_AD | intensity_slope | 0,035 | 0,33 | medium |
| Pathotype | FTLD_vs_amnestic_AD | delta_mfcc_mean_7 | 0,035 | 0,35 | medium |
| Pathotype | nfvPPA_FTLD_vs_lvPPA_FTLD | voyelles_oral_mean | 0,035 | 0,63 | large |
| Pathotype | nfvPPA_FTLD_vs_lvPPA_FTLD | voyelles_oral_std | 0,035 | 0,63 | large |
| Pathotype | nfvPPA_FTLD_vs_lvPPA_FTLD | voyelles_oral_percentile_90 | 0,035 | 0,63 | large |
| Pathotype | nfvPPA_FTLD_vs_lvPPA_FTLD | voyelles_oral_iqr | 0,035 | 0,63 | large |
| Pathotype | nfvPPA_FTLD_vs_lvPPA_FTLD | fricatives_cv | 0,035 | 0,63 | large |
| Pathotype | amnestic_AD_vs_lvPPA_AD | num_pauses | 0,036 | 0,38 | medium |
| Pathotype | amnestic_AD_vs_lvPPA_AD | num_inter_word_pauses | 0,036 | 0,38 | medium |
| Pathotype | healthy_control_vs_amnestic_AD | nasales_percentile_10 | 0,036 | 0,25 | small |
| Pathotype | nfvPPA_FTLD_vs_healthy_control | pause_mean_duration | 0,036 | 0,40 | medium |
| Pathotype | nfvPPA_FTLD_vs_healthy_control | inter_word_pause_mean | 0,036 | 0,40 | medium |
| Pathotype | lvPPA_FTLD_vs_healthy_control | jitter_ppq5 | 0,036 | 0,47 | medium |
| Pathotype | nfvPPA_FTLD_vs_FTLD | voyelles_oral_percentile_10 | 0,036 | 0,48 | medium |
| Pathotype | svPPA_FTLD_vs_FTLD | contrast_mean_6 | 0,036 | 0,48 | medium |
| Pathotype | svPPA_FTLD_vs_FTLD | nasales_percentile_90 | 0,036 | 0,48 | medium |
| Pathotype | svPPA_FTLD_vs_FTLD | spectral_flux | 0,036 | 0,48 | medium |
| Pathotype | healthy_control_vs_amnestic_AD | semi_voyelles_max | 0,036 | 0,26 | small |
| Pathotype | nfvPPA_FTLD_vs_FTLD | liquides_median | 0,036 | 0,48 | medium |
| Pathotype | svPPA_FTLD_vs_FTLD | spectral_flux,1 | 0,036 | 0,48 | medium |
| Pathotype | svPPA_FTLD_vs_FTLD | pause_proportion | 0,036 | 0,48 | medium |
| Pathotype | svPPA_FTLD_vs_amnestic_AD | liquides_percentile_10 | 0,036 | 0,43 | medium |
| Pathotype | healthy_control_vs_amnestic_AD | occlusives_std | 0,037 | 0,25 | small |
| Pathotype | svPPA_FTLD_vs_amnestic_AD | contrast_mean_2 | 0,037 | 0,44 | medium |
| Pathotype | nfvPPA_FTLD_vs_lvPPA_AD | delta_mfcc_mean_2 | 0,037 | 0,50 | large |
| Pathotype | FTLD_vs_amnestic_AD | nasales_cv | 0,037 | 0,34 | medium |
| Pathotype | svPPA_FTLD_vs_lvPPA_AD | liquides_percentile_90 | 0,037 | 0,50 | large |
| Pathotype | nfvPPA_FTLD_vs_lvPPA_AD | voyelles_oral_mean | 0,037 | 0,50 | large |
| Pathotype | nfvPPA_FTLD_vs_lvPPA_AD | fricatives_mean | 0,037 | 0,50 | large |
| Pathotype | FTLD_vs_healthy_control | chroma_mean_0 | 0,037 | 0,30 | small |
| Pathotype | svPPA_FTLD_vs_nfvPPA_FTLD | pause_proportion | 0,037 | 0,56 | large |
| Pathotype | svPPA_FTLD_vs_nfvPPA_FTLD | pause_ratio | 0,037 | 0,56 | large |
| Pathotype | svPPA_FTLD_vs_nfvPPA_FTLD | fricatives_median | 0,037 | 0,56 | large |
| Pathotype | healthy_control_vs_amnestic_AD | pause_cv | 0,037 | 0,25 | small |
| Pathotype | svPPA_FTLD_vs_healthy_control | shimmer_apq3 | 0,037 | 0,40 | medium |
| Pathotype | svPPA_FTLD_vs_healthy_control | shimmer_dda | 0,037 | 0,40 | medium |
| Pathotype | lvPPA_FTLD_vs_amnestic_AD | jitter_rap | 0,038 | 0,51 | large |
| Pathotype | nfvPPA_FTLD_vs_FTLD | semi_voyelles_count | 0,038 | 0,47 | medium |
| Pathotype | lvPPA_FTLD_vs_amnestic_AD | pause_max_duration | 0,038 | 0,51 | large |
| Pathotype | lvPPA_FTLD_vs_amnestic_AD | inter_word_pause_max | 0,038 | 0,51 | large |
| Pathotype | healthy_control_vs_lvPPA_AD | min_phoneme_duration | 0,038 | 0,29 | small |
| Pathotype | FTLD_vs_lvPPA_FTLD | f3_cv | 0,038 | 0,54 | large |
| Pathotype | FTLD_vs_lvPPA_AD | fricatives_std | 0,038 | 0,40 | medium |
| Pathotype | FTLD_vs_lvPPA_FTLD | f3_cv,1 | 0,038 | 0,54 | large |
| Pathotype | FTLD_vs_lvPPA_FTLD | occlusives_skewness | 0,038 | 0,54 | large |
| Pathotype | nfvPPA_FTLD_vs_lvPPA_AD | occlusives_min | 0,038 | 0,49 | medium |
| Pathotype | svPPA_FTLD_vs_healthy_control | total_duration | 0,038 | 0,40 | medium |
| Pathotype | FTLD_vs_amnestic_AD | fricatives_skewness | 0,039 | 0,34 | medium |
| Pathotype | svPPA_FTLD_vs_healthy_control | nasales_median | 0,039 | 0,39 | medium |
| Pathotype | FTLD_vs_healthy_control | fricatives_std | 0,039 | 0,29 | small |
| Pathotype | nfvPPA_FTLD_vs_amnestic_AD | fricatives_cv | 0,039 | 0,44 | medium |
| Pathotype | nfvPPA_FTLD_vs_amnestic_AD | fricatives_max | 0,039 | 0,44 | medium |
| Pathotype | healthy_control_vs_lvPPA_AD | voyelles_oral_percentile_10 | 0,039 | 0,29 | small |
| Pathotype | healthy_control_vs_amnestic_AD | pause_min_duration | 0,040 | 0,25 | small |
| Pathotype | healthy_control_vs_amnestic_AD | inter_word_pause_min | 0,040 | 0,25 | small |
| Pathotype | svPPA_FTLD_vs_amnestic_AD | liquides_cv | 0,040 | 0,44 | medium |
| Pathotype | FTLD_vs_healthy_control | spectral_flux | 0,040 | 0,29 | small |
| Pathotype | lvPPA_FTLD_vs_healthy_control | fricatives_min | 0,040 | 0,45 | medium |
| Pathotype | svPPA_FTLD_vs_FTLD | f3_cv | 0,040 | 0,47 | medium |
| Pathotype | nfvPPA_FTLD_vs_healthy_control | liquides_min | 0,040 | 0,38 | medium |
| Pathotype | nfvPPA_FTLD_vs_FTLD | occlusives_std | 0,040 | 0,47 | medium |
| Pathotype | svPPA_FTLD_vs_FTLD | f3_cv,1 | 0,040 | 0,47 | medium |
| Pathotype | svPPA_FTLD_vs_FTLD | occlusives_skewness | 0,040 | 0,47 | medium |
| Pathotype | nfvPPA_FTLD_vs_FTLD | f3_cv,1 | 0,040 | 0,47 | medium |
| Pathotype | FTLD_vs_healthy_control | contrast_mean_2 | 0,040 | 0,29 | small |
| Pathotype | FTLD_vs_lvPPA_AD | median_phoneme_duration | 0,041 | 0,40 | medium |
| Pathotype | lvPPA_FTLD_vs_lvPPA_AD | f2_cv,1 | 0,041 | 0,55 | large |
| Pathotype | FTLD_vs_lvPPA_AD | occlusives_percentile_90 | 0,041 | 0,40 | medium |
| Pathotype | nfvPPA_FTLD_vs_amnestic_AD | liquides_min | 0,041 | 0,43 | medium |
| Pathotype | FTLD_vs_lvPPA_AD | num_final_pauses | 0,041 | 0,24 | small |
| Pathotype | svPPA_FTLD_vs_healthy_control | nasales_std | 0,042 | 0,39 | medium |
| Pathotype | svPPA_FTLD_vs_healthy_control | spectral_flux | 0,042 | 0,39 | medium |
| Pathotype | svPPA_FTLD_vs_healthy_control | nasales_count | 0,042 | 0,38 | medium |
| Pathotype | svPPA_FTLD_vs_lvPPA_AD | semi_voyelles_iqr | 0,042 | 0,48 | medium |
| Pathotype | healthy_control_vs_lvPPA_AD | fricatives_percentile_10 | 0,042 | 0,30 | medium |
| Pathotype | svPPA_FTLD_vs_healthy_control | syllable_count | 0,042 | 0,39 | medium |
| Pathotype | svPPA_FTLD_vs_healthy_control | voyelles_oral_count | 0,042 | 0,39 | medium |
| Pathotype | nfvPPA_FTLD_vs_FTLD | num_initial_pauses | 0,042 | 0,20 | small |
| Pathotype | svPPA_FTLD_vs_amnestic_AD | semi_voyelles_std | 0,042 | 0,43 | medium |
| Pathotype | nfvPPA_FTLD_vs_healthy_control | voyelles_oral_percentile_10 | 0,042 | 0,36 | medium |
| Pathotype | nfvPPA_FTLD_vs_lvPPA_AD | chroma_mean_5 | 0,042 | 0,49 | medium |
| Pathotype | nfvPPA_FTLD_vs_lvPPA_AD | fricatives_percentile_90 | 0,042 | 0,49 | medium |
| Pathotype | FTLD_vs_amnestic_AD | jitter_rap | 0,042 | 0,33 | medium |
| Pathotype | svPPA_FTLD_vs_lvPPA_AD | kurt_phoneme_duration | 0,042 | 0,49 | medium |
| Pathotype | svPPA_FTLD_vs_lvPPA_AD | voyelles_oral_std | 0,042 | 0,49 | medium |
| Pathotype | FTLD_vs_amnestic_AD | f0_mean | 0,042 | 0,33 | medium |
| Pathotype | FTLD_vs_amnestic_AD | hnr_mean,1 | 0,042 | 0,33 | medium |
| Pathotype | amnestic_AD_vs_lvPPA_AD | b1 | 0,042 | 0,36 | medium |
| Pathotype | amnestic_AD_vs_lvPPA_AD | nasales_median | 0,043 | 0,36 | medium |
| Pathotype | amnestic_AD_vs_lvPPA_AD | occlusives_kurtosis | 0,043 | 0,36 | medium |
| Pathotype | FTLD_vs_lvPPA_FTLD | delta_mfcc_mean_10 | 0,043 | 0,52 | large |
| Pathotype | nfvPPA_FTLD_vs_lvPPA_FTLD | occlusives_kurtosis | 0,043 | 0,60 | large |
| Pathotype | FTLD_vs_lvPPA_FTLD | pause_proportion | 0,044 | 0,52 | large |
| Pathotype | nfvPPA_FTLD_vs_FTLD | semi_voyelles_percentile_10 | 0,044 | 0,46 | medium |
| Pathotype | svPPA_FTLD_vs_healthy_control | liquides_max | 0,044 | 0,39 | medium |
| Pathotype | amnestic_AD_vs_lvPPA_AD | pause_min_duration | 0,044 | 0,36 | medium |
| Pathotype | amnestic_AD_vs_lvPPA_AD | inter_word_pause_min | 0,044 | 0,36 | medium |
| Pathotype | nfvPPA_FTLD_vs_lvPPA_FTLD | contrast_mean_3 | 0,044 | 0,60 | large |
| Pathotype | nfvPPA_FTLD_vs_lvPPA_FTLD | b1 | 0,044 | 0,60 | large |
| Pathotype | FTLD_vs_lvPPA_AD | pause_max_duration | 0,044 | 0,39 | medium |
| Pathotype | FTLD_vs_lvPPA_AD | inter_word_pause_max | 0,044 | 0,39 | medium |
| Pathotype | svPPA_FTLD_vs_nfvPPA_FTLD | f2_cv | 0,044 | 0,54 | large |
| Pathotype | svPPA_FTLD_vs_nfvPPA_FTLD | chroma_mean_7 | 0,044 | 0,54 | large |
| Pathotype | svPPA_FTLD_vs_nfvPPA_FTLD | f2_cv,1 | 0,044 | 0,54 | large |
| Pathotype | svPPA_FTLD_vs_FTLD | chroma_mean_5 | 0,044 | 0,46 | medium |
| Pathotype | svPPA_FTLD_vs_lvPPA_FTLD | delta_mfcc_mean_6 | 0,044 | 0,60 | large |
| Pathotype | nfvPPA_FTLD_vs_healthy_control | max_phoneme_duration | 0,044 | 0,38 | medium |
| Pathotype | FTLD_vs_lvPPA_AD | speech_duration | 0,044 | 0,39 | medium |
| Pathotype | nfvPPA_FTLD_vs_lvPPA_FTLD | spectral_instability,1 | 0,045 | 0,60 | large |
| Pathotype | nfvPPA_FTLD_vs_lvPPA_FTLD | kurt_phoneme_duration | 0,045 | 0,60 | large |
| Pathotype | nfvPPA_FTLD_vs_healthy_control | b1 | 0,045 | 0,39 | medium |
| Pathotype | lvPPA_FTLD_vs_amnestic_AD | articulation_rate | 0,045 | 0,48 | medium |
| Pathotype | svPPA_FTLD_vs_nfvPPA_FTLD | fricatives_cv | 0,045 | 0,54 | large |
| Pathotype | nfvPPA_FTLD_vs_lvPPA_FTLD | pause_frequency | 0,045 | 0,60 | large |
| Pathotype | nfvPPA_FTLD_vs_lvPPA_FTLD | voyelles_oral_percentile_10 | 0,045 | 0,60 | large |
| Pathotype | amnestic_AD_vs_lvPPA_AD | nasales_skewness | 0,045 | 0,36 | medium |
| Pathotype | lvPPA_FTLD_vs_amnestic_AD | f1 | 0,045 | 0,49 | medium |
| Pathotype | svPPA_FTLD_vs_amnestic_AD | total_duration | 0,045 | 0,43 | medium |
| Pathotype | lvPPA_FTLD_vs_amnestic_AD | pause_cv | 0,045 | 0,49 | medium |
| Pathotype | lvPPA_FTLD_vs_amnestic_AD | pause_frequency | 0,045 | 0,49 | medium |
| Pathotype | lvPPA_FTLD_vs_amnestic_AD | voyelles_oral_cv | 0,045 | 0,49 | medium |
| Pathotype | svPPA_FTLD_vs_amnestic_AD | nasales_iqr | 0,046 | 0,43 | medium |
| Pathotype | svPPA_FTLD_vs_FTLD | semi_voyelles_kurtosis | 0,046 | 0,43 | medium |
| Pathotype | nfvPPA_FTLD_vs_amnestic_AD | contrast_mean_6 | 0,046 | 0,43 | medium |
| Pathotype | nfvPPA_FTLD_vs_healthy_control | occlusives_max | 0,046 | 0,38 | medium |
| Pathotype | nfvPPA_FTLD_vs_healthy_control | nasales_percentile_90 | 0,046 | 0,38 | medium |
| Pathotype | FTLD_vs_amnestic_AD | nasales_mean | 0,046 | 0,33 | medium |
| Pathotype | FTLD_vs_healthy_control | f1_cv,1 | 0,047 | 0,28 | small |
| Pathotype | amnestic_AD_vs_lvPPA_AD | pause_frequency | 0,047 | 0,36 | medium |
| Pathotype | amnestic_AD_vs_lvPPA_AD | speech_rate_phonemes_per_sec | 0,047 | 0,36 | medium |
| Pathotype | amnestic_AD_vs_lvPPA_AD | rate_speech_phonemes | 0,047 | 0,36 | medium |
| Pathotype | nfvPPA_FTLD_vs_lvPPA_AD | voyelles_oral_iqr | 0,047 | 0,48 | medium |
| Pathotype | svPPA_FTLD_vs_lvPPA_AD | liquides_kurtosis | 0,047 | 0,48 | medium |
| Pathotype | svPPA_FTLD_vs_lvPPA_AD | delta_mfcc_mean_7 | 0,048 | 0,48 | medium |
| Pathotype | svPPA_FTLD_vs_lvPPA_AD | nasales_iqr | 0,048 | 0,48 | medium |
| Pathotype | lvPPA_FTLD_vs_lvPPA_AD | fricatives_skewness | 0,048 | 0,54 | large |
| Pathotype | svPPA_FTLD_vs_lvPPA_AD | skew_phoneme_duration | 0,048 | 0,48 | medium |
| Pathotype | FTLD_vs_amnestic_AD | f1_cv | 0,048 | 0,32 | medium |
| Pathotype | lvPPA_FTLD_vs_lvPPA_AD | voyelles_oral_cv | 0,048 | 0,54 | large |
| Pathotype | lvPPA_FTLD_vs_lvPPA_AD | occlusives_cv | 0,048 | 0,54 | large |
| Pathotype | svPPA_FTLD_vs_amnestic_AD | chroma_mean_9 | 0,048 | 0,42 | medium |
| Pathotype | svPPA_FTLD_vs_amnestic_AD | zcr_mean | 0,049 | 0,42 | medium |
| Pathotype | FTLD_vs_lvPPA_FTLD | intensity_dynamic_range | 0,049 | 0,51 | large |
| Pathotype | nfvPPA_FTLD_vs_FTLD | fricatives_mean | 0,049 | 0,45 | medium |
| Pathotype | nfvPPA_FTLD_vs_FTLD | spectral_centroid_slope | 0,049 | 0,45 | medium |
| Pathotype | FTLD_vs_healthy_control | semi_voyelles_skewness | 0,049 | 0,28 | small |
| Pathotype | nfvPPA_FTLD_vs_FTLD | pause_ratio | 0,049 | 0,45 | medium |
| Pathotype | FTLD_vs_healthy_control | occlusives_max | 0,050 | 0,28 | small |
| Pathotype | FTLD_vs_amnestic_AD | jitter_ppq5 | 0,050 | 0,32 | medium |
| Physiotype | healthy_vs_AD | contrast_mean_0 | 0,000 | 0,92 | large |
| Physiotype | FTLD_vs_healthy | contrast_mean_0 | 0,000 | 0,85 | large |
| Physiotype | healthy_vs_AD | pause_median_duration | 0,000 | 0,82 | large |
| Physiotype | healthy_vs_AD | inter_word_pause_median | 0,000 | 0,82 | large |
| Physiotype | healthy_vs_AD | chroma_mean_8 | 0,000 | 0,77 | large |
| Physiotype | FTLD_vs_healthy | semi_voyelles_count | 0,000 | 0,74 | large |
| Physiotype | FTLD_vs_healthy | semi_voyelles_iqr | 0,000 | 0,73 | large |
| Physiotype | healthy_vs_AD | spectral_flux,1 | 0,000 | 0,73 | large |
| Physiotype | FTLD_vs_healthy | occlusives_count | 0,000 | 0,73 | large |
| Physiotype | healthy_vs_AD | jitter_local | 0,000 | 0,72 | large |
| Physiotype | FTLD_vs_healthy | contrast_mean_4 | 0,000 | 0,72 | large |
| Physiotype | FTLD_vs_healthy | phoneme_count | 0,000 | 0,71 | large |
| Physiotype | FTLD_vs_healthy | num_speech_phonemes | 0,000 | 0,71 | large |
| Physiotype | FTLD_vs_healthy | semi_voyelles_cv | 0,000 | 0,70 | large |
| Physiotype | healthy_vs_AD | zcr_mean | 0,000 | 0,70 | large |
| Physiotype | healthy_vs_AD | occlusives_count | 0,000 | 0,69 | large |
| Physiotype | healthy_vs_AD | contrast_mean_1 | 0,000 | 0,69 | large |
| Physiotype | healthy_vs_AD | spectral_centroid_std | 0,000 | 0,69 | large |
| Physiotype | FTLD_vs_healthy | semi_voyelles_percentile_90 | 0,000 | 0,69 | large |
| Physiotype | healthy_vs_AD | f3_cv | 0,000 | 0,68 | large |
| Physiotype | FTLD_vs_healthy | speech_duration | 0,000 | 0,68 | large |
| Physiotype | FTLD_vs_healthy | nasales_count | 0,000 | 0,67 | large |
| Physiotype | healthy_vs_AD | rolloff_std | 0,000 | 0,68 | large |
| Physiotype | FTLD_vs_healthy | shimmer_local | 0,000 | 0,67 | large |
| Physiotype | healthy_vs_AD | jitter_rap | 0,000 | 0,67 | large |
| Physiotype | healthy_vs_AD | liquides_count | 0,000 | 0,66 | large |
| Physiotype | FTLD_vs_healthy | semi_voyelles_max | 0,000 | 0,67 | large |
| Physiotype | FTLD_vs_healthy | delta_mfcc_mean_12 | 0,000 | 0,67 | large |
| Physiotype | FTLD_vs_healthy | fricatives_count | 0,000 | 0,66 | large |
| Physiotype | healthy_vs_AD | chroma_mean_7 | 0,000 | 0,66 | large |
| Physiotype | FTLD_vs_healthy | syllable_count | 0,000 | 0,66 | large |
| Physiotype | FTLD_vs_healthy | voyelles_oral_count | 0,000 | 0,66 | large |
| Physiotype | FTLD_vs_healthy | semi_voyelles_std | 0,000 | 0,65 | large |
| Physiotype | healthy_vs_AD | spectral_flux | 0,000 | 0,64 | large |
| Physiotype | healthy_vs_AD | fricatives_kurtosis | 0,000 | 0,64 | large |
| Physiotype | healthy_vs_AD | phoneme_count | 0,000 | 0,64 | large |
| Physiotype | healthy_vs_AD | num_speech_phonemes | 0,000 | 0,64 | large |
| Physiotype | FTLD_vs_healthy | shimmer_apq5 | 0,000 | 0,63 | large |
| Physiotype | healthy_vs_AD | semi_voyelles_count | 0,000 | 0,61 | large |
| Physiotype | healthy_vs_AD | pause_mean_duration | 0,000 | 0,62 | large |
| Physiotype | healthy_vs_AD | inter_word_pause_mean | 0,000 | 0,62 | large |
| Physiotype | healthy_vs_AD | jitter_ppq5 | 0,000 | 0,61 | large |
| Physiotype | healthy_vs_AD | contrast_mean_4 | 0,000 | 0,61 | large |
| Physiotype | FTLD_vs_healthy | semi_voyelles_mean | 0,000 | 0,60 | large |
| Physiotype | healthy_vs_AD | fricatives_count | 0,000 | 0,60 | large |
| Physiotype | FTLD_vs_healthy | pause_min_duration | 0,000 | 0,60 | large |
| Physiotype | FTLD_vs_healthy | inter_word_pause_min | 0,000 | 0,60 | large |
| Physiotype | FTLD_vs_healthy | fricatives_kurtosis | 0,000 | 0,60 | large |
| Physiotype | healthy_vs_AD | pause_ratio | 0,000 | 0,60 | large |
| Physiotype | healthy_vs_AD | pause_proportion | 0,000 | 0,59 | large |
| Physiotype | healthy_vs_AD | delta_mfcc_mean_12 | 0,000 | 0,59 | large |
| Physiotype | healthy_vs_AD | pause_total_duration | 0,000 | 0,59 | large |
| Physiotype | healthy_vs_AD | long_pause_count | 0,000 | 0,58 | large |
| Physiotype | healthy_vs_AD | syllable_count | 0,000 | 0,59 | large |
| Physiotype | healthy_vs_AD | voyelles_oral_count | 0,000 | 0,59 | large |
| Physiotype | FTLD_vs_healthy | pause_ratio | 0,000 | 0,58 | large |
| Physiotype | FTLD_vs_healthy | hnr_std | 0,000 | 0,58 | large |
| Physiotype | FTLD_vs_AD | spectral_flux,1 | 0,000 | 0,64 | large |
| Physiotype | FTLD_vs_healthy | semi_voyelles_median | 0,000 | 0,58 | large |
| Physiotype | FTLD_vs_AD | f0_mean | 0,000 | 0,64 | large |
| Physiotype | FTLD_vs_healthy | pause_proportion | 0,000 | 0,57 | large |
| Physiotype | FTLD_vs_AD | chroma_mean_8 | 0,000 | 0,62 | large |
| Physiotype | healthy_vs_AD | f0_min | 0,000 | 0,52 | large |
| Physiotype | healthy_vs_AD | contrast_mean_6 | 0,000 | 0,55 | large |
| Physiotype | healthy_vs_AD | fricatives_max | 0,000 | 0,55 | large |
| Physiotype | healthy_vs_AD | fricatives_skewness | 0,000 | 0,55 | large |
| Physiotype | FTLD_vs_healthy | intensity_dynamic_range | 0,000 | 0,54 | large |
| Physiotype | FTLD_vs_healthy | delta_mfcc_mean_1 | 0,000 | 0,54 | large |
| Physiotype | healthy_vs_AD | f3_cv,1 | 0,000 | 0,53 | large |
| Physiotype | healthy_vs_AD | pause_std_duration | 0,000 | 0,52 | large |
| Physiotype | healthy_vs_AD | inter_word_pause_std | 0,000 | 0,52 | large |
| Physiotype | healthy_vs_AD | nasales_iqr | 0,000 | 0,52 | large |
| Physiotype | FTLD_vs_AD | spectral_instability,1 | 0,000 | 0,57 | large |
| Physiotype | healthy_vs_AD | fricatives_iqr | 0,000 | 0,51 | large |
| Physiotype | healthy_vs_AD | nasales_std | 0,000 | 0,51 | large |
| Physiotype | FTLD_vs_healthy | rmse_mean | 0,000 | 0,51 | large |
| Physiotype | FTLD_vs_healthy | intensity_mean | 0,000 | 0,51 | large |
| Physiotype | FTLD_vs_AD | jitter_local | 0,000 | 0,56 | large |
| Physiotype | FTLD_vs_AD | shimmer_local | 0,000 | 0,56 | large |
| Physiotype | FTLD_vs_healthy | contrast_mean_3 | 0,000 | 0,50 | large |
| Physiotype | FTLD_vs_AD | shimmer_apq5 | 0,000 | 0,55 | large |
| Physiotype | FTLD_vs_healthy | f3_cv,1 | 0,000 | 0,50 | medium |
| Physiotype | FTLD_vs_AD | jitter_ppq5 | 0,000 | 0,55 | large |
| Physiotype | FTLD_vs_AD | hnr_mean | 0,000 | 0,55 | large |
| Physiotype | healthy_vs_AD | pause_max_duration | 0,000 | 0,50 | medium |
| Physiotype | healthy_vs_AD | inter_word_pause_max | 0,000 | 0,50 | medium |
| Physiotype | FTLD_vs_healthy | f3_cv | 0,000 | 0,50 | medium |
| Physiotype | healthy_vs_AD | chroma_mean_9 | 0,000 | 0,49 | medium |
| Physiotype | FTLD_vs_AD | jitter_rap | 0,000 | 0,54 | large |
| Physiotype | FTLD_vs_healthy | hnr_mean | 0,000 | 0,49 | medium |
| Physiotype | healthy_vs_AD | liquides_percentile_90 | 0,000 | 0,49 | medium |
| Physiotype | healthy_vs_AD | speech_duration | 0,000 | 0,49 | medium |
| Physiotype | FTLD_vs_healthy | fricatives_skewness | 0,000 | 0,49 | medium |
| Physiotype | healthy_vs_AD | hnr_std,1 | 0,000 | 0,49 | medium |
| Physiotype | FTLD_vs_AD | semi_voyelles_mean | 0,000 | 0,54 | large |
| Physiotype | FTLD_vs_healthy | liquides_count | 0,000 | 0,48 | medium |
| Physiotype | healthy_vs_AD | spectral_instability,1 | 0,000 | 0,48 | medium |
| Physiotype | FTLD_vs_AD | semi_voyelles_percentile_90 | 0,000 | 0,53 | large |
| Physiotype | FTLD_vs_healthy | num_inter_pauses | 0,000 | 0,48 | medium |
| Physiotype | healthy_vs_AD | fricatives_mean | 0,000 | 0,48 | medium |
| Physiotype | FTLD_vs_healthy | num_pauses | 0,000 | 0,48 | medium |
| Physiotype | FTLD_vs_healthy | num_inter_word_pauses | 0,000 | 0,48 | medium |
| Physiotype | FTLD_vs_healthy | shimmer_apq3 | 0,000 | 0,48 | medium |
| Physiotype | FTLD_vs_healthy | shimmer_dda | 0,000 | 0,48 | medium |
| Physiotype | FTLD_vs_healthy | semi_voyelles_skewness | 0,000 | 0,47 | medium |
| Physiotype | FTLD_vs_AD | rolloff_mean | 0,000 | 0,53 | large |
| Physiotype | FTLD_vs_healthy | delta_mfcc_mean_8 | 0,000 | 0,48 | medium |
| Physiotype | FTLD_vs_healthy | fricatives_max | 0,000 | 0,48 | medium |
| Physiotype | healthy_vs_AD | spectral_centroid_mean | 0,000 | 0,47 | medium |
| Physiotype | healthy_vs_AD | nasales_percentile_90 | 0,000 | 0,47 | medium |
| Physiotype | healthy_vs_AD | f1_cv,1 | 0,000 | 0,47 | medium |
| Physiotype | healthy_vs_AD | f0_mean | 0,000 | 0,46 | medium |
| Physiotype | healthy_vs_AD | rolloff_mean | 0,000 | 0,46 | medium |
| Physiotype | FTLD_vs_AD | semi_voyelles_iqr | 0,000 | 0,50 | medium |
| Physiotype | FTLD_vs_healthy | chroma_mean_7 | 0,000 | 0,45 | medium |
| Physiotype | FTLD_vs_healthy | f0_mean | 0,000 | 0,45 | medium |
| Physiotype | healthy_vs_AD | liquides_mean | 0,000 | 0,45 | medium |
| Physiotype | FTLD_vs_AD | semi_voyelles_max | 0,000 | 0,50 | medium |
| Physiotype | FTLD_vs_healthy | hnr_mean,1 | 0,000 | 0,45 | medium |
| Physiotype | FTLD_vs_AD | semi_voyelles_std | 0,000 | 0,49 | medium |
| Physiotype | FTLD_vs_AD | delta_mfcc_mean_11 | 0,000 | 0,50 | medium |
| Physiotype | FTLD_vs_healthy | f1_cv,1 | 0,000 | 0,45 | medium |
| Physiotype | FTLD_vs_AD | rolloff_std | 0,000 | 0,49 | medium |
| Physiotype | FTLD_vs_AD | semi_voyelles_cv | 0,000 | 0,48 | medium |
| Physiotype | FTLD_vs_healthy | rolloff_mean | 0,000 | 0,44 | medium |
| Physiotype | healthy_vs_AD | chroma_mean_6 | 0,000 | 0,44 | medium |
| Physiotype | FTLD_vs_AD | hnr_mean,1 | 0,000 | 0,48 | medium |
| Physiotype | healthy_vs_AD | pause_frequency | 0,000 | 0,44 | medium |
| Physiotype | healthy_vs_AD | fricatives_std | 0,000 | 0,43 | medium |
| Physiotype | FTLD_vs_healthy | occlusives_percentile_10 | 0,000 | 0,43 | medium |
| Physiotype | FTLD_vs_AD | shimmer_dda | 0,000 | 0,48 | medium |
| Physiotype | FTLD_vs_healthy | chroma_mean_2 | 0,000 | 0,43 | medium |
| Physiotype | FTLD_vs_AD | spectral_centroid_mean | 0,000 | 0,48 | medium |
| Physiotype | healthy_vs_AD | articulation_rate | 0,000 | 0,42 | medium |
| Physiotype | healthy_vs_AD | nasales_cv | 0,000 | 0,42 | medium |
| Physiotype | FTLD_vs_AD | semi_voyelles_median | 0,000 | 0,47 | medium |
| Physiotype | FTLD_vs_AD | long_pause_count | 0,000 | 0,46 | medium |
| Physiotype | FTLD_vs_healthy | semi_voyelles_kurtosis | 0,000 | 0,42 | medium |
| Physiotype | FTLD_vs_AD | spectral_centroid_std | 0,000 | 0,47 | medium |
| Physiotype | FTLD_vs_AD | hnr_std | 0,000 | 0,46 | medium |
| Physiotype | healthy_vs_AD | nasales_mean | 0,000 | 0,42 | medium |
| Physiotype | FTLD_vs_healthy | voyelles_oral_max | 0,000 | 0,41 | medium |
| Physiotype | healthy_vs_AD | voyelles_oral_median | 0,000 | 0,40 | medium |
| Physiotype | healthy_vs_AD | total_duration | 0,000 | 0,40 | medium |
| Physiotype | healthy_vs_AD | chroma_mean_1 | 0,000 | 0,40 | medium |
| Physiotype | FTLD_vs_AD | occlusives_count | 0,000 | 0,44 | medium |
| Physiotype | FTLD_vs_AD | nasales_count | 0,000 | 0,44 | medium |
| Physiotype | healthy_vs_AD | occlusives_mean | 0,000 | 0,40 | medium |
| Physiotype | FTLD_vs_AD | semi_voyelles_count | 0,000 | 0,44 | medium |
| Physiotype | healthy_vs_AD | b1 | 0,000 | 0,40 | medium |
| Physiotype | FTLD_vs_AD | chroma_mean_9 | 0,000 | 0,44 | medium |
| Physiotype | healthy_vs_AD | liquides_skewness | 0,000 | 0,39 | medium |
| Physiotype | healthy_vs_AD | fricatives_min | 0,000 | 0,38 | medium |
| Physiotype | FTLD_vs_AD | contrast_mean_3 | 0,000 | 0,44 | medium |
| Physiotype | FTLD_vs_healthy | pause_median_duration | 0,000 | 0,39 | medium |
| Physiotype | FTLD_vs_healthy | inter_word_pause_median | 0,000 | 0,39 | medium |
| Physiotype | FTLD_vs_AD | chroma_mean_10 | 0,000 | 0,43 | medium |
| Physiotype | healthy_vs_AD | pause_min_duration | 0,000 | 0,38 | medium |
| Physiotype | healthy_vs_AD | inter_word_pause_min | 0,000 | 0,38 | medium |
| Physiotype | FTLD_vs_healthy | delta_mfcc_mean_11 | 0,000 | 0,39 | medium |
| Physiotype | FTLD_vs_healthy | voyelles_oral_median | 0,000 | 0,38 | medium |
| Physiotype | FTLD_vs_AD | intensity_dynamic_range | 0,000 | 0,43 | medium |
| Physiotype | FTLD_vs_AD | delta_mfcc_mean_8 | 0,000 | 0,42 | medium |
| Physiotype | healthy_vs_AD | liquides_iqr | 0,000 | 0,38 | medium |
| Physiotype | FTLD_vs_healthy | delta_mfcc_mean_5 | 0,000 | 0,38 | medium |
| Physiotype | healthy_vs_AD | delta_mfcc_mean_4 | 0,000 | 0,38 | medium |
| Physiotype | FTLD_vs_healthy | zcr_mean | 0,000 | 0,38 | medium |
| Physiotype | FTLD_vs_healthy | liquides_skewness | 0,001 | 0,37 | medium |
| Physiotype | FTLD_vs_AD | chroma_mean_3 | 0,001 | 0,41 | medium |
| Physiotype | FTLD_vs_healthy | articulation_rate | 0,001 | 0,36 | medium |
| Physiotype | FTLD_vs_healthy | voyelles_oral_kurtosis | 0,001 | 0,36 | medium |
| Physiotype | FTLD_vs_AD | delta_mfcc_mean_1 | 0,001 | 0,40 | medium |
| Physiotype | FTLD_vs_healthy | voyelles_oral_skewness | 0,001 | 0,36 | medium |
| Physiotype | healthy_vs_AD | fricatives_percentile_90 | 0,001 | 0,36 | medium |
| Physiotype | FTLD_vs_healthy | hnr_std,1 | 0,001 | 0,36 | medium |
| Physiotype | FTLD_vs_AD | pause_total_duration | 0,001 | 0,40 | medium |
| Physiotype | healthy_vs_AD | semi_voyelles_min | 0,001 | 0,35 | medium |
| Physiotype | healthy_vs_AD | liquides_kurtosis | 0,001 | 0,36 | medium |
| Physiotype | FTLD_vs_healthy | liquides_cv | 0,001 | 0,35 | medium |
| Physiotype | FTLD_vs_healthy | fricatives_mean | 0,001 | 0,35 | medium |
| Physiotype | healthy_vs_AD | occlusives_median | 0,001 | 0,35 | medium |
| Physiotype | healthy_vs_AD | delta_mfcc_mean_3 | 0,001 | 0,35 | medium |
| Physiotype | FTLD_vs_AD | delta_mfcc_mean_12 | 0,001 | 0,38 | medium |
| Physiotype | FTLD_vs_healthy | contrast_mean_5 | 0,001 | 0,35 | medium |
| Physiotype | healthy_vs_AD | occlusives_percentile_90 | 0,001 | 0,35 | medium |
| Physiotype | healthy_vs_AD | b3 | 0,001 | 0,34 | medium |
| Physiotype | FTLD_vs_AD | fricatives_count | 0,001 | 0,38 | medium |
| Physiotype | FTLD_vs_healthy | chroma_mean_6 | 0,001 | 0,34 | medium |
| Physiotype | FTLD_vs_healthy | kurt_phoneme_duration | 0,001 | 0,34 | medium |
| Physiotype | FTLD_vs_AD | phoneme_count | 0,001 | 0,38 | medium |
| Physiotype | FTLD_vs_AD | num_speech_phonemes | 0,001 | 0,38 | medium |
| Physiotype | FTLD_vs_healthy | nasales_mean | 0,001 | 0,34 | medium |
| Physiotype | healthy_vs_AD | nasales_max | 0,001 | 0,34 | medium |
| Physiotype | healthy_vs_AD | occlusives_iqr | 0,002 | 0,34 | medium |
| Physiotype | FTLD_vs_healthy | nasales_skewness | 0,002 | 0,34 | medium |
| Physiotype | healthy_vs_AD | voyelles_oral_skewness | 0,002 | 0,34 | medium |
| Physiotype | FTLD_vs_healthy | contrast_mean_1 | 0,002 | 0,34 | medium |
| Physiotype | FTLD_vs_AD | contrast_mean_1 | 0,002 | 0,37 | medium |
| Physiotype | healthy_vs_AD | delta_mfcc_mean_1 | 0,002 | 0,33 | medium |
| Physiotype | healthy_vs_AD | semi_voyelles_iqr | 0,002 | 0,33 | medium |
| Physiotype | FTLD_vs_healthy | f0_slope | 0,002 | 0,33 | medium |
| Physiotype | FTLD_vs_AD | chroma_mean_2 | 0,002 | 0,37 | medium |
| Physiotype | FTLD_vs_AD | nasales_cv | 0,002 | 0,36 | medium |
| Physiotype | FTLD_vs_AD | f0_min | 0,002 | 0,31 | medium |
| Physiotype | FTLD_vs_AD | num_inter_pauses | 0,002 | 0,36 | medium |
| Physiotype | FTLD_vs_AD | num_pauses | 0,003 | 0,36 | medium |
| Physiotype | FTLD_vs_AD | num_inter_word_pauses | 0,003 | 0,36 | medium |
| Physiotype | FTLD_vs_healthy | min_phoneme_duration | 0,003 | 0,31 | medium |
| Physiotype | FTLD_vs_healthy | contrast_mean_6 | 0,003 | 0,32 | medium |
| Physiotype | FTLD_vs_AD | shimmer_apq3 | 0,003 | 0,35 | medium |
| Physiotype | healthy_vs_AD | f2_cv | 0,004 | 0,31 | medium |
| Physiotype | FTLD_vs_healthy | intensity_std | 0,004 | 0,31 | medium |
| Physiotype | FTLD_vs_healthy | nasales_iqr | 0,004 | 0,31 | medium |
| Physiotype | healthy_vs_AD | chroma_mean_4 | 0,004 | 0,31 | medium |
| Physiotype | healthy_vs_AD | delta_mfcc_mean_2 | 0,004 | 0,31 | medium |
| Physiotype | FTLD_vs_healthy | voyelles_oral_percentile_10 | 0,004 | 0,29 | small |
| Physiotype | healthy_vs_AD | median_phoneme_duration | 0,005 | 0,29 | small |
| Physiotype | FTLD_vs_AD | f0_std | 0,005 | 0,34 | medium |
| Physiotype | FTLD_vs_AD | nasales_percentile_10 | 0,005 | 0,33 | medium |
| Physiotype | FTLD_vs_healthy | fricatives_median | 0,005 | 0,30 | small |
| Physiotype | FTLD_vs_healthy | chroma_mean_3 | 0,005 | 0,30 | small |
| Physiotype | FTLD_vs_AD | speech_duration | 0,006 | 0,33 | medium |
| Physiotype | FTLD_vs_healthy | fricatives_iqr | 0,006 | 0,30 | small |
| Physiotype | healthy_vs_AD | occlusives_cv | 0,006 | 0,29 | small |
| Physiotype | FTLD_vs_healthy | total_duration | 0,007 | 0,29 | small |
| Physiotype | FTLD_vs_AD | f0_max | 0,007 | 0,32 | medium |
| Physiotype | healthy_vs_AD | semi_voyelles_skewness | 0,007 | 0,29 | small |
| Physiotype | FTLD_vs_healthy | occlusives_iqr | 0,007 | 0,29 | small |
| Physiotype | FTLD_vs_healthy | voyelles_oral_percentile_90 | 0,007 | 0,29 | small |
| Physiotype | FTLD_vs_AD | syllable_count | 0,008 | 0,32 | medium |
| Physiotype | FTLD_vs_AD | voyelles_oral_count | 0,008 | 0,32 | medium |
| Physiotype | healthy_vs_AD | semi_voyelles_kurtosis | 0,008 | 0,28 | small |
| Physiotype | FTLD_vs_healthy | liquides_min | 0,008 | 0,27 | small |
| Physiotype | healthy_vs_AD | nasales_min | 0,009 | 0,28 | small |
| Physiotype | healthy_vs_AD | chroma_mean_3 | 0,009 | 0,28 | small |
| Physiotype | FTLD_vs_AD | spectral_instability | 0,009 | 0,31 | medium |
| Physiotype | FTLD_vs_healthy | contrast_mean_2 | 0,009 | 0,28 | small |
| Physiotype | FTLD_vs_AD | contrast_mean_5 | 0,010 | 0,31 | medium |
| Physiotype | healthy_vs_AD | voyelles_oral_percentile_90 | 0,010 | 0,28 | small |
| Physiotype | FTLD_vs_healthy | voyelles_oral_cv | 0,010 | 0,28 | small |
| Physiotype | FTLD_vs_AD | intensity_std | 0,010 | 0,31 | medium |
| Physiotype | FTLD_vs_healthy | intensity_slope | 0,010 | 0,27 | small |
| Physiotype | FTLD_vs_healthy | pause_std_duration | 0,011 | 0,27 | small |
| Physiotype | FTLD_vs_healthy | inter_word_pause_std | 0,011 | 0,27 | small |
| Physiotype | FTLD_vs_healthy | b1 | 0,011 | 0,27 | small |
| Physiotype | FTLD_vs_healthy | fricatives_std | 0,012 | 0,27 | small |
| Physiotype | FTLD_vs_healthy | pause_mean_duration | 0,012 | 0,27 | small |
| Physiotype | FTLD_vs_healthy | inter_word_pause_mean | 0,012 | 0,27 | small |
| Physiotype | FTLD_vs_healthy | max_phoneme_duration | 0,012 | 0,27 | small |
| Physiotype | FTLD_vs_AD | fricatives_min | 0,012 | 0,29 | small |
| Physiotype | FTLD_vs_AD | min_phoneme_duration | 0,012 | 0,29 | small |
| Physiotype | FTLD_vs_healthy | f1_cv | 0,013 | 0,27 | small |
| Physiotype | FTLD_vs_AD | occlusives_kurtosis | 0,013 | 0,29 | small |
| Physiotype | FTLD_vs_healthy | fricatives_cv | 0,014 | 0,26 | small |
| Physiotype | FTLD_vs_healthy | nasales_percentile_90 | 0,014 | 0,26 | small |
| Physiotype | healthy_vs_AD | num_inter_pauses | 0,014 | 0,26 | small |
| Physiotype | FTLD_vs_AD | num_final_pauses | 0,015 | 0,17 | small |
| Physiotype | healthy_vs_AD | voyelles_oral_kurtosis | 0,015 | 0,26 | small |
| Physiotype | FTLD_vs_healthy | liquides_percentile_90 | 0,015 | 0,26 | small |
| Physiotype | healthy_vs_AD | fricatives_cv | 0,015 | 0,26 | small |
| Physiotype | FTLD_vs_healthy | delta_mfcc_mean_7 | 0,016 | 0,26 | small |
| Physiotype | FTLD_vs_healthy | delta_mfcc_mean_3 | 0,016 | 0,26 | small |
| Physiotype | FTLD_vs_healthy | nasales_median | 0,017 | 0,25 | small |
| Physiotype | healthy_vs_AD | f1_cv | 0,017 | 0,25 | small |
| Physiotype | FTLD_vs_healthy | occlusives_cv | 0,018 | 0,25 | small |
| Physiotype | healthy_vs_AD | contrast_mean_2 | 0,018 | 0,25 | small |
| Physiotype | FTLD_vs_healthy | occlusives_kurtosis | 0,018 | 0,25 | small |
| Physiotype | healthy_vs_AD | fricatives_percentile_10 | 0,019 | 0,23 | small |
| Physiotype | FTLD_vs_healthy | chroma_mean_4 | 0,019 | 0,25 | small |
| Physiotype | FTLD_vs_AD | voyelles_oral_kurtosis | 0,020 | 0,28 | small |
| Physiotype | FTLD_vs_healthy | nasales_kurtosis | 0,020 | 0,25 | small |
| Physiotype | FTLD_vs_AD | spectral_flux | 0,021 | 0,27 | small |
| Physiotype | FTLD_vs_AD | voyelles_oral_skewness | 0,021 | 0,27 | small |
| Physiotype | FTLD_vs_AD | nasales_skewness | 0,021 | 0,27 | small |
| Physiotype | healthy_vs_AD | spectral_centroid_slope | 0,021 | 0,25 | small |
| Physiotype | FTLD_vs_healthy | occlusives_skewness | 0,021 | 0,25 | small |
| Physiotype | healthy_vs_AD | num_pauses | 0,022 | 0,24 | small |
| Physiotype | healthy_vs_AD | num_inter_word_pauses | 0,022 | 0,24 | small |
| Physiotype | FTLD_vs_AD | occlusives_skewness | 0,022 | 0,27 | small |
| Physiotype | healthy_vs_AD | f0_slope | 0,023 | 0,24 | small |
| Physiotype | FTLD_vs_healthy | pause_max_duration | 0,025 | 0,24 | small |
| Physiotype | FTLD_vs_healthy | inter_word_pause_max | 0,025 | 0,24 | small |
| Physiotype | FTLD_vs_AD | occlusives_percentile_10 | 0,027 | 0,25 | small |
| Physiotype | FTLD_vs_healthy | occlusives_median | 0,027 | 0,23 | small |
| Physiotype | healthy_vs_AD | cv_phoneme_duration | 0,028 | 0,24 | small |
| Physiotype | FTLD_vs_healthy | f3 | 0,028 | 0,24 | small |
| Physiotype | FTLD_vs_AD | nasales_median | 0,028 | 0,26 | small |
| Physiotype | FTLD_vs_AD | chroma_mean_11 | 0,029 | 0,26 | small |
| Physiotype | healthy_vs_AD | intensity_slope | 0,029 | 0,23 | small |
| Physiotype | healthy_vs_AD | occlusives_min | 0,031 | 0,22 | small |
| Physiotype | healthy_vs_AD | speech_rate_phonemes_per_sec | 0,032 | 0,23 | small |
| Physiotype | healthy_vs_AD | rate_speech_phonemes | 0,032 | 0,23 | small |
| Physiotype | FTLD_vs_healthy | delta_mfcc_mean_9 | 0,032 | 0,23 | small |
| Physiotype | FTLD_vs_healthy | median_phoneme_duration | 0,032 | 0,22 | small |
| Physiotype | healthy_vs_AD | nasales_percentile_10 | 0,036 | 0,22 | small |
| Physiotype | healthy_vs_AD | voyelles_oral_percentile_10 | 0,036 | 0,20 | small |
| Physiotype | FTLD_vs_healthy | f1 | 0,037 | 0,22 | small |
| Physiotype | FTLD_vs_healthy | nasales_percentile_10 | 0,039 | 0,22 | small |
| Physiotype | healthy_vs_AD | hnr_std | 0,039 | 0,22 | small |
| Physiotype | FTLD_vs_AD | liquides_iqr | 0,040 | 0,24 | small |
| Physiotype | FTLD_vs_healthy | nasales_std | 0,042 | 0,22 | small |
| Physiotype | FTLD_vs_AD | nasales_min | 0,042 | 0,24 | small |
| Physiotype | FTLD_vs_AD | semi_voyelles_percentile_10 | 0,042 | 0,24 | small |
| Physiotype | FTLD_vs_AD | chroma_mean_7 | 0,043 | 0,24 | small |
| Physiotype | FTLD_vs_healthy | liquides_max | 0,043 | 0,22 | small |
| Physiotype | healthy_vs_AD | occlusives_std | 0,044 | 0,22 | small |
| Physiotype | healthy_vs_AD | fricatives_median | 0,044 | 0,21 | small |
| Physiotype | FTLD_vs_AD | voyelles_oral_max | 0,045 | 0,24 | small |
| Physiotype | FTLD_vs_healthy | occlusives_mean | 0,046 | 0,21 | small |
| Physiotype | healthy_vs_AD | liquides_cv | 0,047 | 0,21 | small |
| Physiotype | FTLD_vs_healthy | spectral_instability,1 | 0,048 | 0,21 | small |
| Physiotype | FTLD_vs_healthy | spectral_flux_std | 0,048 | 0,21 | small |
| Physiotype | FTLD_vs_healthy | occlusives_std | 0,049 | 0,21 | small |
| Physiotype | FTLD_vs_AD | delta_mfcc_mean_4 | 0,049 | 0,23 | small |
| Physiotype | FTLD_vs_AD | b1 | 0,049 | 0,23 | small |
| Physiotype | FTLD_vs_healthy | liquides_kurtosis | 0,050 | 0,21 | small |
| POS |  |  |  |  |  |
| Pathotype | healthy_control_vs_amnestic_AD | zcr_mean | 0,000 | 0,88 | large |
| Pathotype | healthy_control_vs_amnestic_AD | contrast_mean_0 | 0,000 | 0,88 | large |
| Pathotype | healthy_control_vs_amnestic_AD | spectral_flux | 0,000 | 0,80 | large |
| Pathotype | healthy_control_vs_amnestic_AD | spectral_flux,1 | 0,000 | 0,80 | large |
| Pathotype | FTLD_vs_healthy_control | contrast_mean_0 | 0,000 | 0,91 | large |
| Pathotype | healthy_control_vs_amnestic_AD | spectral_centroid_mean | 0,000 | 0,72 | large |
| Pathotype | healthy_control_vs_amnestic_AD | rolloff_mean | 0,000 | 0,71 | large |
| Pathotype | healthy_control_vs_lvPPA_AD | contrast_mean_0 | 0,000 | 1,00 | large |
| Pathotype | healthy_control_vs_lvPPA_AD | contrast_mean_4 | 0,000 | 0,98 | large |
| Pathotype | healthy_control_vs_amnestic_AD | spectral_centroid_std | 0,000 | 0,68 | large |
| Pathotype | healthy_control_vs_lvPPA_AD | voyelles_oral_count | 0,000 | 0,96 | large |
| Pathotype | healthy_control_vs_lvPPA_AD | phoneme_count | 0,000 | 0,96 | large |
| Pathotype | healthy_control_vs_lvPPA_AD | num_speech_phonemes | 0,000 | 0,96 | large |
| Pathotype | FTLD_vs_healthy_control | occlusives_mean | 0,000 | 0,81 | large |
| Pathotype | healthy_control_vs_lvPPA_AD | nasales_count | 0,000 | 0,95 | large |
| Pathotype | healthy_control_vs_lvPPA_AD | liquides_count | 0,000 | 0,94 | large |
| Pathotype | healthy_control_vs_amnestic_AD | liquides_mean | 0,000 | 0,66 | large |
| Pathotype | healthy_control_vs_amnestic_AD | liquides_percentile_90 | 0,000 | 0,66 | large |
| Pathotype | healthy_control_vs_amnestic_AD | rolloff_std | 0,000 | 0,66 | large |
| Pathotype | healthy_control_vs_lvPPA_AD | fricatives_count | 0,000 | 0,93 | large |
| Pathotype | healthy_control_vs_lvPPA_AD | pause_proportion | 0,000 | 0,93 | large |
| Pathotype | healthy_control_vs_lvPPA_AD | contrast_mean_1 | 0,000 | 0,92 | large |
| Pathotype | healthy_control_vs_amnestic_AD | fricatives_count | 0,000 | 0,65 | large |
| Pathotype | amnestic_AD_vs_lvPPA_AD | shimmer_local | 0,000 | 0,96 | large |
| Pathotype | healthy_control_vs_lvPPA_AD | pause_median_duration | 0,000 | 0,90 | large |
| Pathotype | healthy_control_vs_lvPPA_AD | inter_word_pause_median | 0,000 | 0,90 | large |
| Pathotype | healthy_control_vs_amnestic_AD | f1 | 0,000 | 0,63 | large |
| Pathotype | FTLD_vs_healthy_control | nasales_median | 0,000 | 0,75 | large |
| Pathotype | amnestic_AD_vs_lvPPA_AD | shimmer_apq5 | 0,000 | 0,94 | large |
| Pathotype | FTLD_vs_healthy_control | nasales_percentile_10 | 0,000 | 0,73 | large |
| Pathotype | FTLD_vs_healthy_control | occlusives_median | 0,000 | 0,73 | large |
| Pathotype | healthy_control_vs_amnestic_AD | contrast_mean_1 | 0,000 | 0,60 | large |
| Pathotype | amnestic_AD_vs_lvPPA_AD | nasales_count | 0,000 | 0,91 | large |
| Pathotype | healthy_control_vs_amnestic_AD | jitter_local | 0,000 | 0,60 | large |
| Pathotype | healthy_control_vs_lvPPA_AD | pause_min_duration | 0,000 | 0,84 | large |
| Pathotype | healthy_control_vs_lvPPA_AD | inter_word_pause_min | 0,000 | 0,84 | large |
| Pathotype | amnestic_AD_vs_lvPPA_AD | shimmer_apq3 | 0,000 | 0,91 | large |
| Pathotype | amnestic_AD_vs_lvPPA_AD | shimmer_dda | 0,000 | 0,91 | large |
| Pathotype | amnestic_AD_vs_lvPPA_AD | intensity_std | 0,000 | 0,91 | large |
| Pathotype | healthy_control_vs_amnestic_AD | liquides_median | 0,000 | 0,59 | large |
| Pathotype | healthy_control_vs_amnestic_AD | std_phoneme_duration | 0,000 | 0,59 | large |
| Pathotype | healthy_control_vs_lvPPA_AD | pause_mean_duration | 0,000 | 0,84 | large |
| Pathotype | amnestic_AD_vs_lvPPA_AD | intensity_dynamic_range | 0,000 | 0,90 | large |
| Pathotype | amnestic_AD_vs_lvPPA_AD | hnr_mean | 0,000 | 0,90 | large |
| Pathotype | amnestic_AD_vs_lvPPA_AD | hnr_mean,1 | 0,000 | 0,90 | large |
| Pathotype | FTLD_vs_healthy_control | occlusives_percentile_90 | 0,000 | 0,71 | large |
| Pathotype | healthy_control_vs_amnestic_AD | hnr_mean,1 | 0,000 | 0,59 | large |
| Pathotype | healthy_control_vs_lvPPA_AD | occlusives_count | 0,000 | 0,84 | large |
| Pathotype | amnestic_AD_vs_lvPPA_AD | fricatives_percentile_10 | 0,000 | 0,87 | large |
| Pathotype | amnestic_AD_vs_lvPPA_AD | delta_mfcc_mean_4 | 0,000 | 0,89 | large |
| Pathotype | healthy_control_vs_amnestic_AD | occlusives_mean | 0,000 | 0,58 | large |
| Pathotype | healthy_control_vs_amnestic_AD | max_phoneme_duration | 0,000 | 0,58 | large |
| Pathotype | healthy_control_vs_lvPPA_AD | intensity_dynamic_range | 0,000 | 0,83 | large |
| Pathotype | healthy_control_vs_amnestic_AD | chroma_mean_8 | 0,000 | 0,58 | large |
| Pathotype | healthy_control_vs_amnestic_AD | pause_max_duration | 0,000 | 0,58 | large |
| Pathotype | healthy_control_vs_amnestic_AD | inter_word_pause_max | 0,000 | 0,58 | large |
| Pathotype | healthy_control_vs_amnestic_AD | liquides_iqr | 0,000 | 0,58 | large |
| Pathotype | healthy_control_vs_lvPPA_AD | rmse_mean | 0,000 | 0,82 | large |
| Pathotype | healthy_control_vs_lvPPA_AD | intensity_mean | 0,000 | 0,82 | large |
| Pathotype | amnestic_AD_vs_lvPPA_AD | spectral_flux_std | 0,000 | 0,88 | large |
| Pathotype | healthy_control_vs_amnestic_AD | nasales_std | 0,000 | 0,57 | large |
| Pathotype | amnestic_AD_vs_lvPPA_AD | liquides_skewness | 0,000 | 0,87 | large |
| Pathotype | healthy_control_vs_amnestic_AD | hnr_mean | 0,000 | 0,57 | large |
| Pathotype | healthy_control_vs_lvPPA_AD | fricatives_percentile_10 | 0,000 | 0,81 | large |
| Pathotype | FTLD_vs_lvPPA_AD | contrast_mean_1 | 0,000 | 0,96 | large |
| Pathotype | FTLD_vs_lvPPA_AD | spectral_flux_mean | 0,000 | 0,96 | large |
| Pathotype | FTLD_vs_healthy_control | contrast_mean_4 | 0,000 | 0,69 | large |
| Pathotype | healthy_control_vs_amnestic_AD | cv_phoneme_duration | 0,000 | 0,56 | large |
| Pathotype | amnestic_AD_vs_lvPPA_AD | pause_proportion | 0,000 | 0,86 | large |
| Pathotype | healthy_control_vs_amnestic_AD | nasales_max | 0,000 | 0,56 | large |
| Pathotype | healthy_control_vs_lvPPA_AD | chroma_mean_1 | 0,000 | 0,80 | large |
| Pathotype | healthy_control_vs_lvPPA_AD | pause_total_duration | 0,000 | 0,80 | large |
| Pathotype | amnestic_AD_vs_lvPPA_AD | rmse_mean | 0,000 | 0,85 | large |
| Pathotype | amnestic_AD_vs_lvPPA_AD | intensity_mean | 0,000 | 0,85 | large |
| Pathotype | amnestic_AD_vs_lvPPA_AD | phoneme_count | 0,000 | 0,84 | large |
| Pathotype | amnestic_AD_vs_lvPPA_AD | num_speech_phonemes | 0,000 | 0,84 | large |
| Pathotype | healthy_control_vs_amnestic_AD | occlusives_std | 0,000 | 0,55 | large |
| Pathotype | healthy_control_vs_amnestic_AD | pause_cv | 0,000 | 0,55 | large |
| Pathotype | svPPA_FTLD_vs_amnestic_AD | delta_mfcc_mean_4 | 0,000 | 0,96 | large |
| Pathotype | FTLD_vs_lvPPA_AD | chroma_mean_3 | 0,000 | 0,93 | large |
| Pathotype | amnestic_AD_vs_lvPPA_AD | liquides_count | 0,000 | 0,84 | large |
| Pathotype | svPPA_FTLD_vs_healthy_control | contrast_mean_4 | 0,000 | 0,90 | large |
| Pathotype | healthy_control_vs_amnestic_AD | pause_std_duration | 0,000 | 0,55 | large |
| Pathotype | healthy_control_vs_amnestic_AD | inter_word_pause_std | 0,000 | 0,55 | large |
| Pathotype | FTLD_vs_lvPPA_AD | shimmer_local | 0,000 | 0,92 | large |
| Pathotype | FTLD_vs_lvPPA_AD | shimmer_apq5 | 0,000 | 0,92 | large |
| Pathotype | amnestic_AD_vs_lvPPA_AD | occlusives_count | 0,000 | 0,83 | large |
| Pathotype | healthy_control_vs_lvPPA_AD | spectral_flux_std | 0,000 | 0,77 | large |
| Pathotype | amnestic_AD_vs_lvPPA_AD | contrast_mean_2 | 0,000 | 0,83 | large |
| Pathotype | healthy_control_vs_lvPPA_AD | spectral_flux_mean | 0,000 | 0,77 | large |
| Pathotype | FTLD_vs_lvPPA_AD | voyelles_oral_count | 0,000 | 0,90 | large |
| Pathotype | amnestic_AD_vs_lvPPA_AD | contrast_mean_4 | 0,000 | 0,82 | large |
| Pathotype | FTLD_vs_lvPPA_AD | chroma_mean_2 | 0,000 | 0,90 | large |
| Pathotype | amnestic_AD_vs_lvPPA_AD | articulation_rate | 0,000 | 0,80 | large |
| Pathotype | FTLD_vs_lvPPA_AD | phoneme_count | 0,000 | 0,90 | large |
| Pathotype | healthy_control_vs_lvPPA_AD | intensity_std | 0,000 | 0,76 | large |
| Pathotype | FTLD_vs_lvPPA_AD | pause_proportion | 0,000 | 0,90 | large |
| Pathotype | healthy_control_vs_amnestic_AD | nasales_kurtosis | 0,000 | 0,53 | large |
| Pathotype | nfvPPA_FTLD_vs_healthy_control | occlusives_median | 0,000 | 1,00 | large |
| Pathotype | amnestic_AD_vs_lvPPA_AD | f2_cv,1 | 0,000 | 0,81 | large |
| Pathotype | FTLD_vs_lvPPA_AD | shimmer_apq3 | 0,000 | 0,89 | large |
| Pathotype | FTLD_vs_lvPPA_AD | shimmer_dda | 0,000 | 0,89 | large |
| Pathotype | healthy_control_vs_lvPPA_AD | inter_word_pause_mean | 0,000 | 0,75 | large |
| Pathotype | healthy_control_vs_lvPPA_AD | pause_ratio | 0,000 | 0,75 | large |
| Pathotype | FTLD_vs_lvPPA_AD | liquides_skewness | 0,000 | 0,89 | large |
| Pathotype | healthy_control_vs_amnestic_AD | contrast_mean_2 | 0,000 | 0,53 | large |
| Pathotype | amnestic_AD_vs_lvPPA_AD | voyelles_oral_count | 0,000 | 0,80 | large |
| Pathotype | amnestic_AD_vs_lvPPA_AD | nasales_kurtosis | 0,000 | 0,80 | large |
| Pathotype | FTLD_vs_lvPPA_AD | num_speech_phonemes | 0,000 | 0,88 | large |
| Pathotype | healthy_control_vs_amnestic_AD | pause_mean_duration | 0,000 | 0,53 | large |
| Pathotype | healthy_control_vs_amnestic_AD | contrast_mean_4 | 0,000 | 0,53 | large |
| Pathotype | healthy_control_vs_amnestic_AD | nasales_cv | 0,000 | 0,52 | large |
| Pathotype | healthy_control_vs_amnestic_AD | mean_phoneme_duration | 0,000 | 0,52 | large |
| Pathotype | FTLD_vs_lvPPA_AD | fricatives_count | 0,000 | 0,88 | large |
| Pathotype | lvPPA_FTLD_vs_healthy_control | semi_voyelles_count | 0,000 | 1,00 | large |
| Pathotype | lvPPA_FTLD_vs_healthy_control | liquides_count | 0,000 | 1,00 | large |
| Pathotype | lvPPA_FTLD_vs_healthy_control | num_pauses | 0,000 | 1,00 | large |
| Pathotype | lvPPA_FTLD_vs_healthy_control | num_inter_word_pauses | 0,000 | 1,00 | large |
| Pathotype | lvPPA_FTLD_vs_healthy_control | occlusives_max | 0,000 | 1,00 | large |
| Pathotype | lvPPA_FTLD_vs_healthy_control | num_inter_pauses | 0,000 | 1,00 | large |
| Pathotype | lvPPA_FTLD_vs_healthy_control | nasales_count | 0,000 | 1,00 | large |
| Pathotype | lvPPA_FTLD_vs_healthy_control | nasales_max | 0,000 | 1,00 | large |
| Pathotype | nfvPPA_FTLD_vs_healthy_control | occlusives_percentile_90 | 0,000 | 1,00 | large |
| Pathotype | lvPPA_FTLD_vs_healthy_control | fricatives_count | 0,000 | 1,00 | large |
| Pathotype | lvPPA_FTLD_vs_healthy_control | occlusives_count | 0,000 | 1,00 | large |
| Pathotype | lvPPA_FTLD_vs_healthy_control | syllable_count | 0,000 | 1,00 | large |
| Pathotype | lvPPA_FTLD_vs_healthy_control | voyelles_oral_count | 0,000 | 1,00 | large |
| Pathotype | lvPPA_FTLD_vs_healthy_control | nasales_iqr | 0,000 | 1,00 | large |
| Pathotype | lvPPA_FTLD_vs_healthy_control | liquides_max | 0,000 | 1,00 | large |
| Pathotype | lvPPA_FTLD_vs_healthy_control | nasales_std | 0,000 | 1,00 | large |
| Pathotype | lvPPA_FTLD_vs_healthy_control | nasales_cv | 0,000 | 1,00 | large |
| Pathotype | lvPPA_FTLD_vs_healthy_control | voyelles_oral_kurtosis | 0,000 | 1,00 | large |
| Pathotype | lvPPA_FTLD_vs_healthy_control | phoneme_count | 0,000 | 1,00 | large |
| Pathotype | lvPPA_FTLD_vs_healthy_control | num_speech_phonemes | 0,000 | 1,00 | large |
| Pathotype | nfvPPA_FTLD_vs_healthy_control | occlusives_mean | 0,000 | 1,00 | large |
| Pathotype | lvPPA_FTLD_vs_healthy_control | liquides_percentile_90 | 0,000 | 1,00 | large |
| Pathotype | lvPPA_FTLD_vs_healthy_control | voyelles_oral_skewness | 0,000 | 1,00 | large |
| Pathotype | lvPPA_FTLD_vs_healthy_control | kurt_phoneme_duration | 0,000 | 1,00 | large |
| Pathotype | lvPPA_FTLD_vs_healthy_control | speech_duration | 0,000 | 1,00 | large |
| Pathotype | lvPPA_FTLD_vs_healthy_control | contrast_mean_0 | 0,000 | 1,00 | large |
| Pathotype | lvPPA_FTLD_vs_healthy_control | contrast_mean_4 | 0,000 | 1,00 | large |
| Pathotype | lvPPA_FTLD_vs_healthy_control | rmse_mean | 0,000 | 1,00 | large |
| Pathotype | lvPPA_FTLD_vs_healthy_control | rolloff_mean | 0,000 | 1,00 | large |
| Pathotype | lvPPA_FTLD_vs_healthy_control | intensity_mean | 0,000 | 1,00 | large |
| Pathotype | nfvPPA_FTLD_vs_healthy_control | shimmer_local | 0,000 | 1,00 | large |
| Pathotype | nfvPPA_FTLD_vs_healthy_control | shimmer_apq5 | 0,000 | 1,00 | large |
| Pathotype | nfvPPA_FTLD_vs_healthy_control | hnr_mean | 0,000 | 1,00 | large |
| Pathotype | nfvPPA_FTLD_vs_healthy_control | hnr_mean,1 | 0,000 | 1,00 | large |
| Pathotype | healthy_control_vs_lvPPA_AD | shimmer_apq5 | 0,000 | 0,74 | large |
| Pathotype | healthy_control_vs_lvPPA_AD | speech_duration | 0,000 | 0,74 | large |
| Pathotype | lvPPA_FTLD_vs_healthy_control | voyelles_oral_percentile_10 | 0,000 | 0,91 | large |
| Pathotype | lvPPA_FTLD_vs_healthy_control | liquides_iqr | 0,000 | 0,99 | large |
| Pathotype | nfvPPA_FTLD_vs_healthy_control | liquides_iqr | 0,000 | 0,99 | large |
| Pathotype | lvPPA_FTLD_vs_healthy_control | total_duration | 0,000 | 0,99 | large |
| Pathotype | lvPPA_FTLD_vs_healthy_control | liquides_std | 0,000 | 0,99 | large |
| Pathotype | lvPPA_FTLD_vs_healthy_control | liquides_cv | 0,000 | 0,99 | large |
| Pathotype | lvPPA_FTLD_vs_healthy_control | f2_cv | 0,000 | 0,99 | large |
| Pathotype | lvPPA_FTLD_vs_healthy_control | contrast_mean_3 | 0,000 | 0,99 | large |
| Pathotype | FTLD_vs_lvPPA_AD | fricatives_percentile_10 | 0,000 | 0,86 | large |
| Pathotype | healthy_control_vs_lvPPA_AD | articulation_rate | 0,000 | 0,71 | large |
| Pathotype | lvPPA_FTLD_vs_healthy_control | semi_voyelles_max | 0,000 | 0,98 | large |
| Pathotype | lvPPA_FTLD_vs_healthy_control | semi_voyelles_percentile_90 | 0,000 | 0,98 | large |
| Pathotype | lvPPA_FTLD_vs_healthy_control | semi_voyelles_mean | 0,000 | 0,98 | large |
| Pathotype | lvPPA_FTLD_vs_healthy_control | chroma_mean_1 | 0,000 | 0,98 | large |
| Pathotype | lvPPA_FTLD_vs_healthy_control | spectral_centroid_mean | 0,000 | 0,98 | large |
| Pathotype | nfvPPA_FTLD_vs_healthy_control | shimmer_apq3 | 0,000 | 0,98 | large |
| Pathotype | nfvPPA_FTLD_vs_healthy_control | shimmer_dda | 0,000 | 0,98 | large |
| Pathotype | nfvPPA_FTLD_vs_healthy_control | occlusives_iqr | 0,000 | 0,98 | large |
| Pathotype | healthy_control_vs_lvPPA_AD | shimmer_apq3 | 0,000 | 0,72 | large |
| Pathotype | healthy_control_vs_lvPPA_AD | shimmer_dda | 0,000 | 0,72 | large |
| Pathotype | FTLD_vs_amnestic_AD | b1 | 0,000 | 0,67 | large |
| Pathotype | FTLD_vs_healthy_control | chroma_mean_2 | 0,000 | 0,61 | large |
| Pathotype | nfvPPA_FTLD_vs_healthy_control | occlusives_std | 0,000 | 0,97 | large |
| Pathotype | lvPPA_FTLD_vs_healthy_control | occlusives_min | 0,000 | 0,94 | large |
| Pathotype | FTLD_vs_amnestic_AD | nasales_min | 0,000 | 0,66 | large |
| Pathotype | amnestic_AD_vs_lvPPA_AD | contrast_mean_0 | 0,000 | 0,77 | large |
| Pathotype | svPPA_FTLD_vs_amnestic_AD | rolloff_mean | 0,000 | 0,87 | large |
| Pathotype | lvPPA_FTLD_vs_amnestic_AD | semi_voyelles_iqr | 0,000 | 1,00 | large |
| Pathotype | healthy_control_vs_amnestic_AD | liquides_std | 0,000 | 0,50 | large |
| Pathotype | healthy_control_vs_amnestic_AD | occlusives_max | 0,000 | 0,50 | large |
| Pathotype | amnestic_AD_vs_lvPPA_AD | spectral_flux_mean | 0,000 | 0,77 | large |
| Pathotype | FTLD_vs_lvPPA_AD | occlusives_count | 0,000 | 0,84 | large |
| Pathotype | nfvPPA_FTLD_vs_healthy_control | phoneme_count | 0,000 | 0,97 | large |
| Pathotype | nfvPPA_FTLD_vs_healthy_control | num_speech_phonemes | 0,000 | 0,97 | large |
| Pathotype | lvPPA_FTLD_vs_amnestic_AD | liquides_std | 0,000 | 1,00 | large |
| Pathotype | lvPPA_FTLD_vs_healthy_control | max_phoneme_duration | 0,000 | 0,97 | large |
| Pathotype | lvPPA_FTLD_vs_amnestic_AD | syllable_count | 0,000 | 1,00 | large |
| Pathotype | lvPPA_FTLD_vs_amnestic_AD | liquides_mean | 0,000 | 1,00 | large |
| Pathotype | FTLD_vs_lvPPA_AD | spectral_flux_std | 0,000 | 0,84 | large |
| Pathotype | lvPPA_FTLD_vs_amnestic_AD | num_inter_word_pauses | 0,000 | 1,00 | large |
| Pathotype | nfvPPA_FTLD_vs_amnestic_AD | occlusives_median | 0,000 | 1,00 | large |
| Pathotype | lvPPA_FTLD_vs_amnestic_AD | nasales_std | 0,000 | 1,00 | large |
| Pathotype | lvPPA_FTLD_vs_amnestic_AD | liquides_iqr | 0,000 | 1,00 | large |
| Pathotype | lvPPA_FTLD_vs_amnestic_AD | rolloff_mean | 0,000 | 1,00 | large |
| Pathotype | nfvPPA_FTLD_vs_amnestic_AD | rolloff_mean | 0,000 | 1,00 | large |
| Pathotype | lvPPA_FTLD_vs_amnestic_AD | total_duration | 0,000 | 1,00 | large |
| Pathotype | lvPPA_FTLD_vs_amnestic_AD | semi_voyelles_std | 0,000 | 1,00 | large |
| Pathotype | lvPPA_FTLD_vs_amnestic_AD | semi_voyelles_cv | 0,000 | 1,00 | large |
| Pathotype | lvPPA_FTLD_vs_amnestic_AD | num_speech_phonemes | 0,000 | 1,00 | large |
| Pathotype | lvPPA_FTLD_vs_amnestic_AD | num_pauses | 0,000 | 1,00 | large |
| Pathotype | amnestic_AD_vs_lvPPA_AD | nasales_skewness | 0,000 | 0,76 | large |
| Pathotype | lvPPA_FTLD_vs_amnestic_AD | nasales_iqr | 0,000 | 1,00 | large |
| Pathotype | lvPPA_FTLD_vs_amnestic_AD | voyelles_oral_kurtosis | 0,000 | 1,00 | large |
| Pathotype | nfvPPA_FTLD_vs_amnestic_AD | semi_voyelles_percentile_90 | 0,000 | 1,00 | large |
| Pathotype | nfvPPA_FTLD_vs_amnestic_AD | occlusives_percentile_90 | 0,000 | 1,00 | large |
| Pathotype | lvPPA_FTLD_vs_amnestic_AD | contrast_mean_3 | 0,000 | 1,00 | large |
| Pathotype | lvPPA_FTLD_vs_amnestic_AD | spectral_centroid_mean | 0,000 | 1,00 | large |
| Pathotype | lvPPA_FTLD_vs_amnestic_AD | rmse_mean | 0,000 | 1,00 | large |
| Pathotype | lvPPA_FTLD_vs_amnestic_AD | intensity_mean | 0,000 | 1,00 | large |
| Pathotype | nfvPPA_FTLD_vs_amnestic_AD | hnr_mean | 0,000 | 1,00 | large |
| Pathotype | nfvPPA_FTLD_vs_amnestic_AD | hnr_mean,1 | 0,000 | 1,00 | large |
| Pathotype | lvPPA_FTLD_vs_amnestic_AD | speech_duration | 0,000 | 1,00 | large |
| Pathotype | lvPPA_FTLD_vs_amnestic_AD | liquides_cv | 0,000 | 0,99 | large |
| Pathotype | healthy_control_vs_amnestic_AD | jitter_rap | 0,000 | 0,50 | medium |
| Pathotype | nfvPPA_FTLD_vs_healthy_control | syllable_count | 0,000 | 0,96 | large |
| Pathotype | nfvPPA_FTLD_vs_healthy_control | voyelles_oral_count | 0,000 | 0,96 | large |
| Pathotype | lvPPA_FTLD_vs_healthy_control | occlusives_iqr | 0,000 | 0,96 | large |
| Pathotype | lvPPA_FTLD_vs_healthy_control | chroma_mean_4 | 0,000 | 0,96 | large |
| Pathotype | FTLD_vs_lvPPA_AD | nasales_count | 0,000 | 0,83 | large |
| Pathotype | lvPPA_FTLD_vs_amnestic_AD | median_phoneme_duration | 0,000 | 0,98 | large |
| Pathotype | lvPPA_FTLD_vs_healthy_control | min_phoneme_duration | 0,000 | 0,92 | large |
| Pathotype | lvPPA_FTLD_vs_amnestic_AD | num_inter_pauses | 0,000 | 0,99 | large |
| Pathotype | lvPPA_FTLD_vs_amnestic_AD | nasales_cv | 0,000 | 0,99 | large |
| Pathotype | FTLD_vs_amnestic_AD | spectral_centroid_std | 0,000 | 0,66 | large |
| Pathotype | nfvPPA_FTLD_vs_healthy_control | fricatives_count | 0,000 | 0,95 | large |
| Pathotype | lvPPA_FTLD_vs_healthy_control | occlusives_std | 0,000 | 0,95 | large |
| Pathotype | healthy_control_vs_lvPPA_AD | long_pause_count | 0,000 | 0,70 | large |
| Pathotype | healthy_control_vs_amnestic_AD | nasales_skewness | 0,000 | 0,49 | medium |
| Pathotype | amnestic_AD_vs_lvPPA_AD | f2_cv | 0,000 | 0,75 | large |
| Pathotype | lvPPA_FTLD_vs_healthy_control | occlusives_percentile_90 | 0,000 | 0,95 | large |
| Pathotype | svPPA_FTLD_vs_healthy_control | chroma_mean_0 | 0,000 | 0,81 | large |
| Pathotype | lvPPA_FTLD_vs_healthy_control | fricatives_max | 0,000 | 0,95 | large |
| Pathotype | lvPPA_FTLD_vs_healthy_control | liquides_mean | 0,000 | 0,95 | large |
| Pathotype | lvPPA_FTLD_vs_amnestic_AD | voyelles_oral_count | 0,000 | 0,98 | large |
| Pathotype | svPPA_FTLD_vs_lvPPA_AD | chroma_mean_1 | 0,000 | 1,00 | large |
| Pathotype | lvPPA_FTLD_vs_amnestic_AD | zcr_mean | 0,000 | 0,98 | large |
| Pathotype | lvPPA_FTLD_vs_healthy_control | semi_voyelles_median | 0,000 | 0,94 | large |
| Pathotype | lvPPA_FTLD_vs_amnestic_AD | occlusives_iqr | 0,000 | 0,98 | large |
| Pathotype | lvPPA_FTLD_vs_amnestic_AD | chroma_mean_4 | 0,000 | 0,98 | large |
| Pathotype | svPPA_FTLD_vs_healthy_control | occlusives_iqr | 0,000 | 0,80 | large |
| Pathotype | healthy_control_vs_amnestic_AD | chroma_mean_7 | 0,000 | 0,49 | medium |
| Pathotype | lvPPA_FTLD_vs_healthy_control | semi_voyelles_iqr | 0,000 | 0,94 | large |
| Pathotype | lvPPA_FTLD_vs_healthy_control | semi_voyelles_std | 0,000 | 0,94 | large |
| Pathotype | lvPPA_FTLD_vs_healthy_control | semi_voyelles_cv | 0,000 | 0,94 | large |
| Pathotype | nfvPPA_FTLD_vs_healthy_control | contrast_mean_0 | 0,000 | 0,94 | large |
| Pathotype | FTLD_vs_healthy_control | nasales_mean | 0,000 | 0,59 | large |
| Pathotype | lvPPA_FTLD_vs_healthy_control | pause_min_duration | 0,000 | 0,93 | large |
| Pathotype | lvPPA_FTLD_vs_healthy_control | inter_word_pause_min | 0,000 | 0,93 | large |
| Pathotype | svPPA_FTLD_vs_lvPPA_AD | pause_proportion | 0,000 | 0,99 | large |
| Pathotype | svPPA_FTLD_vs_healthy_control | intensity_std | 0,000 | 0,80 | large |
| Pathotype | svPPA_FTLD_vs_amnestic_AD | liquides_median | 0,000 | 0,84 | large |
| Pathotype | healthy_control_vs_lvPPA_AD | jitter_local | 0,000 | 0,69 | large |
| Pathotype | healthy_control_vs_lvPPA_AD | shimmer_local | 0,000 | 0,69 | large |
| Pathotype | healthy_control_vs_amnestic_AD | speech_rate_phonemes_per_sec | 0,000 | 0,48 | medium |
| Pathotype | FTLD_vs_lvPPA_AD | liquides_count | 0,000 | 0,81 | large |
| Pathotype | svPPA_FTLD_vs_lvPPA_AD | b3 | 0,000 | 0,99 | large |
| Pathotype | svPPA_FTLD_vs_amnestic_AD | chroma_mean_0 | 0,000 | 0,84 | large |
| Pathotype | healthy_control_vs_lvPPA_AD | hnr_mean | 0,000 | 0,69 | large |
| Pathotype | healthy_control_vs_lvPPA_AD | hnr_mean,1 | 0,000 | 0,69 | large |
| Pathotype | lvPPA_FTLD_vs_amnestic_AD | nasales_max | 0,000 | 0,97 | large |
| Pathotype | healthy_control_vs_amnestic_AD | semi_voyelles_min | 0,000 | 0,47 | medium |
| Pathotype | FTLD_vs_amnestic_AD | articulation_rate | 0,000 | 0,63 | large |
| Pathotype | lvPPA_FTLD_vs_amnestic_AD | voyelles_oral_percentile_10 | 0,000 | 0,91 | large |
| Pathotype | nfvPPA_FTLD_vs_amnestic_AD | semi_voyelles_std | 0,000 | 0,97 | large |
| Pathotype | lvPPA_FTLD_vs_amnestic_AD | semi_voyelles_percentile_90 | 0,000 | 0,97 | large |
| Pathotype | lvPPA_FTLD_vs_amnestic_AD | liquides_median | 0,000 | 0,97 | large |
| Pathotype | nfvPPA_FTLD_vs_amnestic_AD | f0_mean | 0,000 | 0,97 | large |
| Pathotype | lvPPA_FTLD_vs_amnestic_AD | f2_cv | 0,000 | 0,97 | large |
| Pathotype | lvPPA_FTLD_vs_amnestic_AD | contrast_mean_0 | 0,000 | 0,97 | large |
| Pathotype | nfvPPA_FTLD_vs_amnestic_AD | spectral_centroid_mean | 0,000 | 0,97 | large |
| Pathotype | healthy_control_vs_lvPPA_AD | jitter_rap | 0,000 | 0,69 | large |
| Pathotype | healthy_control_vs_lvPPA_AD | jitter_ppq5 | 0,000 | 0,69 | large |
| Pathotype | lvPPA_FTLD_vs_amnestic_AD | phoneme_count | 0,000 | 0,96 | large |
| Pathotype | svPPA_FTLD_vs_amnestic_AD | spectral_instability | 0,000 | 0,83 | large |
| Pathotype | svPPA_FTLD_vs_amnestic_AD | spectral_instability,1 | 0,000 | 0,83 | large |
| Pathotype | lvPPA_FTLD_vs_amnestic_AD | nasales_count | 0,000 | 0,96 | large |
| Pathotype | lvPPA_FTLD_vs_amnestic_AD | liquides_skewness | 0,000 | 0,96 | large |
| Pathotype | lvPPA_FTLD_vs_amnestic_AD | kurt_phoneme_duration | 0,000 | 0,96 | large |
| Pathotype | svPPA_FTLD_vs_amnestic_AD | liquides_mean | 0,000 | 0,83 | large |
| Pathotype | lvPPA_FTLD_vs_amnestic_AD | intensity_dynamic_range | 0,000 | 0,96 | large |
| Pathotype | lvPPA_FTLD_vs_amnestic_AD | liquides_percentile_90 | 0,000 | 0,96 | large |
| Pathotype | lvPPA_FTLD_vs_amnestic_AD | nasales_percentile_90 | 0,000 | 0,96 | large |
| Pathotype | nfvPPA_FTLD_vs_amnestic_AD | shimmer_apq3 | 0,000 | 0,96 | large |
| Pathotype | nfvPPA_FTLD_vs_amnestic_AD | shimmer_dda | 0,000 | 0,96 | large |
| Pathotype | lvPPA_FTLD_vs_amnestic_AD | skew_phoneme_duration | 0,000 | 0,96 | large |
| Pathotype | healthy_control_vs_amnestic_AD | shimmer_local | 0,000 | 0,48 | medium |
| Pathotype | healthy_control_vs_amnestic_AD | semi_voyelles_mean | 0,000 | 0,48 | medium |
| Pathotype | healthy_control_vs_lvPPA_AD | pause_std_duration | 0,000 | 0,68 | large |
| Pathotype | healthy_control_vs_lvPPA_AD | inter_word_pause_std | 0,000 | 0,68 | large |
| Pathotype | lvPPA_FTLD_vs_amnestic_AD | long_pause_count | 0,000 | 0,95 | large |
| Pathotype | nfvPPA_FTLD_vs_healthy_control | occlusives_count | 0,000 | 0,92 | large |
| Pathotype | lvPPA_FTLD_vs_healthy_control | voyelles_oral_max | 0,000 | 0,92 | large |
| Pathotype | healthy_control_vs_amnestic_AD | nasales_iqr | 0,000 | 0,48 | medium |
| Pathotype | lvPPA_FTLD_vs_amnestic_AD | intensity_slope | 0,000 | 0,95 | large |
| Pathotype | lvPPA_FTLD_vs_amnestic_AD | semi_voyelles_count | 0,000 | 0,95 | large |
| Pathotype | lvPPA_FTLD_vs_amnestic_AD | semi_voyelles_median | 0,000 | 0,95 | large |
| Pathotype | lvPPA_FTLD_vs_amnestic_AD | semi_voyelles_max | 0,000 | 0,95 | large |
| Pathotype | svPPA_FTLD_vs_amnestic_AD | chroma_mean_1 | 0,000 | 0,82 | large |
| Pathotype | lvPPA_FTLD_vs_healthy_control | liquides_skewness | 0,000 | 0,91 | large |
| Pathotype | nfvPPA_FTLD_vs_amnestic_AD | semi_voyelles_iqr | 0,000 | 0,94 | large |
| Pathotype | lvPPA_FTLD_vs_amnestic_AD | semi_voyelles_mean | 0,000 | 0,95 | large |
| Pathotype | lvPPA_FTLD_vs_healthy_control | spectral_flux | 0,000 | 0,91 | large |
| Pathotype | lvPPA_FTLD_vs_amnestic_AD | contrast_mean_4 | 0,000 | 0,95 | large |
| Pathotype | lvPPA_FTLD_vs_amnestic_AD | occlusives_std | 0,000 | 0,95 | large |
| Pathotype | lvPPA_FTLD_vs_amnestic_AD | liquides_max | 0,000 | 0,94 | large |
| Pathotype | healthy_control_vs_lvPPA_AD | syllable_count | 0,000 | 0,67 | large |
| Pathotype | amnestic_AD_vs_lvPPA_AD | f0_max | 0,000 | 0,72 | large |
| Pathotype | nfvPPA_FTLD_vs_healthy_control | nasales_count | 0,000 | 0,91 | large |
| Pathotype | amnestic_AD_vs_lvPPA_AD | chroma_mean_3 | 0,000 | 0,72 | large |
| Pathotype | lvPPA_FTLD_vs_amnestic_AD | occlusives_count | 0,000 | 0,94 | large |
| Pathotype | lvPPA_FTLD_vs_healthy_control | spectral_flux_std | 0,000 | 0,91 | large |
| Pathotype | lvPPA_FTLD_vs_amnestic_AD | liquides_count | 0,000 | 0,94 | large |
| Pathotype | lvPPA_FTLD_vs_amnestic_AD | fricatives_count | 0,000 | 0,94 | large |
| Pathotype | lvPPA_FTLD_vs_amnestic_AD | occlusives_max | 0,000 | 0,94 | large |
| Pathotype | lvPPA_FTLD_vs_healthy_control | long_pause_count | 0,000 | 0,90 | large |
| Pathotype | lvPPA_FTLD_vs_amnestic_AD | mean_phoneme_duration | 0,000 | 0,94 | large |
| Pathotype | lvPPA_FTLD_vs_amnestic_AD | speech_rate_phonemes_per_sec | 0,000 | 0,94 | large |
| Pathotype | lvPPA_FTLD_vs_amnestic_AD | voyelles_oral_skewness | 0,000 | 0,94 | large |
| Pathotype | FTLD_vs_lvPPA_FTLD | long_pause_count | 0,000 | 1,00 | large |
| Pathotype | FTLD_vs_amnestic_AD | cv_phoneme_duration | 0,000 | 0,63 | large |
| Pathotype | svPPA_FTLD_vs_lvPPA_AD | shimmer_local | 0,000 | 0,96 | large |
| Pathotype | FTLD_vs_amnestic_AD | nasales_percentile_10 | 0,000 | 0,62 | large |
| Pathotype | lvPPA_FTLD_vs_amnestic_AD | occlusives_percentile_90 | 0,000 | 0,94 | large |
| Pathotype | lvPPA_FTLD_vs_amnestic_AD | max_phoneme_duration | 0,000 | 0,94 | large |
| Pathotype | nfvPPA_FTLD_vs_amnestic_AD | shimmer_apq5 | 0,000 | 0,94 | large |
| Pathotype | nfvPPA_FTLD_vs_amnestic_AD | spectral_flux | 0,000 | 0,94 | large |
| Pathotype | nfvPPA_FTLD_vs_amnestic_AD | delta_mfcc_mean_3 | 0,000 | 0,94 | large |
| Pathotype | nfvPPA_FTLD_vs_amnestic_AD | spectral_flux,1 | 0,000 | 0,94 | large |
| Pathotype | lvPPA_FTLD_vs_amnestic_AD | rate_speech_phonemes | 0,000 | 0,94 | large |
| Pathotype | nfvPPA_FTLD_vs_healthy_control | semi_voyelles_min | 0,000 | 0,89 | large |
| Pathotype | lvPPA_FTLD_vs_healthy_control | nasales_percentile_90 | 0,000 | 0,90 | large |
| Pathotype | nfvPPA_FTLD_vs_healthy_control | semi_voyelles_mean | 0,000 | 0,90 | large |
| Pathotype | nfvPPA_FTLD_vs_healthy_control | speech_duration | 0,000 | 0,90 | large |
| Pathotype | healthy_control_vs_amnestic_AD | nasales_percentile_90 | 0,000 | 0,47 | medium |
| Pathotype | svPPA_FTLD_vs_amnestic_AD | liquides_std | 0,000 | 0,81 | large |
| Pathotype | FTLD_vs_lvPPA_FTLD | liquides_max | 0,000 | 1,00 | large |
| Pathotype | FTLD_vs_lvPPA_FTLD | occlusives_max | 0,000 | 1,00 | large |
| Pathotype | FTLD_vs_lvPPA_FTLD | num_pauses | 0,000 | 1,00 | large |
| Pathotype | FTLD_vs_lvPPA_FTLD | num_inter_word_pauses | 0,000 | 1,00 | large |
| Pathotype | FTLD_vs_lvPPA_FTLD | num_inter_pauses | 0,000 | 1,00 | large |
| Pathotype | FTLD_vs_lvPPA_FTLD | occlusives_count | 0,000 | 1,00 | large |
| Pathotype | FTLD_vs_lvPPA_FTLD | occlusives_percentile_90 | 0,000 | 1,00 | large |
| Pathotype | nfvPPA_FTLD_vs_healthy_control | mean_phoneme_duration | 0,000 | 0,90 | large |
| Pathotype | nfvPPA_FTLD_vs_healthy_control | speech_rate_phonemes_per_sec | 0,000 | 0,90 | large |
| Pathotype | nfvPPA_FTLD_vs_healthy_control | rate_speech_phonemes | 0,000 | 0,90 | large |
| Pathotype | FTLD_vs_lvPPA_FTLD | liquides_std | 0,000 | 1,00 | large |
| Pathotype | FTLD_vs_lvPPA_FTLD | liquides_cv | 0,000 | 1,00 | large |
| Pathotype | svPPA_FTLD_vs_amnestic_AD | intensity_dynamic_range | 0,000 | 0,81 | large |
| Pathotype | FTLD_vs_lvPPA_FTLD | nasales_std | 0,000 | 1,00 | large |
| Pathotype | FTLD_vs_lvPPA_FTLD | nasales_cv | 0,000 | 1,00 | large |
| Pathotype | FTLD_vs_lvPPA_FTLD | nasales_iqr | 0,000 | 1,00 | large |
| Pathotype | FTLD_vs_lvPPA_FTLD | occlusives_kurtosis | 0,000 | 1,00 | large |
| Pathotype | healthy_control_vs_lvPPA_AD | voyelles_oral_min | 0,000 | 0,61 | large |
| Pathotype | FTLD_vs_lvPPA_FTLD | delta_mfcc_mean_6 | 0,000 | 1,00 | large |
| Pathotype | amnestic_AD_vs_lvPPA_AD | semi_voyelles_median | 0,000 | 0,71 | large |
| Pathotype | FTLD_vs_lvPPA_FTLD | nasales_count | 0,000 | 0,99 | large |
| Pathotype | FTLD_vs_lvPPA_FTLD | contrast_mean_3 | 0,000 | 1,00 | large |
| Pathotype | FTLD_vs_lvPPA_FTLD | rmse_mean | 0,000 | 1,00 | large |
| Pathotype | FTLD_vs_lvPPA_FTLD | intensity_mean | 0,000 | 1,00 | large |
| Pathotype | nfvPPA_FTLD_vs_amnestic_AD | occlusives_iqr | 0,000 | 0,94 | large |
| Pathotype | nfvPPA_FTLD_vs_FTLD | hnr_mean | 0,000 | 1,00 | large |
| Pathotype | nfvPPA_FTLD_vs_FTLD | hnr_mean,1 | 0,000 | 1,00 | large |
| Pathotype | FTLD_vs_lvPPA_FTLD | occlusives_iqr | 0,000 | 1,00 | large |
| Pathotype | FTLD_vs_lvPPA_FTLD | liquides_percentile_90 | 0,000 | 1,00 | large |
| Pathotype | FTLD_vs_lvPPA_FTLD | nasales_max | 0,000 | 1,00 | large |
| Pathotype | nfvPPA_FTLD_vs_amnestic_AD | shimmer_local | 0,000 | 0,94 | large |
| Pathotype | lvPPA_FTLD_vs_amnestic_AD | intensity_std | 0,000 | 0,94 | large |
| Pathotype | lvPPA_FTLD_vs_amnestic_AD | cv_phoneme_duration | 0,000 | 0,94 | large |
| Pathotype | FTLD_vs_lvPPA_FTLD | liquides_mean | 0,000 | 1,00 | large |
| Pathotype | FTLD_vs_lvPPA_FTLD | nasales_percentile_90 | 0,000 | 1,00 | large |
| Pathotype | FTLD_vs_lvPPA_FTLD | occlusives_std | 0,000 | 1,00 | large |
| Pathotype | lvPPA_FTLD_vs_healthy_control | median_phoneme_duration | 0,000 | 0,87 | large |
| Pathotype | nfvPPA_FTLD_vs_healthy_control | nasales_iqr | 0,000 | 0,90 | large |
| Pathotype | lvPPA_FTLD_vs_healthy_control | mean_phoneme_duration | 0,000 | 0,90 | large |
| Pathotype | lvPPA_FTLD_vs_healthy_control | speech_rate_phonemes_per_sec | 0,000 | 0,90 | large |
| Pathotype | lvPPA_FTLD_vs_healthy_control | rate_speech_phonemes | 0,000 | 0,90 | large |
| Pathotype | lvPPA_FTLD_vs_healthy_control | intensity_std | 0,000 | 0,90 | large |
| Pathotype | nfvPPA_FTLD_vs_healthy_control | contrast_mean_4 | 0,000 | 0,90 | large |
| Pathotype | svPPA_FTLD_vs_healthy_control | contrast_mean_0 | 0,000 | 0,76 | large |
| Pathotype | FTLD_vs_lvPPA_AD | f0_max | 0,000 | 0,78 | large |
| Pathotype | FTLD_vs_lvPPA_FTLD | liquides_count | 0,000 | 0,99 | large |
| Pathotype | svPPA_FTLD_vs_amnestic_AD | intensity_std | 0,000 | 0,81 | large |
| Pathotype | nfvPPA_FTLD_vs_amnestic_AD | f3 | 0,000 | 0,93 | large |
| Pathotype | nfvPPA_FTLD_vs_FTLD | liquides_iqr | 0,000 | 0,99 | large |
| Pathotype | nfvPPA_FTLD_vs_FTLD | occlusives_mean | 0,000 | 0,99 | large |
| Pathotype | amnestic_AD_vs_lvPPA_AD | chroma_mean_1 | 0,000 | 0,71 | large |
| Pathotype | healthy_control_vs_amnestic_AD | inter_word_pause_mean | 0,000 | 0,46 | medium |
| Pathotype | svPPA_FTLD_vs_FTLD | occlusives_percentile_10 | 0,000 | 0,86 | large |
| Pathotype | nfvPPA_FTLD_vs_FTLD | occlusives_percentile_90 | 0,000 | 0,99 | large |
| Pathotype | FTLD_vs_lvPPA_FTLD | syllable_count | 0,000 | 0,99 | large |
| Pathotype | FTLD_vs_lvPPA_FTLD | voyelles_oral_count | 0,000 | 0,99 | large |
| Pathotype | svPPA_FTLD_vs_amnestic_AD | spectral_flux | 0,000 | 0,80 | large |
| Pathotype | svPPA_FTLD_vs_amnestic_AD | spectral_flux,1 | 0,000 | 0,80 | large |
| Pathotype | healthy_control_vs_lvPPA_AD | f1_cv | 0,000 | 0,66 | large |
| Pathotype | FTLD_vs_lvPPA_FTLD | phoneme_count | 0,000 | 0,99 | large |
| Pathotype | FTLD_vs_lvPPA_FTLD | num_speech_phonemes | 0,000 | 0,99 | large |
| Pathotype | FTLD_vs_lvPPA_FTLD | speech_duration | 0,000 | 0,99 | large |
| Pathotype | lvPPA_FTLD_vs_amnestic_AD | spectral_centroid_std | 0,000 | 0,92 | large |
| Pathotype | lvPPA_FTLD_vs_amnestic_AD | std_phoneme_duration | 0,000 | 0,92 | large |
| Pathotype | svPPA_FTLD_vs_lvPPA_AD | pause_mean_duration | 0,000 | 0,93 | large |
| Pathotype | svPPA_FTLD_vs_lvPPA_AD | spectral_flux_mean | 0,000 | 0,93 | large |
| Pathotype | lvPPA_FTLD_vs_healthy_control | nasales_skewness | 0,000 | 0,88 | large |
| Pathotype | nfvPPA_FTLD_vs_healthy_control | semi_voyelles_percentile_90 | 0,000 | 0,88 | large |
| Pathotype | nfvPPA_FTLD_vs_healthy_control | semi_voyelles_std | 0,000 | 0,88 | large |
| Pathotype | lvPPA_FTLD_vs_healthy_control | intensity_slope | 0,000 | 0,88 | large |
| Pathotype | amnestic_AD_vs_lvPPA_AD | liquides_kurtosis | 0,000 | 0,69 | large |
| Pathotype | FTLD_vs_lvPPA_FTLD | median_phoneme_duration | 0,000 | 0,97 | large |
| Pathotype | lvPPA_FTLD_vs_amnestic_AD | fricatives_max | 0,000 | 0,91 | large |
| Pathotype | FTLD_vs_lvPPA_FTLD | fricatives_max | 0,000 | 0,97 | large |
| Pathotype | FTLD_vs_lvPPA_FTLD | chroma_mean_2 | 0,000 | 0,97 | large |
| Pathotype | FTLD_vs_lvPPA_FTLD | chroma_mean_3 | 0,000 | 0,97 | large |
| Pathotype | FTLD_vs_lvPPA_FTLD | chroma_mean_4 | 0,000 | 0,97 | large |
| Pathotype | FTLD_vs_lvPPA_FTLD | max_phoneme_duration | 0,000 | 0,97 | large |
| Pathotype | FTLD_vs_lvPPA_FTLD | mean_phoneme_duration | 0,000 | 0,97 | large |
| Pathotype | FTLD_vs_lvPPA_FTLD | speech_rate_phonemes_per_sec | 0,000 | 0,97 | large |
| Pathotype | FTLD_vs_lvPPA_FTLD | rate_speech_phonemes | 0,000 | 0,97 | large |
| Pathotype | nfvPPA_FTLD_vs_amnestic_AD | syllable_count | 0,000 | 0,90 | large |
| Pathotype | FTLD_vs_healthy_control | jitter_local | 0,000 | 0,55 | large |
| Pathotype | svPPA_FTLD_vs_healthy_control | jitter_rap | 0,000 | 0,74 | large |
| Pathotype | lvPPA_FTLD_vs_healthy_control | fricatives_skewness | 0,000 | 0,87 | large |
| Pathotype | healthy_control_vs_amnestic_AD | syllable_rate_per_sec | 0,000 | 0,45 | medium |
| Pathotype | lvPPA_FTLD_vs_healthy_control | skew_phoneme_duration | 0,000 | 0,87 | large |
| Pathotype | amnestic_AD_vs_lvPPA_AD | contrast_mean_1 | 0,000 | 0,69 | large |
| Pathotype | nfvPPA_FTLD_vs_healthy_control | rolloff_mean | 0,000 | 0,87 | large |
| Pathotype | healthy_control_vs_lvPPA_AD | liquides_mean | 0,000 | 0,64 | large |
| Pathotype | lvPPA_FTLD_vs_amnestic_AD | chroma_mean_1 | 0,000 | 0,90 | large |
| Pathotype | svPPA_FTLD_vs_amnestic_AD | spectral_centroid_mean | 0,000 | 0,78 | large |
| Pathotype | healthy_control_vs_amnestic_AD | nasales_mean | 0,000 | 0,45 | medium |
| Pathotype | lvPPA_FTLD_vs_amnestic_AD | voyelles_oral_max | 0,000 | 0,90 | large |
| Pathotype | lvPPA_FTLD_vs_amnestic_AD | chroma_mean_2 | 0,000 | 0,90 | large |
| Pathotype | lvPPA_FTLD_vs_amnestic_AD | chroma_mean_3 | 0,000 | 0,90 | large |
| Pathotype | lvPPA_FTLD_vs_amnestic_AD | contrast_mean_2 | 0,000 | 0,90 | large |
| Pathotype | lvPPA_FTLD_vs_amnestic_AD | fricatives_std | 0,000 | 0,90 | large |
| Pathotype | amnestic_AD_vs_lvPPA_AD | intensity_slope | 0,000 | 0,69 | large |
| Pathotype | FTLD_vs_lvPPA_FTLD | min_phoneme_duration | 0,000 | 0,91 | large |
| Pathotype | healthy_control_vs_amnestic_AD | f3 | 0,000 | 0,45 | medium |
| Pathotype | amnestic_AD_vs_lvPPA_AD | b3 | 0,000 | 0,69 | large |
| Pathotype | amnestic_AD_vs_lvPPA_AD | median_phoneme_duration | 0,000 | 0,67 | large |
| Pathotype | svPPA_FTLD_vs_lvPPA_AD | chroma_mean_0 | 0,000 | 0,91 | large |
| Pathotype | FTLD_vs_lvPPA_FTLD | fricatives_count | 0,000 | 0,96 | large |
| Pathotype | nfvPPA_FTLD_vs_healthy_control | pause_mean_duration | 0,000 | 0,86 | large |
| Pathotype | nfvPPA_FTLD_vs_healthy_control | inter_word_pause_mean | 0,000 | 0,86 | large |
| Pathotype | lvPPA_FTLD_vs_healthy_control | spectral_flux_mean | 0,000 | 0,86 | large |
| Pathotype | nfvPPA_FTLD_vs_FTLD | shimmer_apq5 | 0,000 | 0,96 | large |
| Pathotype | lvPPA_FTLD_vs_lvPPA_AD | semi_voyelles_std | 0,000 | 1,00 | large |
| Pathotype | lvPPA_FTLD_vs_lvPPA_AD | semi_voyelles_cv | 0,000 | 1,00 | large |
| Pathotype | lvPPA_FTLD_vs_lvPPA_AD | semi_voyelles_iqr | 0,000 | 1,00 | large |
| Pathotype | svPPA_FTLD_vs_amnestic_AD | liquides_max | 0,000 | 0,77 | large |
| Pathotype | FTLD_vs_lvPPA_FTLD | semi_voyelles_count | 0,000 | 0,92 | large |
| Pathotype | amnestic_AD_vs_lvPPA_AD | contrast_mean_5 | 0,000 | 0,68 | large |
| Pathotype | svPPA_FTLD_vs_healthy_control | chroma_mean_11 | 0,000 | 0,73 | large |
| Pathotype | svPPA_FTLD_vs_amnestic_AD | total_duration | 0,000 | 0,80 | large |
| Pathotype | healthy_control_vs_lvPPA_AD | f0_mean | 0,000 | 0,63 | large |
| Pathotype | lvPPA_FTLD_vs_lvPPA_AD | num_pauses | 0,000 | 1,00 | large |
| Pathotype | lvPPA_FTLD_vs_lvPPA_AD | num_inter_word_pauses | 0,000 | 1,00 | large |
| Pathotype | healthy_control_vs_lvPPA_AD | f1_cv,1 | 0,000 | 0,63 | large |
| Pathotype | lvPPA_FTLD_vs_amnestic_AD | spectral_flux_mean | 0,000 | 0,89 | large |
| Pathotype | lvPPA_FTLD_vs_amnestic_AD | nasales_skewness | 0,000 | 0,89 | large |
| Pathotype | FTLD_vs_healthy_control | f3 | 0,000 | 0,54 | large |
| Pathotype | nfvPPA_FTLD_vs_healthy_control | pause_median_duration | 0,000 | 0,85 | large |
| Pathotype | nfvPPA_FTLD_vs_healthy_control | inter_word_pause_median | 0,000 | 0,85 | large |
| Pathotype | lvPPA_FTLD_vs_healthy_control | voyelles_oral_cv | 0,000 | 0,85 | large |
| Pathotype | svPPA_FTLD_vs_lvPPA_AD | fricatives_percentile_10 | 0,000 | 0,90 | large |
| Pathotype | lvPPA_FTLD_vs_healthy_control | intensity_dynamic_range | 0,000 | 0,85 | large |
| Pathotype | nfvPPA_FTLD_vs_healthy_control | pause_std_duration | 0,000 | 0,85 | large |
| Pathotype | nfvPPA_FTLD_vs_healthy_control | inter_word_pause_std | 0,000 | 0,85 | large |
| Pathotype | nfvPPA_FTLD_vs_amnestic_AD | speech_duration | 0,000 | 0,89 | large |
| Pathotype | FTLD_vs_lvPPA_FTLD | liquides_iqr | 0,000 | 0,95 | large |
| Pathotype | lvPPA_FTLD_vs_lvPPA_AD | voyelles_oral_kurtosis | 0,000 | 1,00 | large |
| Pathotype | lvPPA_FTLD_vs_lvPPA_AD | liquides_std | 0,000 | 1,00 | large |
| Pathotype | lvPPA_FTLD_vs_lvPPA_AD | liquides_iqr | 0,000 | 1,00 | large |
| Pathotype | lvPPA_FTLD_vs_lvPPA_AD | nasales_std | 0,000 | 1,00 | large |
| Pathotype | lvPPA_FTLD_vs_lvPPA_AD | nasales_iqr | 0,000 | 1,00 | large |
| Pathotype | healthy_control_vs_amnestic_AD | semi_voyelles_median | 0,000 | 0,44 | medium |
| Pathotype | FTLD_vs_lvPPA_FTLD | chroma_mean_1 | 0,000 | 0,95 | large |
| Pathotype | healthy_control_vs_amnestic_AD | shimmer_apq3 | 0,000 | 0,44 | medium |
| Pathotype | healthy_control_vs_amnestic_AD | shimmer_dda | 0,000 | 0,44 | medium |
| Pathotype | healthy_control_vs_lvPPA_AD | chroma_mean_3 | 0,000 | 0,63 | large |
| Pathotype | nfvPPA_FTLD_vs_amnestic_AD | nasales_count | 0,000 | 0,88 | large |
| Pathotype | FTLD_vs_lvPPA_FTLD | occlusives_mean | 0,000 | 0,95 | large |
| Pathotype | FTLD_vs_lvPPA_FTLD | total_duration | 0,000 | 0,95 | large |
| Pathotype | nfvPPA_FTLD_vs_lvPPA_AD | occlusives_percentile_90 | 0,000 | 1,00 | large |
| Pathotype | FTLD_vs_lvPPA_FTLD | liquides_skewness | 0,000 | 0,94 | large |
| Pathotype | FTLD_vs_lvPPA_FTLD | fricatives_skewness | 0,000 | 0,94 | large |
| Pathotype | lvPPA_FTLD_vs_lvPPA_AD | total_duration | 0,000 | 1,00 | large |
| Pathotype | lvPPA_FTLD_vs_lvPPA_AD | speech_duration | 0,000 | 1,00 | large |
| Pathotype | lvPPA_FTLD_vs_lvPPA_AD | syllable_count | 0,000 | 1,00 | large |
| Pathotype | lvPPA_FTLD_vs_lvPPA_AD | num_speech_phonemes | 0,000 | 1,00 | large |
| Pathotype | lvPPA_FTLD_vs_lvPPA_AD | mean_phoneme_duration | 0,000 | 1,00 | large |
| Pathotype | lvPPA_FTLD_vs_lvPPA_AD | speech_rate_phonemes_per_sec | 0,000 | 1,00 | large |
| Pathotype | lvPPA_FTLD_vs_lvPPA_AD | occlusives_std | 0,000 | 1,00 | large |
| Pathotype | lvPPA_FTLD_vs_lvPPA_AD | occlusives_iqr | 0,000 | 1,00 | large |
| Pathotype | lvPPA_FTLD_vs_lvPPA_AD | liquides_mean | 0,000 | 1,00 | large |
| Pathotype | lvPPA_FTLD_vs_amnestic_AD | hnr_mean | 0,000 | 0,88 | large |
| Pathotype | lvPPA_FTLD_vs_amnestic_AD | f2_cv,1 | 0,000 | 0,88 | large |
| Pathotype | lvPPA_FTLD_vs_lvPPA_AD | contrast_mean_3 | 0,000 | 1,00 | large |
| Pathotype | nfvPPA_FTLD_vs_lvPPA_AD | shimmer_local | 0,000 | 1,00 | large |
| Pathotype | nfvPPA_FTLD_vs_lvPPA_AD | shimmer_apq3 | 0,000 | 1,00 | large |
| Pathotype | nfvPPA_FTLD_vs_lvPPA_AD | shimmer_apq5 | 0,000 | 1,00 | large |
| Pathotype | nfvPPA_FTLD_vs_lvPPA_AD | shimmer_dda | 0,000 | 1,00 | large |
| Pathotype | nfvPPA_FTLD_vs_lvPPA_AD | hnr_mean | 0,000 | 1,00 | large |
| Pathotype | nfvPPA_FTLD_vs_lvPPA_AD | hnr_mean,1 | 0,000 | 1,00 | large |
| Pathotype | svPPA_FTLD_vs_healthy_control | occlusives_median | 0,000 | 0,71 | large |
| Pathotype | healthy_control_vs_amnestic_AD | occlusives_kurtosis | 0,000 | 0,44 | medium |
| Pathotype | healthy_control_vs_lvPPA_AD | semi_voyelles_count | 0,000 | 0,62 | large |
| Pathotype | lvPPA_FTLD_vs_amnestic_AD | min_phoneme_duration | 0,000 | 0,84 | large |
| Pathotype | FTLD_vs_amnestic_AD | spectral_flux,1 | 0,000 | 0,58 | large |
| Pathotype | nfvPPA_FTLD_vs_FTLD | occlusives_median | 0,000 | 0,93 | large |
| Pathotype | nfvPPA_FTLD_vs_FTLD | semi_voyelles_min | 0,000 | 0,82 | large |
| Pathotype | lvPPA_FTLD_vs_amnestic_AD | fricatives_skewness | 0,000 | 0,87 | large |
| Pathotype | lvPPA_FTLD_vs_amnestic_AD | chroma_mean_8 | 0,000 | 0,87 | large |
| Pathotype | svPPA_FTLD_vs_FTLD | spectral_instability | 0,000 | 0,82 | large |
| Pathotype | FTLD_vs_amnestic_AD | f1_cv,1 | 0,000 | 0,58 | large |
| Pathotype | svPPA_FTLD_vs_amnestic_AD | voyelles_oral_mean | 0,000 | 0,76 | large |
| Pathotype | svPPA_FTLD_vs_lvPPA_AD | chroma_mean_2 | 0,000 | 0,89 | large |
| Pathotype | svPPA_FTLD_vs_lvPPA_AD | contrast_mean_1 | 0,000 | 0,89 | large |
| Pathotype | svPPA_FTLD_vs_lvPPA_AD | f0_std | 0,000 | 0,89 | large |
| Pathotype | FTLD_vs_lvPPA_FTLD | voyelles_oral_max | 0,000 | 0,93 | large |
| Pathotype | nfvPPA_FTLD_vs_FTLD | shimmer_local | 0,000 | 0,93 | large |
| Pathotype | nfvPPA_FTLD_vs_FTLD | shimmer_apq3 | 0,000 | 0,93 | large |
| Pathotype | nfvPPA_FTLD_vs_FTLD | shimmer_dda | 0,000 | 0,93 | large |
| Pathotype | FTLD_vs_lvPPA_FTLD | kurt_phoneme_duration | 0,000 | 0,93 | large |
| Pathotype | nfvPPA_FTLD_vs_FTLD | f3 | 0,000 | 0,93 | large |
| Pathotype | FTLD_vs_lvPPA_FTLD | voyelles_oral_skewness | 0,000 | 0,93 | large |
| Pathotype | nfvPPA_FTLD_vs_healthy_control | semi_voyelles_max | 0,000 | 0,84 | large |
| Pathotype | svPPA_FTLD_vs_amnestic_AD | voyelles_oral_iqr | 0,000 | 0,75 | large |
| Pathotype | lvPPA_FTLD_vs_healthy_control | chroma_mean_3 | 0,000 | 0,84 | large |
| Pathotype | nfvPPA_FTLD_vs_amnestic_AD | phoneme_count | 0,000 | 0,87 | large |
| Pathotype | nfvPPA_FTLD_vs_amnestic_AD | num_speech_phonemes | 0,000 | 0,87 | large |
| Pathotype | svPPA_FTLD_vs_lvPPA_AD | nasales_count | 0,000 | 0,88 | large |
| Pathotype | svPPA_FTLD_vs_amnestic_AD | chroma_mean_11 | 0,000 | 0,75 | large |
| Pathotype | svPPA_FTLD_vs_amnestic_AD | skew_phoneme_duration | 0,000 | 0,75 | large |
| Pathotype | healthy_control_vs_lvPPA_AD | spectral_centroid_std | 0,000 | 0,61 | large |
| Pathotype | amnestic_AD_vs_lvPPA_AD | jitter_rap | 0,000 | 0,66 | large |
| Pathotype | amnestic_AD_vs_lvPPA_AD | rolloff_mean | 0,000 | 0,66 | large |
| Pathotype | nfvPPA_FTLD_vs_healthy_control | semi_voyelles_iqr | 0,000 | 0,83 | large |
| Pathotype | nfvPPA_FTLD_vs_healthy_control | liquides_percentile_90 | 0,000 | 0,83 | large |
| Pathotype | nfvPPA_FTLD_vs_healthy_control | chroma_mean_2 | 0,000 | 0,83 | large |
| Pathotype | FTLD_vs_healthy_control | f1_cv,1 | 0,000 | 0,52 | large |
| Pathotype | lvPPA_FTLD_vs_amnestic_AD | spectral_flux,1 | 0,000 | 0,87 | large |
| Pathotype | svPPA_FTLD_vs_FTLD | spectral_instability,1 | 0,000 | 0,81 | large |
| Pathotype | FTLD_vs_amnestic_AD | f1 | 0,000 | 0,57 | large |
| Pathotype | FTLD_vs_lvPPA_FTLD | semi_voyelles_max | 0,000 | 0,92 | large |
| Pathotype | nfvPPA_FTLD_vs_lvPPA_AD | chroma_mean_2 | 0,000 | 0,98 | large |
| Pathotype | nfvPPA_FTLD_vs_lvPPA_AD | chroma_mean_3 | 0,000 | 0,98 | large |
| Pathotype | FTLD_vs_lvPPA_FTLD | semi_voyelles_median | 0,000 | 0,92 | large |
| Pathotype | FTLD_vs_lvPPA_FTLD | semi_voyelles_mean | 0,000 | 0,92 | large |
| Pathotype | nfvPPA_FTLD_vs_amnestic_AD | occlusives_count | 0,000 | 0,86 | large |
| Pathotype | svPPA_FTLD_vs_lvPPA_AD | spectral_instability | 0,000 | 0,87 | large |
| Pathotype | svPPA_FTLD_vs_lvPPA_AD | spectral_instability,1 | 0,000 | 0,87 | large |
| Pathotype | FTLD_vs_lvPPA_FTLD | semi_voyelles_percentile_90 | 0,000 | 0,92 | large |
| Pathotype | svPPA_FTLD_vs_healthy_control | voyelles_oral_mean | 0,000 | 0,70 | large |
| Pathotype | lvPPA_FTLD_vs_amnestic_AD | delta_mfcc_mean_12 | 0,000 | 0,86 | large |
| Pathotype | healthy_control_vs_amnestic_AD | occlusives_cv | 0,000 | 0,43 | medium |
| Pathotype | FTLD_vs_amnestic_AD | rolloff_std | 0,000 | 0,57 | large |
| Pathotype | nfvPPA_FTLD_vs_healthy_control | std_phoneme_duration | 0,000 | 0,82 | large |
| Pathotype | healthy_control_vs_amnestic_AD | shimmer_apq5 | 0,000 | 0,43 | medium |
| Pathotype | FTLD_vs_healthy_control | f1_cv | 0,000 | 0,52 | large |
| Pathotype | svPPA_FTLD_vs_lvPPA_AD | liquides_count | 0,000 | 0,86 | large |
| Pathotype | amnestic_AD_vs_lvPPA_AD | spectral_centroid_mean | 0,000 | 0,65 | large |
| Pathotype | lvPPA_FTLD_vs_lvPPA_AD | num_inter_pauses | 0,000 | 0,96 | large |
| Pathotype | svPPA_FTLD_vs_amnestic_AD | b1 | 0,000 | 0,74 | large |
| Pathotype | FTLD_vs_lvPPA_AD | median_phoneme_duration | 0,000 | 0,71 | large |
| Pathotype | lvPPA_FTLD_vs_healthy_control | delta_mfcc_mean_9 | 0,000 | 0,82 | large |
| Pathotype | nfvPPA_FTLD_vs_amnestic_AD | voyelles_oral_count | 0,000 | 0,85 | large |
| Pathotype | FTLD_vs_healthy_control | articulation_rate | 0,000 | 0,50 | large |
| Pathotype | nfvPPA_FTLD_vs_amnestic_AD | liquides_iqr | 0,000 | 0,85 | large |
| Pathotype | FTLD_vs_lvPPA_AD | hnr_mean | 0,000 | 0,71 | large |
| Pathotype | FTLD_vs_amnestic_AD | contrast_mean_5 | 0,000 | 0,56 | large |
| Pathotype | FTLD_vs_lvPPA_AD | hnr_mean,1 | 0,000 | 0,71 | large |
| Pathotype | nfvPPA_FTLD_vs_lvPPA_AD | semi_voyelles_median | 0,000 | 0,96 | large |
| Pathotype | lvPPA_FTLD_vs_lvPPA_AD | nasales_cv | 0,000 | 0,96 | large |
| Pathotype | nfvPPA_FTLD_vs_amnestic_AD | liquides_median | 0,000 | 0,85 | large |
| Pathotype | nfvPPA_FTLD_vs_healthy_control | fricatives_percentile_90 | 0,000 | 0,81 | large |
| Pathotype | healthy_control_vs_lvPPA_AD | liquides_skewness | 0,000 | 0,60 | large |
| Pathotype | lvPPA_FTLD_vs_amnestic_AD | spectral_flux_std | 0,000 | 0,85 | large |
| Pathotype | FTLD_vs_lvPPA_FTLD | voyelles_oral_kurtosis | 0,000 | 0,90 | large |
| Pathotype | nfvPPA_FTLD_vs_healthy_control | liquides_count | 0,000 | 0,81 | large |
| Pathotype | healthy_control_vs_lvPPA_AD | f2_cv | 0,000 | 0,60 | large |
| Pathotype | lvPPA_FTLD_vs_healthy_control | fricatives_std | 0,000 | 0,81 | large |
| Pathotype | FTLD_vs_lvPPA_FTLD | f2_cv | 0,000 | 0,90 | large |
| Pathotype | nfvPPA_FTLD_vs_lvPPA_AD | rate_speech_phonemes | 0,000 | 0,96 | large |
| Pathotype | nfvPPA_FTLD_vs_lvPPA_AD | semi_voyelles_iqr | 0,000 | 0,96 | large |
| Pathotype | FTLD_vs_lvPPA_FTLD | occlusives_min | 0,000 | 0,86 | large |
| Pathotype | lvPPA_FTLD_vs_lvPPA_AD | rate_speech_phonemes | 0,000 | 0,96 | large |
| Pathotype | nfvPPA_FTLD_vs_healthy_control | syllable_rate_per_sec | 0,000 | 0,81 | large |
| Pathotype | lvPPA_FTLD_vs_lvPPA_AD | b3 | 0,000 | 0,96 | large |
| Pathotype | svPPA_FTLD_vs_lvPPA_FTLD | nasales_skewness | 0,000 | 1,00 | large |
| Pathotype | nfvPPA_FTLD_vs_lvPPA_AD | contrast_mean_1 | 0,000 | 0,96 | large |
| Pathotype | FTLD_vs_lvPPA_AD | rmse_mean | 0,000 | 0,71 | large |
| Pathotype | FTLD_vs_lvPPA_AD | intensity_mean | 0,000 | 0,71 | large |
| Pathotype | FTLD_vs_healthy_control | median_phoneme_duration | 0,000 | 0,50 | medium |
| Pathotype | svPPA_FTLD_vs_amnestic_AD | intensity_mean | 0,000 | 0,73 | large |
| Pathotype | lvPPA_FTLD_vs_amnestic_AD | occlusives_mean | 0,000 | 0,84 | large |
| Pathotype | nfvPPA_FTLD_vs_FTLD | speech_duration | 0,001 | 0,90 | large |
| Pathotype | nfvPPA_FTLD_vs_healthy_control | nasales_percentile_90 | 0,001 | 0,81 | large |
| Pathotype | healthy_control_vs_lvPPA_AD | f2_cv,1 | 0,001 | 0,60 | large |
| Pathotype | lvPPA_FTLD_vs_healthy_control | std_phoneme_duration | 0,001 | 0,81 | large |
| Pathotype | amnestic_AD_vs_lvPPA_AD | jitter_local | 0,001 | 0,64 | large |
| Pathotype | svPPA_FTLD_vs_healthy_control | nasales_median | 0,001 | 0,68 | large |
| Pathotype | FTLD_vs_lvPPA_FTLD | voyelles_oral_percentile_10 | 0,001 | 0,82 | large |
| Pathotype | svPPA_FTLD_vs_healthy_control | intensity_mean | 0,001 | 0,68 | large |
| Pathotype | lvPPA_FTLD_vs_amnestic_AD | nasales_kurtosis | 0,001 | 0,83 | large |
| Pathotype | nfvPPA_FTLD_vs_amnestic_AD | nasales_kurtosis | 0,001 | 0,83 | large |
| Pathotype | lvPPA_FTLD_vs_healthy_control | pause_ratio | 0,001 | 0,80 | large |
| Pathotype | amnestic_AD_vs_lvPPA_AD | pause_total_duration | 0,001 | 0,63 | large |
| Pathotype | FTLD_vs_lvPPA_FTLD | skew_phoneme_duration | 0,001 | 0,89 | large |
| Pathotype | svPPA_FTLD_vs_FTLD | occlusives_skewness | 0,001 | 0,78 | large |
| Pathotype | svPPA_FTLD_vs_lvPPA_AD | inter_word_pause_mean | 0,001 | 0,84 | large |
| Pathotype | lvPPA_FTLD_vs_lvPPA_AD | fricatives_skewness | 0,001 | 0,94 | large |
| Pathotype | svPPA_FTLD_vs_healthy_control | spectral_instability,1 | 0,001 | 0,68 | large |
| Pathotype | svPPA_FTLD_vs_healthy_control | chroma_mean_2 | 0,001 | 0,68 | large |
| Pathotype | svPPA_FTLD_vs_lvPPA_FTLD | long_pause_count | 0,001 | 1,00 | large |
| Pathotype | svPPA_FTLD_vs_lvPPA_FTLD | num_pauses | 0,001 | 1,00 | large |
| Pathotype | svPPA_FTLD_vs_lvPPA_FTLD | num_inter_word_pauses | 0,001 | 1,00 | large |
| Pathotype | svPPA_FTLD_vs_lvPPA_FTLD | num_inter_pauses | 0,001 | 1,00 | large |
| Pathotype | svPPA_FTLD_vs_lvPPA_FTLD | occlusives_count | 0,001 | 1,00 | large |
| Pathotype | svPPA_FTLD_vs_lvPPA_FTLD | liquides_count | 0,001 | 1,00 | large |
| Pathotype | healthy_control_vs_amnestic_AD | occlusives_count | 0,001 | 0,41 | medium |
| Pathotype | nfvPPA_FTLD_vs_healthy_control | semi_voyelles_percentile_10 | 0,001 | 0,79 | large |
| Pathotype | nfvPPA_FTLD_vs_lvPPA_AD | occlusives_median | 0,001 | 0,94 | large |
| Pathotype | healthy_control_vs_lvPPA_AD | spectral_flux | 0,001 | 0,59 | large |
| Pathotype | lvPPA_FTLD_vs_lvPPA_AD | std_phoneme_duration | 0,001 | 0,94 | large |
| Pathotype | lvPPA_FTLD_vs_amnestic_AD | chroma_mean_0 | 0,001 | 0,83 | large |
| Pathotype | FTLD_vs_healthy_control | nasales_min | 0,001 | 0,49 | medium |
| Pathotype | lvPPA_FTLD_vs_amnestic_AD | delta_mfcc_mean_8 | 0,001 | 0,83 | large |
| Pathotype | lvPPA_FTLD_vs_amnestic_AD | rolloff_std | 0,001 | 0,83 | large |
| Pathotype | svPPA_FTLD_vs_lvPPA_FTLD | liquides_std | 0,001 | 1,00 | large |
| Pathotype | svPPA_FTLD_vs_lvPPA_FTLD | liquides_cv | 0,001 | 1,00 | large |
| Pathotype | svPPA_FTLD_vs_lvPPA_FTLD | liquides_iqr | 0,001 | 1,00 | large |
| Pathotype | healthy_control_vs_lvPPA_AD | rolloff_std | 0,001 | 0,58 | large |
| Pathotype | svPPA_FTLD_vs_lvPPA_FTLD | voyelles_oral_kurtosis | 0,001 | 1,00 | large |
| Pathotype | svPPA_FTLD_vs_lvPPA_FTLD | nasales_std | 0,001 | 1,00 | large |
| Pathotype | svPPA_FTLD_vs_lvPPA_FTLD | nasales_cv | 0,001 | 1,00 | large |
| Pathotype | svPPA_FTLD_vs_lvPPA_FTLD | nasales_iqr | 0,001 | 1,00 | large |
| Pathotype | amnestic_AD_vs_lvPPA_AD | semi_voyelles_percentile_90 | 0,001 | 0,63 | large |
| Pathotype | amnestic_AD_vs_lvPPA_AD | pause_ratio | 0,001 | 0,63 | large |
| Pathotype | amnestic_AD_vs_lvPPA_AD | f0_mean | 0,001 | 0,63 | large |
| Pathotype | svPPA_FTLD_vs_FTLD | nasales_percentile_10 | 0,001 | 0,76 | large |
| Pathotype | svPPA_FTLD_vs_healthy_control | jitter_local | 0,001 | 0,67 | large |
| Pathotype | svPPA_FTLD_vs_healthy_control | zcr_mean | 0,001 | 0,67 | large |
| Pathotype | svPPA_FTLD_vs_healthy_control | rmse_mean | 0,001 | 0,67 | large |
| Pathotype | svPPA_FTLD_vs_lvPPA_FTLD | f2_cv | 0,001 | 1,00 | large |
| Pathotype | svPPA_FTLD_vs_lvPPA_FTLD | chroma_mean_4 | 0,001 | 1,00 | large |
| Pathotype | svPPA_FTLD_vs_lvPPA_FTLD | spectral_centroid_mean | 0,001 | 1,00 | large |
| Pathotype | svPPA_FTLD_vs_lvPPA_FTLD | rolloff_mean | 0,001 | 1,00 | large |
| Pathotype | FTLD_vs_lvPPA_FTLD | semi_voyelles_iqr | 0,001 | 0,86 | large |
| Pathotype | svPPA_FTLD_vs_nfvPPA_FTLD | shimmer_local | 0,001 | 1,00 | large |
| Pathotype | svPPA_FTLD_vs_nfvPPA_FTLD | shimmer_apq3 | 0,001 | 1,00 | large |
| Pathotype | svPPA_FTLD_vs_nfvPPA_FTLD | shimmer_apq5 | 0,001 | 1,00 | large |
| Pathotype | svPPA_FTLD_vs_nfvPPA_FTLD | shimmer_dda | 0,001 | 1,00 | large |
| Pathotype | svPPA_FTLD_vs_nfvPPA_FTLD | rolloff_mean | 0,001 | 1,00 | large |
| Pathotype | svPPA_FTLD_vs_lvPPA_FTLD | occlusives_percentile_90 | 0,001 | 1,00 | large |
| Pathotype | svPPA_FTLD_vs_lvPPA_FTLD | median_phoneme_duration | 0,001 | 1,00 | large |
| Pathotype | FTLD_vs_healthy_control | chroma_mean_5 | 0,001 | 0,49 | medium |
| Pathotype | FTLD_vs_lvPPA_FTLD | semi_voyelles_std | 0,001 | 0,86 | large |
| Pathotype | FTLD_vs_lvPPA_FTLD | semi_voyelles_cv | 0,001 | 0,86 | large |
| Pathotype | svPPA_FTLD_vs_lvPPA_FTLD | phoneme_count | 0,001 | 1,00 | large |
| Pathotype | svPPA_FTLD_vs_lvPPA_FTLD | syllable_count | 0,001 | 1,00 | large |
| Pathotype | svPPA_FTLD_vs_lvPPA_FTLD | num_speech_phonemes | 0,001 | 1,00 | large |
| Pathotype | svPPA_FTLD_vs_lvPPA_FTLD | voyelles_oral_count | 0,001 | 1,00 | large |
| Pathotype | svPPA_FTLD_vs_lvPPA_FTLD | nasales_max | 0,001 | 1,00 | large |
| Pathotype | healthy_control_vs_amnestic_AD | num_intra_word_pauses | 0,001 | 0,17 | small |
| Pathotype | healthy_control_vs_amnestic_AD | voyelles_nasales_count | 0,001 | 0,17 | small |
| Pathotype | FTLD_vs_lvPPA_AD | f0_mean | 0,001 | 0,69 | large |
| Pathotype | amnestic_AD_vs_lvPPA_AD | chroma_mean_7 | 0,001 | 0,62 | large |
| Pathotype | lvPPA_FTLD_vs_healthy_control | liquides_min | 0,001 | 0,76 | large |
| Pathotype | svPPA_FTLD_vs_lvPPA_AD | pause_median_duration | 0,001 | 0,83 | large |
| Pathotype | svPPA_FTLD_vs_lvPPA_AD | inter_word_pause_median | 0,001 | 0,83 | large |
| Pathotype | svPPA_FTLD_vs_lvPPA_AD | nasales_skewness | 0,001 | 0,83 | large |
| Pathotype | svPPA_FTLD_vs_lvPPA_FTLD | delta_mfcc_mean_11 | 0,001 | 1,00 | large |
| Pathotype | svPPA_FTLD_vs_lvPPA_FTLD | chroma_mean_1 | 0,001 | 1,00 | large |
| Pathotype | svPPA_FTLD_vs_lvPPA_FTLD | chroma_mean_2 | 0,001 | 1,00 | large |
| Pathotype | svPPA_FTLD_vs_lvPPA_FTLD | chroma_mean_3 | 0,001 | 1,00 | large |
| Pathotype | svPPA_FTLD_vs_lvPPA_FTLD | contrast_mean_3 | 0,001 | 1,00 | large |
| Pathotype | svPPA_FTLD_vs_lvPPA_FTLD | rmse_mean | 0,001 | 1,00 | large |
| Pathotype | svPPA_FTLD_vs_lvPPA_FTLD | intensity_mean | 0,001 | 1,00 | large |
| Pathotype | svPPA_FTLD_vs_lvPPA_FTLD | occlusives_max | 0,001 | 1,00 | large |
| Pathotype | nfvPPA_FTLD_vs_healthy_control | fricatives_iqr | 0,001 | 0,78 | large |
| Pathotype | svPPA_FTLD_vs_nfvPPA_FTLD | hnr_mean | 0,001 | 1,00 | large |
| Pathotype | svPPA_FTLD_vs_nfvPPA_FTLD | hnr_mean,1 | 0,001 | 1,00 | large |
| Pathotype | svPPA_FTLD_vs_lvPPA_FTLD | liquides_max | 0,001 | 1,00 | large |
| Pathotype | svPPA_FTLD_vs_lvPPA_FTLD | liquides_percentile_90 | 0,001 | 1,00 | large |
| Pathotype | nfvPPA_FTLD_vs_healthy_control | pause_ratio | 0,001 | 0,78 | large |
| Pathotype | lvPPA_FTLD_vs_healthy_control | f2_cv,1 | 0,001 | 0,78 | large |
| Pathotype | nfvPPA_FTLD_vs_healthy_control | voyelles_oral_min | 0,001 | 0,71 | large |
| Pathotype | amnestic_AD_vs_lvPPA_AD | fricatives_median | 0,001 | 0,62 | large |
| Pathotype | healthy_control_vs_amnestic_AD | skew_phoneme_duration | 0,001 | 0,41 | medium |
| Pathotype | nfvPPA_FTLD_vs_FTLD | phoneme_count | 0,001 | 0,87 | large |
| Pathotype | nfvPPA_FTLD_vs_FTLD | num_speech_phonemes | 0,001 | 0,87 | large |
| Pathotype | svPPA_FTLD_vs_amnestic_AD | occlusives_skewness | 0,001 | 0,71 | large |
| Pathotype | svPPA_FTLD_vs_amnestic_AD | occlusives_kurtosis | 0,001 | 0,71 | large |
| Pathotype | svPPA_FTLD_vs_amnestic_AD | rmse_mean | 0,001 | 0,71 | large |
| Pathotype | FTLD_vs_amnestic_AD | fricatives_count | 0,001 | 0,54 | large |
| Pathotype | svPPA_FTLD_vs_lvPPA_FTLD | occlusives_std | 0,001 | 1,00 | large |
| Pathotype | svPPA_FTLD_vs_lvPPA_FTLD | occlusives_iqr | 0,001 | 1,00 | large |
| Pathotype | svPPA_FTLD_vs_lvPPA_FTLD | fricatives_count | 0,001 | 1,00 | large |
| Pathotype | svPPA_FTLD_vs_lvPPA_FTLD | liquides_mean | 0,001 | 1,00 | large |
| Pathotype | svPPA_FTLD_vs_lvPPA_FTLD | nasales_count | 0,001 | 1,00 | large |
| Pathotype | healthy_control_vs_lvPPA_AD | spectral_flux,1 | 0,001 | 0,58 | large |
| Pathotype | svPPA_FTLD_vs_lvPPA_FTLD | speech_duration | 0,001 | 1,00 | large |
| Pathotype | svPPA_FTLD_vs_lvPPA_FTLD | voyelles_oral_skewness | 0,001 | 1,00 | large |
| Pathotype | svPPA_FTLD_vs_lvPPA_FTLD | nasales_percentile_90 | 0,001 | 1,00 | large |
| Pathotype | nfvPPA_FTLD_vs_FTLD | fricatives_kurtosis | 0,001 | 0,87 | large |
| Pathotype | svPPA_FTLD_vs_healthy_control | spectral_instability | 0,001 | 0,67 | large |
| Pathotype | healthy_control_vs_amnestic_AD | liquides_max | 0,001 | 0,40 | medium |
| Pathotype | svPPA_FTLD_vs_amnestic_AD | liquides_cv | 0,001 | 0,70 | large |
| Pathotype | healthy_control_vs_amnestic_AD | hnr_std,1 | 0,001 | 0,40 | medium |
| Pathotype | svPPA_FTLD_vs_FTLD | delta_mfcc_mean_6 | 0,001 | 0,76 | large |
| Pathotype | healthy_control_vs_lvPPA_AD | pause_max_duration | 0,001 | 0,58 | large |
| Pathotype | healthy_control_vs_lvPPA_AD | inter_word_pause_max | 0,001 | 0,58 | large |
| Pathotype | svPPA_FTLD_vs_healthy_control | nasales_max | 0,001 | 0,66 | large |
| Pathotype | svPPA_FTLD_vs_healthy_control | fricatives_count | 0,001 | 0,66 | large |
| Pathotype | nfvPPA_FTLD_vs_healthy_control | semi_voyelles_median | 0,001 | 0,77 | large |
| Pathotype | svPPA_FTLD_vs_healthy_control | occlusives_skewness | 0,001 | 0,66 | large |
| Pathotype | svPPA_FTLD_vs_amnestic_AD | f3 | 0,001 | 0,70 | large |
| Pathotype | FTLD_vs_lvPPA_AD | pause_ratio | 0,001 | 0,68 | large |
| Pathotype | nfvPPA_FTLD_vs_amnestic_AD | delta_mfcc_mean_2 | 0,001 | 0,81 | large |
| Pathotype | nfvPPA_FTLD_vs_lvPPA_AD | median_phoneme_duration | 0,001 | 0,92 | large |
| Pathotype | FTLD_vs_lvPPA_AD | spectral_centroid_std | 0,001 | 0,68 | large |
| Pathotype | nfvPPA_FTLD_vs_lvPPA_AD | fricatives_kurtosis | 0,001 | 0,92 | large |
| Pathotype | svPPA_FTLD_vs_amnestic_AD | occlusives_iqr | 0,001 | 0,70 | large |
| Pathotype | healthy_control_vs_amnestic_AD | intensity_std | 0,001 | 0,40 | medium |
| Pathotype | FTLD_vs_lvPPA_AD | voyelles_oral_min | 0,001 | 0,64 | large |
| Pathotype | svPPA_FTLD_vs_amnestic_AD | spectral_centroid_slope | 0,001 | 0,70 | large |
| Pathotype | amnestic_AD_vs_lvPPA_AD | long_pause_count | 0,001 | 0,61 | large |
| Pathotype | lvPPA_FTLD_vs_lvPPA_AD | pause_total_duration | 0,001 | 0,92 | large |
| Pathotype | lvPPA_FTLD_vs_lvPPA_AD | phoneme_count | 0,001 | 0,92 | large |
| Pathotype | lvPPA_FTLD_vs_lvPPA_AD | voyelles_oral_count | 0,001 | 0,92 | large |
| Pathotype | nfvPPA_FTLD_vs_amnestic_AD | pause_ratio | 0,001 | 0,81 | large |
| Pathotype | lvPPA_FTLD_vs_amnestic_AD | hnr_std,1 | 0,001 | 0,81 | large |
| Pathotype | healthy_control_vs_lvPPA_AD | contrast_mean_3 | 0,001 | 0,57 | large |
| Pathotype | FTLD_vs_lvPPA_FTLD | delta_mfcc_mean_11 | 0,001 | 0,86 | large |
| Pathotype | amnestic_AD_vs_lvPPA_AD | b1 | 0,001 | 0,62 | large |
| Pathotype | nfvPPA_FTLD_vs_lvPPA_AD | f0_max | 0,001 | 0,92 | large |
| Pathotype | lvPPA_FTLD_vs_healthy_control | fricatives_cv | 0,001 | 0,77 | large |
| Pathotype | healthy_control_vs_amnestic_AD | liquides_min | 0,001 | 0,39 | medium |
| Pathotype | healthy_control_vs_amnestic_AD | rate_speech_phonemes | 0,001 | 0,40 | medium |
| Pathotype | lvPPA_FTLD_vs_lvPPA_AD | delta_mfcc_mean_9 | 0,001 | 0,92 | large |
| Pathotype | nfvPPA_FTLD_vs_lvPPA_AD | intensity_dynamic_range | 0,001 | 0,92 | large |
| Pathotype | nfvPPA_FTLD_vs_FTLD | fricatives_count | 0,001 | 0,86 | large |
| Pathotype | svPPA_FTLD_vs_healthy_control | occlusives_percentile_90 | 0,001 | 0,66 | large |
| Pathotype | FTLD_vs_healthy_control | pause_std_duration | 0,001 | 0,48 | medium |
| Pathotype | FTLD_vs_healthy_control | inter_word_pause_std | 0,001 | 0,48 | medium |
| Pathotype | lvPPA_FTLD_vs_amnestic_AD | fricatives_percentile_90 | 0,001 | 0,80 | large |
| Pathotype | amnestic_AD_vs_lvPPA_AD | speech_duration | 0,001 | 0,61 | large |
| Pathotype | lvPPA_FTLD_vs_amnestic_AD | spectral_instability,1 | 0,001 | 0,80 | large |
| Pathotype | nfvPPA_FTLD_vs_FTLD | occlusives_count | 0,001 | 0,86 | large |
| Pathotype | amnestic_AD_vs_lvPPA_AD | pause_min_duration | 0,001 | 0,61 | large |
| Pathotype | amnestic_AD_vs_lvPPA_AD | inter_word_pause_min | 0,001 | 0,61 | large |
| Pathotype | FTLD_vs_lvPPA_AD | speech_duration | 0,001 | 0,67 | large |
| Pathotype | svPPA_FTLD_vs_lvPPA_AD | chroma_mean_3 | 0,001 | 0,81 | large |
| Pathotype | nfvPPA_FTLD_vs_FTLD | mean_phoneme_duration | 0,001 | 0,86 | large |
| Pathotype | FTLD_vs_healthy_control | chroma_mean_6 | 0,001 | 0,48 | medium |
| Pathotype | svPPA_FTLD_vs_lvPPA_AD | voyelles_oral_min | 0,001 | 0,77 | large |
| Pathotype | svPPA_FTLD_vs_healthy_control | occlusives_percentile_10 | 0,001 | 0,63 | large |
| Pathotype | healthy_control_vs_lvPPA_AD | std_phoneme_duration | 0,001 | 0,57 | large |
| Pathotype | svPPA_FTLD_vs_healthy_control | f1 | 0,001 | 0,65 | large |
| Pathotype | healthy_control_vs_amnestic_AD | phoneme_count | 0,001 | 0,40 | medium |
| Pathotype | healthy_control_vs_amnestic_AD | num_speech_phonemes | 0,001 | 0,40 | medium |
| Pathotype | nfvPPA_FTLD_vs_healthy_control | pause_proportion | 0,001 | 0,77 | large |
| Pathotype | FTLD_vs_lvPPA_FTLD | occlusives_median | 0,001 | 0,85 | large |
| Pathotype | amnestic_AD_vs_lvPPA_AD | f1_cv,1 | 0,001 | 0,61 | large |
| Pathotype | healthy_control_vs_lvPPA_AD | pause_cv | 0,001 | 0,56 | large |
| Pathotype | svPPA_FTLD_vs_amnestic_AD | liquides_percentile_90 | 0,001 | 0,69 | large |
| Pathotype | svPPA_FTLD_vs_healthy_control | occlusives_kurtosis | 0,001 | 0,65 | large |
| Pathotype | FTLD_vs_lvPPA_FTLD | spectral_flux_std | 0,001 | 0,85 | large |
| Pathotype | nfvPPA_FTLD_vs_lvPPA_FTLD | semi_voyelles_std | 0,001 | 1,00 | large |
| Pathotype | nfvPPA_FTLD_vs_lvPPA_FTLD | semi_voyelles_cv | 0,001 | 1,00 | large |
| Pathotype | nfvPPA_FTLD_vs_lvPPA_FTLD | semi_voyelles_iqr | 0,001 | 1,00 | large |
| Pathotype | nfvPPA_FTLD_vs_lvPPA_FTLD | nasales_skewness | 0,001 | 1,00 | large |
| Pathotype | lvPPA_FTLD_vs_lvPPA_AD | occlusives_kurtosis | 0,001 | 0,90 | large |
| Pathotype | lvPPA_FTLD_vs_lvPPA_AD | liquides_cv | 0,001 | 0,90 | large |
| Pathotype | nfvPPA_FTLD_vs_amnestic_AD | kurt_phoneme_duration | 0,001 | 0,79 | large |
| Pathotype | lvPPA_FTLD_vs_amnestic_AD | delta_mfcc_mean_9 | 0,001 | 0,79 | large |
| Pathotype | healthy_control_vs_amnestic_AD | occlusives_skewness | 0,001 | 0,40 | medium |
| Pathotype | svPPA_FTLD_vs_healthy_control | total_duration | 0,001 | 0,68 | large |
| Pathotype | healthy_control_vs_lvPPA_AD | chroma_mean_8 | 0,001 | 0,56 | large |
| Pathotype | nfvPPA_FTLD_vs_amnestic_AD | inter_word_pause_mean | 0,001 | 0,79 | large |
| Pathotype | lvPPA_FTLD_vs_amnestic_AD | chroma_mean_7 | 0,001 | 0,79 | large |
| Pathotype | lvPPA_FTLD_vs_amnestic_AD | occlusives_skewness | 0,001 | 0,79 | large |
| Pathotype | lvPPA_FTLD_vs_amnestic_AD | voyelles_oral_std | 0,001 | 0,79 | large |
| Pathotype | lvPPA_FTLD_vs_amnestic_AD | voyelles_oral_cv | 0,001 | 0,79 | large |
| Pathotype | healthy_control_vs_amnestic_AD | delta_mfcc_mean_10 | 0,001 | 0,39 | medium |
| Pathotype | amnestic_AD_vs_lvPPA_AD | jitter_ppq5 | 0,001 | 0,60 | large |
| Pathotype | FTLD_vs_healthy_control | pause_mean_duration | 0,001 | 0,48 | medium |
| Pathotype | FTLD_vs_healthy_control | inter_word_pause_mean | 0,001 | 0,48 | medium |
| Pathotype | nfvPPA_FTLD_vs_FTLD | syllable_count | 0,001 | 0,84 | large |
| Pathotype | nfvPPA_FTLD_vs_FTLD | voyelles_oral_count | 0,001 | 0,84 | large |
| Pathotype | lvPPA_FTLD_vs_lvPPA_AD | fricatives_std | 0,001 | 0,90 | large |
| Pathotype | healthy_control_vs_lvPPA_AD | chroma_mean_7 | 0,001 | 0,56 | large |
| Pathotype | FTLD_vs_amnestic_AD | intensity_dynamic_range | 0,001 | 0,52 | large |
| Pathotype | svPPA_FTLD_vs_amnestic_AD | spectral_flux_std | 0,001 | 0,68 | large |
| Pathotype | lvPPA_FTLD_vs_healthy_control | pause_proportion | 0,001 | 0,76 | large |
| Pathotype | lvPPA_FTLD_vs_healthy_control | chroma_mean_0 | 0,001 | 0,76 | large |
| Pathotype | lvPPA_FTLD_vs_healthy_control | contrast_mean_1 | 0,001 | 0,76 | large |
| Pathotype | lvPPA_FTLD_vs_healthy_control | voyelles_oral_median | 0,001 | 0,74 | large |
| Pathotype | svPPA_FTLD_vs_lvPPA_AD | shimmer_apq5 | 0,001 | 0,80 | large |
| Pathotype | svPPA_FTLD_vs_lvPPA_AD | contrast_mean_2 | 0,001 | 0,80 | large |
| Pathotype | lvPPA_FTLD_vs_amnestic_AD | voyelles_oral_median | 0,001 | 0,78 | large |
| Pathotype | healthy_control_vs_amnestic_AD | voyelles_oral_percentile_90 | 0,001 | 0,39 | medium |
| Pathotype | FTLD_vs_amnestic_AD | delta_mfcc_mean_2 | 0,001 | 0,52 | large |
| Pathotype | FTLD_vs_lvPPA_FTLD | liquides_median | 0,001 | 0,84 | large |
| Pathotype | nfvPPA_FTLD_vs_healthy_control | median_phoneme_duration | 0,001 | 0,73 | large |
| Pathotype | nfvPPA_FTLD_vs_amnestic_AD | articulation_rate | 0,001 | 0,78 | large |
| Pathotype | svPPA_FTLD_vs_nfvPPA_FTLD | occlusives_std | 0,001 | 0,96 | large |
| Pathotype | svPPA_FTLD_vs_nfvPPA_FTLD | fricatives_percentile_90 | 0,001 | 0,96 | large |
| Pathotype | svPPA_FTLD_vs_nfvPPA_FTLD | liquides_mean | 0,001 | 0,96 | large |
| Pathotype | amnestic_AD_vs_lvPPA_AD | semi_voyelles_max | 0,001 | 0,60 | large |
| Pathotype | svPPA_FTLD_vs_nfvPPA_FTLD | fricatives_iqr | 0,001 | 0,96 | large |
| Pathotype | svPPA_FTLD_vs_nfvPPA_FTLD | nasales_iqr | 0,001 | 0,96 | large |
| Pathotype | FTLD_vs_lvPPA_FTLD | delta_mfcc_mean_12 | 0,001 | 0,84 | large |
| Pathotype | FTLD_vs_lvPPA_FTLD | spectral_flux_mean | 0,001 | 0,84 | large |
| Pathotype | FTLD_vs_lvPPA_FTLD | spectral_centroid_mean | 0,001 | 0,84 | large |
| Pathotype | FTLD_vs_lvPPA_FTLD | rolloff_mean | 0,001 | 0,84 | large |
| Pathotype | svPPA_FTLD_vs_FTLD | f3 | 0,001 | 0,73 | large |
| Pathotype | amnestic_AD_vs_lvPPA_AD | f1_cv | 0,001 | 0,60 | large |
| Pathotype | amnestic_AD_vs_lvPPA_AD | hnr_std,1 | 0,001 | 0,60 | large |
| Pathotype | svPPA_FTLD_vs_FTLD | chroma_mean_0 | 0,001 | 0,73 | large |
| Pathotype | svPPA_FTLD_vs_lvPPA_FTLD | semi_voyelles_count | 0,001 | 0,94 | large |
| Pathotype | lvPPA_FTLD_vs_lvPPA_AD | semi_voyelles_max | 0,001 | 0,86 | large |
| Pathotype | healthy_control_vs_lvPPA_AD | fricatives_median | 0,001 | 0,55 | large |
| Pathotype | lvPPA_FTLD_vs_amnestic_AD | f3 | 0,001 | 0,78 | large |
| Pathotype | nfvPPA_FTLD_vs_amnestic_AD | delta_mfcc_mean_12 | 0,001 | 0,78 | large |
| Pathotype | healthy_control_vs_lvPPA_AD | fricatives_std | 0,001 | 0,55 | large |
| Pathotype | svPPA_FTLD_vs_lvPPA_FTLD | occlusives_min | 0,001 | 0,94 | large |
| Pathotype | FTLD_vs_amnestic_AD | spectral_flux | 0,001 | 0,52 | large |
| Pathotype | FTLD_vs_lvPPA_AD | jitter_rap | 0,001 | 0,65 | large |
| Pathotype | FTLD_vs_healthy_control | contrast_mean_1 | 0,001 | 0,47 | medium |
| Pathotype | svPPA_FTLD_vs_lvPPA_FTLD | semi_voyelles_std | 0,001 | 0,90 | large |
| Pathotype | svPPA_FTLD_vs_lvPPA_FTLD | semi_voyelles_cv | 0,001 | 0,90 | large |
| Pathotype | lvPPA_FTLD_vs_amnestic_AD | delta_mfcc_mean_11 | 0,001 | 0,78 | large |
| Pathotype | svPPA_FTLD_vs_lvPPA_FTLD | liquides_skewness | 0,001 | 0,94 | large |
| Pathotype | nfvPPA_FTLD_vs_FTLD | occlusives_iqr | 0,001 | 0,83 | large |
| Pathotype | svPPA_FTLD_vs_lvPPA_FTLD | min_phoneme_duration | 0,001 | 0,94 | large |
| Pathotype | nfvPPA_FTLD_vs_FTLD | speech_rate_phonemes_per_sec | 0,001 | 0,83 | large |
| Pathotype | nfvPPA_FTLD_vs_FTLD | rate_speech_phonemes | 0,001 | 0,83 | large |
| Pathotype | nfvPPA_FTLD_vs_healthy_control | liquides_mean | 0,001 | 0,74 | large |
| Pathotype | nfvPPA_FTLD_vs_healthy_control | pause_frequency | 0,001 | 0,74 | large |
| Pathotype | lvPPA_FTLD_vs_healthy_control | cv_phoneme_duration | 0,001 | 0,74 | large |
| Pathotype | svPPA_FTLD_vs_lvPPA_AD | fricatives_median | 0,001 | 0,79 | large |
| Pathotype | nfvPPA_FTLD_vs_lvPPA_FTLD | liquides_count | 0,001 | 1,00 | large |
| Pathotype | svPPA_FTLD_vs_lvPPA_AD | fricatives_skewness | 0,001 | 0,79 | large |
| Pathotype | svPPA_FTLD_vs_amnestic_AD | voyelles_oral_percentile_10 | 0,001 | 0,63 | large |
| Pathotype | FTLD_vs_amnestic_AD | f1_cv | 0,001 | 0,51 | large |
| Pathotype | svPPA_FTLD_vs_lvPPA_FTLD | spectral_instability | 0,001 | 0,94 | large |
| Pathotype | svPPA_FTLD_vs_lvPPA_FTLD | zcr_mean | 0,001 | 0,94 | large |
| Pathotype | svPPA_FTLD_vs_lvPPA_FTLD | intensity_slope | 0,001 | 0,94 | large |
| Pathotype | svPPA_FTLD_vs_lvPPA_AD | contrast_mean_0 | 0,001 | 0,79 | large |
| Pathotype | svPPA_FTLD_vs_lvPPA_FTLD | spectral_flux | 0,001 | 0,94 | large |
| Pathotype | svPPA_FTLD_vs_lvPPA_FTLD | spectral_flux_mean | 0,001 | 0,94 | large |
| Pathotype | svPPA_FTLD_vs_lvPPA_FTLD | semi_voyelles_iqr | 0,001 | 0,90 | large |
| Pathotype | nfvPPA_FTLD_vs_amnestic_AD | nasales_min | 0,001 | 0,76 | large |
| Pathotype | lvPPA_FTLD_vs_lvPPA_AD | voyelles_oral_skewness | 0,001 | 0,88 | large |
| Pathotype | lvPPA_FTLD_vs_lvPPA_AD | nasales_percentile_90 | 0,001 | 0,88 | large |
| Pathotype | svPPA_FTLD_vs_amnestic_AD | zcr_mean | 0,001 | 0,67 | large |
| Pathotype | lvPPA_FTLD_vs_healthy_control | fricatives_percentile_90 | 0,001 | 0,74 | large |
| Pathotype | svPPA_FTLD_vs_amnestic_AD | kurt_phoneme_duration | 0,001 | 0,67 | large |
| Pathotype | lvPPA_FTLD_vs_healthy_control | spectral_instability | 0,001 | 0,74 | large |
| Pathotype | lvPPA_FTLD_vs_healthy_control | chroma_mean_2 | 0,001 | 0,74 | large |
| Pathotype | lvPPA_FTLD_vs_lvPPA_AD | shimmer_local | 0,001 | 0,88 | large |
| Pathotype | lvPPA_FTLD_vs_lvPPA_AD | shimmer_apq5 | 0,001 | 0,88 | large |
| Pathotype | lvPPA_FTLD_vs_lvPPA_AD | zcr_mean | 0,001 | 0,88 | large |
| Pathotype | lvPPA_FTLD_vs_lvPPA_AD | spectral_centroid_mean | 0,001 | 0,88 | large |
| Pathotype | svPPA_FTLD_vs_FTLD | occlusives_kurtosis | 0,001 | 0,72 | large |
| Pathotype | nfvPPA_FTLD_vs_lvPPA_AD | chroma_mean_1 | 0,001 | 0,88 | large |
| Pathotype | nfvPPA_FTLD_vs_amnestic_AD | contrast_mean_5 | 0,001 | 0,77 | large |
| Pathotype | FTLD_vs_lvPPA_AD | semi_voyelles_percentile_90 | 0,001 | 0,65 | large |
| Pathotype | nfvPPA_FTLD_vs_lvPPA_AD | f0_std | 0,001 | 0,88 | large |
| Pathotype | svPPA_FTLD_vs_lvPPA_FTLD | delta_mfcc_mean_6 | 0,001 | 0,94 | large |
| Pathotype | svPPA_FTLD_vs_lvPPA_FTLD | chroma_mean_0 | 0,001 | 0,94 | large |
| Pathotype | FTLD_vs_amnestic_AD | std_phoneme_duration | 0,001 | 0,51 | large |
| Pathotype | FTLD_vs_lvPPA_FTLD | fricatives_std | 0,001 | 0,82 | large |
| Pathotype | svPPA_FTLD_vs_nfvPPA_FTLD | delta_mfcc_mean_4 | 0,001 | 0,94 | large |
| Pathotype | svPPA_FTLD_vs_nfvPPA_FTLD | delta_mfcc_mean_6 | 0,001 | 0,94 | large |
| Pathotype | svPPA_FTLD_vs_nfvPPA_FTLD | b3 | 0,001 | 0,94 | large |
| Pathotype | svPPA_FTLD_vs_lvPPA_FTLD | occlusives_mean | 0,001 | 0,94 | large |
| Pathotype | FTLD_vs_lvPPA_AD | jitter_local | 0,001 | 0,65 | large |
| Pathotype | FTLD_vs_lvPPA_AD | chroma_mean_1 | 0,001 | 0,65 | large |
| Pathotype | FTLD_vs_lvPPA_FTLD | voyelles_oral_cv | 0,001 | 0,82 | large |
| Pathotype | nfvPPA_FTLD_vs_amnestic_AD | jitter_local | 0,001 | 0,77 | large |
| Pathotype | healthy_control_vs_amnestic_AD | hnr_std | 0,001 | 0,38 | medium |
| Pathotype | svPPA_FTLD_vs_lvPPA_FTLD | max_phoneme_duration | 0,001 | 0,94 | large |
| Pathotype | svPPA_FTLD_vs_lvPPA_FTLD | fricatives_max | 0,001 | 0,94 | large |
| Pathotype | FTLD_vs_amnestic_AD | occlusives_median | 0,001 | 0,51 | large |
| Pathotype | lvPPA_FTLD_vs_healthy_control | pause_median_duration | 0,001 | 0,74 | large |
| Pathotype | lvPPA_FTLD_vs_healthy_control | inter_word_pause_median | 0,001 | 0,74 | large |
| Pathotype | lvPPA_FTLD_vs_healthy_control | occlusives_skewness | 0,001 | 0,74 | large |
| Pathotype | lvPPA_FTLD_vs_healthy_control | voyelles_oral_std | 0,001 | 0,74 | large |
| Pathotype | FTLD_vs_amnestic_AD | chroma_mean_3 | 0,002 | 0,51 | large |
| Pathotype | FTLD_vs_lvPPA_AD | fricatives_median | 0,002 | 0,64 | large |
| Pathotype | nfvPPA_FTLD_vs_amnestic_AD | semi_voyelles_median | 0,002 | 0,76 | large |
| Pathotype | svPPA_FTLD_vs_nfvPPA_FTLD | occlusives_percentile_90 | 0,002 | 0,93 | large |
| Pathotype | nfvPPA_FTLD_vs_lvPPA_AD | semi_voyelles_max | 0,002 | 0,86 | large |
| Pathotype | nfvPPA_FTLD_vs_FTLD | pause_ratio | 0,002 | 0,82 | large |
| Pathotype | nfvPPA_FTLD_vs_healthy_control | pause_min_duration | 0,002 | 0,73 | large |
| Pathotype | nfvPPA_FTLD_vs_healthy_control | inter_word_pause_min | 0,002 | 0,73 | large |
| Pathotype | healthy_control_vs_lvPPA_AD | liquides_iqr | 0,002 | 0,54 | large |
| Pathotype | lvPPA_FTLD_vs_amnestic_AD | fricatives_cv | 0,002 | 0,76 | large |
| Pathotype | svPPA_FTLD_vs_healthy_control | occlusives_mean | 0,002 | 0,62 | large |
| Pathotype | lvPPA_FTLD_vs_healthy_control | fricatives_iqr | 0,002 | 0,73 | large |
| Pathotype | lvPPA_FTLD_vs_healthy_control | occlusives_kurtosis | 0,002 | 0,73 | large |
| Pathotype | nfvPPA_FTLD_vs_lvPPA_FTLD | num_pauses | 0,002 | 1,00 | large |
| Pathotype | nfvPPA_FTLD_vs_lvPPA_FTLD | num_inter_word_pauses | 0,002 | 1,00 | large |
| Pathotype | nfvPPA_FTLD_vs_lvPPA_FTLD | num_inter_pauses | 0,002 | 1,00 | large |
| Pathotype | nfvPPA_FTLD_vs_lvPPA_FTLD | long_pause_count | 0,002 | 1,00 | large |
| Pathotype | nfvPPA_FTLD_vs_lvPPA_FTLD | voyelles_oral_kurtosis | 0,002 | 1,00 | large |
| Pathotype | nfvPPA_FTLD_vs_lvPPA_FTLD | occlusives_count | 0,002 | 1,00 | large |
| Pathotype | nfvPPA_FTLD_vs_lvPPA_FTLD | liquides_std | 0,002 | 1,00 | large |
| Pathotype | nfvPPA_FTLD_vs_lvPPA_FTLD | liquides_cv | 0,002 | 1,00 | large |
| Pathotype | nfvPPA_FTLD_vs_lvPPA_FTLD | liquides_iqr | 0,002 | 1,00 | large |
| Pathotype | nfvPPA_FTLD_vs_lvPPA_FTLD | nasales_std | 0,002 | 1,00 | large |
| Pathotype | nfvPPA_FTLD_vs_lvPPA_FTLD | nasales_cv | 0,002 | 1,00 | large |
| Pathotype | nfvPPA_FTLD_vs_lvPPA_FTLD | nasales_iqr | 0,002 | 1,00 | large |
| Pathotype | lvPPA_FTLD_vs_amnestic_AD | occlusives_kurtosis | 0,002 | 0,76 | large |
| Pathotype | svPPA_FTLD_vs_lvPPA_AD | liquides_mean | 0,002 | 0,77 | large |
| Pathotype | lvPPA_FTLD_vs_amnestic_AD | delta_mfcc_mean_7 | 0,002 | 0,76 | large |
| Pathotype | lvPPA_FTLD_vs_amnestic_AD | voyelles_oral_mean | 0,002 | 0,76 | large |
| Pathotype | nfvPPA_FTLD_vs_amnestic_AD | b1 | 0,002 | 0,76 | large |
| Pathotype | svPPA_FTLD_vs_lvPPA_AD | shimmer_apq3 | 0,002 | 0,77 | large |
| Pathotype | svPPA_FTLD_vs_lvPPA_AD | shimmer_dda | 0,002 | 0,77 | large |
| Pathotype | healthy_control_vs_amnestic_AD | voyelles_oral_count | 0,002 | 0,38 | medium |
| Pathotype | svPPA_FTLD_vs_amnestic_AD | voyelles_oral_median | 0,002 | 0,65 | large |
| Pathotype | nfvPPA_FTLD_vs_FTLD | occlusives_percentile_10 | 0,002 | 0,80 | large |
| Pathotype | svPPA_FTLD_vs_nfvPPA_FTLD | pause_mean_duration | 0,002 | 0,93 | large |
| Pathotype | svPPA_FTLD_vs_nfvPPA_FTLD | pause_std_duration | 0,002 | 0,93 | large |
| Pathotype | svPPA_FTLD_vs_nfvPPA_FTLD | inter_word_pause_mean | 0,002 | 0,93 | large |
| Pathotype | svPPA_FTLD_vs_nfvPPA_FTLD | inter_word_pause_std | 0,002 | 0,93 | large |
| Pathotype | svPPA_FTLD_vs_lvPPA_AD | f0_max | 0,002 | 0,77 | large |
| Pathotype | svPPA_FTLD_vs_lvPPA_AD | hnr_mean | 0,002 | 0,77 | large |
| Pathotype | svPPA_FTLD_vs_lvPPA_AD | hnr_mean,1 | 0,002 | 0,77 | large |
| Pathotype | svPPA_FTLD_vs_healthy_control | intensity_dynamic_range | 0,002 | 0,62 | large |
| Pathotype | svPPA_FTLD_vs_healthy_control | f3_cv,1 | 0,002 | 0,62 | large |
| Pathotype | healthy_control_vs_amnestic_AD | pause_ratio | 0,002 | 0,38 | medium |
| Pathotype | healthy_control_vs_amnestic_AD | jitter_ppq5 | 0,002 | 0,38 | medium |
| Pathotype | lvPPA_FTLD_vs_healthy_control | pause_std_duration | 0,002 | 0,73 | large |
| Pathotype | lvPPA_FTLD_vs_healthy_control | inter_word_pause_std | 0,002 | 0,73 | large |
| Pathotype | svPPA_FTLD_vs_amnestic_AD | contrast_mean_4 | 0,002 | 0,66 | large |
| Pathotype | FTLD_vs_lvPPA_FTLD | delta_mfcc_mean_9 | 0,002 | 0,81 | large |
| Pathotype | FTLD_vs_lvPPA_FTLD | chroma_mean_8 | 0,002 | 0,81 | large |
| Pathotype | FTLD_vs_lvPPA_FTLD | intensity_slope | 0,002 | 0,81 | large |
| Pathotype | healthy_control_vs_lvPPA_AD | f0_max | 0,002 | 0,54 | large |
| Pathotype | FTLD_vs_lvPPA_FTLD | spectral_flux | 0,002 | 0,81 | large |
| Pathotype | amnestic_AD_vs_lvPPA_AD | chroma_mean_2 | 0,002 | 0,58 | large |
| Pathotype | healthy_control_vs_amnestic_AD | num_inter_pauses | 0,002 | 0,38 | medium |
| Pathotype | nfvPPA_FTLD_vs_lvPPA_AD | semi_voyelles_std | 0,002 | 0,86 | large |
| Pathotype | nfvPPA_FTLD_vs_lvPPA_AD | semi_voyelles_percentile_90 | 0,002 | 0,86 | large |
| Pathotype | amnestic_AD_vs_lvPPA_AD | fricatives_count | 0,002 | 0,57 | large |
| Pathotype | lvPPA_FTLD_vs_lvPPA_AD | fricatives_iqr | 0,002 | 0,86 | large |
| Pathotype | FTLD_vs_amnestic_AD | contrast_mean_3 | 0,002 | 0,50 | large |
| Pathotype | nfvPPA_FTLD_vs_lvPPA_FTLD | semi_voyelles_count | 0,002 | 1,00 | large |
| Pathotype | healthy_control_vs_amnestic_AD | pause_median_duration | 0,002 | 0,38 | medium |
| Pathotype | healthy_control_vs_amnestic_AD | inter_word_pause_median | 0,002 | 0,38 | medium |
| Pathotype | amnestic_AD_vs_lvPPA_AD | syllable_count | 0,002 | 0,57 | large |
| Pathotype | lvPPA_FTLD_vs_healthy_control | delta_mfcc_mean_8 | 0,002 | 0,72 | large |
| Pathotype | nfvPPA_FTLD_vs_healthy_control | voyelles_oral_median | 0,002 | 0,71 | large |
| Pathotype | FTLD_vs_amnestic_AD | max_phoneme_duration | 0,002 | 0,50 | medium |
| Pathotype | nfvPPA_FTLD_vs_lvPPA_AD | intensity_slope | 0,002 | 0,86 | large |
| Pathotype | svPPA_FTLD_vs_FTLD | delta_mfcc_mean_2 | 0,002 | 0,70 | large |
| Pathotype | lvPPA_FTLD_vs_amnestic_AD | articulation_rate | 0,002 | 0,75 | large |
| Pathotype | nfvPPA_FTLD_vs_lvPPA_FTLD | semi_voyelles_median | 0,002 | 1,00 | large |
| Pathotype | FTLD_vs_healthy_control | liquides_count | 0,002 | 0,45 | medium |
| Pathotype | svPPA_FTLD_vs_nfvPPA_FTLD | spectral_centroid_mean | 0,002 | 0,91 | large |
| Pathotype | svPPA_FTLD_vs_nfvPPA_FTLD | f3 | 0,002 | 0,91 | large |
| Pathotype | svPPA_FTLD_vs_lvPPA_FTLD | occlusives_median | 0,002 | 0,91 | large |
| Pathotype | nfvPPA_FTLD_vs_lvPPA_FTLD | shimmer_local | 0,002 | 1,00 | large |
| Pathotype | nfvPPA_FTLD_vs_lvPPA_FTLD | shimmer_apq3 | 0,002 | 1,00 | large |
| Pathotype | nfvPPA_FTLD_vs_lvPPA_FTLD | shimmer_apq5 | 0,002 | 1,00 | large |
| Pathotype | nfvPPA_FTLD_vs_lvPPA_FTLD | shimmer_dda | 0,002 | 1,00 | large |
| Pathotype | nfvPPA_FTLD_vs_lvPPA_FTLD | hnr_mean | 0,002 | 1,00 | large |
| Pathotype | nfvPPA_FTLD_vs_lvPPA_FTLD | delta_mfcc_mean_6 | 0,002 | 1,00 | large |
| Pathotype | nfvPPA_FTLD_vs_lvPPA_FTLD | delta_mfcc_mean_9 | 0,002 | 1,00 | large |
| Pathotype | nfvPPA_FTLD_vs_lvPPA_FTLD | chroma_mean_1 | 0,002 | 1,00 | large |
| Pathotype | nfvPPA_FTLD_vs_lvPPA_FTLD | chroma_mean_2 | 0,002 | 1,00 | large |
| Pathotype | nfvPPA_FTLD_vs_lvPPA_FTLD | chroma_mean_3 | 0,002 | 1,00 | large |
| Pathotype | nfvPPA_FTLD_vs_lvPPA_FTLD | chroma_mean_4 | 0,002 | 1,00 | large |
| Pathotype | nfvPPA_FTLD_vs_lvPPA_FTLD | spectral_centroid_mean | 0,002 | 1,00 | large |
| Pathotype | nfvPPA_FTLD_vs_lvPPA_FTLD | rolloff_mean | 0,002 | 1,00 | large |
| Pathotype | nfvPPA_FTLD_vs_lvPPA_FTLD | hnr_mean,1 | 0,002 | 1,00 | large |
| Pathotype | nfvPPA_FTLD_vs_lvPPA_FTLD | occlusives_max | 0,002 | 1,00 | large |
| Pathotype | lvPPA_FTLD_vs_healthy_control | pause_mean_duration | 0,002 | 0,72 | large |
| Pathotype | lvPPA_FTLD_vs_healthy_control | inter_word_pause_mean | 0,002 | 0,72 | large |
| Pathotype | nfvPPA_FTLD_vs_lvPPA_FTLD | spectral_flux | 0,002 | 1,00 | large |
| Pathotype | nfvPPA_FTLD_vs_lvPPA_FTLD | kurt_phoneme_duration | 0,002 | 1,00 | large |
| Pathotype | nfvPPA_FTLD_vs_lvPPA_FTLD | occlusives_mean | 0,002 | 1,00 | large |
| Pathotype | nfvPPA_FTLD_vs_lvPPA_FTLD | occlusives_percentile_90 | 0,002 | 1,00 | large |
| Pathotype | nfvPPA_FTLD_vs_lvPPA_FTLD | liquides_max | 0,002 | 1,00 | large |
| Pathotype | nfvPPA_FTLD_vs_lvPPA_FTLD | liquides_percentile_90 | 0,002 | 1,00 | large |
| Pathotype | healthy_control_vs_amnestic_AD | pause_proportion | 0,002 | 0,37 | medium |
| Pathotype | svPPA_FTLD_vs_lvPPA_FTLD | total_duration | 0,002 | 0,94 | large |
| Pathotype | FTLD_vs_lvPPA_AD | pause_min_duration | 0,002 | 0,62 | large |
| Pathotype | FTLD_vs_lvPPA_AD | inter_word_pause_min | 0,002 | 0,62 | large |
| Pathotype | FTLD_vs_healthy_control | semi_voyelles_min | 0,002 | 0,43 | medium |
| Pathotype | nfvPPA_FTLD_vs_lvPPA_FTLD | zcr_mean | 0,002 | 1,00 | large |
| Pathotype | nfvPPA_FTLD_vs_lvPPA_FTLD | contrast_mean_3 | 0,002 | 1,00 | large |
| Pathotype | nfvPPA_FTLD_vs_lvPPA_FTLD | rmse_mean | 0,002 | 1,00 | large |
| Pathotype | nfvPPA_FTLD_vs_lvPPA_FTLD | intensity_mean | 0,002 | 1,00 | large |
| Pathotype | nfvPPA_FTLD_vs_lvPPA_FTLD | intensity_slope | 0,002 | 1,00 | large |
| Pathotype | nfvPPA_FTLD_vs_lvPPA_FTLD | hnr_std,1 | 0,002 | 1,00 | large |
| Pathotype | lvPPA_FTLD_vs_amnestic_AD | delta_mfcc_mean_1 | 0,002 | 0,75 | large |
| Pathotype | healthy_control_vs_lvPPA_AD | voyelles_oral_percentile_90 | 0,002 | 0,53 | large |
| Pathotype | amnestic_AD_vs_lvPPA_AD | zcr_mean | 0,002 | 0,57 | large |
| Pathotype | nfvPPA_FTLD_vs_lvPPA_FTLD | speech_duration | 0,002 | 1,00 | large |
| Pathotype | nfvPPA_FTLD_vs_lvPPA_FTLD | phoneme_count | 0,002 | 1,00 | large |
| Pathotype | nfvPPA_FTLD_vs_lvPPA_FTLD | syllable_count | 0,002 | 1,00 | large |
| Pathotype | nfvPPA_FTLD_vs_lvPPA_FTLD | num_speech_phonemes | 0,002 | 1,00 | large |
| Pathotype | nfvPPA_FTLD_vs_lvPPA_FTLD | mean_phoneme_duration | 0,002 | 1,00 | large |
| Pathotype | nfvPPA_FTLD_vs_lvPPA_FTLD | median_phoneme_duration | 0,002 | 1,00 | large |
| Pathotype | nfvPPA_FTLD_vs_lvPPA_FTLD | max_phoneme_duration | 0,002 | 1,00 | large |
| Pathotype | nfvPPA_FTLD_vs_lvPPA_FTLD | speech_rate_phonemes_per_sec | 0,002 | 1,00 | large |
| Pathotype | nfvPPA_FTLD_vs_lvPPA_FTLD | rate_speech_phonemes | 0,002 | 1,00 | large |
| Pathotype | nfvPPA_FTLD_vs_lvPPA_FTLD | voyelles_oral_count | 0,002 | 1,00 | large |
| Pathotype | nfvPPA_FTLD_vs_lvPPA_FTLD | voyelles_oral_skewness | 0,002 | 1,00 | large |
| Pathotype | nfvPPA_FTLD_vs_lvPPA_FTLD | semi_voyelles_mean | 0,002 | 1,00 | large |
| Pathotype | nfvPPA_FTLD_vs_lvPPA_FTLD | semi_voyelles_max | 0,002 | 1,00 | large |
| Pathotype | nfvPPA_FTLD_vs_lvPPA_FTLD | semi_voyelles_percentile_90 | 0,002 | 1,00 | large |
| Pathotype | nfvPPA_FTLD_vs_lvPPA_FTLD | occlusives_median | 0,002 | 1,00 | large |
| Pathotype | nfvPPA_FTLD_vs_lvPPA_FTLD | occlusives_std | 0,002 | 1,00 | large |
| Pathotype | nfvPPA_FTLD_vs_lvPPA_FTLD | occlusives_iqr | 0,002 | 1,00 | large |
| Pathotype | nfvPPA_FTLD_vs_lvPPA_FTLD | fricatives_count | 0,002 | 1,00 | large |
| Pathotype | nfvPPA_FTLD_vs_lvPPA_FTLD | fricatives_percentile_90 | 0,002 | 1,00 | large |
| Pathotype | nfvPPA_FTLD_vs_lvPPA_FTLD | liquides_mean | 0,002 | 1,00 | large |
| Pathotype | nfvPPA_FTLD_vs_lvPPA_FTLD | nasales_count | 0,002 | 1,00 | large |
| Pathotype | nfvPPA_FTLD_vs_lvPPA_FTLD | nasales_percentile_90 | 0,002 | 1,00 | large |
| Pathotype | svPPA_FTLD_vs_healthy_control | voyelles_oral_iqr | 0,002 | 0,61 | large |
| Pathotype | amnestic_AD_vs_lvPPA_AD | semi_voyelles_std | 0,002 | 0,57 | large |
| Pathotype | amnestic_AD_vs_lvPPA_AD | delta_mfcc_mean_5 | 0,002 | 0,57 | large |
| Pathotype | FTLD_vs_lvPPA_FTLD | voyelles_oral_median | 0,002 | 0,80 | large |
| Pathotype | svPPA_FTLD_vs_lvPPA_AD | total_duration | 0,002 | 0,78 | large |
| Pathotype | nfvPPA_FTLD_vs_healthy_control | pause_max_duration | 0,002 | 0,71 | large |
| Pathotype | nfvPPA_FTLD_vs_healthy_control | inter_word_pause_max | 0,002 | 0,71 | large |
| Pathotype | lvPPA_FTLD_vs_healthy_control | voyelles_oral_mean | 0,002 | 0,71 | large |
| Pathotype | lvPPA_FTLD_vs_healthy_control | pause_frequency | 0,002 | 0,71 | large |
| Pathotype | lvPPA_FTLD_vs_healthy_control | spectral_centroid_std | 0,002 | 0,71 | large |
| Pathotype | nfvPPA_FTLD_vs_healthy_control | hnr_std | 0,002 | 0,71 | large |
| Pathotype | nfvPPA_FTLD_vs_healthy_control | hnr_std,1 | 0,002 | 0,71 | large |
| Pathotype | FTLD_vs_lvPPA_FTLD | intensity_dynamic_range | 0,002 | 0,80 | large |
| Pathotype | FTLD_vs_lvPPA_FTLD | std_phoneme_duration | 0,002 | 0,80 | large |
| Pathotype | nfvPPA_FTLD_vs_lvPPA_FTLD | nasales_max | 0,002 | 1,00 | large |
| Pathotype | healthy_control_vs_amnestic_AD | delta_mfcc_mean_2 | 0,002 | 0,37 | medium |
| Pathotype | amnestic_AD_vs_lvPPA_AD | chroma_mean_0 | 0,002 | 0,56 | large |
| Pathotype | svPPA_FTLD_vs_nfvPPA_FTLD | semi_voyelles_std | 0,002 | 0,90 | large |
| Pathotype | healthy_control_vs_lvPPA_AD | num_intra_word_pauses | 0,002 | 0,14 | small |
| Pathotype | healthy_control_vs_lvPPA_AD | voyelles_nasales_count | 0,002 | 0,14 | small |
| Pathotype | amnestic_AD_vs_lvPPA_AD | f0_std | 0,002 | 0,56 | large |
| Pathotype | healthy_control_vs_amnestic_AD | b3 | 0,002 | 0,37 | medium |
| Pathotype | healthy_control_vs_amnestic_AD | liquides_percentile_10 | 0,002 | 0,36 | medium |
| Pathotype | nfvPPA_FTLD_vs_FTLD | semi_voyelles_percentile_10 | 0,002 | 0,73 | large |
| Pathotype | nfvPPA_FTLD_vs_amnestic_AD | pause_proportion | 0,002 | 0,74 | large |
| Pathotype | lvPPA_FTLD_vs_amnestic_AD | fricatives_iqr | 0,002 | 0,74 | large |
| Pathotype | healthy_control_vs_lvPPA_AD | chroma_mean_6 | 0,002 | 0,52 | large |
| Pathotype | FTLD_vs_lvPPA_AD | syllable_count | 0,002 | 0,62 | large |
| Pathotype | svPPA_FTLD_vs_amnestic_AD | delta_mfcc_mean_5 | 0,002 | 0,64 | large |
| Pathotype | nfvPPA_FTLD_vs_amnestic_AD | fricatives_kurtosis | 0,002 | 0,74 | large |
| Pathotype | svPPA_FTLD_vs_nfvPPA_FTLD | occlusives_mean | 0,002 | 0,90 | large |
| Pathotype | svPPA_FTLD_vs_nfvPPA_FTLD | liquides_std | 0,002 | 0,90 | large |
| Pathotype | svPPA_FTLD_vs_nfvPPA_FTLD | liquides_percentile_90 | 0,002 | 0,90 | large |
| Pathotype | svPPA_FTLD_vs_nfvPPA_FTLD | liquides_iqr | 0,002 | 0,90 | large |
| Pathotype | lvPPA_FTLD_vs_lvPPA_AD | nasales_max | 0,002 | 0,84 | large |
| Pathotype | amnestic_AD_vs_lvPPA_AD | hnr_std | 0,002 | 0,56 | large |
| Pathotype | lvPPA_FTLD_vs_healthy_control | delta_mfcc_mean_12 | 0,002 | 0,71 | large |
| Pathotype | lvPPA_FTLD_vs_healthy_control | contrast_mean_2 | 0,002 | 0,71 | large |
| Pathotype | svPPA_FTLD_vs_healthy_control | voyelles_oral_median | 0,002 | 0,59 | large |
| Pathotype | amnestic_AD_vs_lvPPA_AD | syllable_rate_per_sec | 0,002 | 0,56 | large |
| Pathotype | lvPPA_FTLD_vs_lvPPA_AD | f2_cv | 0,002 | 0,84 | large |
| Pathotype | lvPPA_FTLD_vs_lvPPA_AD | chroma_mean_7 | 0,002 | 0,84 | large |
| Pathotype | lvPPA_FTLD_vs_lvPPA_AD | chroma_mean_8 | 0,002 | 0,84 | large |
| Pathotype | lvPPA_FTLD_vs_lvPPA_AD | rolloff_mean | 0,002 | 0,84 | large |
| Pathotype | lvPPA_FTLD_vs_lvPPA_AD | delta_mfcc_mean_5 | 0,002 | 0,84 | large |
| Pathotype | FTLD_vs_healthy_control | shimmer_apq5 | 0,002 | 0,44 | medium |
| Pathotype | svPPA_FTLD_vs_amnestic_AD | occlusives_max | 0,002 | 0,63 | large |
| Pathotype | healthy_control_vs_amnestic_AD | fricatives_kurtosis | 0,002 | 0,37 | medium |
| Pathotype | svPPA_FTLD_vs_lvPPA_AD | nasales_kurtosis | 0,002 | 0,74 | large |
| Pathotype | nfvPPA_FTLD_vs_healthy_control | semi_voyelles_count | 0,002 | 0,70 | large |
| Pathotype | healthy_control_vs_amnestic_AD | liquides_cv | 0,002 | 0,36 | medium |
| Pathotype | nfvPPA_FTLD_vs_FTLD | voyelles_oral_percentile_10 | 0,002 | 0,72 | large |
| Pathotype | FTLD_vs_healthy_control | jitter_rap | 0,002 | 0,44 | medium |
| Pathotype | FTLD_vs_lvPPA_FTLD | contrast_mean_0 | 0,002 | 0,78 | large |
| Pathotype | svPPA_FTLD_vs_amnestic_AD | speech_duration | 0,002 | 0,63 | large |
| Pathotype | nfvPPA_FTLD_vs_healthy_control | num_inter_pauses | 0,002 | 0,70 | large |
| Pathotype | FTLD_vs_lvPPA_FTLD | zcr_mean | 0,002 | 0,78 | large |
| Pathotype | FTLD_vs_lvPPA_FTLD | contrast_mean_4 | 0,002 | 0,78 | large |
| Pathotype | nfvPPA_FTLD_vs_FTLD | jitter_local | 0,002 | 0,78 | large |
| Pathotype | svPPA_FTLD_vs_lvPPA_AD | spectral_centroid_slope | 0,002 | 0,74 | large |
| Pathotype | nfvPPA_FTLD_vs_amnestic_AD | fricatives_iqr | 0,002 | 0,73 | large |
| Pathotype | FTLD_vs_lvPPA_FTLD | nasales_mean | 0,002 | 0,78 | large |
| Pathotype | nfvPPA_FTLD_vs_healthy_control | voyelles_oral_cv | 0,002 | 0,70 | large |
| Pathotype | nfvPPA_FTLD_vs_healthy_control | semi_voyelles_cv | 0,002 | 0,70 | large |
| Pathotype | svPPA_FTLD_vs_healthy_control | voyelles_oral_percentile_10 | 0,002 | 0,55 | large |
| Pathotype | nfvPPA_FTLD_vs_healthy_control | fricatives_kurtosis | 0,002 | 0,70 | large |
| Pathotype | nfvPPA_FTLD_vs_healthy_control | f0_mean | 0,002 | 0,70 | large |
| Pathotype | healthy_control_vs_amnestic_AD | kurt_phoneme_duration | 0,003 | 0,36 | medium |
| Pathotype | FTLD_vs_lvPPA_AD | pause_median_duration | 0,003 | 0,61 | large |
| Pathotype | FTLD_vs_lvPPA_AD | inter_word_pause_median | 0,003 | 0,61 | large |
| Pathotype | svPPA_FTLD_vs_healthy_control | nasales_kurtosis | 0,003 | 0,60 | large |
| Pathotype | nfvPPA_FTLD_vs_amnestic_AD | skew_phoneme_duration | 0,003 | 0,73 | large |
| Pathotype | healthy_control_vs_amnestic_AD | num_inter_word_pauses | 0,003 | 0,36 | medium |
| Pathotype | lvPPA_FTLD_vs_amnestic_AD | semi_voyelles_skewness | 0,003 | 0,72 | large |
| Pathotype | lvPPA_FTLD_vs_amnestic_AD | pause_cv | 0,003 | 0,73 | large |
| Pathotype | FTLD_vs_lvPPA_AD | jitter_ppq5 | 0,003 | 0,61 | large |
| Pathotype | FTLD_vs_lvPPA_AD | f0_std | 0,003 | 0,61 | large |
| Pathotype | lvPPA_FTLD_vs_healthy_control | semi_voyelles_skewness | 0,003 | 0,70 | large |
| Pathotype | nfvPPA_FTLD_vs_healthy_control | num_pauses | 0,003 | 0,70 | large |
| Pathotype | nfvPPA_FTLD_vs_healthy_control | num_inter_word_pauses | 0,003 | 0,70 | large |
| Pathotype | healthy_control_vs_lvPPA_AD | median_phoneme_duration | 0,003 | 0,50 | large |
| Pathotype | lvPPA_FTLD_vs_healthy_control | voyelles_oral_percentile_90 | 0,003 | 0,70 | large |
| Pathotype | nfvPPA_FTLD_vs_FTLD | semi_voyelles_iqr | 0,003 | 0,78 | large |
| Pathotype | healthy_control_vs_lvPPA_AD | f0_std | 0,003 | 0,52 | large |
| Pathotype | svPPA_FTLD_vs_lvPPA_FTLD | delta_mfcc_mean_8 | 0,003 | 0,89 | large |
| Pathotype | svPPA_FTLD_vs_lvPPA_FTLD | chroma_mean_11 | 0,003 | 0,89 | large |
| Pathotype | svPPA_FTLD_vs_lvPPA_FTLD | spectral_centroid_std | 0,003 | 0,89 | large |
| Pathotype | svPPA_FTLD_vs_lvPPA_FTLD | f1 | 0,003 | 0,89 | large |
| Pathotype | svPPA_FTLD_vs_lvPPA_FTLD | hnr_std,1 | 0,003 | 0,89 | large |
| Pathotype | lvPPA_FTLD_vs_healthy_control | spectral_centroid_slope | 0,003 | 0,70 | large |
| Pathotype | FTLD_vs_lvPPA_AD | liquides_kurtosis | 0,003 | 0,61 | large |
| Pathotype | amnestic_AD_vs_lvPPA_AD | semi_voyelles_count | 0,003 | 0,55 | large |
| Pathotype | nfvPPA_FTLD_vs_FTLD | fricatives_percentile_90 | 0,003 | 0,78 | large |
| Pathotype | svPPA_FTLD_vs_healthy_control | semi_voyelles_skewness | 0,003 | 0,59 | large |
| Pathotype | FTLD_vs_healthy_control | contrast_mean_3 | 0,003 | 0,44 | medium |
| Pathotype | svPPA_FTLD_vs_lvPPA_FTLD | semi_voyelles_max | 0,003 | 0,89 | large |
| Pathotype | amnestic_AD_vs_lvPPA_AD | chroma_mean_6 | 0,003 | 0,55 | large |
| Pathotype | svPPA_FTLD_vs_lvPPA_FTLD | hnr_mean | 0,003 | 0,89 | large |
| Pathotype | svPPA_FTLD_vs_lvPPA_FTLD | delta_mfcc_mean_9 | 0,003 | 0,89 | large |
| Pathotype | svPPA_FTLD_vs_lvPPA_FTLD | contrast_mean_0 | 0,003 | 0,89 | large |
| Pathotype | svPPA_FTLD_vs_FTLD | total_duration | 0,003 | 0,70 | large |
| Pathotype | svPPA_FTLD_vs_lvPPA_FTLD | delta_mfcc_mean_4 | 0,003 | 0,89 | large |
| Pathotype | svPPA_FTLD_vs_lvPPA_FTLD | f2 | 0,003 | 0,89 | large |
| Pathotype | svPPA_FTLD_vs_healthy_control | nasales_mean | 0,003 | 0,59 | large |
| Pathotype | svPPA_FTLD_vs_healthy_control | chroma_mean_1 | 0,003 | 0,59 | large |
| Pathotype | nfvPPA_FTLD_vs_amnestic_AD | nasales_skewness | 0,003 | 0,72 | large |
| Pathotype | lvPPA_FTLD_vs_amnestic_AD | pause_ratio | 0,003 | 0,72 | large |
| Pathotype | svPPA_FTLD_vs_lvPPA_FTLD | semi_voyelles_mean | 0,003 | 0,89 | large |
| Pathotype | svPPA_FTLD_vs_lvPPA_FTLD | semi_voyelles_median | 0,003 | 0,89 | large |
| Pathotype | svPPA_FTLD_vs_lvPPA_FTLD | semi_voyelles_percentile_90 | 0,003 | 0,89 | large |
| Pathotype | FTLD_vs_lvPPA_AD | intensity_dynamic_range | 0,003 | 0,61 | large |
| Pathotype | svPPA_FTLD_vs_lvPPA_FTLD | nasales_mean | 0,003 | 0,89 | large |
| Pathotype | svPPA_FTLD_vs_lvPPA_FTLD | nasales_median | 0,003 | 0,89 | large |
| Pathotype | healthy_control_vs_lvPPA_AD | cv_phoneme_duration | 0,003 | 0,51 | large |
| Pathotype | svPPA_FTLD_vs_healthy_control | voyelles_oral_percentile_90 | 0,003 | 0,59 | large |
| Pathotype | lvPPA_FTLD_vs_healthy_control | f0_min | 0,003 | 0,61 | large |
| Pathotype | FTLD_vs_healthy_control | chroma_mean_8 | 0,003 | 0,43 | medium |
| Pathotype | svPPA_FTLD_vs_lvPPA_AD | phoneme_count | 0,003 | 0,73 | large |
| Pathotype | svPPA_FTLD_vs_lvPPA_AD | num_speech_phonemes | 0,003 | 0,73 | large |
| Pathotype | svPPA_FTLD_vs_lvPPA_AD | pause_min_duration | 0,003 | 0,73 | large |
| Pathotype | svPPA_FTLD_vs_lvPPA_AD | inter_word_pause_min | 0,003 | 0,73 | large |
| Pathotype | amnestic_AD_vs_lvPPA_AD | pause_median_duration | 0,003 | 0,55 | large |
| Pathotype | amnestic_AD_vs_lvPPA_AD | inter_word_pause_median | 0,003 | 0,55 | large |
| Pathotype | nfvPPA_FTLD_vs_lvPPA_AD | voyelles_oral_median | 0,003 | 0,82 | large |
| Pathotype | svPPA_FTLD_vs_lvPPA_AD | fricatives_kurtosis | 0,003 | 0,73 | large |
| Pathotype | svPPA_FTLD_vs_healthy_control | f3_cv | 0,003 | 0,59 | large |
| Pathotype | lvPPA_FTLD_vs_lvPPA_AD | fricatives_cv | 0,003 | 0,82 | large |
| Pathotype | FTLD_vs_lvPPA_FTLD | fricatives_cv | 0,003 | 0,77 | large |
| Pathotype | nfvPPA_FTLD_vs_lvPPA_FTLD | semi_voyelles_min | 0,003 | 0,96 | large |
| Pathotype | nfvPPA_FTLD_vs_lvPPA_FTLD | semi_voyelles_percentile_10 | 0,003 | 0,96 | large |
| Pathotype | lvPPA_FTLD_vs_healthy_control | delta_mfcc_mean_1 | 0,003 | 0,69 | large |
| Pathotype | nfvPPA_FTLD_vs_healthy_control | delta_mfcc_mean_12 | 0,003 | 0,69 | large |
| Pathotype | lvPPA_FTLD_vs_amnestic_AD | pause_min_duration | 0,003 | 0,71 | large |
| Pathotype | lvPPA_FTLD_vs_amnestic_AD | inter_word_pause_min | 0,003 | 0,71 | large |
| Pathotype | nfvPPA_FTLD_vs_FTLD | voyelles_oral_min | 0,003 | 0,71 | large |
| Pathotype | nfvPPA_FTLD_vs_lvPPA_AD | liquides_count | 0,003 | 0,81 | large |
| Pathotype | svPPA_FTLD_vs_amnestic_AD | pause_max_duration | 0,003 | 0,62 | large |
| Pathotype | svPPA_FTLD_vs_amnestic_AD | inter_word_pause_max | 0,003 | 0,62 | large |
| Pathotype | nfvPPA_FTLD_vs_lvPPA_FTLD | std_phoneme_duration | 0,003 | 0,96 | large |
| Pathotype | nfvPPA_FTLD_vs_lvPPA_FTLD | voyelles_oral_median | 0,003 | 0,96 | large |
| Pathotype | lvPPA_FTLD_vs_amnestic_AD | f1 | 0,003 | 0,71 | large |
| Pathotype | nfvPPA_FTLD_vs_amnestic_AD | jitter_rap | 0,003 | 0,71 | large |
| Pathotype | FTLD_vs_amnestic_AD | nasales_cv | 0,003 | 0,47 | medium |
| Pathotype | svPPA_FTLD_vs_healthy_control | nasales_std | 0,003 | 0,58 | large |
| Pathotype | svPPA_FTLD_vs_amnestic_AD | semi_voyelles_kurtosis | 0,003 | 0,62 | large |
| Pathotype | lvPPA_FTLD_vs_amnestic_AD | pause_median_duration | 0,003 | 0,71 | large |
| Pathotype | lvPPA_FTLD_vs_amnestic_AD | inter_word_pause_median | 0,003 | 0,71 | large |
| Pathotype | nfvPPA_FTLD_vs_amnestic_AD | contrast_mean_4 | 0,003 | 0,71 | large |
| Pathotype | svPPA_FTLD_vs_nfvPPA_FTLD | pause_frequency | 0,003 | 0,87 | large |
| Pathotype | healthy_control_vs_amnestic_AD | semi_voyelles_std | 0,003 | 0,36 | medium |
| Pathotype | nfvPPA_FTLD_vs_healthy_control | zcr_mean | 0,003 | 0,68 | large |
| Pathotype | svPPA_FTLD_vs_nfvPPA_FTLD | pause_ratio | 0,003 | 0,87 | large |
| Pathotype | FTLD_vs_lvPPA_AD | intensity_std | 0,003 | 0,60 | large |
| Pathotype | svPPA_FTLD_vs_amnestic_AD | fricatives_kurtosis | 0,003 | 0,62 | large |
| Pathotype | nfvPPA_FTLD_vs_FTLD | std_phoneme_duration | 0,003 | 0,76 | large |
| Pathotype | healthy_control_vs_lvPPA_AD | fricatives_iqr | 0,003 | 0,50 | large |
| Pathotype | svPPA_FTLD_vs_amnestic_AD | semi_voyelles_skewness | 0,003 | 0,61 | large |
| Pathotype | nfvPPA_FTLD_vs_amnestic_AD | liquides_count | 0,003 | 0,71 | large |
| Pathotype | healthy_control_vs_amnestic_AD | liquides_count | 0,003 | 0,35 | medium |
| Pathotype | healthy_control_vs_amnestic_AD | occlusives_min | 0,003 | 0,34 | medium |
| Pathotype | svPPA_FTLD_vs_amnestic_AD | std_phoneme_duration | 0,003 | 0,61 | large |
| Pathotype | FTLD_vs_lvPPA_FTLD | spectral_centroid_slope | 0,003 | 0,76 | large |
| Pathotype | FTLD_vs_lvPPA_FTLD | fricatives_iqr | 0,003 | 0,76 | large |
| Pathotype | FTLD_vs_lvPPA_FTLD | voyelles_oral_std | 0,003 | 0,76 | large |
| Pathotype | healthy_control_vs_lvPPA_AD | nasales_iqr | 0,003 | 0,50 | large |
| Pathotype | FTLD_vs_healthy_control | occlusives_percentile_10 | 0,004 | 0,41 | medium |
| Pathotype | svPPA_FTLD_vs_lvPPA_AD | liquides_skewness | 0,004 | 0,71 | large |
| Pathotype | svPPA_FTLD_vs_lvPPA_AD | chroma_mean_11 | 0,004 | 0,71 | large |
| Pathotype | FTLD_vs_lvPPA_FTLD | pause_min_duration | 0,004 | 0,74 | large |
| Pathotype | FTLD_vs_lvPPA_FTLD | inter_word_pause_min | 0,004 | 0,74 | large |
| Pathotype | FTLD_vs_lvPPA_FTLD | f0_min | 0,004 | 0,65 | large |
| Pathotype | svPPA_FTLD_vs_lvPPA_FTLD | f2_cv,1 | 0,004 | 0,86 | large |
| Pathotype | lvPPA_FTLD_vs_lvPPA_AD | kurt_phoneme_duration | 0,004 | 0,80 | large |
| Pathotype | lvPPA_FTLD_vs_lvPPA_AD | liquides_max | 0,004 | 0,80 | large |
| Pathotype | lvPPA_FTLD_vs_lvPPA_AD | liquides_percentile_90 | 0,004 | 0,80 | large |
| Pathotype | lvPPA_FTLD_vs_lvPPA_AD | skew_phoneme_duration | 0,004 | 0,80 | large |
| Pathotype | lvPPA_FTLD_vs_lvPPA_AD | voyelles_oral_median | 0,004 | 0,80 | large |
| Pathotype | svPPA_FTLD_vs_healthy_control | occlusives_count | 0,004 | 0,57 | large |
| Pathotype | nfvPPA_FTLD_vs_FTLD | fricatives_iqr | 0,004 | 0,75 | large |
| Pathotype | svPPA_FTLD_vs_FTLD | speech_duration | 0,004 | 0,66 | large |
| Pathotype | svPPA_FTLD_vs_FTLD | fricatives_skewness | 0,004 | 0,66 | large |
| Pathotype | lvPPA_FTLD_vs_healthy_control | occlusives_mean | 0,004 | 0,67 | large |
| Pathotype | lvPPA_FTLD_vs_healthy_control | zcr_mean | 0,004 | 0,67 | large |
| Pathotype | lvPPA_FTLD_vs_amnestic_AD | pause_proportion | 0,004 | 0,70 | large |
| Pathotype | svPPA_FTLD_vs_nfvPPA_FTLD | chroma_mean_1 | 0,004 | 0,86 | large |
| Pathotype | lvPPA_FTLD_vs_lvPPA_AD | chroma_mean_4 | 0,004 | 0,80 | large |
| Pathotype | FTLD_vs_healthy_control | nasales_percentile_90 | 0,004 | 0,42 | medium |
| Pathotype | FTLD_vs_healthy_control | pause_max_duration | 0,004 | 0,42 | medium |
| Pathotype | FTLD_vs_healthy_control | inter_word_pause_max | 0,004 | 0,42 | medium |
| Pathotype | lvPPA_FTLD_vs_amnestic_AD | f0_min | 0,004 | 0,62 | large |
| Pathotype | FTLD_vs_healthy_control | chroma_mean_7 | 0,004 | 0,42 | medium |
| Pathotype | svPPA_FTLD_vs_lvPPA_FTLD | kurt_phoneme_duration | 0,004 | 0,86 | large |
| Pathotype | svPPA_FTLD_vs_lvPPA_FTLD | pause_min_duration | 0,004 | 0,86 | large |
| Pathotype | svPPA_FTLD_vs_lvPPA_FTLD | inter_word_pause_min | 0,004 | 0,86 | large |
| Pathotype | amnestic_AD_vs_lvPPA_AD | voyelles_oral_min | 0,004 | 0,50 | large |
| Pathotype | nfvPPA_FTLD_vs_lvPPA_AD | spectral_flux_std | 0,004 | 0,80 | large |
| Pathotype | nfvPPA_FTLD_vs_lvPPA_AD | f0_mean | 0,004 | 0,80 | large |
| Pathotype | nfvPPA_FTLD_vs_amnestic_AD | semi_voyelles_max | 0,004 | 0,70 | large |
| Pathotype | lvPPA_FTLD_vs_amnestic_AD | nasales_mean | 0,004 | 0,70 | large |
| Pathotype | nfvPPA_FTLD_vs_amnestic_AD | chroma_mean_2 | 0,004 | 0,70 | large |
| Pathotype | svPPA_FTLD_vs_lvPPA_FTLD | skew_phoneme_duration | 0,004 | 0,86 | large |
| Pathotype | FTLD_vs_lvPPA_AD | pause_total_duration | 0,004 | 0,59 | large |
| Pathotype | svPPA_FTLD_vs_healthy_control | chroma_mean_5 | 0,004 | 0,57 | large |
| Pathotype | svPPA_FTLD_vs_amnestic_AD | pause_proportion | 0,004 | 0,60 | large |
| Pathotype | healthy_control_vs_amnestic_AD | intensity_slope | 0,004 | 0,35 | medium |
| Pathotype | FTLD_vs_amnestic_AD | delta_mfcc_mean_5 | 0,004 | 0,46 | medium |
| Pathotype | FTLD_vs_lvPPA_FTLD | fricatives_percentile_90 | 0,004 | 0,74 | large |
| Pathotype | svPPA_FTLD_vs_amnestic_AD | f3_cv,1 | 0,004 | 0,60 | large |
| Pathotype | FTLD_vs_lvPPA_FTLD | f3 | 0,004 | 0,74 | large |
| Pathotype | nfvPPA_FTLD_vs_FTLD | nasales_count | 0,004 | 0,73 | large |
| Pathotype | FTLD_vs_lvPPA_FTLD | pause_total_duration | 0,004 | 0,74 | large |
| Pathotype | FTLD_vs_lvPPA_FTLD | pause_ratio | 0,004 | 0,74 | large |
| Pathotype | FTLD_vs_lvPPA_AD | nasales_percentile_10 | 0,004 | 0,58 | large |
| Pathotype | svPPA_FTLD_vs_healthy_control | b3 | 0,004 | 0,57 | large |
| Pathotype | FTLD_vs_healthy_control | b1 | 0,004 | 0,42 | medium |
| Pathotype | healthy_control_vs_amnestic_AD | chroma_mean_2 | 0,004 | 0,34 | medium |
| Pathotype | nfvPPA_FTLD_vs_healthy_control | nasales_mean | 0,004 | 0,66 | large |
| Pathotype | lvPPA_FTLD_vs_healthy_control | pause_max_duration | 0,004 | 0,66 | large |
| Pathotype | lvPPA_FTLD_vs_healthy_control | inter_word_pause_max | 0,004 | 0,66 | large |
| Pathotype | svPPA_FTLD_vs_lvPPA_AD | pause_ratio | 0,004 | 0,70 | large |
| Pathotype | svPPA_FTLD_vs_lvPPA_AD | jitter_local | 0,004 | 0,70 | large |
| Pathotype | svPPA_FTLD_vs_lvPPA_AD | jitter_rap | 0,004 | 0,70 | large |
| Pathotype | nfvPPA_FTLD_vs_amnestic_AD | rate_speech_phonemes | 0,004 | 0,69 | large |
| Pathotype | nfvPPA_FTLD_vs_amnestic_AD | jitter_ppq5 | 0,004 | 0,69 | large |
| Pathotype | nfvPPA_FTLD_vs_amnestic_AD | chroma_mean_3 | 0,004 | 0,69 | large |
| Pathotype | nfvPPA_FTLD_vs_amnestic_AD | contrast_mean_1 | 0,004 | 0,69 | large |
| Pathotype | svPPA_FTLD_vs_nfvPPA_FTLD | liquides_cv | 0,004 | 0,84 | large |
| Pathotype | svPPA_FTLD_vs_nfvPPA_FTLD | nasales_count | 0,004 | 0,84 | large |
| Pathotype | healthy_control_vs_amnestic_AD | pause_min_duration | 0,004 | 0,34 | medium |
| Pathotype | healthy_control_vs_amnestic_AD | inter_word_pause_min | 0,004 | 0,34 | medium |
| Pathotype | svPPA_FTLD_vs_nfvPPA_FTLD | pause_proportion | 0,004 | 0,84 | large |
| Pathotype | FTLD_vs_healthy_control | shimmer_local | 0,004 | 0,42 | medium |
| Pathotype | svPPA_FTLD_vs_lvPPA_AD | jitter_ppq5 | 0,004 | 0,70 | large |
| Pathotype | FTLD_vs_lvPPA_AD | f2_cv,1 | 0,004 | 0,58 | large |
| Pathotype | svPPA_FTLD_vs_FTLD | liquides_median | 0,004 | 0,64 | large |
| Pathotype | nfvPPA_FTLD_vs_lvPPA_FTLD | contrast_mean_1 | 0,004 | 0,92 | large |
| Pathotype | nfvPPA_FTLD_vs_lvPPA_FTLD | spectral_centroid_std | 0,004 | 0,92 | large |
| Pathotype | nfvPPA_FTLD_vs_lvPPA_FTLD | f1_cv,1 | 0,004 | 0,92 | large |
| Pathotype | nfvPPA_FTLD_vs_FTLD | semi_voyelles_mean | 0,004 | 0,73 | large |
| Pathotype | FTLD_vs_amnestic_AD | rolloff_mean | 0,004 | 0,46 | medium |
| Pathotype | svPPA_FTLD_vs_amnestic_AD | pause_cv | 0,004 | 0,59 | large |
| Pathotype | FTLD_vs_amnestic_AD | chroma_mean_2 | 0,004 | 0,46 | medium |
| Pathotype | healthy_control_vs_amnestic_AD | pause_total_duration | 0,005 | 0,34 | medium |
| Pathotype | nfvPPA_FTLD_vs_lvPPA_AD | pause_frequency | 0,005 | 0,78 | large |
| Pathotype | lvPPA_FTLD_vs_lvPPA_AD | fricatives_max | 0,005 | 0,78 | large |
| Pathotype | nfvPPA_FTLD_vs_amnestic_AD | occlusives_kurtosis | 0,005 | 0,68 | large |
| Pathotype | lvPPA_FTLD_vs_amnestic_AD | pause_std_duration | 0,005 | 0,68 | large |
| Pathotype | lvPPA_FTLD_vs_amnestic_AD | inter_word_pause_mean | 0,005 | 0,68 | large |
| Pathotype | lvPPA_FTLD_vs_amnestic_AD | inter_word_pause_std | 0,005 | 0,68 | large |
| Pathotype | lvPPA_FTLD_vs_lvPPA_AD | f1_cv | 0,005 | 0,78 | large |
| Pathotype | FTLD_vs_lvPPA_FTLD | contrast_mean_2 | 0,005 | 0,73 | large |
| Pathotype | FTLD_vs_lvPPA_FTLD | spectral_centroid_std | 0,005 | 0,73 | large |
| Pathotype | FTLD_vs_lvPPA_AD | spectral_centroid_slope | 0,005 | 0,57 | large |
| Pathotype | nfvPPA_FTLD_vs_amnestic_AD | fricatives_count | 0,005 | 0,68 | large |
| Pathotype | FTLD_vs_lvPPA_FTLD | pause_proportion | 0,005 | 0,73 | large |
| Pathotype | FTLD_vs_lvPPA_FTLD | voyelles_oral_mean | 0,005 | 0,73 | large |
| Pathotype | FTLD_vs_lvPPA_FTLD | fricatives_mean | 0,005 | 0,73 | large |
| Pathotype | svPPA_FTLD_vs_nfvPPA_FTLD | nasales_percentile_10 | 0,005 | 0,81 | large |
| Pathotype | FTLD_vs_lvPPA_FTLD | cv_phoneme_duration | 0,005 | 0,73 | large |
| Pathotype | svPPA_FTLD_vs_FTLD | delta_mfcc_mean_4 | 0,005 | 0,64 | large |
| Pathotype | svPPA_FTLD_vs_FTLD | chroma_mean_1 | 0,005 | 0,64 | large |
| Pathotype | svPPA_FTLD_vs_lvPPA_FTLD | contrast_mean_2 | 0,005 | 0,83 | large |
| Pathotype | svPPA_FTLD_vs_nfvPPA_FTLD | spectral_instability | 0,005 | 0,83 | large |
| Pathotype | svPPA_FTLD_vs_nfvPPA_FTLD | f1_cv | 0,005 | 0,83 | large |
| Pathotype | svPPA_FTLD_vs_nfvPPA_FTLD | delta_mfcc_mean_2 | 0,005 | 0,83 | large |
| Pathotype | svPPA_FTLD_vs_nfvPPA_FTLD | chroma_mean_11 | 0,005 | 0,83 | large |
| Pathotype | svPPA_FTLD_vs_nfvPPA_FTLD | spectral_instability,1 | 0,005 | 0,83 | large |
| Pathotype | svPPA_FTLD_vs_nfvPPA_FTLD | f1_cv,1 | 0,005 | 0,83 | large |
| Pathotype | svPPA_FTLD_vs_nfvPPA_FTLD | pause_median_duration | 0,005 | 0,83 | large |
| Pathotype | svPPA_FTLD_vs_nfvPPA_FTLD | inter_word_pause_median | 0,005 | 0,83 | large |
| Pathotype | svPPA_FTLD_vs_nfvPPA_FTLD | semi_voyelles_mean | 0,005 | 0,83 | large |
| Pathotype | svPPA_FTLD_vs_nfvPPA_FTLD | semi_voyelles_percentile_90 | 0,005 | 0,83 | large |
| Pathotype | healthy_control_vs_amnestic_AD | occlusives_median | 0,005 | 0,33 | medium |
| Pathotype | svPPA_FTLD_vs_lvPPA_AD | mean_phoneme_duration | 0,005 | 0,69 | large |
| Pathotype | svPPA_FTLD_vs_lvPPA_AD | speech_rate_phonemes_per_sec | 0,005 | 0,69 | large |
| Pathotype | svPPA_FTLD_vs_lvPPA_AD | fricatives_std | 0,005 | 0,69 | large |
| Pathotype | svPPA_FTLD_vs_lvPPA_FTLD | spectral_centroid_slope | 0,005 | 0,83 | large |
| Pathotype | nfvPPA_FTLD_vs_amnestic_AD | liquides_kurtosis | 0,005 | 0,67 | large |
| Pathotype | svPPA_FTLD_vs_nfvPPA_FTLD | chroma_mean_0 | 0,005 | 0,83 | large |
| Pathotype | svPPA_FTLD_vs_nfvPPA_FTLD | intensity_std | 0,005 | 0,83 | large |
| Pathotype | FTLD_vs_amnestic_AD | chroma_mean_5 | 0,005 | 0,45 | medium |
| Pathotype | svPPA_FTLD_vs_amnestic_AD | max_phoneme_duration | 0,005 | 0,58 | large |
| Pathotype | svPPA_FTLD_vs_amnestic_AD | shimmer_apq3 | 0,005 | 0,58 | large |
| Pathotype | svPPA_FTLD_vs_amnestic_AD | shimmer_dda | 0,005 | 0,58 | large |
| Pathotype | svPPA_FTLD_vs_healthy_control | nasales_percentile_90 | 0,005 | 0,55 | large |
| Pathotype | svPPA_FTLD_vs_healthy_control | pause_frequency | 0,005 | 0,55 | large |
| Pathotype | svPPA_FTLD_vs_lvPPA_FTLD | voyelles_oral_max | 0,005 | 0,83 | large |
| Pathotype | nfvPPA_FTLD_vs_FTLD | syllable_rate_per_sec | 0,005 | 0,72 | large |
| Pathotype | nfvPPA_FTLD_vs_amnestic_AD | syllable_rate_per_sec | 0,005 | 0,67 | large |
| Pathotype | nfvPPA_FTLD_vs_amnestic_AD | occlusives_std | 0,005 | 0,67 | large |
| Pathotype | lvPPA_FTLD_vs_amnestic_AD | pause_frequency | 0,005 | 0,67 | large |
| Pathotype | svPPA_FTLD_vs_lvPPA_FTLD | fricatives_mean | 0,005 | 0,83 | large |
| Pathotype | FTLD_vs_lvPPA_FTLD | semi_voyelles_min | 0,005 | 0,62 | large |
| Pathotype | svPPA_FTLD_vs_lvPPA_FTLD | nasales_kurtosis | 0,005 | 0,80 | large |
| Pathotype | nfvPPA_FTLD_vs_amnestic_AD | median_phoneme_duration | 0,005 | 0,66 | large |
| Pathotype | FTLD_vs_healthy_control | jitter_ppq5 | 0,005 | 0,41 | medium |
| Pathotype | FTLD_vs_lvPPA_AD | f2_cv | 0,005 | 0,56 | large |
| Pathotype | healthy_control_vs_amnestic_AD | voyelles_oral_median | 0,005 | 0,32 | medium |
| Pathotype | nfvPPA_FTLD_vs_healthy_control | voyelles_oral_percentile_10 | 0,005 | 0,59 | large |
| Pathotype | FTLD_vs_lvPPA_AD | semi_voyelles_count | 0,006 | 0,54 | large |
| Pathotype | FTLD_vs_lvPPA_FTLD | nasales_median | 0,006 | 0,71 | large |
| Pathotype | svPPA_FTLD_vs_amnestic_AD | liquides_skewness | 0,006 | 0,58 | large |
| Pathotype | lvPPA_FTLD_vs_amnestic_AD | liquides_kurtosis | 0,006 | 0,67 | large |
| Pathotype | healthy_control_vs_lvPPA_AD | contrast_mean_5 | 0,006 | 0,47 | medium |
| Pathotype | FTLD_vs_lvPPA_FTLD | semi_voyelles_percentile_10 | 0,006 | 0,65 | large |
| Pathotype | lvPPA_FTLD_vs_lvPPA_AD | semi_voyelles_count | 0,006 | 0,76 | large |
| Pathotype | FTLD_vs_lvPPA_FTLD | pause_median_duration | 0,006 | 0,71 | large |
| Pathotype | FTLD_vs_lvPPA_FTLD | inter_word_pause_median | 0,006 | 0,71 | large |
| Pathotype | svPPA_FTLD_vs_nfvPPA_FTLD | voyelles_oral_min | 0,006 | 0,80 | large |
| Pathotype | lvPPA_FTLD_vs_healthy_control | delta_mfcc_mean_11 | 0,006 | 0,64 | large |
| Pathotype | FTLD_vs_lvPPA_FTLD | chroma_mean_0 | 0,006 | 0,71 | large |
| Pathotype | lvPPA_FTLD_vs_amnestic_AD | b2 | 0,006 | 0,67 | large |
| Pathotype | FTLD_vs_lvPPA_FTLD | occlusives_skewness | 0,006 | 0,71 | large |
| Pathotype | amnestic_AD_vs_lvPPA_AD | fricatives_kurtosis | 0,006 | 0,51 | large |
| Pathotype | FTLD_vs_lvPPA_FTLD | pause_mean_duration | 0,006 | 0,71 | large |
| Pathotype | FTLD_vs_lvPPA_FTLD | inter_word_pause_mean | 0,006 | 0,71 | large |
| Pathotype | svPPA_FTLD_vs_healthy_control | pause_proportion | 0,006 | 0,55 | large |
| Pathotype | svPPA_FTLD_vs_healthy_control | pause_ratio | 0,006 | 0,55 | large |
| Pathotype | nfvPPA_FTLD_vs_amnestic_AD | occlusives_mean | 0,006 | 0,67 | large |
| Pathotype | lvPPA_FTLD_vs_lvPPA_AD | semi_voyelles_percentile_90 | 0,006 | 0,76 | large |
| Pathotype | svPPA_FTLD_vs_nfvPPA_FTLD | fricatives_std | 0,006 | 0,81 | large |
| Pathotype | svPPA_FTLD_vs_lvPPA_AD | voyelles_oral_count | 0,006 | 0,67 | large |
| Pathotype | nfvPPA_FTLD_vs_lvPPA_AD | syllable_rate_per_sec | 0,006 | 0,76 | large |
| Pathotype | lvPPA_FTLD_vs_lvPPA_AD | occlusives_mean | 0,006 | 0,76 | large |
| Pathotype | nfvPPA_FTLD_vs_amnestic_AD | liquides_percentile_10 | 0,006 | 0,65 | large |
| Pathotype | lvPPA_FTLD_vs_lvPPA_AD | pause_frequency | 0,006 | 0,76 | large |
| Pathotype | lvPPA_FTLD_vs_lvPPA_AD | pause_ratio | 0,006 | 0,76 | large |
| Pathotype | lvPPA_FTLD_vs_lvPPA_AD | long_pause_count | 0,006 | 0,73 | large |
| Pathotype | lvPPA_FTLD_vs_amnestic_AD | semi_voyelles_min | 0,006 | 0,65 | large |
| Pathotype | lvPPA_FTLD_vs_healthy_control | nasales_mean | 0,006 | 0,64 | large |
| Pathotype | nfvPPA_FTLD_vs_healthy_control | chroma_mean_3 | 0,006 | 0,64 | large |
| Pathotype | nfvPPA_FTLD_vs_healthy_control | articulation_rate | 0,006 | 0,63 | large |
| Pathotype | lvPPA_FTLD_vs_amnestic_AD | occlusives_min | 0,006 | 0,65 | large |
| Pathotype | svPPA_FTLD_vs_healthy_control | occlusives_std | 0,006 | 0,54 | large |
| Pathotype | svPPA_FTLD_vs_healthy_control | chroma_mean_6 | 0,006 | 0,54 | large |
| Pathotype | lvPPA_FTLD_vs_lvPPA_AD | shimmer_apq3 | 0,006 | 0,76 | large |
| Pathotype | lvPPA_FTLD_vs_lvPPA_AD | shimmer_dda | 0,006 | 0,76 | large |
| Pathotype | lvPPA_FTLD_vs_lvPPA_AD | contrast_mean_0 | 0,006 | 0,76 | large |
| Pathotype | lvPPA_FTLD_vs_lvPPA_AD | intensity_slope | 0,006 | 0,76 | large |
| Pathotype | lvPPA_FTLD_vs_lvPPA_AD | spectral_instability,1 | 0,006 | 0,76 | large |
| Pathotype | FTLD_vs_amnestic_AD | spectral_centroid_slope | 0,006 | 0,44 | medium |
| Pathotype | svPPA_FTLD_vs_lvPPA_AD | occlusives_kurtosis | 0,006 | 0,67 | large |
| Pathotype | lvPPA_FTLD_vs_amnestic_AD | pause_max_duration | 0,006 | 0,66 | large |
| Pathotype | lvPPA_FTLD_vs_amnestic_AD | inter_word_pause_max | 0,006 | 0,66 | large |
| Pathotype | svPPA_FTLD_vs_lvPPA_AD | std_phoneme_duration | 0,006 | 0,67 | large |
| Pathotype | nfvPPA_FTLD_vs_FTLD | semi_voyelles_std | 0,006 | 0,71 | large |
| Pathotype | nfvPPA_FTLD_vs_lvPPA_AD | hnr_std | 0,006 | 0,76 | large |
| Pathotype | nfvPPA_FTLD_vs_lvPPA_AD | hnr_std,1 | 0,006 | 0,76 | large |
| Pathotype | svPPA_FTLD_vs_lvPPA_AD | intensity_slope | 0,006 | 0,67 | large |
| Pathotype | svPPA_FTLD_vs_amnestic_AD | chroma_mean_2 | 0,006 | 0,57 | large |
| Pathotype | nfvPPA_FTLD_vs_FTLD | semi_voyelles_percentile_90 | 0,006 | 0,71 | large |
| Pathotype | FTLD_vs_amnestic_AD | nasales_median | 0,006 | 0,44 | medium |
| Pathotype | FTLD_vs_amnestic_AD | occlusives_percentile_90 | 0,006 | 0,44 | medium |
| Pathotype | svPPA_FTLD_vs_FTLD | fricatives_count | 0,006 | 0,62 | large |
| Pathotype | svPPA_FTLD_vs_lvPPA_AD | chroma_mean_7 | 0,006 | 0,67 | large |
| Pathotype | svPPA_FTLD_vs_lvPPA_AD | f0_mean | 0,006 | 0,67 | large |
| Pathotype | svPPA_FTLD_vs_healthy_control | nasales_skewness | 0,006 | 0,54 | large |
| Pathotype | svPPA_FTLD_vs_healthy_control | fricatives_skewness | 0,006 | 0,54 | large |
| Pathotype | svPPA_FTLD_vs_FTLD | intensity_std | 0,006 | 0,62 | large |
| Pathotype | FTLD_vs_amnestic_AD | nasales_skewness | 0,006 | 0,44 | medium |
| Pathotype | lvPPA_FTLD_vs_lvPPA_AD | nasales_count | 0,006 | 0,74 | large |
| Pathotype | nfvPPA_FTLD_vs_amnestic_AD | semi_voyelles_cv | 0,006 | 0,66 | large |
| Pathotype | nfvPPA_FTLD_vs_healthy_control | nasales_min | 0,007 | 0,62 | large |
| Pathotype | healthy_control_vs_lvPPA_AD | chroma_mean_4 | 0,007 | 0,47 | medium |
| Pathotype | nfvPPA_FTLD_vs_lvPPA_FTLD | intensity_dynamic_range | 0,007 | 0,88 | large |
| Pathotype | svPPA_FTLD_vs_nfvPPA_FTLD | occlusives_median | 0,007 | 0,80 | large |
| Pathotype | FTLD_vs_lvPPA_AD | contrast_mean_4 | 0,007 | 0,55 | large |
| Pathotype | nfvPPA_FTLD_vs_lvPPA_FTLD | intensity_std | 0,007 | 0,88 | large |
| Pathotype | nfvPPA_FTLD_vs_lvPPA_FTLD | occlusives_min | 0,007 | 0,88 | large |
| Pathotype | svPPA_FTLD_vs_lvPPA_FTLD | f1_cv | 0,007 | 0,80 | large |
| Pathotype | FTLD_vs_amnestic_AD | delta_mfcc_mean_6 | 0,007 | 0,43 | medium |
| Pathotype | svPPA_FTLD_vs_nfvPPA_FTLD | jitter_local | 0,007 | 0,80 | large |
| Pathotype | nfvPPA_FTLD_vs_FTLD | jitter_ppq5 | 0,007 | 0,70 | large |
| Pathotype | FTLD_vs_lvPPA_FTLD | spectral_instability,1 | 0,007 | 0,70 | large |
| Pathotype | svPPA_FTLD_vs_amnestic_AD | chroma_mean_5 | 0,007 | 0,57 | large |
| Pathotype | FTLD_vs_lvPPA_FTLD | voyelles_oral_percentile_90 | 0,007 | 0,70 | large |
| Pathotype | healthy_control_vs_amnestic_AD | syllable_count | 0,007 | 0,33 | medium |
| Pathotype | nfvPPA_FTLD_vs_healthy_control | nasales_std | 0,007 | 0,63 | large |
| Pathotype | nfvPPA_FTLD_vs_healthy_control | rolloff_std | 0,007 | 0,63 | large |
| Pathotype | nfvPPA_FTLD_vs_lvPPA_FTLD | f0_mean | 0,007 | 0,88 | large |
| Pathotype | nfvPPA_FTLD_vs_lvPPA_FTLD | voyelles_oral_max | 0,007 | 0,88 | large |
| Pathotype | nfvPPA_FTLD_vs_lvPPA_FTLD | voyelles_oral_percentile_10 | 0,007 | 0,88 | large |
| Pathotype | nfvPPA_FTLD_vs_lvPPA_FTLD | fricatives_std | 0,007 | 0,88 | large |
| Pathotype | nfvPPA_FTLD_vs_lvPPA_FTLD | fricatives_max | 0,007 | 0,88 | large |
| Pathotype | nfvPPA_FTLD_vs_lvPPA_FTLD | fricatives_cv | 0,007 | 0,88 | large |
| Pathotype | healthy_control_vs_amnestic_AD | semi_voyelles_max | 0,007 | 0,32 | medium |
| Pathotype | amnestic_AD_vs_lvPPA_AD | delta_mfcc_mean_1 | 0,007 | 0,50 | medium |
| Pathotype | svPPA_FTLD_vs_nfvPPA_FTLD | mean_phoneme_duration | 0,007 | 0,80 | large |
| Pathotype | svPPA_FTLD_vs_nfvPPA_FTLD | std_phoneme_duration | 0,007 | 0,80 | large |
| Pathotype | svPPA_FTLD_vs_nfvPPA_FTLD | speech_rate_phonemes_per_sec | 0,007 | 0,80 | large |
| Pathotype | svPPA_FTLD_vs_nfvPPA_FTLD | rate_speech_phonemes | 0,007 | 0,80 | large |
| Pathotype | svPPA_FTLD_vs_lvPPA_AD | median_phoneme_duration | 0,007 | 0,66 | large |
| Pathotype | svPPA_FTLD_vs_healthy_control | delta_mfcc_mean_2 | 0,007 | 0,53 | large |
| Pathotype | svPPA_FTLD_vs_healthy_control | spectral_flux_std | 0,007 | 0,53 | large |
| Pathotype | svPPA_FTLD_vs_nfvPPA_FTLD | delta_mfcc_mean_3 | 0,007 | 0,80 | large |
| Pathotype | amnestic_AD_vs_lvPPA_AD | contrast_mean_3 | 0,007 | 0,50 | medium |
| Pathotype | nfvPPA_FTLD_vs_lvPPA_FTLD | cv_phoneme_duration | 0,007 | 0,88 | large |
| Pathotype | FTLD_vs_healthy_control | spectral_flux,1 | 0,007 | 0,39 | medium |
| Pathotype | svPPA_FTLD_vs_lvPPA_FTLD | pause_frequency | 0,007 | 0,80 | large |
| Pathotype | svPPA_FTLD_vs_lvPPA_FTLD | fricatives_median | 0,007 | 0,80 | large |
| Pathotype | svPPA_FTLD_vs_lvPPA_FTLD | voyelles_oral_cv | 0,007 | 0,80 | large |
| Pathotype | nfvPPA_FTLD_vs_amnestic_AD | occlusives_skewness | 0,007 | 0,65 | large |
| Pathotype | svPPA_FTLD_vs_healthy_control | syllable_count | 0,007 | 0,53 | large |
| Pathotype | svPPA_FTLD_vs_healthy_control | voyelles_oral_count | 0,007 | 0,53 | large |
| Pathotype | FTLD_vs_healthy_control | spectral_flux | 0,007 | 0,39 | medium |
| Pathotype | svPPA_FTLD_vs_amnestic_AD | f3_cv | 0,007 | 0,56 | large |
| Pathotype | nfvPPA_FTLD_vs_FTLD | voyelles_oral_cv | 0,007 | 0,69 | large |
| Pathotype | nfvPPA_FTLD_vs_amnestic_AD | voyelles_oral_median | 0,007 | 0,64 | large |
| Pathotype | svPPA_FTLD_vs_healthy_control | kurt_phoneme_duration | 0,007 | 0,53 | large |
| Pathotype | svPPA_FTLD_vs_lvPPA_AD | voyelles_oral_kurtosis | 0,007 | 0,66 | large |
| Pathotype | FTLD_vs_amnestic_AD | nasales_kurtosis | 0,007 | 0,43 | medium |
| Pathotype | FTLD_vs_amnestic_AD | f0_mean | 0,007 | 0,43 | medium |
| Pathotype | svPPA_FTLD_vs_amnestic_AD | num_final_pauses | 0,007 | 0,20 | small |
| Pathotype | lvPPA_FTLD_vs_lvPPA_AD | occlusives_skewness | 0,007 | 0,73 | large |
| Pathotype | healthy_control_vs_amnestic_AD | semi_voyelles_kurtosis | 0,008 | 0,32 | medium |
| Pathotype | svPPA_FTLD_vs_FTLD | voyelles_oral_iqr | 0,008 | 0,60 | large |
| Pathotype | svPPA_FTLD_vs_amnestic_AD | fricatives_median | 0,008 | 0,56 | large |
| Pathotype | nfvPPA_FTLD_vs_lvPPA_AD | intensity_std | 0,008 | 0,73 | large |
| Pathotype | healthy_control_vs_lvPPA_AD | occlusives_min | 0,008 | 0,44 | medium |
| Pathotype | nfvPPA_FTLD_vs_amnestic_AD | liquides_skewness | 0,008 | 0,64 | large |
| Pathotype | FTLD_vs_healthy_control | pause_proportion | 0,008 | 0,39 | medium |
| Pathotype | svPPA_FTLD_vs_nfvPPA_FTLD | hnr_std | 0,008 | 0,79 | large |
| Pathotype | svPPA_FTLD_vs_nfvPPA_FTLD | hnr_std,1 | 0,008 | 0,79 | large |
| Pathotype | svPPA_FTLD_vs_amnestic_AD | rolloff_std | 0,008 | 0,56 | large |
| Pathotype | nfvPPA_FTLD_vs_amnestic_AD | pause_std_duration | 0,008 | 0,64 | large |
| Pathotype | nfvPPA_FTLD_vs_amnestic_AD | inter_word_pause_std | 0,008 | 0,64 | large |
| Pathotype | svPPA_FTLD_vs_amnestic_AD | liquides_percentile_10 | 0,008 | 0,54 | large |
| Pathotype | amnestic_AD_vs_lvPPA_AD | inter_word_pause_mean | 0,008 | 0,49 | medium |
| Pathotype | healthy_control_vs_lvPPA_AD | chroma_mean_9 | 0,008 | 0,45 | medium |
| Pathotype | lvPPA_FTLD_vs_lvPPA_AD | occlusives_min | 0,008 | 0,72 | large |
| Pathotype | lvPPA_FTLD_vs_lvPPA_AD | occlusives_count | 0,008 | 0,71 | large |
| Pathotype | FTLD_vs_lvPPA_AD | cv_phoneme_duration | 0,008 | 0,54 | large |
| Pathotype | svPPA_FTLD_vs_FTLD | occlusives_count | 0,008 | 0,60 | large |
| Pathotype | FTLD_vs_lvPPA_AD | b3 | 0,008 | 0,54 | large |
| Pathotype | FTLD_vs_healthy_control | pause_ratio | 0,008 | 0,39 | medium |
| Pathotype | svPPA_FTLD_vs_FTLD | pause_frequency | 0,008 | 0,60 | large |
| Pathotype | svPPA_FTLD_vs_healthy_control | nasales_cv | 0,008 | 0,52 | large |
| Pathotype | svPPA_FTLD_vs_amnestic_AD | occlusives_cv | 0,008 | 0,55 | large |
| Pathotype | FTLD_vs_healthy_control | chroma_mean_3 | 0,008 | 0,38 | medium |
| Pathotype | nfvPPA_FTLD_vs_amnestic_AD | speech_rate_phonemes_per_sec | 0,009 | 0,63 | large |
| Pathotype | nfvPPA_FTLD_vs_FTLD | occlusives_kurtosis | 0,009 | 0,68 | large |
| Pathotype | lvPPA_FTLD_vs_amnestic_AD | pause_mean_duration | 0,009 | 0,63 | large |
| Pathotype | nfvPPA_FTLD_vs_amnestic_AD | total_duration | 0,009 | 0,63 | large |
| Pathotype | svPPA_FTLD_vs_lvPPA_AD | long_pause_count | 0,009 | 0,63 | large |
| Pathotype | svPPA_FTLD_vs_lvPPA_AD | occlusives_skewness | 0,009 | 0,64 | large |
| Pathotype | svPPA_FTLD_vs_healthy_control | fricatives_max | 0,009 | 0,52 | large |
| Pathotype | svPPA_FTLD_vs_healthy_control | f1_cv | 0,009 | 0,52 | large |
| Pathotype | svPPA_FTLD_vs_healthy_control | fricatives_kurtosis | 0,009 | 0,52 | large |
| Pathotype | nfvPPA_FTLD_vs_healthy_control | nasales_max | 0,009 | 0,61 | large |
| Pathotype | svPPA_FTLD_vs_nfvPPA_FTLD | phoneme_count | 0,009 | 0,77 | large |
| Pathotype | svPPA_FTLD_vs_nfvPPA_FTLD | num_speech_phonemes | 0,009 | 0,77 | large |
| Pathotype | amnestic_AD_vs_lvPPA_AD | pause_mean_duration | 0,009 | 0,48 | medium |
| Pathotype | healthy_control_vs_lvPPA_AD | num_inter_pauses | 0,009 | 0,45 | medium |
| Pathotype | svPPA_FTLD_vs_lvPPA_FTLD | delta_mfcc_mean_12 | 0,009 | 0,77 | large |
| Pathotype | nfvPPA_FTLD_vs_lvPPA_AD | num_pauses | 0,009 | 0,71 | large |
| Pathotype | nfvPPA_FTLD_vs_FTLD | semi_voyelles_median | 0,009 | 0,67 | large |
| Pathotype | svPPA_FTLD_vs_amnestic_AD | liquides_kurtosis | 0,009 | 0,54 | large |
| Pathotype | FTLD_vs_healthy_control | liquides_min | 0,009 | 0,37 | medium |
| Pathotype | FTLD_vs_lvPPA_FTLD | f1_cv | 0,009 | 0,67 | large |
| Pathotype | FTLD_vs_lvPPA_FTLD | delta_mfcc_mean_8 | 0,009 | 0,67 | large |
| Pathotype | FTLD_vs_lvPPA_FTLD | chroma_mean_7 | 0,009 | 0,67 | large |
| Pathotype | svPPA_FTLD_vs_nfvPPA_FTLD | semi_voyelles_iqr | 0,009 | 0,77 | large |
| Pathotype | svPPA_FTLD_vs_nfvPPA_FTLD | speech_duration | 0,009 | 0,77 | large |
| Pathotype | svPPA_FTLD_vs_nfvPPA_FTLD | nasales_kurtosis | 0,009 | 0,77 | large |
| Pathotype | lvPPA_FTLD_vs_lvPPA_AD | occlusives_max | 0,009 | 0,71 | large |
| Pathotype | svPPA_FTLD_vs_lvPPA_FTLD | chroma_mean_7 | 0,009 | 0,77 | large |
| Pathotype | nfvPPA_FTLD_vs_lvPPA_AD | occlusives_mean | 0,009 | 0,71 | large |
| Pathotype | nfvPPA_FTLD_vs_lvPPA_AD | occlusives_iqr | 0,009 | 0,71 | large |
| Pathotype | lvPPA_FTLD_vs_lvPPA_AD | occlusives_percentile_90 | 0,009 | 0,71 | large |
| Pathotype | svPPA_FTLD_vs_nfvPPA_FTLD | jitter_ppq5 | 0,009 | 0,77 | large |
| Pathotype | lvPPA_FTLD_vs_lvPPA_AD | max_phoneme_duration | 0,009 | 0,71 | large |
| Pathotype | lvPPA_FTLD_vs_lvPPA_AD | pause_median_duration | 0,009 | 0,71 | large |
| Pathotype | lvPPA_FTLD_vs_lvPPA_AD | pause_std_duration | 0,009 | 0,71 | large |
| Pathotype | lvPPA_FTLD_vs_lvPPA_AD | inter_word_pause_mean | 0,009 | 0,71 | large |
| Pathotype | lvPPA_FTLD_vs_lvPPA_AD | inter_word_pause_median | 0,009 | 0,71 | large |
| Pathotype | lvPPA_FTLD_vs_lvPPA_AD | inter_word_pause_std | 0,009 | 0,71 | large |
| Pathotype | lvPPA_FTLD_vs_lvPPA_AD | voyelles_oral_max | 0,009 | 0,71 | large |
| Pathotype | lvPPA_FTLD_vs_lvPPA_AD | fricatives_count | 0,009 | 0,71 | large |
| Pathotype | lvPPA_FTLD_vs_lvPPA_AD | nasales_mean | 0,009 | 0,71 | large |
| Pathotype | lvPPA_FTLD_vs_amnestic_AD | occlusives_cv | 0,009 | 0,63 | large |
| Pathotype | nfvPPA_FTLD_vs_healthy_control | occlusives_kurtosis | 0,009 | 0,60 | large |
| Pathotype | nfvPPA_FTLD_vs_healthy_control | liquides_kurtosis | 0,009 | 0,60 | large |
| Pathotype | nfvPPA_FTLD_vs_lvPPA_FTLD | min_phoneme_duration | 0,009 | 0,80 | large |
| Pathotype | lvPPA_FTLD_vs_healthy_control | delta_mfcc_mean_4 | 0,009 | 0,60 | large |
| Pathotype | healthy_control_vs_amnestic_AD | speech_duration | 0,009 | 0,31 | medium |
| Pathotype | healthy_control_vs_amnestic_AD | b1 | 0,009 | 0,31 | medium |
| Pathotype | svPPA_FTLD_vs_nfvPPA_FTLD | semi_voyelles_min | 0,009 | 0,74 | large |
| Pathotype | svPPA_FTLD_vs_nfvPPA_FTLD | semi_voyelles_percentile_10 | 0,009 | 0,74 | large |
| Pathotype | FTLD_vs_amnestic_AD | spectral_centroid_mean | 0,010 | 0,42 | medium |
| Pathotype | svPPA_FTLD_vs_lvPPA_FTLD | mean_phoneme_duration | 0,010 | 0,77 | large |
| Pathotype | svPPA_FTLD_vs_lvPPA_FTLD | std_phoneme_duration | 0,010 | 0,77 | large |
| Pathotype | svPPA_FTLD_vs_lvPPA_FTLD | cv_phoneme_duration | 0,010 | 0,77 | large |
| Pathotype | svPPA_FTLD_vs_lvPPA_FTLD | speech_rate_phonemes_per_sec | 0,010 | 0,77 | large |
| Pathotype | svPPA_FTLD_vs_lvPPA_FTLD | rate_speech_phonemes | 0,010 | 0,77 | large |
| Pathotype | svPPA_FTLD_vs_lvPPA_FTLD | fricatives_cv | 0,010 | 0,77 | large |
| Pathotype | lvPPA_FTLD_vs_lvPPA_AD | jitter_local | 0,010 | 0,71 | large |
| Pathotype | lvPPA_FTLD_vs_lvPPA_AD | jitter_rap | 0,010 | 0,71 | large |
| Pathotype | lvPPA_FTLD_vs_lvPPA_AD | jitter_ppq5 | 0,010 | 0,71 | large |
| Pathotype | lvPPA_FTLD_vs_lvPPA_AD | delta_mfcc_mean_12 | 0,010 | 0,71 | large |
| Pathotype | lvPPA_FTLD_vs_lvPPA_AD | spectral_centroid_std | 0,010 | 0,71 | large |
| Pathotype | lvPPA_FTLD_vs_lvPPA_AD | f0_mean | 0,010 | 0,71 | large |
| Pathotype | lvPPA_FTLD_vs_lvPPA_AD | hnr_mean,1 | 0,010 | 0,71 | large |
| Pathotype | nfvPPA_FTLD_vs_amnestic_AD | liquides_percentile_90 | 0,010 | 0,63 | large |
| Pathotype | nfvPPA_FTLD_vs_lvPPA_AD | jitter_local | 0,010 | 0,71 | large |
| Pathotype | nfvPPA_FTLD_vs_lvPPA_AD | jitter_rap | 0,010 | 0,71 | large |
| Pathotype | nfvPPA_FTLD_vs_lvPPA_AD | jitter_ppq5 | 0,010 | 0,71 | large |
| Pathotype | nfvPPA_FTLD_vs_lvPPA_AD | spectral_flux | 0,010 | 0,71 | large |
| Pathotype | nfvPPA_FTLD_vs_lvPPA_AD | spectral_flux,1 | 0,010 | 0,71 | large |
| Pathotype | lvPPA_FTLD_vs_lvPPA_AD | hnr_std | 0,010 | 0,71 | large |
| Pathotype | lvPPA_FTLD_vs_lvPPA_AD | delta_mfcc_mean_4 | 0,010 | 0,71 | large |
| Pathotype | lvPPA_FTLD_vs_amnestic_AD | chroma_mean_10 | 0,010 | 0,63 | large |
| Pathotype | svPPA_FTLD_vs_healthy_control | speech_duration | 0,010 | 0,51 | large |
| Pathotype | svPPA_FTLD_vs_healthy_control | skew_phoneme_duration | 0,010 | 0,51 | large |
| Pathotype | lvPPA_FTLD_vs_lvPPA_AD | fricatives_min | 0,010 | 0,69 | large |
| Pathotype | nfvPPA_FTLD_vs_lvPPA_FTLD | spectral_instability | 0,010 | 0,84 | large |
| Pathotype | nfvPPA_FTLD_vs_lvPPA_FTLD | chroma_mean_0 | 0,010 | 0,84 | large |
| Pathotype | nfvPPA_FTLD_vs_lvPPA_FTLD | rolloff_std | 0,010 | 0,84 | large |
| Pathotype | FTLD_vs_healthy_control | fricatives_count | 0,010 | 0,38 | medium |
| Pathotype | nfvPPA_FTLD_vs_lvPPA_FTLD | f2 | 0,010 | 0,84 | large |
| Pathotype | svPPA_FTLD_vs_amnestic_AD | fricatives_skewness | 0,010 | 0,54 | large |
| Pathotype | healthy_control_vs_amnestic_AD | intensity_dynamic_range | 0,010 | 0,31 | medium |
| Pathotype | nfvPPA_FTLD_vs_lvPPA_FTLD | contrast_mean_0 | 0,010 | 0,84 | large |
| Pathotype | FTLD_vs_amnestic_AD | liquides_iqr | 0,010 | 0,41 | medium |
| Pathotype | FTLD_vs_amnestic_AD | zcr_mean | 0,010 | 0,41 | medium |
| Pathotype | nfvPPA_FTLD_vs_lvPPA_FTLD | delta_mfcc_mean_11 | 0,010 | 0,84 | large |
| Pathotype | svPPA_FTLD_vs_amnestic_AD | occlusives_min | 0,010 | 0,53 | large |
| Pathotype | nfvPPA_FTLD_vs_healthy_control | cv_phoneme_duration | 0,010 | 0,60 | large |
| Pathotype | nfvPPA_FTLD_vs_lvPPA_FTLD | liquides_median | 0,010 | 0,84 | large |
| Pathotype | svPPA_FTLD_vs_amnestic_AD | occlusives_median | 0,010 | 0,54 | large |
| Pathotype | svPPA_FTLD_vs_nfvPPA_FTLD | semi_voyelles_cv | 0,010 | 0,76 | large |
| Pathotype | lvPPA_FTLD_vs_lvPPA_AD | nasales_skewness | 0,010 | 0,64 | large |
| Pathotype | nfvPPA_FTLD_vs_lvPPA_FTLD | total_duration | 0,010 | 0,84 | large |
| Pathotype | healthy_control_vs_lvPPA_AD | total_duration | 0,010 | 0,44 | medium |
| Pathotype | nfvPPA_FTLD_vs_amnestic_AD | delta_mfcc_mean_1 | 0,010 | 0,62 | large |
| Pathotype | svPPA_FTLD_vs_lvPPA_AD | semi_voyelles_percentile_90 | 0,010 | 0,63 | large |
| Pathotype | svPPA_FTLD_vs_healthy_control | f1_cv,1 | 0,010 | 0,51 | large |
| Pathotype | nfvPPA_FTLD_vs_amnestic_AD | semi_voyelles_mean | 0,011 | 0,62 | large |
| Pathotype | lvPPA_FTLD_vs_healthy_control | fricatives_mean | 0,011 | 0,59 | large |
| Pathotype | lvPPA_FTLD_vs_amnestic_AD | delta_mfcc_mean_10 | 0,011 | 0,62 | large |
| Pathotype | lvPPA_FTLD_vs_amnestic_AD | f1_cv,1 | 0,011 | 0,62 | large |
| Pathotype | svPPA_FTLD_vs_nfvPPA_FTLD | voyelles_oral_iqr | 0,011 | 0,76 | large |
| Pathotype | svPPA_FTLD_vs_nfvPPA_FTLD | nasales_skewness | 0,011 | 0,76 | large |
| Pathotype | lvPPA_FTLD_vs_healthy_control | liquides_median | 0,011 | 0,59 | large |
| Pathotype | FTLD_vs_lvPPA_FTLD | f2_cv,1 | 0,011 | 0,66 | large |
| Pathotype | nfvPPA_FTLD_vs_FTLD | jitter_rap | 0,011 | 0,66 | large |
| Pathotype | FTLD_vs_lvPPA_AD | pause_mean_duration | 0,011 | 0,52 | large |
| Pathotype | lvPPA_FTLD_vs_lvPPA_AD | f0_min | 0,011 | 0,59 | large |
| Pathotype | lvPPA_FTLD_vs_healthy_control | fricatives_kurtosis | 0,011 | 0,59 | large |
| Pathotype | healthy_control_vs_lvPPA_AD | zcr_mean | 0,011 | 0,44 | medium |
| Pathotype | healthy_control_vs_lvPPA_AD | chroma_mean_0 | 0,011 | 0,44 | medium |
| Pathotype | svPPA_FTLD_vs_lvPPA_AD | semi_voyelles_median | 0,011 | 0,62 | large |
| Pathotype | nfvPPA_FTLD_vs_lvPPA_FTLD | semi_voyelles_skewness | 0,011 | 0,71 | large |
| Pathotype | svPPA_FTLD_vs_lvPPA_FTLD | f0_min | 0,011 | 0,71 | large |
| Pathotype | FTLD_vs_healthy_control | f3_cv,1 | 0,011 | 0,37 | medium |
| Pathotype | lvPPA_FTLD_vs_healthy_control | articulation_rate | 0,011 | 0,58 | large |
| Pathotype | nfvPPA_FTLD_vs_FTLD | semi_voyelles_max | 0,011 | 0,65 | large |
| Pathotype | healthy_control_vs_lvPPA_AD | semi_voyelles_percentile_90 | 0,011 | 0,43 | medium |
| Pathotype | healthy_control_vs_lvPPA_AD | nasales_std | 0,011 | 0,43 | medium |
| Pathotype | FTLD_vs_healthy_control | pause_frequency | 0,012 | 0,37 | medium |
| Pathotype | FTLD_vs_healthy_control | chroma_mean_9 | 0,012 | 0,37 | medium |
| Pathotype | nfvPPA_FTLD_vs_lvPPA_AD | semi_voyelles_mean | 0,012 | 0,69 | large |
| Pathotype | nfvPPA_FTLD_vs_lvPPA_AD | semi_voyelles_cv | 0,012 | 0,69 | large |
| Pathotype | nfvPPA_FTLD_vs_lvPPA_AD | occlusives_kurtosis | 0,012 | 0,69 | large |
| Pathotype | svPPA_FTLD_vs_lvPPA_AD | semi_voyelles_kurtosis | 0,012 | 0,61 | large |
| Pathotype | svPPA_FTLD_vs_amnestic_AD | f1_cv,1 | 0,012 | 0,53 | large |
| Pathotype | lvPPA_FTLD_vs_lvPPA_AD | voyelles_oral_percentile_10 | 0,012 | 0,69 | large |
| Pathotype | lvPPA_FTLD_vs_lvPPA_AD | cv_phoneme_duration | 0,012 | 0,69 | large |
| Pathotype | nfvPPA_FTLD_vs_FTLD | liquides_percentile_10 | 0,012 | 0,63 | large |
| Pathotype | lvPPA_FTLD_vs_healthy_control | b3 | 0,012 | 0,58 | large |
| Pathotype | svPPA_FTLD_vs_FTLD | voyelles_oral_percentile_90 | 0,012 | 0,57 | large |
| Pathotype | svPPA_FTLD_vs_FTLD | spectral_flux_std | 0,012 | 0,57 | large |
| Pathotype | nfvPPA_FTLD_vs_lvPPA_AD | f1_cv | 0,012 | 0,69 | large |
| Pathotype | nfvPPA_FTLD_vs_lvPPA_AD | b3 | 0,012 | 0,69 | large |
| Pathotype | nfvPPA_FTLD_vs_lvPPA_AD | f1_cv,1 | 0,012 | 0,69 | large |
| Pathotype | FTLD_vs_lvPPA_AD | voyelles_oral_percentile_90 | 0,012 | 0,51 | large |
| Pathotype | FTLD_vs_healthy_control | hnr_mean | 0,012 | 0,37 | medium |
| Pathotype | nfvPPA_FTLD_vs_lvPPA_AD | contrast_mean_4 | 0,012 | 0,69 | large |
| Pathotype | nfvPPA_FTLD_vs_lvPPA_AD | rmse_mean | 0,012 | 0,69 | large |
| Pathotype | nfvPPA_FTLD_vs_lvPPA_AD | intensity_mean | 0,012 | 0,69 | large |
| Pathotype | svPPA_FTLD_vs_nfvPPA_FTLD | jitter_rap | 0,012 | 0,74 | large |
| Pathotype | svPPA_FTLD_vs_nfvPPA_FTLD | pause_min_duration | 0,012 | 0,74 | large |
| Pathotype | svPPA_FTLD_vs_nfvPPA_FTLD | inter_word_pause_min | 0,012 | 0,74 | large |
| Pathotype | lvPPA_FTLD_vs_amnestic_AD | voyelles_oral_percentile_90 | 0,012 | 0,60 | large |
| Pathotype | svPPA_FTLD_vs_lvPPA_AD | pause_std_duration | 0,012 | 0,61 | large |
| Pathotype | svPPA_FTLD_vs_lvPPA_AD | inter_word_pause_std | 0,012 | 0,61 | large |
| Pathotype | nfvPPA_FTLD_vs_healthy_control | occlusives_skewness | 0,012 | 0,58 | large |
| Pathotype | lvPPA_FTLD_vs_healthy_control | f3_cv,1 | 0,012 | 0,58 | large |
| Pathotype | lvPPA_FTLD_vs_amnestic_AD | occlusives_median | 0,012 | 0,60 | large |
| Pathotype | nfvPPA_FTLD_vs_amnestic_AD | fricatives_percentile_90 | 0,012 | 0,60 | large |
| Pathotype | nfvPPA_FTLD_vs_lvPPA_FTLD | liquides_skewness | 0,013 | 0,80 | large |
| Pathotype | FTLD_vs_lvPPA_FTLD | pause_frequency | 0,013 | 0,65 | large |
| Pathotype | svPPA_FTLD_vs_lvPPA_FTLD | fricatives_percentile_90 | 0,013 | 0,74 | large |
| Pathotype | lvPPA_FTLD_vs_amnestic_AD | nasales_median | 0,013 | 0,60 | large |
| Pathotype | FTLD_vs_healthy_control | delta_mfcc_mean_10 | 0,013 | 0,36 | medium |
| Pathotype | svPPA_FTLD_vs_lvPPA_FTLD | voyelles_oral_median | 0,013 | 0,74 | large |
| Pathotype | svPPA_FTLD_vs_lvPPA_FTLD | fricatives_std | 0,013 | 0,74 | large |
| Pathotype | amnestic_AD_vs_lvPPA_AD | voyelles_oral_percentile_10 | 0,013 | 0,44 | medium |
| Pathotype | lvPPA_FTLD_vs_amnestic_AD | hnr_mean,1 | 0,013 | 0,60 | large |
| Pathotype | healthy_control_vs_lvPPA_AD | chroma_mean_5 | 0,013 | 0,43 | medium |
| Pathotype | FTLD_vs_lvPPA_AD | semi_voyelles_median | 0,013 | 0,50 | large |
| Pathotype | lvPPA_FTLD_vs_healthy_control | spectral_instability,1 | 0,013 | 0,58 | large |
| Pathotype | lvPPA_FTLD_vs_healthy_control | f1_cv,1 | 0,013 | 0,58 | large |
| Pathotype | lvPPA_FTLD_vs_healthy_control | hnr_std | 0,013 | 0,58 | large |
| Pathotype | svPPA_FTLD_vs_healthy_control | voyelles_oral_std | 0,013 | 0,49 | medium |
| Pathotype | lvPPA_FTLD_vs_healthy_control | semi_voyelles_percentile_10 | 0,013 | 0,57 | large |
| Pathotype | FTLD_vs_lvPPA_AD | liquides_iqr | 0,013 | 0,50 | large |
| Pathotype | amnestic_AD_vs_lvPPA_AD | chroma_mean_4 | 0,013 | 0,46 | medium |
| Pathotype | nfvPPA_FTLD_vs_FTLD | voyelles_oral_median | 0,013 | 0,64 | large |
| Pathotype | FTLD_vs_lvPPA_AD | chroma_mean_7 | 0,013 | 0,50 | large |
| Pathotype | svPPA_FTLD_vs_FTLD | delta_mfcc_mean_12 | 0,013 | 0,56 | large |
| Pathotype | healthy_control_vs_lvPPA_AD | semi_voyelles_iqr | 0,013 | 0,42 | medium |
| Pathotype | FTLD_vs_healthy_control | pause_median_duration | 0,013 | 0,36 | medium |
| Pathotype | FTLD_vs_healthy_control | inter_word_pause_median | 0,013 | 0,36 | medium |
| Pathotype | FTLD_vs_healthy_control | hnr_mean,1 | 0,013 | 0,36 | medium |
| Pathotype | lvPPA_FTLD_vs_amnestic_AD | voyelles_oral_iqr | 0,013 | 0,60 | large |
| Pathotype | svPPA_FTLD_vs_nfvPPA_FTLD | semi_voyelles_max | 0,013 | 0,73 | large |
| Pathotype | nfvPPA_FTLD_vs_FTLD | liquides_kurtosis | 0,013 | 0,64 | large |
| Pathotype | nfvPPA_FTLD_vs_FTLD | occlusives_std | 0,013 | 0,64 | large |
| Pathotype | svPPA_FTLD_vs_amnestic_AD | pause_frequency | 0,014 | 0,52 | large |
| Pathotype | svPPA_FTLD_vs_amnestic_AD | cv_phoneme_duration | 0,014 | 0,52 | large |
| Pathotype | FTLD_vs_lvPPA_AD | semi_voyelles_max | 0,014 | 0,50 | medium |
| Pathotype | FTLD_vs_healthy_control | f2 | 0,014 | 0,36 | medium |
| Pathotype | nfvPPA_FTLD_vs_amnestic_AD | num_pauses | 0,014 | 0,60 | large |
| Pathotype | amnestic_AD_vs_lvPPA_AD | f3 | 0,014 | 0,45 | medium |
| Pathotype | lvPPA_FTLD_vs_lvPPA_AD | fricatives_kurtosis | 0,014 | 0,67 | large |
| Pathotype | svPPA_FTLD_vs_nfvPPA_FTLD | occlusives_iqr | 0,014 | 0,73 | large |
| Pathotype | svPPA_FTLD_vs_nfvPPA_FTLD | liquides_max | 0,014 | 0,73 | large |
| Pathotype | nfvPPA_FTLD_vs_lvPPA_FTLD | jitter_local | 0,014 | 0,80 | large |
| Pathotype | FTLD_vs_healthy_control | mean_phoneme_duration | 0,014 | 0,36 | medium |
| Pathotype | FTLD_vs_healthy_control | speech_rate_phonemes_per_sec | 0,014 | 0,36 | medium |
| Pathotype | FTLD_vs_healthy_control | rate_speech_phonemes | 0,014 | 0,36 | medium |
| Pathotype | FTLD_vs_amnestic_AD | num_initial_pauses | 0,014 | 0,25 | small |
| Pathotype | nfvPPA_FTLD_vs_lvPPA_AD | semi_voyelles_min | 0,014 | 0,67 | large |
| Pathotype | nfvPPA_FTLD_vs_lvPPA_AD | mean_phoneme_duration | 0,014 | 0,67 | large |
| Pathotype | nfvPPA_FTLD_vs_lvPPA_AD | speech_rate_phonemes_per_sec | 0,014 | 0,67 | large |
| Pathotype | nfvPPA_FTLD_vs_lvPPA_FTLD | spectral_flux_std | 0,014 | 0,80 | large |
| Pathotype | svPPA_FTLD_vs_nfvPPA_FTLD | f0_mean | 0,014 | 0,73 | large |
| Pathotype | svPPA_FTLD_vs_amnestic_AD | nasales_cv | 0,014 | 0,51 | large |
| Pathotype | svPPA_FTLD_vs_amnestic_AD | mean_phoneme_duration | 0,014 | 0,51 | large |
| Pathotype | healthy_control_vs_lvPPA_AD | nasales_median | 0,014 | 0,42 | medium |
| Pathotype | nfvPPA_FTLD_vs_lvPPA_FTLD | fricatives_iqr | 0,014 | 0,80 | large |
| Pathotype | nfvPPA_FTLD_vs_healthy_control | voyelles_oral_mean | 0,014 | 0,57 | large |
| Pathotype | FTLD_vs_lvPPA_AD | rolloff_std | 0,014 | 0,50 | medium |
| Pathotype | FTLD_vs_lvPPA_FTLD | delta_mfcc_mean_10 | 0,014 | 0,63 | large |
| Pathotype | lvPPA_FTLD_vs_lvPPA_AD | spectral_instability | 0,015 | 0,67 | large |
| Pathotype | lvPPA_FTLD_vs_lvPPA_AD | chroma_mean_6 | 0,015 | 0,67 | large |
| Pathotype | nfvPPA_FTLD_vs_lvPPA_AD | delta_mfcc_mean_12 | 0,015 | 0,67 | large |
| Pathotype | healthy_control_vs_lvPPA_AD | intensity_slope | 0,015 | 0,42 | medium |
| Pathotype | FTLD_vs_lvPPA_AD | nasales_min | 0,015 | 0,49 | medium |
| Pathotype | svPPA_FTLD_vs_healthy_control | semi_voyelles_mean | 0,015 | 0,48 | medium |
| Pathotype | svPPA_FTLD_vs_FTLD | intensity_dynamic_range | 0,015 | 0,55 | large |
| Pathotype | lvPPA_FTLD_vs_amnestic_AD | spectral_instability | 0,015 | 0,59 | large |
| Pathotype | nfvPPA_FTLD_vs_healthy_control | total_duration | 0,015 | 0,56 | large |
| Pathotype | lvPPA_FTLD_vs_healthy_control | f2 | 0,015 | 0,56 | large |
| Pathotype | nfvPPA_FTLD_vs_amnestic_AD | b3 | 0,015 | 0,59 | large |
| Pathotype | healthy_control_vs_lvPPA_AD | semi_voyelles_cv | 0,015 | 0,42 | medium |
| Pathotype | svPPA_FTLD_vs_lvPPA_FTLD | fricatives_skewness | 0,015 | 0,71 | large |
| Pathotype | nfvPPA_FTLD_vs_FTLD | liquides_skewness | 0,016 | 0,63 | large |
| Pathotype | nfvPPA_FTLD_vs_FTLD | pause_mean_duration | 0,016 | 0,63 | large |
| Pathotype | nfvPPA_FTLD_vs_FTLD | inter_word_pause_mean | 0,016 | 0,63 | large |
| Pathotype | svPPA_FTLD_vs_amnestic_AD | f2_cv,1 | 0,016 | 0,51 | large |
| Pathotype | svPPA_FTLD_vs_amnestic_AD | hnr_std,1 | 0,016 | 0,51 | large |
| Pathotype | svPPA_FTLD_vs_nfvPPA_FTLD | intensity_dynamic_range | 0,016 | 0,71 | large |
| Pathotype | FTLD_vs_healthy_control | liquides_median | 0,016 | 0,35 | medium |
| Pathotype | FTLD_vs_amnestic_AD | intensity_slope | 0,016 | 0,39 | medium |
| Pathotype | FTLD_vs_lvPPA_AD | rate_speech_phonemes | 0,016 | 0,49 | medium |
| Pathotype | FTLD_vs_amnestic_AD | skew_phoneme_duration | 0,016 | 0,39 | medium |
| Pathotype | nfvPPA_FTLD_vs_healthy_control | spectral_flux_mean | 0,016 | 0,56 | large |
| Pathotype | svPPA_FTLD_vs_nfvPPA_FTLD | pause_max_duration | 0,016 | 0,71 | large |
| Pathotype | svPPA_FTLD_vs_nfvPPA_FTLD | inter_word_pause_max | 0,016 | 0,71 | large |
| Pathotype | svPPA_FTLD_vs_lvPPA_FTLD | pause_median_duration | 0,016 | 0,71 | large |
| Pathotype | svPPA_FTLD_vs_lvPPA_FTLD | inter_word_pause_median | 0,016 | 0,71 | large |
| Pathotype | svPPA_FTLD_vs_lvPPA_FTLD | pause_mean_duration | 0,017 | 0,71 | large |
| Pathotype | svPPA_FTLD_vs_lvPPA_FTLD | pause_std_duration | 0,017 | 0,71 | large |
| Pathotype | svPPA_FTLD_vs_lvPPA_FTLD | inter_word_pause_mean | 0,017 | 0,71 | large |
| Pathotype | svPPA_FTLD_vs_lvPPA_FTLD | inter_word_pause_std | 0,017 | 0,71 | large |
| Pathotype | svPPA_FTLD_vs_lvPPA_FTLD | pause_proportion | 0,017 | 0,71 | large |
| Pathotype | svPPA_FTLD_vs_lvPPA_FTLD | pause_ratio | 0,017 | 0,71 | large |
| Pathotype | svPPA_FTLD_vs_lvPPA_FTLD | fricatives_iqr | 0,017 | 0,71 | large |
| Pathotype | FTLD_vs_amnestic_AD | kurt_phoneme_duration | 0,017 | 0,38 | medium |
| Pathotype | svPPA_FTLD_vs_amnestic_AD | b2 | 0,017 | 0,50 | large |
| Pathotype | lvPPA_FTLD_vs_amnestic_AD | b1 | 0,017 | 0,58 | large |
| Pathotype | nfvPPA_FTLD_vs_FTLD | delta_mfcc_mean_12 | 0,017 | 0,62 | large |
| Pathotype | FTLD_vs_lvPPA_FTLD | hnr_mean | 0,017 | 0,62 | large |
| Pathotype | nfvPPA_FTLD_vs_FTLD | hnr_std | 0,017 | 0,62 | large |
| Pathotype | svPPA_FTLD_vs_amnestic_AD | f2_cv | 0,017 | 0,50 | large |
| Pathotype | nfvPPA_FTLD_vs_FTLD | hnr_std,1 | 0,017 | 0,62 | large |
| Pathotype | svPPA_FTLD_vs_lvPPA_AD | pause_max_duration | 0,017 | 0,59 | large |
| Pathotype | svPPA_FTLD_vs_lvPPA_AD | inter_word_pause_max | 0,017 | 0,59 | large |
| Pathotype | FTLD_vs_healthy_control | shimmer_apq3 | 0,017 | 0,35 | medium |
| Pathotype | FTLD_vs_healthy_control | shimmer_dda | 0,017 | 0,35 | medium |
| Pathotype | healthy_control_vs_amnestic_AD | f0_mean | 0,017 | 0,29 | small |
| Pathotype | lvPPA_FTLD_vs_healthy_control | occlusives_median | 0,017 | 0,54 | large |
| Pathotype | FTLD_vs_amnestic_AD | intensity_std | 0,017 | 0,38 | medium |
| Pathotype | FTLD_vs_amnestic_AD | voyelles_oral_percentile_90 | 0,018 | 0,38 | medium |
| Pathotype | svPPA_FTLD_vs_FTLD | liquides_percentile_10 | 0,018 | 0,52 | large |
| Pathotype | nfvPPA_FTLD_vs_lvPPA_AD | fricatives_skewness | 0,018 | 0,65 | large |
| Pathotype | lvPPA_FTLD_vs_lvPPA_AD | nasales_median | 0,018 | 0,65 | large |
| Pathotype | svPPA_FTLD_vs_amnestic_AD | occlusives_percentile_10 | 0,018 | 0,49 | medium |
| Pathotype | lvPPA_FTLD_vs_lvPPA_AD | fricatives_percentile_90 | 0,018 | 0,65 | large |
| Pathotype | nfvPPA_FTLD_vs_healthy_control | liquides_std | 0,018 | 0,55 | large |
| Pathotype | lvPPA_FTLD_vs_lvPPA_AD | liquides_skewness | 0,018 | 0,63 | large |
| Pathotype | lvPPA_FTLD_vs_lvPPA_AD | hnr_mean | 0,018 | 0,65 | large |
| Pathotype | FTLD_vs_healthy_control | f0_max | 0,018 | 0,34 | medium |
| Pathotype | FTLD_vs_healthy_control | occlusives_std | 0,018 | 0,34 | medium |
| Pathotype | healthy_control_vs_amnestic_AD | contrast_mean_5 | 0,018 | 0,28 | small |
| Pathotype | svPPA_FTLD_vs_amnestic_AD | f1_cv | 0,018 | 0,49 | medium |
| Pathotype | svPPA_FTLD_vs_amnestic_AD | contrast_mean_3 | 0,018 | 0,49 | medium |
| Pathotype | svPPA_FTLD_vs_amnestic_AD | pause_ratio | 0,018 | 0,49 | medium |
| Pathotype | svPPA_FTLD_vs_nfvPPA_FTLD | occlusives_max | 0,018 | 0,70 | large |
| Pathotype | svPPA_FTLD_vs_nfvPPA_FTLD | fricatives_cv | 0,018 | 0,70 | large |
| Pathotype | svPPA_FTLD_vs_nfvPPA_FTLD | cv_phoneme_duration | 0,019 | 0,70 | large |
| Pathotype | lvPPA_FTLD_vs_healthy_control | delta_mfcc_mean_6 | 0,019 | 0,55 | large |
| Pathotype | svPPA_FTLD_vs_FTLD | nasales_skewness | 0,019 | 0,53 | large |
| Pathotype | svPPA_FTLD_vs_FTLD | voyelles_oral_mean | 0,019 | 0,53 | large |
| Pathotype | svPPA_FTLD_vs_FTLD | mean_phoneme_duration | 0,019 | 0,53 | large |
| Pathotype | svPPA_FTLD_vs_FTLD | speech_rate_phonemes_per_sec | 0,019 | 0,53 | large |
| Pathotype | svPPA_FTLD_vs_FTLD | rate_speech_phonemes | 0,019 | 0,53 | large |
| Pathotype | svPPA_FTLD_vs_FTLD | fricatives_kurtosis | 0,019 | 0,53 | large |
| Pathotype | FTLD_vs_lvPPA_AD | semi_voyelles_cv | 0,019 | 0,48 | medium |
| Pathotype | FTLD_vs_lvPPA_AD | fricatives_std | 0,019 | 0,48 | medium |
| Pathotype | svPPA_FTLD_vs_amnestic_AD | delta_mfcc_mean_1 | 0,019 | 0,49 | medium |
| Pathotype | FTLD_vs_amnestic_AD | occlusives_min | 0,019 | 0,36 | medium |
| Pathotype | nfvPPA_FTLD_vs_FTLD | contrast_mean_1 | 0,019 | 0,61 | large |
| Pathotype | FTLD_vs_lvPPA_FTLD | hnr_std,1 | 0,019 | 0,61 | large |
| Pathotype | svPPA_FTLD_vs_FTLD | voyelles_oral_percentile_10 | 0,019 | 0,49 | medium |
| Pathotype | svPPA_FTLD_vs_amnestic_AD | shimmer_apq5 | 0,019 | 0,49 | medium |
| Pathotype | FTLD_vs_lvPPA_FTLD | pause_std_duration | 0,019 | 0,61 | large |
| Pathotype | FTLD_vs_lvPPA_FTLD | inter_word_pause_std | 0,019 | 0,61 | large |
| Pathotype | FTLD_vs_healthy_control | f3_cv | 0,019 | 0,34 | medium |
| Pathotype | svPPA_FTLD_vs_lvPPA_AD | semi_voyelles_count | 0,020 | 0,57 | large |
| Pathotype | amnestic_AD_vs_lvPPA_AD | delta_mfcc_mean_6 | 0,020 | 0,43 | medium |
| Pathotype | amnestic_AD_vs_lvPPA_AD | rate_speech_phonemes | 0,020 | 0,43 | medium |
| Pathotype | nfvPPA_FTLD_vs_lvPPA_FTLD | f3 | 0,020 | 0,76 | large |
| Pathotype | FTLD_vs_amnestic_AD | contrast_mean_1 | 0,020 | 0,37 | medium |
| Pathotype | svPPA_FTLD_vs_lvPPA_AD | spectral_centroid_std | 0,020 | 0,57 | large |
| Pathotype | nfvPPA_FTLD_vs_lvPPA_FTLD | spectral_flux_mean | 0,020 | 0,76 | large |
| Pathotype | svPPA_FTLD_vs_FTLD | nasales_min | 0,020 | 0,52 | large |
| Pathotype | nfvPPA_FTLD_vs_lvPPA_FTLD | spectral_centroid_slope | 0,020 | 0,76 | large |
| Pathotype | healthy_control_vs_lvPPA_AD | num_inter_word_pauses | 0,020 | 0,40 | medium |
| Pathotype | amnestic_AD_vs_lvPPA_AD | semi_voyelles_iqr | 0,020 | 0,42 | medium |
| Pathotype | nfvPPA_FTLD_vs_lvPPA_FTLD | chroma_mean_7 | 0,020 | 0,76 | large |
| Pathotype | nfvPPA_FTLD_vs_lvPPA_FTLD | chroma_mean_8 | 0,020 | 0,76 | large |
| Pathotype | nfvPPA_FTLD_vs_lvPPA_FTLD | contrast_mean_2 | 0,020 | 0,76 | large |
| Pathotype | nfvPPA_FTLD_vs_lvPPA_FTLD | contrast_mean_4 | 0,020 | 0,76 | large |
| Pathotype | nfvPPA_FTLD_vs_lvPPA_FTLD | skew_phoneme_duration | 0,020 | 0,76 | large |
| Pathotype | nfvPPA_FTLD_vs_lvPPA_FTLD | nasales_mean | 0,020 | 0,76 | large |
| Pathotype | svPPA_FTLD_vs_lvPPA_FTLD | chroma_mean_10 | 0,020 | 0,69 | large |
| Pathotype | nfvPPA_FTLD_vs_amnestic_AD | semi_voyelles_percentile_10 | 0,021 | 0,56 | large |
| Pathotype | FTLD_vs_healthy_control | syllable_rate_per_sec | 0,021 | 0,34 | medium |
| Pathotype | healthy_control_vs_lvPPA_AD | semi_voyelles_max | 0,021 | 0,40 | medium |
| Pathotype | svPPA_FTLD_vs_amnestic_AD | delta_mfcc_mean_12 | 0,021 | 0,48 | medium |
| Pathotype | svPPA_FTLD_vs_amnestic_AD | speech_rate_phonemes_per_sec | 0,021 | 0,48 | medium |
| Pathotype | healthy_control_vs_lvPPA_AD | delta_mfcc_mean_1 | 0,021 | 0,40 | medium |
| Pathotype | nfvPPA_FTLD_vs_FTLD | occlusives_skewness | 0,021 | 0,60 | large |
| Pathotype | svPPA_FTLD_vs_nfvPPA_FTLD | liquides_count | 0,021 | 0,67 | large |
| Pathotype | nfvPPA_FTLD_vs_lvPPA_AD | num_inter_word_pauses | 0,021 | 0,63 | large |
| Pathotype | nfvPPA_FTLD_vs_lvPPA_FTLD | nasales_median | 0,021 | 0,76 | large |
| Pathotype | svPPA_FTLD_vs_amnestic_AD | voyelles_oral_std | 0,021 | 0,48 | medium |
| Pathotype | nfvPPA_FTLD_vs_lvPPA_AD | nasales_min | 0,021 | 0,63 | large |
| Pathotype | nfvPPA_FTLD_vs_amnestic_AD | num_inter_word_pauses | 0,021 | 0,56 | large |
| Pathotype | healthy_control_vs_lvPPA_AD | fricatives_cv | 0,021 | 0,39 | medium |
| Pathotype | lvPPA_FTLD_vs_lvPPA_AD | voyelles_oral_mean | 0,022 | 0,63 | large |
| Pathotype | lvPPA_FTLD_vs_healthy_control | delta_mfcc_mean_3 | 0,022 | 0,53 | large |
| Pathotype | nfvPPA_FTLD_vs_amnestic_AD | pause_frequency | 0,022 | 0,56 | large |
| Pathotype | lvPPA_FTLD_vs_lvPPA_AD | spectral_centroid_slope | 0,022 | 0,63 | large |
| Pathotype | lvPPA_FTLD_vs_lvPPA_AD | spectral_flux,1 | 0,022 | 0,63 | large |
| Pathotype | healthy_control_vs_amnestic_AD | articulation_rate | 0,022 | 0,27 | small |
| Pathotype | nfvPPA_FTLD_vs_lvPPA_AD | f3 | 0,022 | 0,63 | large |
| Pathotype | nfvPPA_FTLD_vs_lvPPA_AD | contrast_mean_2 | 0,022 | 0,63 | large |
| Pathotype | svPPA_FTLD_vs_amnestic_AD | liquides_iqr | 0,022 | 0,48 | medium |
| Pathotype | FTLD_vs_lvPPA_FTLD | spectral_instability | 0,022 | 0,59 | large |
| Pathotype | FTLD_vs_lvPPA_FTLD | delta_mfcc_mean_7 | 0,022 | 0,59 | large |
| Pathotype | FTLD_vs_lvPPA_FTLD | delta_mfcc_mean_4 | 0,022 | 0,59 | large |
| Pathotype | amnestic_AD_vs_lvPPA_AD | voyelles_oral_median | 0,022 | 0,42 | medium |
| Pathotype | nfvPPA_FTLD_vs_amnestic_AD | num_inter_pauses | 0,022 | 0,55 | large |
| Pathotype | FTLD_vs_lvPPA_FTLD | delta_mfcc_mean_1 | 0,022 | 0,59 | large |
| Pathotype | FTLD_vs_lvPPA_AD | semi_voyelles_std | 0,023 | 0,46 | medium |
| Pathotype | svPPA_FTLD_vs_healthy_control | semi_voyelles_median | 0,023 | 0,45 | medium |
| Pathotype | healthy_control_vs_lvPPA_AD | liquides_kurtosis | 0,023 | 0,39 | medium |
| Pathotype | healthy_control_vs_amnestic_AD | median_phoneme_duration | 0,023 | 0,26 | small |
| Pathotype | healthy_control_vs_amnestic_AD | intensity_mean | 0,023 | 0,27 | small |
| Pathotype | lvPPA_FTLD_vs_lvPPA_AD | min_phoneme_duration | 0,023 | 0,61 | large |
| Pathotype | svPPA_FTLD_vs_lvPPA_AD | rolloff_std | 0,023 | 0,56 | large |
| Pathotype | svPPA_FTLD_vs_healthy_control | phoneme_count | 0,023 | 0,45 | medium |
| Pathotype | svPPA_FTLD_vs_healthy_control | num_speech_phonemes | 0,023 | 0,45 | medium |
| Pathotype | FTLD_vs_healthy_control | pause_total_duration | 0,023 | 0,33 | medium |
| Pathotype | lvPPA_FTLD_vs_amnestic_AD | fricatives_mean | 0,023 | 0,55 | large |
| Pathotype | nfvPPA_FTLD_vs_amnestic_AD | semi_voyelles_min | 0,023 | 0,54 | large |
| Pathotype | svPPA_FTLD_vs_nfvPPA_FTLD | semi_voyelles_skewness | 0,023 | 0,67 | large |
| Pathotype | svPPA_FTLD_vs_nfvPPA_FTLD | fricatives_percentile_10 | 0,024 | 0,67 | large |
| Pathotype | amnestic_AD_vs_lvPPA_AD | pause_std_duration | 0,024 | 0,42 | medium |
| Pathotype | amnestic_AD_vs_lvPPA_AD | inter_word_pause_std | 0,024 | 0,42 | medium |
| Pathotype | svPPA_FTLD_vs_lvPPA_AD | delta_mfcc_mean_6 | 0,024 | 0,56 | large |
| Pathotype | svPPA_FTLD_vs_amnestic_AD | articulation_rate | 0,024 | 0,47 | medium |
| Pathotype | nfvPPA_FTLD_vs_FTLD | voyelles_oral_kurtosis | 0,024 | 0,59 | large |
| Pathotype | svPPA_FTLD_vs_amnestic_AD | semi_voyelles_iqr | 0,024 | 0,47 | medium |
| Pathotype | lvPPA_FTLD_vs_healthy_control | f0_slope | 0,024 | 0,52 | large |
| Pathotype | lvPPA_FTLD_vs_healthy_control | f3_cv | 0,024 | 0,52 | large |
| Pathotype | FTLD_vs_healthy_control | semi_voyelles_percentile_90 | 0,024 | 0,33 | medium |
| Pathotype | svPPA_FTLD_vs_amnestic_AD | spectral_centroid_std | 0,024 | 0,47 | medium |
| Pathotype | svPPA_FTLD_vs_amnestic_AD | pause_std_duration | 0,024 | 0,47 | medium |
| Pathotype | svPPA_FTLD_vs_amnestic_AD | inter_word_pause_std | 0,024 | 0,47 | medium |
| Pathotype | FTLD_vs_lvPPA_FTLD | semi_voyelles_skewness | 0,024 | 0,57 | large |
| Pathotype | svPPA_FTLD_vs_nfvPPA_FTLD | median_phoneme_duration | 0,025 | 0,66 | large |
| Pathotype | amnestic_AD_vs_lvPPA_AD | fricatives_skewness | 0,025 | 0,41 | medium |
| Pathotype | healthy_control_vs_amnestic_AD | rmse_mean | 0,025 | 0,27 | small |
| Pathotype | FTLD_vs_lvPPA_AD | delta_mfcc_mean_4 | 0,025 | 0,46 | medium |
| Pathotype | FTLD_vs_lvPPA_AD | chroma_mean_4 | 0,025 | 0,46 | medium |
| Pathotype | FTLD_vs_lvPPA_AD | f3 | 0,025 | 0,46 | medium |
| Pathotype | amnestic_AD_vs_lvPPA_AD | chroma_mean_10 | 0,025 | 0,41 | medium |
| Pathotype | FTLD_vs_healthy_control | nasales_count | 0,025 | 0,33 | medium |
| Pathotype | lvPPA_FTLD_vs_amnestic_AD | semi_voyelles_percentile_10 | 0,025 | 0,54 | large |
| Pathotype | nfvPPA_FTLD_vs_amnestic_AD | pause_mean_duration | 0,026 | 0,54 | large |
| Pathotype | FTLD_vs_lvPPA_FTLD | jitter_ppq5 | 0,026 | 0,58 | large |
| Pathotype | FTLD_vs_lvPPA_FTLD | liquides_kurtosis | 0,026 | 0,57 | large |
| Pathotype | svPPA_FTLD_vs_lvPPA_FTLD | occlusives_percentile_10 | 0,026 | 0,66 | large |
| Pathotype | nfvPPA_FTLD_vs_amnestic_AD | voyelles_oral_cv | 0,026 | 0,54 | large |
| Pathotype | nfvPPA_FTLD_vs_lvPPA_AD | occlusives_skewness | 0,026 | 0,61 | large |
| Pathotype | lvPPA_FTLD_vs_lvPPA_AD | pause_cv | 0,026 | 0,61 | large |
| Pathotype | lvPPA_FTLD_vs_lvPPA_AD | pause_proportion | 0,026 | 0,61 | large |
| Pathotype | lvPPA_FTLD_vs_healthy_control | chroma_mean_7 | 0,026 | 0,52 | large |
| Pathotype | FTLD_vs_healthy_control | syllable_count | 0,027 | 0,32 | medium |
| Pathotype | FTLD_vs_healthy_control | voyelles_oral_count | 0,027 | 0,32 | medium |
| Pathotype | svPPA_FTLD_vs_nfvPPA_FTLD | rolloff_std | 0,027 | 0,66 | large |
| Pathotype | nfvPPA_FTLD_vs_amnestic_AD | semi_voyelles_count | 0,027 | 0,54 | large |
| Pathotype | svPPA_FTLD_vs_nfvPPA_FTLD | voyelles_oral_percentile_10 | 0,027 | 0,66 | large |
| Pathotype | nfvPPA_FTLD_vs_healthy_control | occlusives_percentile_10 | 0,027 | 0,49 | medium |
| Pathotype | svPPA_FTLD_vs_healthy_control | semi_voyelles_iqr | 0,027 | 0,44 | medium |
| Pathotype | nfvPPA_FTLD_vs_amnestic_AD | f0_max | 0,027 | 0,54 | large |
| Pathotype | FTLD_vs_amnestic_AD | voyelles_oral_kurtosis | 0,027 | 0,35 | medium |
| Pathotype | svPPA_FTLD_vs_amnestic_AD | num_inter_pauses | 0,027 | 0,46 | medium |
| Pathotype | FTLD_vs_lvPPA_AD | syllable_rate_per_sec | 0,027 | 0,45 | medium |
| Pathotype | svPPA_FTLD_vs_lvPPA_AD | speech_duration | 0,027 | 0,54 | large |
| Pathotype | svPPA_FTLD_vs_lvPPA_AD | spectral_flux | 0,027 | 0,54 | large |
| Pathotype | svPPA_FTLD_vs_lvPPA_AD | spectral_flux,1 | 0,027 | 0,54 | large |
| Pathotype | nfvPPA_FTLD_vs_FTLD | pause_median_duration | 0,027 | 0,57 | large |
| Pathotype | nfvPPA_FTLD_vs_FTLD | inter_word_pause_median | 0,027 | 0,57 | large |
| Pathotype | svPPA_FTLD_vs_amnestic_AD | hnr_std | 0,027 | 0,46 | medium |
| Pathotype | svPPA_FTLD_vs_amnestic_AD | contrast_mean_5 | 0,027 | 0,46 | medium |
| Pathotype | svPPA_FTLD_vs_amnestic_AD | intensity_slope | 0,027 | 0,46 | medium |
| Pathotype | lvPPA_FTLD_vs_lvPPA_AD | liquides_count | 0,027 | 0,60 | large |
| Pathotype | nfvPPA_FTLD_vs_FTLD | nasales_kurtosis | 0,027 | 0,57 | large |
| Pathotype | svPPA_FTLD_vs_healthy_control | pause_std_duration | 0,027 | 0,44 | medium |
| Pathotype | svPPA_FTLD_vs_healthy_control | inter_word_pause_std | 0,027 | 0,44 | medium |
| Pathotype | nfvPPA_FTLD_vs_FTLD | voyelles_oral_skewness | 0,027 | 0,57 | large |
| Pathotype | svPPA_FTLD_vs_lvPPA_AD | b2 | 0,027 | 0,54 | large |
| Pathotype | nfvPPA_FTLD_vs_FTLD | total_duration | 0,027 | 0,57 | large |
| Pathotype | nfvPPA_FTLD_vs_FTLD | cv_phoneme_duration | 0,028 | 0,57 | large |
| Pathotype | healthy_control_vs_amnestic_AD | spectral_instability | 0,028 | 0,27 | small |
| Pathotype | svPPA_FTLD_vs_lvPPA_FTLD | pause_max_duration | 0,028 | 0,66 | large |
| Pathotype | svPPA_FTLD_vs_lvPPA_FTLD | inter_word_pause_max | 0,028 | 0,66 | large |
| Pathotype | lvPPA_FTLD_vs_healthy_control | delta_mfcc_mean_7 | 0,028 | 0,51 | large |
| Pathotype | nfvPPA_FTLD_vs_healthy_control | delta_mfcc_mean_3 | 0,028 | 0,51 | large |
| Pathotype | svPPA_FTLD_vs_lvPPA_FTLD | voyelles_oral_mean | 0,028 | 0,66 | large |
| Pathotype | FTLD_vs_healthy_control | nasales_max | 0,028 | 0,32 | medium |
| Pathotype | amnestic_AD_vs_lvPPA_AD | fricatives_std | 0,028 | 0,40 | medium |
| Pathotype | nfvPPA_FTLD_vs_healthy_control | nasales_percentile_10 | 0,028 | 0,50 | large |
| Pathotype | nfvPPA_FTLD_vs_amnestic_AD | delta_mfcc_mean_8 | 0,028 | 0,53 | large |
| Pathotype | FTLD_vs_lvPPA_AD | occlusives_min | 0,028 | 0,42 | medium |
| Pathotype | nfvPPA_FTLD_vs_lvPPA_FTLD | f2_cv | 0,028 | 0,71 | large |
| Pathotype | nfvPPA_FTLD_vs_lvPPA_FTLD | pause_mean_duration | 0,029 | 0,71 | large |
| Pathotype | nfvPPA_FTLD_vs_lvPPA_FTLD | pause_median_duration | 0,029 | 0,71 | large |
| Pathotype | nfvPPA_FTLD_vs_lvPPA_FTLD | inter_word_pause_mean | 0,029 | 0,71 | large |
| Pathotype | nfvPPA_FTLD_vs_lvPPA_FTLD | inter_word_pause_median | 0,029 | 0,71 | large |
| Pathotype | nfvPPA_FTLD_vs_lvPPA_FTLD | pause_proportion | 0,029 | 0,71 | large |
| Pathotype | nfvPPA_FTLD_vs_lvPPA_FTLD | pause_ratio | 0,029 | 0,71 | large |
| Pathotype | nfvPPA_FTLD_vs_lvPPA_FTLD | voyelles_oral_mean | 0,029 | 0,71 | large |
| Pathotype | nfvPPA_FTLD_vs_lvPPA_FTLD | voyelles_oral_std | 0,029 | 0,71 | large |
| Pathotype | nfvPPA_FTLD_vs_lvPPA_FTLD | voyelles_oral_cv | 0,029 | 0,71 | large |
| Pathotype | nfvPPA_FTLD_vs_lvPPA_FTLD | voyelles_oral_percentile_90 | 0,029 | 0,71 | large |
| Pathotype | svPPA_FTLD_vs_amnestic_AD | num_inter_word_pauses | 0,029 | 0,46 | medium |
| Pathotype | lvPPA_FTLD_vs_healthy_control | b2 | 0,029 | 0,51 | large |
| Pathotype | amnestic_AD_vs_lvPPA_AD | occlusives_max | 0,029 | 0,40 | medium |
| Pathotype | FTLD_vs_lvPPA_FTLD | contrast_mean_1 | 0,029 | 0,56 | large |
| Pathotype | FTLD_vs_lvPPA_AD | occlusives_percentile_90 | 0,029 | 0,44 | medium |
| Pathotype | FTLD_vs_lvPPA_FTLD | contrast_mean_6 | 0,029 | 0,56 | large |
| Pathotype | FTLD_vs_lvPPA_FTLD | rolloff_std | 0,029 | 0,56 | large |
| Pathotype | amnestic_AD_vs_lvPPA_AD | semi_voyelles_mean | 0,029 | 0,40 | medium |
| Pathotype | svPPA_FTLD_vs_healthy_control | jitter_ppq5 | 0,030 | 0,43 | medium |
| Pathotype | healthy_control_vs_lvPPA_AD | nasales_mean | 0,030 | 0,37 | medium |
| Pathotype | healthy_control_vs_lvPPA_AD | voyelles_oral_percentile_10 | 0,030 | 0,34 | medium |
| Pathotype | svPPA_FTLD_vs_lvPPA_FTLD | semi_voyelles_kurtosis | 0,030 | 0,60 | large |
| Pathotype | lvPPA_FTLD_vs_healthy_control | fricatives_min | 0,030 | 0,49 | medium |
| Pathotype | FTLD_vs_healthy_control | phoneme_count | 0,030 | 0,32 | medium |
| Pathotype | FTLD_vs_healthy_control | num_speech_phonemes | 0,030 | 0,32 | medium |
| Pathotype | svPPA_FTLD_vs_lvPPA_AD | semi_voyelles_skewness | 0,030 | 0,53 | large |
| Pathotype | nfvPPA_FTLD_vs_FTLD | semi_voyelles_count | 0,030 | 0,54 | large |
| Pathotype | nfvPPA_FTLD_vs_amnestic_AD | zcr_mean | 0,030 | 0,52 | large |
| Pathotype | svPPA_FTLD_vs_nfvPPA_FTLD | max_phoneme_duration | 0,030 | 0,64 | large |
| Pathotype | lvPPA_FTLD_vs_healthy_control | syllable_rate_per_sec | 0,030 | 0,50 | large |
| Pathotype | FTLD_vs_healthy_control | nasales_iqr | 0,031 | 0,32 | medium |
| Pathotype | lvPPA_FTLD_vs_amnestic_AD | f0_mean | 0,031 | 0,52 | large |
| Pathotype | lvPPA_FTLD_vs_amnestic_AD | spectral_flux | 0,031 | 0,52 | large |
| Pathotype | amnestic_AD_vs_lvPPA_AD | fricatives_iqr | 0,031 | 0,40 | medium |
| Pathotype | FTLD_vs_healthy_control | semi_voyelles_cv | 0,031 | 0,31 | medium |
| Pathotype | svPPA_FTLD_vs_FTLD | occlusives_iqr | 0,031 | 0,49 | medium |
| Pathotype | healthy_control_vs_amnestic_AD | occlusives_percentile_90 | 0,031 | 0,26 | small |
| Pathotype | svPPA_FTLD_vs_lvPPA_AD | f2_cv | 0,032 | 0,53 | large |
| Pathotype | svPPA_FTLD_vs_lvPPA_AD | f2_cv,1 | 0,032 | 0,53 | large |
| Pathotype | svPPA_FTLD_vs_lvPPA_FTLD | liquides_min | 0,032 | 0,63 | large |
| Pathotype | FTLD_vs_healthy_control | semi_voyelles_mean | 0,032 | 0,31 | medium |
| Pathotype | nfvPPA_FTLD_vs_lvPPA_AD | syllable_count | 0,032 | 0,59 | large |
| Pathotype | amnestic_AD_vs_lvPPA_AD | semi_voyelles_min | 0,032 | 0,39 | medium |
| Pathotype | lvPPA_FTLD_vs_lvPPA_AD | voyelles_oral_std | 0,032 | 0,59 | large |
| Pathotype | lvPPA_FTLD_vs_lvPPA_AD | semi_voyelles_mean | 0,032 | 0,59 | large |
| Pathotype | lvPPA_FTLD_vs_lvPPA_AD | semi_voyelles_median | 0,032 | 0,59 | large |
| Pathotype | lvPPA_FTLD_vs_lvPPA_AD | fricatives_mean | 0,032 | 0,59 | large |
| Pathotype | lvPPA_FTLD_vs_lvPPA_AD | liquides_median | 0,032 | 0,59 | large |
| Pathotype | lvPPA_FTLD_vs_healthy_control | delta_mfcc_mean_2 | 0,032 | 0,50 | medium |
| Pathotype | FTLD_vs_amnestic_AD | delta_mfcc_mean_4 | 0,032 | 0,34 | medium |
| Pathotype | lvPPA_FTLD_vs_lvPPA_AD | delta_mfcc_mean_3 | 0,032 | 0,59 | large |
| Pathotype | lvPPA_FTLD_vs_lvPPA_AD | delta_mfcc_mean_8 | 0,032 | 0,59 | large |
| Pathotype | lvPPA_FTLD_vs_lvPPA_AD | delta_mfcc_mean_11 | 0,032 | 0,59 | large |
| Pathotype | lvPPA_FTLD_vs_lvPPA_AD | chroma_mean_2 | 0,032 | 0,59 | large |
| Pathotype | lvPPA_FTLD_vs_lvPPA_AD | contrast_mean_6 | 0,032 | 0,59 | large |
| Pathotype | lvPPA_FTLD_vs_lvPPA_AD | spectral_flux | 0,032 | 0,59 | large |
| Pathotype | svPPA_FTLD_vs_FTLD | semi_voyelles_skewness | 0,032 | 0,49 | medium |
| Pathotype | svPPA_FTLD_vs_FTLD | chroma_mean_3 | 0,033 | 0,49 | medium |
| Pathotype | svPPA_FTLD_vs_FTLD | nasales_kurtosis | 0,033 | 0,49 | medium |
| Pathotype | svPPA_FTLD_vs_lvPPA_AD | occlusives_min | 0,033 | 0,51 | large |
| Pathotype | lvPPA_FTLD_vs_amnestic_AD | delta_mfcc_mean_6 | 0,033 | 0,52 | large |
| Pathotype | nfvPPA_FTLD_vs_amnestic_AD | spectral_flux_mean | 0,033 | 0,52 | large |
| Pathotype | nfvPPA_FTLD_vs_amnestic_AD | b2 | 0,033 | 0,52 | large |
| Pathotype | nfvPPA_FTLD_vs_amnestic_AD | voyelles_oral_min | 0,033 | 0,48 | medium |
| Pathotype | FTLD_vs_amnestic_AD | contrast_mean_6 | 0,033 | 0,34 | medium |
| Pathotype | FTLD_vs_healthy_control | intensity_dynamic_range | 0,033 | 0,31 | medium |
| Pathotype | FTLD_vs_lvPPA_AD | fricatives_iqr | 0,033 | 0,43 | medium |
| Pathotype | nfvPPA_FTLD_vs_healthy_control | fricatives_std | 0,033 | 0,49 | medium |
| Pathotype | nfvPPA_FTLD_vs_FTLD | rolloff_std | 0,034 | 0,55 | large |
| Pathotype | svPPA_FTLD_vs_healthy_control | num_final_pauses | 0,034 | 0,16 | small |
| Pathotype | svPPA_FTLD_vs_nfvPPA_FTLD | voyelles_oral_percentile_90 | 0,034 | 0,63 | large |
| Pathotype | svPPA_FTLD_vs_amnestic_AD | rate_speech_phonemes | 0,034 | 0,44 | medium |
| Pathotype | healthy_control_vs_lvPPA_AD | b3 | 0,034 | 0,36 | medium |
| Pathotype | svPPA_FTLD_vs_nfvPPA_FTLD | delta_mfcc_mean_8 | 0,034 | 0,63 | large |
| Pathotype | healthy_control_vs_lvPPA_AD | min_phoneme_duration | 0,034 | 0,34 | medium |
| Pathotype | nfvPPA_FTLD_vs_FTLD | f0_min | 0,035 | 0,46 | medium |
| Pathotype | FTLD_vs_lvPPA_AD | semi_voyelles_iqr | 0,035 | 0,43 | medium |
| Pathotype | svPPA_FTLD_vs_nfvPPA_FTLD | syllable_rate_per_sec | 0,035 | 0,63 | large |
| Pathotype | svPPA_FTLD_vs_nfvPPA_FTLD | voyelles_oral_skewness | 0,035 | 0,63 | large |
| Pathotype | svPPA_FTLD_vs_nfvPPA_FTLD | voyelles_oral_kurtosis | 0,035 | 0,63 | large |
| Pathotype | svPPA_FTLD_vs_lvPPA_FTLD | b1 | 0,035 | 0,63 | large |
| Pathotype | FTLD_vs_lvPPA_AD | std_phoneme_duration | 0,035 | 0,43 | medium |
| Pathotype | lvPPA_FTLD_vs_healthy_control | voyelles_oral_iqr | 0,035 | 0,49 | medium |
| Pathotype | nfvPPA_FTLD_vs_healthy_control | nasales_kurtosis | 0,035 | 0,49 | medium |
| Pathotype | svPPA_FTLD_vs_amnestic_AD | voyelles_oral_min | 0,035 | 0,39 | medium |
| Pathotype | nfvPPA_FTLD_vs_healthy_control | delta_mfcc_mean_10 | 0,035 | 0,49 | medium |
| Pathotype | FTLD_vs_lvPPA_AD | chroma_mean_0 | 0,035 | 0,43 | medium |
| Pathotype | svPPA_FTLD_vs_lvPPA_FTLD | voyelles_oral_std | 0,035 | 0,63 | large |
| Pathotype | nfvPPA_FTLD_vs_FTLD | fricatives_std | 0,036 | 0,54 | large |
| Pathotype | healthy_control_vs_amnestic_AD | nasales_min | 0,036 | 0,25 | small |
| Pathotype | svPPA_FTLD_vs_FTLD | max_phoneme_duration | 0,036 | 0,48 | medium |
| Pathotype | svPPA_FTLD_vs_FTLD | voyelles_oral_std | 0,036 | 0,48 | medium |
| Pathotype | svPPA_FTLD_vs_lvPPA_AD | semi_voyelles_iqr | 0,036 | 0,51 | large |
| Pathotype | svPPA_FTLD_vs_lvPPA_AD | fricatives_count | 0,036 | 0,51 | large |
| Pathotype | svPPA_FTLD_vs_lvPPA_AD | cv_phoneme_duration | 0,036 | 0,51 | large |
| Pathotype | nfvPPA_FTLD_vs_healthy_control | chroma_mean_7 | 0,037 | 0,48 | medium |
| Pathotype | nfvPPA_FTLD_vs_healthy_control | b1 | 0,037 | 0,48 | medium |
| Pathotype | FTLD_vs_amnestic_AD | semi_voyelles_mean | 0,037 | 0,33 | medium |
| Pathotype | svPPA_FTLD_vs_healthy_control | articulation_rate | 0,037 | 0,41 | medium |
| Pathotype | FTLD_vs_lvPPA_AD | voyelles_oral_median | 0,038 | 0,42 | medium |
| Pathotype | FTLD_vs_lvPPA_AD | delta_mfcc_mean_1 | 0,038 | 0,42 | medium |
| Pathotype | nfvPPA_FTLD_vs_FTLD | spectral_flux_mean | 0,038 | 0,54 | large |
| Pathotype | nfvPPA_FTLD_vs_FTLD | f1_cv,1 | 0,038 | 0,54 | large |
| Pathotype | nfvPPA_FTLD_vs_FTLD | f1_cv | 0,038 | 0,54 | large |
| Pathotype | FTLD_vs_lvPPA_FTLD | intensity_std | 0,038 | 0,54 | large |
| Pathotype | amnestic_AD_vs_lvPPA_AD | delta_mfcc_mean_9 | 0,038 | 0,38 | medium |
| Pathotype | lvPPA_FTLD_vs_lvPPA_AD | occlusives_median | 0,038 | 0,57 | large |
| Pathotype | nfvPPA_FTLD_vs_lvPPA_FTLD | jitter_rap | 0,038 | 0,67 | large |
| Pathotype | amnestic_AD_vs_lvPPA_AD | semi_voyelles_cv | 0,039 | 0,38 | medium |
| Pathotype | amnestic_AD_vs_lvPPA_AD | chroma_mean_5 | 0,039 | 0,38 | medium |
| Pathotype | lvPPA_FTLD_vs_lvPPA_AD | delta_mfcc_mean_6 | 0,039 | 0,57 | large |
| Pathotype | lvPPA_FTLD_vs_lvPPA_AD | rmse_mean | 0,039 | 0,57 | large |
| Pathotype | lvPPA_FTLD_vs_lvPPA_AD | intensity_mean | 0,039 | 0,57 | large |
| Pathotype | svPPA_FTLD_vs_nfvPPA_FTLD | voyelles_oral_cv | 0,039 | 0,61 | large |
| Pathotype | nfvPPA_FTLD_vs_lvPPA_AD | spectral_centroid_mean | 0,039 | 0,57 | large |
| Pathotype | nfvPPA_FTLD_vs_lvPPA_AD | rolloff_mean | 0,039 | 0,57 | large |
| Pathotype | nfvPPA_FTLD_vs_lvPPA_AD | spectral_flux_mean | 0,039 | 0,57 | large |
| Pathotype | nfvPPA_FTLD_vs_lvPPA_FTLD | delta_mfcc_mean_10 | 0,039 | 0,67 | large |
| Pathotype | FTLD_vs_healthy_control | liquides_percentile_10 | 0,039 | 0,30 | small |
| Pathotype | nfvPPA_FTLD_vs_lvPPA_FTLD | delta_mfcc_mean_5 | 0,039 | 0,67 | large |
| Pathotype | nfvPPA_FTLD_vs_lvPPA_FTLD | delta_mfcc_mean_7 | 0,039 | 0,67 | large |
| Pathotype | FTLD_vs_amnestic_AD | rmse_mean | 0,039 | 0,33 | medium |
| Pathotype | FTLD_vs_amnestic_AD | intensity_mean | 0,039 | 0,33 | medium |
| Pathotype | nfvPPA_FTLD_vs_lvPPA_FTLD | pause_total_duration | 0,039 | 0,67 | large |
| Pathotype | nfvPPA_FTLD_vs_healthy_control | min_phoneme_duration | 0,040 | 0,45 | medium |
| Pathotype | healthy_control_vs_amnestic_AD | spectral_flux_mean | 0,040 | 0,25 | small |
| Pathotype | svPPA_FTLD_vs_amnestic_AD | syllable_count | 0,040 | 0,43 | medium |
| Pathotype | nfvPPA_FTLD_vs_FTLD | num_pauses | 0,040 | 0,53 | large |
| Pathotype | nfvPPA_FTLD_vs_FTLD | num_inter_word_pauses | 0,040 | 0,53 | large |
| Pathotype | nfvPPA_FTLD_vs_FTLD | num_inter_pauses | 0,040 | 0,53 | large |
| Pathotype | nfvPPA_FTLD_vs_healthy_control | fricatives_skewness | 0,040 | 0,48 | medium |
| Pathotype | svPPA_FTLD_vs_amnestic_AD | occlusives_count | 0,040 | 0,43 | medium |
| Pathotype | FTLD_vs_amnestic_AD | median_phoneme_duration | 0,040 | 0,32 | medium |
| Pathotype | svPPA_FTLD_vs_amnestic_AD | voyelles_oral_skewness | 0,041 | 0,43 | medium |
| Pathotype | FTLD_vs_amnestic_AD | f3_cv,1 | 0,041 | 0,33 | medium |
| Pathotype | FTLD_vs_lvPPA_AD | occlusives_median | 0,041 | 0,41 | medium |
| Pathotype | svPPA_FTLD_vs_amnestic_AD | shimmer_local | 0,041 | 0,43 | medium |
| Pathotype | healthy_control_vs_lvPPA_AD | voyelles_oral_median | 0,041 | 0,34 | medium |
| Pathotype | healthy_control_vs_amnestic_AD | voyelles_oral_skewness | 0,041 | 0,25 | small |
| Pathotype | FTLD_vs_lvPPA_AD | occlusives_max | 0,042 | 0,41 | medium |
| Pathotype | nfvPPA_FTLD_vs_amnestic_AD | fricatives_skewness | 0,042 | 0,49 | medium |
| Pathotype | svPPA_FTLD_vs_FTLD | num_initial_pauses | 0,042 | 0,20 | small |
| Pathotype | svPPA_FTLD_vs_FTLD | num_final_pauses | 0,042 | 0,20 | small |
| Pathotype | nfvPPA_FTLD_vs_healthy_control | voyelles_oral_max | 0,042 | 0,47 | medium |
| Pathotype | healthy_control_vs_lvPPA_AD | max_phoneme_duration | 0,042 | 0,35 | medium |
| Pathotype | svPPA_FTLD_vs_lvPPA_AD | fricatives_max | 0,042 | 0,50 | large |
| Pathotype | svPPA_FTLD_vs_lvPPA_AD | liquides_std | 0,042 | 0,50 | large |
| Pathotype | svPPA_FTLD_vs_lvPPA_AD | liquides_iqr | 0,042 | 0,50 | large |
| Pathotype | svPPA_FTLD_vs_lvPPA_AD | pause_total_duration | 0,042 | 0,50 | large |
| Pathotype | svPPA_FTLD_vs_lvPPA_AD | pause_cv | 0,042 | 0,50 | large |
| Pathotype | lvPPA_FTLD_vs_amnestic_AD | liquides_min | 0,042 | 0,48 | medium |
| Pathotype | lvPPA_FTLD_vs_amnestic_AD | f0_slope | 0,042 | 0,49 | medium |
| Pathotype | lvPPA_FTLD_vs_amnestic_AD | spectral_centroid_slope | 0,042 | 0,49 | medium |
| Pathotype | lvPPA_FTLD_vs_amnestic_AD | pause_total_duration | 0,042 | 0,49 | medium |
| Pathotype | healthy_control_vs_amnestic_AD | contrast_mean_6 | 0,043 | 0,24 | small |
| Pathotype | healthy_control_vs_amnestic_AD | chroma_mean_9 | 0,043 | 0,24 | small |
| Pathotype | FTLD_vs_lvPPA_AD | nasales_median | 0,043 | 0,41 | medium |
| Pathotype | svPPA_FTLD_vs_nfvPPA_FTLD | syllable_count | 0,043 | 0,60 | large |
| Pathotype | svPPA_FTLD_vs_nfvPPA_FTLD | voyelles_oral_count | 0,043 | 0,60 | large |
| Pathotype | svPPA_FTLD_vs_nfvPPA_FTLD | occlusives_count | 0,043 | 0,60 | large |
| Pathotype | FTLD_vs_lvPPA_FTLD | fricatives_median | 0,043 | 0,52 | large |
| Pathotype | svPPA_FTLD_vs_nfvPPA_FTLD | semi_voyelles_median | 0,043 | 0,60 | large |
| Pathotype | svPPA_FTLD_vs_lvPPA_FTLD | contrast_mean_4 | 0,043 | 0,60 | large |
| Pathotype | svPPA_FTLD_vs_healthy_control | max_phoneme_duration | 0,043 | 0,40 | medium |
| Pathotype | svPPA_FTLD_vs_healthy_control | contrast_mean_1 | 0,043 | 0,40 | medium |
| Pathotype | svPPA_FTLD_vs_healthy_control | contrast_mean_3 | 0,043 | 0,40 | medium |
| Pathotype | nfvPPA_FTLD_vs_FTLD | delta_mfcc_mean_3 | 0,043 | 0,52 | large |
| Pathotype | healthy_control_vs_lvPPA_AD | delta_mfcc_mean_4 | 0,043 | 0,35 | medium |
| Pathotype | FTLD_vs_healthy_control | semi_voyelles_std | 0,043 | 0,29 | small |
| Pathotype | svPPA_FTLD_vs_amnestic_AD | pause_min_duration | 0,044 | 0,42 | medium |
| Pathotype | svPPA_FTLD_vs_amnestic_AD | inter_word_pause_min | 0,044 | 0,42 | medium |
| Pathotype | svPPA_FTLD_vs_lvPPA_FTLD | voyelles_oral_percentile_90 | 0,044 | 0,60 | large |
| Pathotype | amnestic_AD_vs_lvPPA_AD | min_phoneme_duration | 0,044 | 0,35 | medium |
| Pathotype | FTLD_vs_amnestic_AD | f2 | 0,044 | 0,32 | medium |
| Pathotype | nfvPPA_FTLD_vs_healthy_control | f3_cv,1 | 0,044 | 0,47 | medium |
| Pathotype | svPPA_FTLD_vs_nfvPPA_FTLD | fricatives_count | 0,044 | 0,60 | large |
| Pathotype | FTLD_vs_lvPPA_AD | long_pause_count | 0,044 | 0,40 | medium |
| Pathotype | svPPA_FTLD_vs_nfvPPA_FTLD | voyelles_oral_mean | 0,044 | 0,60 | large |
| Pathotype | svPPA_FTLD_vs_nfvPPA_FTLD | chroma_mean_5 | 0,044 | 0,60 | large |
| Pathotype | svPPA_FTLD_vs_nfvPPA_FTLD | b2 | 0,044 | 0,60 | large |
| Pathotype | svPPA_FTLD_vs_lvPPA_FTLD | delta_mfcc_mean_5 | 0,044 | 0,60 | large |
| Pathotype | FTLD_vs_lvPPA_AD | occlusives_percentile_10 | 0,044 | 0,40 | medium |
| Pathotype | FTLD_vs_lvPPA_AD | contrast_mean_2 | 0,045 | 0,41 | medium |
| Pathotype | svPPA_FTLD_vs_healthy_control | hnr_mean,1 | 0,045 | 0,40 | medium |
| Pathotype | amnestic_AD_vs_lvPPA_AD | voyelles_oral_std | 0,045 | 0,37 | medium |
| Pathotype | FTLD_vs_lvPPA_AD | semi_voyelles_min | 0,045 | 0,38 | medium |
| Pathotype | healthy_control_vs_amnestic_AD | semi_voyelles_percentile_10 | 0,046 | 0,24 | small |
| Pathotype | nfvPPA_FTLD_vs_lvPPA_AD | pause_proportion | 0,046 | 0,55 | large |
| Pathotype | nfvPPA_FTLD_vs_lvPPA_AD | occlusives_std | 0,046 | 0,55 | large |
| Pathotype | nfvPPA_FTLD_vs_amnestic_AD | chroma_mean_10 | 0,046 | 0,48 | medium |
| Pathotype | FTLD_vs_healthy_control | semi_voyelles_max | 0,046 | 0,29 | small |
| Pathotype | lvPPA_FTLD_vs_lvPPA_AD | pause_mean_duration | 0,046 | 0,55 | large |
| Pathotype | lvPPA_FTLD_vs_lvPPA_AD | voyelles_oral_cv | 0,046 | 0,55 | large |
| Pathotype | lvPPA_FTLD_vs_lvPPA_AD | voyelles_oral_percentile_90 | 0,046 | 0,55 | large |
| Pathotype | lvPPA_FTLD_vs_lvPPA_AD | f0_max | 0,046 | 0,55 | large |
| Pathotype | lvPPA_FTLD_vs_lvPPA_AD | delta_mfcc_mean_1 | 0,046 | 0,55 | large |
| Pathotype | lvPPA_FTLD_vs_lvPPA_AD | chroma_mean_1 | 0,046 | 0,55 | large |
| Pathotype | lvPPA_FTLD_vs_lvPPA_AD | contrast_mean_4 | 0,046 | 0,55 | large |
| Pathotype | lvPPA_FTLD_vs_lvPPA_AD | rolloff_std | 0,046 | 0,55 | large |
| Pathotype | nfvPPA_FTLD_vs_lvPPA_AD | chroma_mean_0 | 0,047 | 0,55 | large |
| Pathotype | svPPA_FTLD_vs_amnestic_AD | occlusives_percentile_90 | 0,047 | 0,42 | medium |
| Pathotype | nfvPPA_FTLD_vs_lvPPA_AD | contrast_mean_0 | 0,047 | 0,55 | large |
| Pathotype | lvPPA_FTLD_vs_lvPPA_AD | semi_voyelles_skewness | 0,047 | 0,50 | large |
| Pathotype | FTLD_vs_amnestic_AD | voyelles_oral_skewness | 0,048 | 0,32 | medium |
| Pathotype | healthy_control_vs_amnestic_AD | nasales_median | 0,048 | 0,24 | small |
| Pathotype | lvPPA_FTLD_vs_healthy_control | nasales_median | 0,048 | 0,46 | medium |
| Pathotype | FTLD_vs_amnestic_AD | chroma_mean_9 | 0,048 | 0,32 | medium |
| Pathotype | nfvPPA_FTLD_vs_healthy_control | f3 | 0,048 | 0,46 | medium |
| Pathotype | svPPA_FTLD_vs_lvPPA_AD | max_phoneme_duration | 0,048 | 0,49 | medium |
| Pathotype | svPPA_FTLD_vs_FTLD | occlusives_max | 0,048 | 0,45 | medium |
| Pathotype | svPPA_FTLD_vs_lvPPA_AD | skew_phoneme_duration | 0,048 | 0,49 | medium |
| Pathotype | svPPA_FTLD_vs_lvPPA_AD | fricatives_cv | 0,048 | 0,49 | medium |
| Pathotype | FTLD_vs_lvPPA_AD | contrast_mean_0 | 0,048 | 0,40 | medium |
| Pathotype | svPPA_FTLD_vs_lvPPA_AD | intensity_dynamic_range | 0,048 | 0,49 | medium |
| Pathotype | healthy_control_vs_amnestic_AD | f3_cv,1 | 0,049 | 0,24 | small |
| Pathotype | lvPPA_FTLD_vs_amnestic_AD | semi_voyelles_kurtosis | 0,049 | 0,47 | medium |
| Pathotype | svPPA_FTLD_vs_nfvPPA_FTLD | liquides_skewness | 0,049 | 0,59 | large |
| Pathotype | svPPA_FTLD_vs_nfvPPA_FTLD | liquides_kurtosis | 0,049 | 0,59 | large |
| Pathotype | lvPPA_FTLD_vs_amnestic_AD | f2 | 0,049 | 0,48 | medium |
| Pathotype | svPPA_FTLD_vs_FTLD | chroma_mean_11 | 0,049 | 0,45 | medium |
| Pathotype | svPPA_FTLD_vs_FTLD | f1 | 0,049 | 0,45 | medium |
| Pathotype | nfvPPA_FTLD_vs_amnestic_AD | hnr_std | 0,049 | 0,48 | medium |
| Pathotype | nfvPPA_FTLD_vs_FTLD | f2 | 0,049 | 0,51 | large |
| Pathotype | svPPA_FTLD_vs_FTLD | b2 | 0,049 | 0,45 | medium |
| Pathotype | FTLD_vs_lvPPA_FTLD | delta_mfcc_mean_5 | 0,049 | 0,51 | large |
| Pathotype | nfvPPA_FTLD_vs_amnestic_AD | voyelles_oral_max | 0,050 | 0,48 | medium |
| Pathotype | nfvPPA_FTLD_vs_amnestic_AD | chroma_mean_11 | 0,050 | 0,48 | medium |
| Pathotype | healthy_control_vs_lvPPA_AD | voyelles_oral_max | 0,050 | 0,34 | medium |
| Pathotype | FTLD_vs_amnestic_AD | chroma_mean_6 | 0,050 | 0,31 | medium |
| Pathotype | svPPA_FTLD_vs_amnestic_AD | chroma_mean_6 | 0,050 | 0,41 | medium |
| Pathotype | svPPA_FTLD_vs_healthy_control | fricatives_median | 0,050 | 0,39 | medium |
| Pathotype | nfvPPA_FTLD_vs_healthy_control | occlusives_max | 0,050 | 0,45 | medium |
| Pathotype | amnestic_AD_vs_lvPPA_AD | voyelles_oral_mean | 0,050 | 0,36 | medium |
| Pathotype | svPPA_FTLD_vs_healthy_control | voyelles_oral_max | 0,050 | 0,39 | medium |
| Pathotype | amnestic_AD_vs_lvPPA_AD | delta_mfcc_mean_7 | 0,050 | 0,36 | medium |
| Physiotype | healthy_vs_AD | contrast_mean_0 | 0,000 | 0,91 | large |
| Physiotype | FTLD_vs_healthy | contrast_mean_0 | 0,000 | 0,89 | large |
| Physiotype | FTLD_vs_healthy | contrast_mean_4 | 0,000 | 0,82 | large |
| Physiotype | healthy_vs_AD | zcr_mean | 0,000 | 0,76 | large |
| Physiotype | healthy_vs_AD | spectral_flux | 0,000 | 0,74 | large |
| Physiotype | healthy_vs_AD | spectral_flux,1 | 0,000 | 0,74 | large |
| Physiotype | healthy_vs_AD | fricatives_count | 0,000 | 0,73 | large |
| Physiotype | healthy_vs_AD | contrast_mean_1 | 0,000 | 0,69 | large |
| Physiotype | healthy_vs_AD | spectral_centroid_std | 0,000 | 0,66 | large |
| Physiotype | healthy_vs_AD | liquides_mean | 0,000 | 0,65 | large |
| Physiotype | healthy_vs_AD | contrast_mean_4 | 0,000 | 0,65 | large |
| Physiotype | healthy_vs_AD | rolloff_std | 0,000 | 0,64 | large |
| Physiotype | healthy_vs_AD | jitter_local | 0,000 | 0,62 | large |
| Physiotype | healthy_vs_AD | pause_mean_duration | 0,000 | 0,61 | large |
| Physiotype | FTLD_vs_healthy | fricatives_count | 0,000 | 0,63 | large |
| Physiotype | FTLD_vs_AD | spectral_flux,1 | 0,000 | 0,66 | large |
| Physiotype | healthy_vs_AD | std_phoneme_duration | 0,000 | 0,59 | large |
| Physiotype | healthy_vs_AD | pause_std_duration | 0,000 | 0,58 | large |
| Physiotype | healthy_vs_AD | inter_word_pause_std | 0,000 | 0,58 | large |
| Physiotype | healthy_vs_AD | spectral_centroid_mean | 0,000 | 0,58 | large |
| Physiotype | healthy_vs_AD | pause_max_duration | 0,000 | 0,58 | large |
| Physiotype | healthy_vs_AD | inter_word_pause_max | 0,000 | 0,58 | large |
| Physiotype | healthy_vs_AD | chroma_mean_8 | 0,000 | 0,58 | large |
| Physiotype | healthy_vs_AD | liquides_iqr | 0,000 | 0,57 | large |
| Physiotype | healthy_vs_AD | liquides_percentile_90 | 0,000 | 0,56 | large |
| Physiotype | healthy_vs_AD | pause_cv | 0,000 | 0,56 | large |
| Physiotype | FTLD_vs_healthy | syllable_count | 0,000 | 0,57 | large |
| Physiotype | FTLD_vs_healthy | voyelles_oral_count | 0,000 | 0,57 | large |
| Physiotype | healthy_vs_AD | phoneme_count | 0,000 | 0,56 | large |
| Physiotype | healthy_vs_AD | num_speech_phonemes | 0,000 | 0,56 | large |
| Physiotype | FTLD_vs_healthy | occlusives_median | 0,000 | 0,57 | large |
| Physiotype | FTLD_vs_healthy | pause_std_duration | 0,000 | 0,57 | large |
| Physiotype | FTLD_vs_healthy | inter_word_pause_std | 0,000 | 0,57 | large |
| Physiotype | healthy_vs_AD | jitter_rap | 0,000 | 0,55 | large |
| Physiotype | healthy_vs_AD | cv_phoneme_duration | 0,000 | 0,55 | large |
| Physiotype | FTLD_vs_healthy | occlusives_mean | 0,000 | 0,57 | large |
| Physiotype | healthy_vs_AD | inter_word_pause_mean | 0,000 | 0,54 | large |
| Physiotype | healthy_vs_AD | voyelles_oral_count | 0,000 | 0,54 | large |
| Physiotype | FTLD_vs_AD | spectral_centroid_std | 0,000 | 0,59 | large |
| Physiotype | FTLD_vs_healthy | pause_mean_duration | 0,000 | 0,55 | large |
| Physiotype | FTLD_vs_healthy | inter_word_pause_mean | 0,000 | 0,55 | large |
| Physiotype | FTLD_vs_healthy | phoneme_count | 0,000 | 0,55 | large |
| Physiotype | FTLD_vs_healthy | num_speech_phonemes | 0,000 | 0,55 | large |
| Physiotype | healthy_vs_AD | nasales_std | 0,000 | 0,53 | large |
| Physiotype | FTLD_vs_AD | rolloff_mean | 0,000 | 0,58 | large |
| Physiotype | healthy_vs_AD | occlusives_count | 0,000 | 0,53 | large |
| Physiotype | FTLD_vs_healthy | pause_ratio | 0,000 | 0,55 | large |
| Physiotype | healthy_vs_AD | pause_proportion | 0,000 | 0,53 | large |
| Physiotype | FTLD_vs_healthy | liquides_count | 0,000 | 0,54 | large |
| Physiotype | healthy_vs_AD | f1 | 0,000 | 0,52 | large |
| Physiotype | FTLD_vs_healthy | pause_proportion | 0,000 | 0,54 | large |
| Physiotype | healthy_vs_AD | pause_median_duration | 0,000 | 0,52 | large |
| Physiotype | healthy_vs_AD | inter_word_pause_median | 0,000 | 0,52 | large |
| Physiotype | FTLD_vs_healthy | articulation_rate | 0,000 | 0,51 | large |
| Physiotype | FTLD_vs_AD | spectral_centroid_mean | 0,000 | 0,57 | large |
| Physiotype | healthy_vs_AD | liquides_count | 0,000 | 0,52 | large |
| Physiotype | healthy_vs_AD | max_phoneme_duration | 0,000 | 0,52 | large |
| Physiotype | healthy_vs_AD | chroma_mean_7 | 0,000 | 0,51 | large |
| Physiotype | FTLD_vs_healthy | occlusives_count | 0,000 | 0,52 | large |
| Physiotype | FTLD_vs_AD | skew_phoneme_duration | 0,000 | 0,55 | large |
| Physiotype | healthy_vs_AD | occlusives_std | 0,000 | 0,49 | medium |
| Physiotype | healthy_vs_AD | rolloff_mean | 0,000 | 0,49 | medium |
| Physiotype | FTLD_vs_healthy | contrast_mean_3 | 0,000 | 0,51 | large |
| Physiotype | FTLD_vs_AD | kurt_phoneme_duration | 0,000 | 0,53 | large |
| Physiotype | healthy_vs_AD | pause_min_duration | 0,000 | 0,48 | medium |
| Physiotype | healthy_vs_AD | inter_word_pause_min | 0,000 | 0,48 | medium |
| Physiotype | healthy_vs_AD | liquides_median | 0,000 | 0,48 | medium |
| Physiotype | healthy_vs_AD | pause_ratio | 0,000 | 0,48 | medium |
| Physiotype | FTLD_vs_AD | cv_phoneme_duration | 0,000 | 0,53 | large |
| Physiotype | healthy_vs_AD | nasales_iqr | 0,000 | 0,48 | medium |
| Physiotype | FTLD_vs_AD | b1 | 0,000 | 0,53 | large |
| Physiotype | FTLD_vs_healthy | speech_duration | 0,000 | 0,49 | medium |
| Physiotype | FTLD_vs_AD | f0_mean | 0,000 | 0,52 | large |
| Physiotype | FTLD_vs_healthy | occlusives_percentile_90 | 0,000 | 0,49 | medium |
| Physiotype | FTLD_vs_healthy | nasales_median | 0,000 | 0,49 | medium |
| Physiotype | healthy_vs_AD | pause_total_duration | 0,000 | 0,47 | medium |
| Physiotype | healthy_vs_AD | jitter_ppq5 | 0,000 | 0,46 | medium |
| Physiotype | healthy_vs_AD | mean_phoneme_duration | 0,000 | 0,46 | medium |
| Physiotype | FTLD_vs_healthy | f3_cv,1 | 0,000 | 0,47 | medium |
| Physiotype | FTLD_vs_healthy | pause_median_duration | 0,000 | 0,47 | medium |
| Physiotype | FTLD_vs_healthy | inter_word_pause_median | 0,000 | 0,47 | medium |
| Physiotype | FTLD_vs_healthy | hnr_mean,1 | 0,000 | 0,47 | medium |
| Physiotype | FTLD_vs_healthy | nasales_count | 0,000 | 0,46 | medium |
| Physiotype | FTLD_vs_AD | max_phoneme_duration | 0,000 | 0,49 | medium |
| Physiotype | FTLD_vs_healthy | pause_max_duration | 0,000 | 0,46 | medium |
| Physiotype | FTLD_vs_healthy | inter_word_pause_max | 0,000 | 0,46 | medium |
| Physiotype | healthy_vs_AD | occlusives_mean | 0,000 | 0,44 | medium |
| Physiotype | FTLD_vs_AD | liquides_mean | 0,000 | 0,49 | medium |
| Physiotype | healthy_vs_AD | nasales_cv | 0,000 | 0,44 | medium |
| Physiotype | FTLD_vs_AD | zcr_mean | 0,000 | 0,48 | medium |
| Physiotype | FTLD_vs_AD | rolloff_std | 0,000 | 0,48 | medium |
| Physiotype | FTLD_vs_healthy | chroma_mean_2 | 0,000 | 0,45 | medium |
| Physiotype | healthy_vs_AD | speech_rate_phonemes_per_sec | 0,000 | 0,43 | medium |
| Physiotype | FTLD_vs_healthy | f1_cv,1 | 0,000 | 0,45 | medium |
| Physiotype | FTLD_vs_healthy | total_duration | 0,000 | 0,45 | medium |
| Physiotype | FTLD_vs_AD | spectral_instability,1 | 0,000 | 0,47 | medium |
| Physiotype | healthy_vs_AD | speech_duration | 0,000 | 0,43 | medium |
| Physiotype | healthy_vs_AD | voyelles_oral_percentile_90 | 0,000 | 0,43 | medium |
| Physiotype | healthy_vs_AD | nasales_mean | 0,000 | 0,43 | medium |
| Physiotype | FTLD_vs_healthy | shimmer_local | 0,000 | 0,44 | medium |
| Physiotype | FTLD_vs_healthy | f3_cv | 0,000 | 0,44 | medium |
| Physiotype | healthy_vs_AD | syllable_count | 0,000 | 0,42 | medium |
| Physiotype | FTLD_vs_AD | nasales_min | 0,000 | 0,46 | medium |
| Physiotype | FTLD_vs_AD | articulation_rate | 0,000 | 0,42 | medium |
| Physiotype | healthy_vs_AD | liquides_std | 0,000 | 0,42 | medium |
| Physiotype | FTLD_vs_AD | occlusives_kurtosis | 0,000 | 0,46 | medium |
| Physiotype | FTLD_vs_AD | liquides_median | 0,000 | 0,46 | medium |
| Physiotype | FTLD_vs_AD | occlusives_skewness | 0,000 | 0,46 | medium |
| Physiotype | FTLD_vs_AD | nasales_cv | 0,000 | 0,45 | medium |
| Physiotype | FTLD_vs_healthy | shimmer_apq5 | 0,000 | 0,42 | medium |
| Physiotype | FTLD_vs_AD | std_phoneme_duration | 0,000 | 0,45 | medium |
| Physiotype | FTLD_vs_healthy | nasales_mean | 0,000 | 0,41 | medium |
| Physiotype | FTLD_vs_healthy | contrast_mean_1 | 0,000 | 0,41 | medium |
| Physiotype | FTLD_vs_healthy | jitter_local | 0,000 | 0,41 | medium |
| Physiotype | healthy_vs_AD | num_inter_pauses | 0,000 | 0,40 | medium |
| Physiotype | FTLD_vs_healthy | intensity_dynamic_range | 0,000 | 0,41 | medium |
| Physiotype | FTLD_vs_AD | contrast_mean_1 | 0,000 | 0,43 | medium |
| Physiotype | healthy_vs_AD | spectral_flux_mean | 0,000 | 0,39 | medium |
| Physiotype | FTLD_vs_healthy | semi_voyelles_count | 0,000 | 0,40 | medium |
| Physiotype | FTLD_vs_healthy | intensity_std | 0,000 | 0,40 | medium |
| Physiotype | healthy_vs_AD | nasales_max | 0,000 | 0,39 | medium |
| Physiotype | FTLD_vs_healthy | syllable_rate_per_sec | 0,000 | 0,40 | medium |
| Physiotype | FTLD_vs_healthy | kurt_phoneme_duration | 0,000 | 0,40 | medium |
| Physiotype | healthy_vs_AD | occlusives_cv | 0,000 | 0,38 | medium |
| Physiotype | FTLD_vs_AD | spectral_flux | 0,000 | 0,42 | medium |
| Physiotype | healthy_vs_AD | f0_mean | 0,000 | 0,38 | medium |
| Physiotype | healthy_vs_AD | nasales_count | 0,000 | 0,38 | medium |
| Physiotype | FTLD_vs_healthy | intensity_mean | 0,000 | 0,39 | medium |
| Physiotype | healthy_vs_AD | occlusives_min | 0,001 | 0,37 | medium |
| Physiotype | FTLD_vs_healthy | rmse_mean | 0,001 | 0,39 | medium |
| Physiotype | FTLD_vs_AD | chroma_mean_2 | 0,001 | 0,41 | medium |
| Physiotype | healthy_vs_AD | num_inter_word_pauses | 0,001 | 0,37 | medium |
| Physiotype | healthy_vs_AD | f3 | 0,001 | 0,37 | medium |
| Physiotype | FTLD_vs_healthy | b1 | 0,001 | 0,38 | medium |
| Physiotype | healthy_vs_AD | nasales_kurtosis | 0,001 | 0,37 | medium |
| Physiotype | healthy_vs_AD | num_intra_word_pauses | 0,001 | 0,16 | small |
| Physiotype | healthy_vs_AD | voyelles_nasales_count | 0,001 | 0,16 | small |
| Physiotype | healthy_vs_AD | skew_phoneme_duration | 0,001 | 0,36 | medium |
| Physiotype | FTLD_vs_healthy | num_inter_pauses | 0,001 | 0,37 | medium |
| Physiotype | FTLD_vs_AD | occlusives_median | 0,001 | 0,40 | medium |
| Physiotype | FTLD_vs_AD | f0_std | 0,001 | 0,39 | medium |
| Physiotype | FTLD_vs_AD | liquides_std | 0,001 | 0,39 | medium |
| Physiotype | FTLD_vs_healthy | num_pauses | 0,001 | 0,36 | medium |
| Physiotype | FTLD_vs_healthy | num_inter_word_pauses | 0,001 | 0,36 | medium |
| Physiotype | FTLD_vs_healthy | spectral_flux | 0,001 | 0,36 | medium |
| Physiotype | healthy_vs_AD | nasales_percentile_90 | 0,001 | 0,35 | medium |
| Physiotype | FTLD_vs_healthy | fricatives_skewness | 0,001 | 0,36 | medium |
| Physiotype | FTLD_vs_AD | liquides_iqr | 0,001 | 0,38 | medium |
| Physiotype | healthy_vs_AD | semi_voyelles_min | 0,001 | 0,34 | medium |
| Physiotype | FTLD_vs_healthy | delta_mfcc_mean_10 | 0,001 | 0,36 | medium |
| Physiotype | FTLD_vs_healthy | chroma_mean_5 | 0,002 | 0,36 | medium |
| Physiotype | FTLD_vs_AD | contrast_mean_3 | 0,002 | 0,38 | medium |
| Physiotype | FTLD_vs_healthy | pause_min_duration | 0,002 | 0,35 | medium |
| Physiotype | FTLD_vs_healthy | inter_word_pause_min | 0,002 | 0,35 | medium |
| Physiotype | healthy_vs_AD | long_pause_count | 0,002 | 0,34 | medium |
| Physiotype | FTLD_vs_healthy | liquides_min | 0,002 | 0,35 | medium |
| Physiotype | FTLD_vs_healthy | fricatives_kurtosis | 0,002 | 0,35 | medium |
| Physiotype | FTLD_vs_healthy | chroma_mean_9 | 0,002 | 0,35 | medium |
| Physiotype | FTLD_vs_healthy | shimmer_apq3 | 0,002 | 0,35 | medium |
| Physiotype | FTLD_vs_healthy | shimmer_dda | 0,002 | 0,35 | medium |
| Physiotype | FTLD_vs_AD | f0_max | 0,002 | 0,37 | medium |
| Physiotype | healthy_vs_AD | delta_mfcc_mean_10 | 0,002 | 0,34 | medium |
| Physiotype | FTLD_vs_healthy | nasales_percentile_10 | 0,002 | 0,34 | medium |
| Physiotype | healthy_vs_AD | delta_mfcc_mean_2 | 0,002 | 0,34 | medium |
| Physiotype | FTLD_vs_healthy | semi_voyelles_min | 0,002 | 0,33 | medium |
| Physiotype | FTLD_vs_healthy | hnr_mean | 0,002 | 0,35 | medium |
| Physiotype | FTLD_vs_healthy | fricatives_max | 0,002 | 0,34 | medium |
| Physiotype | healthy_vs_AD | liquides_min | 0,002 | 0,32 | medium |
| Physiotype | FTLD_vs_AD | chroma_mean_8 | 0,003 | 0,36 | medium |
| Physiotype | FTLD_vs_AD | f1 | 0,003 | 0,36 | medium |
| Physiotype | healthy_vs_AD | occlusives_kurtosis | 0,003 | 0,33 | medium |
| Physiotype | FTLD_vs_AD | f3_cv,1 | 0,003 | 0,35 | medium |
| Physiotype | FTLD_vs_healthy | nasales_min | 0,003 | 0,33 | medium |
| Physiotype | FTLD_vs_healthy | rolloff_mean | 0,003 | 0,33 | medium |
| Physiotype | FTLD_vs_healthy | jitter_rap | 0,003 | 0,33 | medium |
| Physiotype | FTLD_vs_healthy | f1_cv | 0,004 | 0,33 | medium |
| Physiotype | FTLD_vs_AD | fricatives_std | 0,004 | 0,35 | medium |
| Physiotype | FTLD_vs_AD | chroma_mean_7 | 0,004 | 0,35 | medium |
| Physiotype | healthy_vs_AD | semi_voyelles_kurtosis | 0,004 | 0,31 | medium |
| Physiotype | healthy_vs_AD | syllable_rate_per_sec | 0,004 | 0,31 | medium |
| Physiotype | FTLD_vs_AD | long_pause_count | 0,004 | 0,34 | medium |
| Physiotype | FTLD_vs_healthy | skew_phoneme_duration | 0,004 | 0,32 | medium |
| Physiotype | healthy_vs_AD | semi_voyelles_mean | 0,004 | 0,31 | medium |
| Physiotype | healthy_vs_AD | rate_speech_phonemes | 0,004 | 0,31 | medium |
| Physiotype | healthy_vs_AD | semi_voyelles_count | 0,005 | 0,31 | medium |
[truncated: 287,353 more chars]
