# Supplementary material for: Speech-based digital biomarkers for early etiological stratification of Alzheimer’s disease and frontotemporal degeneration: a biomarker-confirmed prospective study
Source: J Prev Alzheimers Dis. 2026 Apr 17;13(6):100573. doi: 10.1016/j.tjpad.2026.100573 (PMC13098405; doi:10.1016/j.tjpad.2026.100573)
Supplement: Supplementary file 5 [file mmc5.docx]

**Supplementary Material 5 : Demographic Confounding Analyses**

# Overview

To assess whether classification performance could be driven by demographic differences across diagnostic groups rather than by pathology-specific speech alterations, three complementary analyses were conducted on the held-out test set after model development was complete:

1. Spearman correlations between age and top discriminative features within each diagnostic group (FDR correction).
2. Logistic regression with and without age as a covariate to assess coefficient stability.
3. Mann–Whitney U tests comparing top features between male and female participants within each diagnostic group (FDR correction).

# Summary of demographic bias analyses for Physiotype

| **Task** | **Age-feature pairs (FDR)** | **Acc M1** | **Acc M2 (+age)** | **Age β** | **Sex-feature pairs (FDR)** |
| --- | --- | --- | --- | --- | --- |
| NEG | 0 / 30 | 0.922 | 0.92 | -0.17 | 0 / 30 |
| POS | 0 / 30 | 0.78 | 0.82 | -0.27 | 0 / 30 |
| SST | 0 / 30 | 0.72 | 0.72 | -0.11 | 0 / 30 |
| MPT | 0 / 30 | 0.90 | 0.90 | 0.013 | 0 / 30 |

*FDR: False Discovery Rate (Benjamini–Hochberg). 30 pairs = 10 top features × 3 diagnostic groups (FTLD, AD and healthy controls). M1: logistic regression on top speech features only; M2: model adding age as a covariate. Δ coef: mean relative change in feature coefficients between M1 and M2. Age β: standardised age coefficient in M2.*

# Summary of demographic bias analyses for Pathotype

| **Task** | **Age-feature pairs (FDR)** | **Acc M1** | **Acc M2 (+age)** | **Age β** | **Sex-feature pairs (FDR)** |
| --- | --- | --- | --- | --- | --- |
| NEG | 0 / 70 | 0.922 | 0.902 | 0.08 | 0 / 70 |
| POS | 0 / 70 | 0.776 | 0.816 | −0.05 | 0 / 70 |
| SST | 0 / 70 | 0.724 | 0.760 | 0.09 | 0 / 70 |
| MPT | 0 / 70 | 0.897 | 0.887 | 0.25 | 0 / 70 |

*FDR: False Discovery Rate (Benjamini–Hochberg). 70 pairs = 10 top features × 7 diagnostic groups (FTLD, amnestic-AD, healthy control, lvPPA-AD, lvPPA-FTLD, nfvPPA-FTLD, svPPA-FTLD). M1: logistic regression on top speech features only; M2: model adding age as a covariate. Δ coef: mean relative change in feature coefficients between M1 and M2. Age β: standardised age coefficient in M2.*
