# Supplementary material for: Speech-based digital biomarkers for early etiological stratification of Alzheimer’s disease and frontotemporal degeneration: a biomarker-confirmed prospective study
Source: J Prev Alzheimers Dis. 2026 Apr 17;13(6):100573. doi: 10.1016/j.tjpad.2026.100573 (PMC13098405; doi:10.1016/j.tjpad.2026.100573)

**Supplementary Table 6 : Confusion matrices of physiotype and pathotype independent and hierarchical classification of best models**

1. **Physiotype confusion matrices**


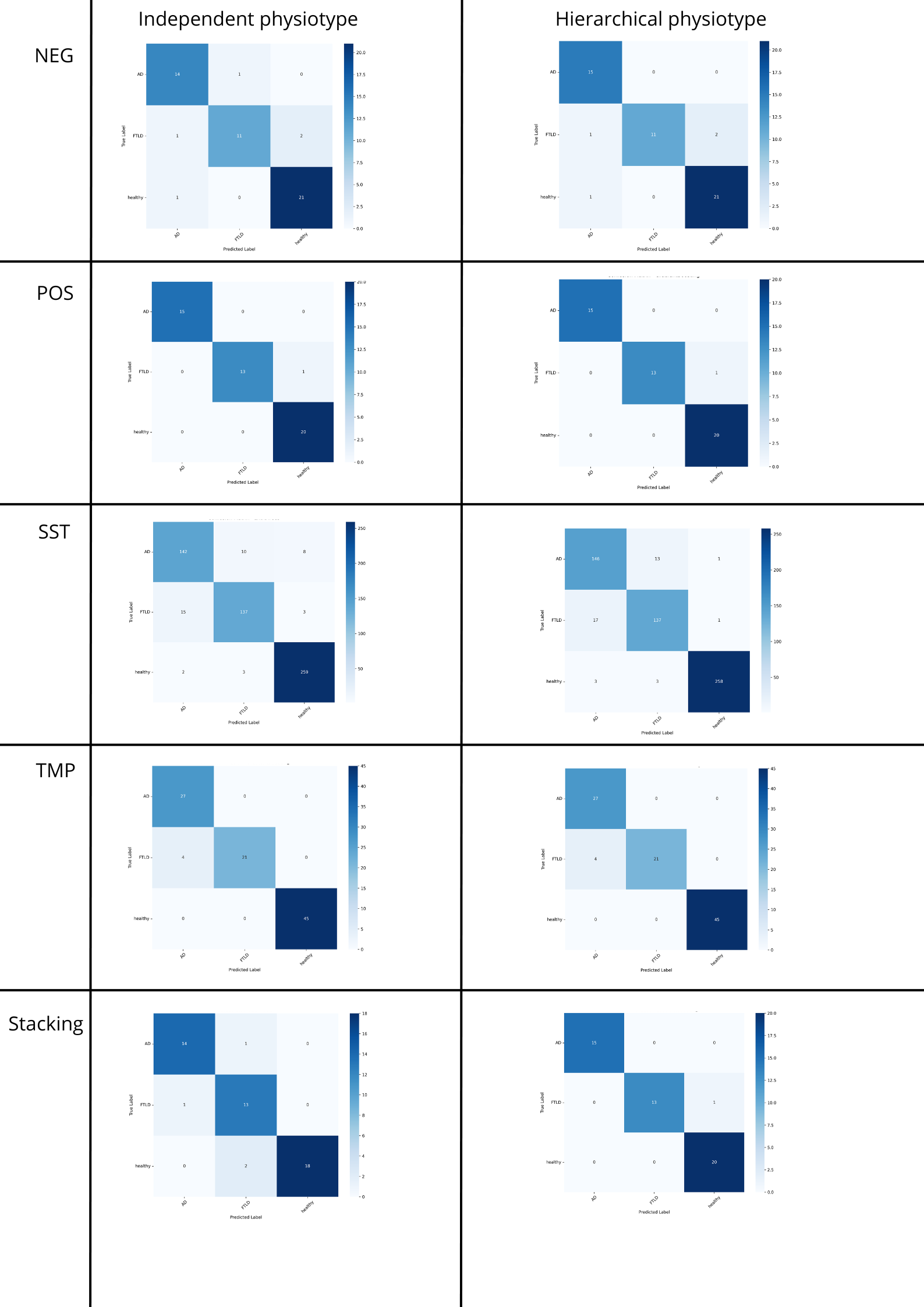


1. **Pathotype confusion matrices**


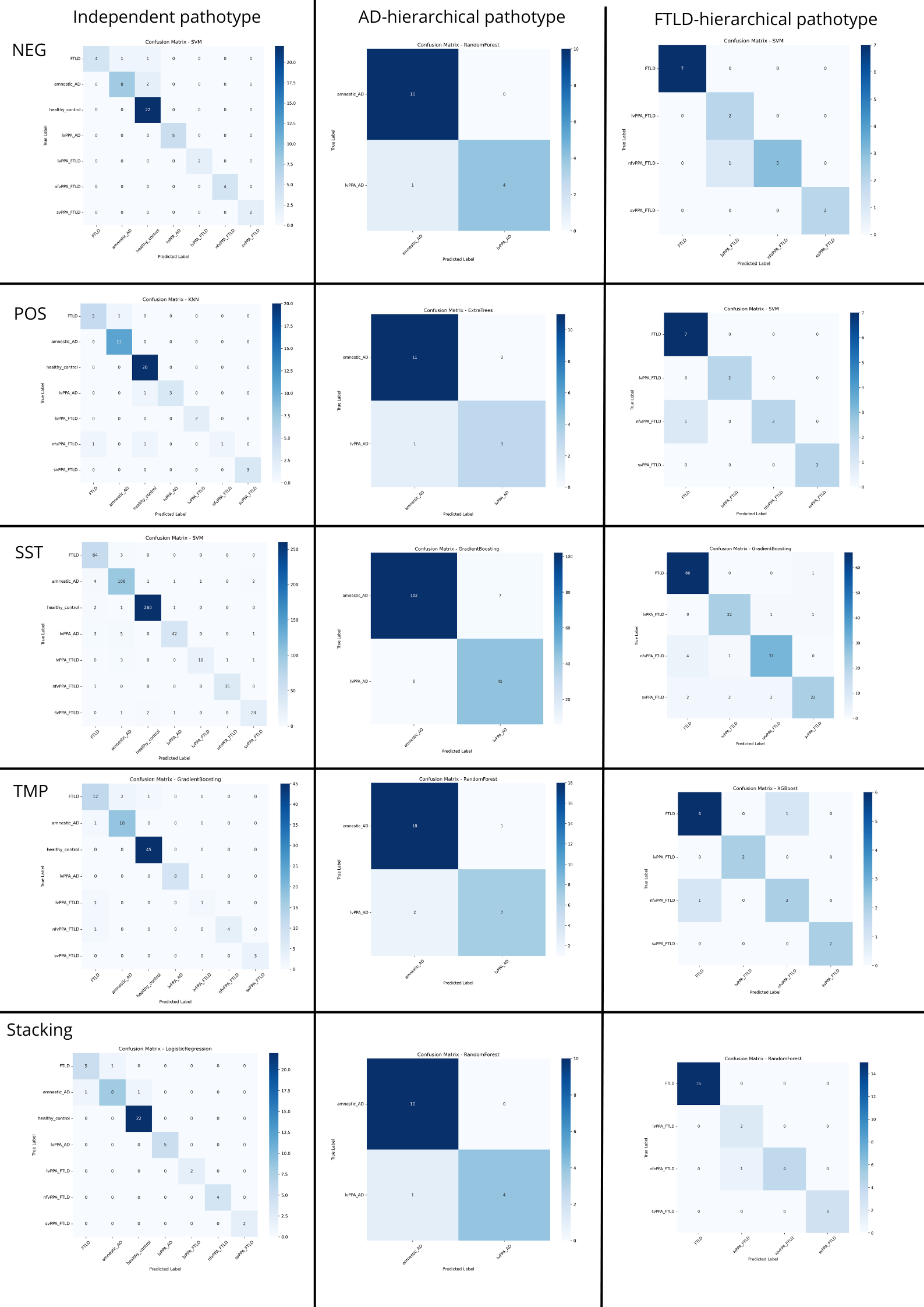

Supplement: Supplementary file 6 [file mmc6.docx]
